# Transition Metal‐Free Regio‐ and Stereo‐Selective *trans* Hydroboration of 1,3‐Diynes: A Phosphine‐Catalyzed Access to (*E*)‐1‐Boryl‐1,3‐Enynes

**DOI:** 10.1002/chem.202202349

**Published:** 2022-09-01

**Authors:** Swetha Jos, Connor Szwetkowski, Carla Slebodnick, Robert Ricker, Ka Lok Chan, Wing Chun Chan, Udo Radius, Zhenyang Lin, Todd B. Marder, Webster L. Santos

**Affiliations:** ^1^ Department of Chemistry Virginia Tech Blacksburg Virginia United States; ^2^ Institute of Inorganic Chemistry Institute for Sustainable Chemistry & Catalysis with Boron Julius-Maximilians-Universität Würzburg Germany; ^3^ Department of Chemistry The Hong Kong University of Science and Technology Clear Water Bay Kowloon Hong Kong SAR China

**Keywords:** enediyne, enyne, hydroboration, organocatalytic, stereoselective

## Abstract

We report a transition metal‐free, regio‐ and stereo‐selective, phosphine‐catalyzed method for the *trans* hydroboration of 1,3‐diynes with pinacolborane that affords (*E*)‐1‐boryl‐1,3‐enynes. The reaction proceeds with excellent selectivity for boron addition to the external carbon of the 1,3‐diyne framework as unambiguously established by NMR and X‐ray crystallographic studies. The reaction displays a broad substrate scope including unsymmetrical diynes to generate products in high yield (up to 95 %). Experimental and theoretical studies suggest that phosphine attack on the alkyne is a key process in the catalytic cycle.

## Graphical abstract

A transition metal‐free method for the *trans‐*hydroboration of 1,3‐diynes to afford (*E*)‐1‐boryl‐1,3‐enynes is reported. Boron adds to the external carbon of the diyne framework. Experimental and DFT studies suggest that phosphine attack on the diyne is a key step in the catalytic cycle.

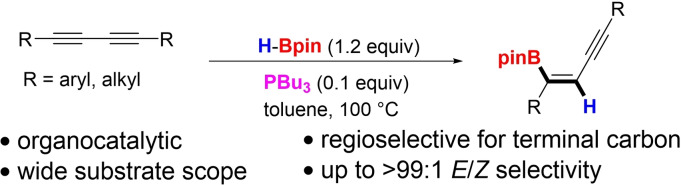

## Introduction

Organocatalysis has emerged as a fast‐growing alternative to transition metal catalysis^[1]^ due to cost‐effectiveness, low toxicity, ready availability, low sensitivity to moisture and air, and minimization of potential metal contamination especially for the production of food and pharmaceutical products. As a functional group handle, organoboron compounds are versatile and attractive due to myriad transformations they can undergo,^[2]^ with the Suzuki‐Miyaura^[3]^ and Chan‐Lam coupling^[4]^ being prominent examples. Thus, organocatalytic methods for borylation of organic compounds that afford high value commodity materials are needed. Diynes are molecules characterized by their rod‐like structure with extended π conjugation^[5]^ and are important precursors in the synthesis of various natural products,^[6]^ biologically active molecules,^[7]^ polymers,^[8]^ and supramolecular systems.^[9]^ Selective borylation of 1,3‐diynes provides access to organoboron transformations^[10]^ and facile medicinal chemistry derivations, for example, to generate enediynes, which are a class of antibiotic and anti‐cancer agents.[^5b^, ^11^]

**Scheme 1:**
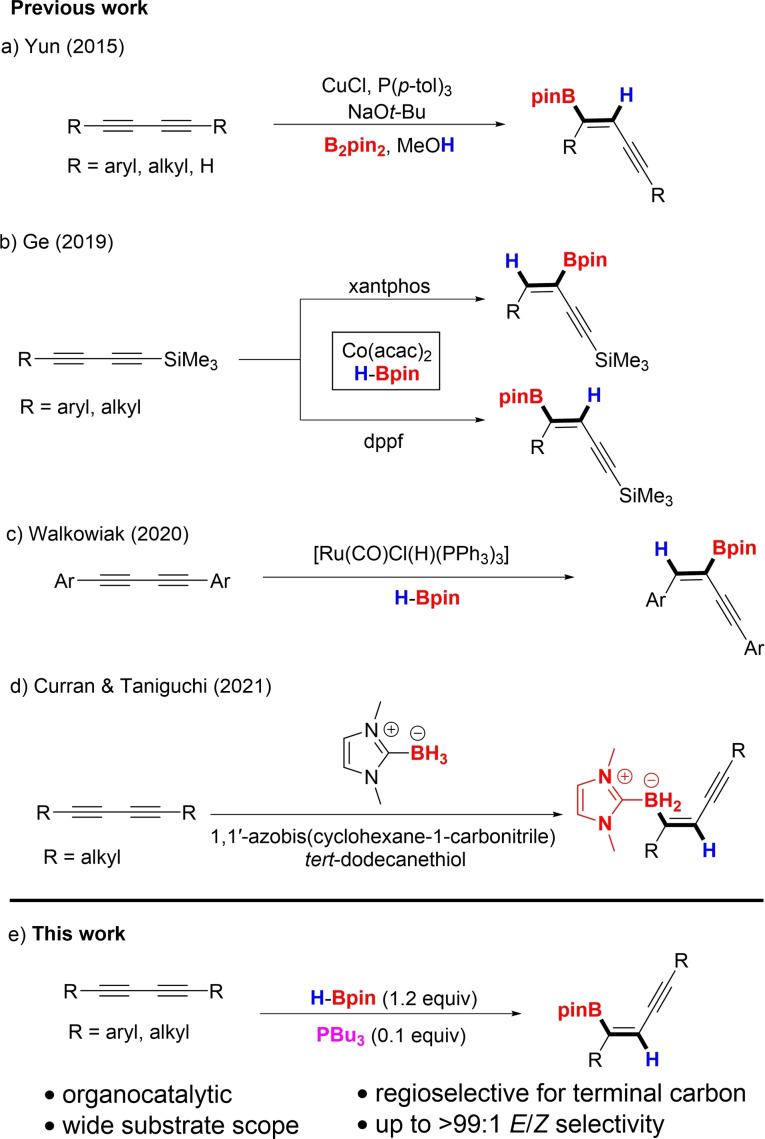
Strategies for hydroboration of 1,3‐diynes.

However, methods for borylating 1,3‐diynes are scarce^[12]^ and protocols often use metal catalysts.[^5b^, ^13^] In 2015, Yun and co‐workers developed a method for the *cis* hydroboration of diynes, placing a boryl group on the external carbon using CuCl as the catalyst (Scheme 1a).^[14]^ Interestingly, hydroboration with HBpin catalyzed by Co(acac)_2_ in the presence of xantphos or dppf afforded borylated enynes wherein boron added to the internal or external carbon of the 1,3‐diyne unit, respectively (Scheme 1b).^[15]^ In both cases, the isolated products were derived from *cis* addition. In 2020, Walkowiak et al. reported a ruthenium‐based method for transforming 1,3‐diynes to *cis* borylated enynes in which boron was installed at the internal carbon (Scheme 1c).^[16]^ However, this method is restricted to aryl‐substituted 1,3‐diynes. Recently, a *trans* hydroboration process was achieved using an *N*‐heterocyclic carbene borane adduct^[17]^ via radical borylation with an initiator (ACCN, 1,1‐azobis(cyclohexane‐1‐carbonitrile)) (Scheme 1d). However, this method is limited to alkyl substrates. Previous work from our groups has established a platinum catalyzed diboration of diynes^[18]^ as well as a phosphine catalyzed *trans* hydroboration of alkynoic acid derivatives^[19]^ and propiolonitriles.^[20]^ Because of the limitations of existing methods and our interest in the *trans* borylation of substituted alkynes, our laboratories focused on an organocatalytic process for hydroboration of 1,3‐diynes. In this report, we disclose a method for the borylation of 1,3‐diynes with pinacol borane using tri‐*n*‐butyl phosphine (*n*‐Bu_3_P) as the catalyst to synthesize previously elusive (*E*)‐1‐boryl‐1,3‐enynes. Notably, the boron moiety is installed on the external carbon of the 1,3‐diyne framework in a *trans* fashion.

## Results and Discussion

We initiated our studies with diphenyl butadiyne (**1 a**) as a model substrate (Table 1). When the reaction was performed with pinacol borane (H‐Bpin, 2.0 equiv) in toluene at 100 °C, no product was formed (entry 1). While the addition of a catalytic amount of *n*‐Bu_3_P at room temperature resulted in a trace amount of **2 a**, heating to 70 °C afforded 80 % conversion (entries 2–3). Further increasing the temperature to 100 °C significantly improved the conversion and *E*/*Z* selectivity to 98 : 2 (entry 4). To assign the stereochemistry, **2 a** was protodeborylated with AgF^[21]^ to give enyne **3 a** wherein the coupling constant of 16.2 Hz from the alkene protons is consistent with an *E* double bond geometry. The conversion to the corresponding *Z* isomer from (*Z*)**‐2 a** was also consistent with literature data.^[14]^ Furthermore, the GC/MS retention times compared from authentic samples also confirm *E* and *Z* isomers (see Supporting Information). With the stereochemistry assigned, we continued our optimization with various solvents. Changing the solvent to THF or acetonitrile reduced the conversion or stereoselectivity (entries 5–6). We then investigated the effect of catalyst. Among the phosphine catalysts screened, PPh_3_ slightly decreased conversion while PMe_3_ and PCy_3_ decreased the yield and stereoselectivity (entries 7–9). Optimization of the amount of HBpin indicated that 1.2 equivalents is sufficient for complete conversion (entries 10–11). As adventitious *in situ* borane^[22]^ (BH_3_) can be generated from ‘hidden boron catalysis’,^[23]^ we performed the reaction in the presence of BH_3_ (from a commercial source) both with (entry 12) and without the phosphine catalyst (entry 13). Furthermore, we ran the reaction with TMEDA (used to quench BH_3_) (entry 14).^[23]^ There was only trace product formation without the phosphine catalyst (entry 13), and the reaction proceeded to 97 % product formation even in the presence of TMEDA, therefore suggesting that *in situ* generated BH_3_ does not catalyze the reaction. To understand the effect of additive amount on the reaction outcome, we performed the reaction with 1.0 equiv of *n*‐Bu_3_P and observed 100 % conversion with a slight reduction in *E*/*Z* selectivity (entry 15). Decreasing the amount of *n*‐Bu_3_P to 0.05 equiv reduced the conversion of **1 a** to the products (entry 16). Finally, because PPh_3_ was an efficient catalyst (entry 7) and potentially a more accessible reagent, we used a more challenging substrate (*p*‐methoxy‐substituted **1 g**) and observed trace product formation (entry 17). Therefore, we proceeded with *n*‐Bu_3_P as the optimal catalyst and entry 10 as our optimized set of reaction conditions.

**Table 1:** Optimization of the reaction conditions.^[a]^

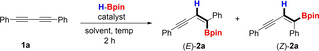					
Entry	H‐Bpin (equiv)	Catalyst (0.1 equiv)	Solvent	Temp (°C)	Conversion (*E/Z*)^[b]^
1	2	–	toluene	100	trace
2	2	*n*‐Bu_3_P	toluene	rt	trace
3	2	*n*‐Bu_3_P	toluene	70	80 (94 : 6)
4	2	*n*‐Bu_3_P	toluene	100	100 (98 : 2)
5	2	*n*‐Bu_3_P	THF	70	40 (90 : 10)
6	2	*n*‐Bu_3_P	MeCN	70	100 (86 : 14)
7	2	PPh_3_	toluene	100	82 (98 : 2)
8	2	PCy_3_	toluene	100	4 (66 : 34)
9	2	PMe_3_	toluene	100	7 (77 : 23)
10	1.2	*n*‐Bu_3_P	toluene	100	100 (97 : 3)
11	1.0	*n*‐Bu_3_P	toluene	100	77 (97 : 3)
12^[c]^	1.2	*n*‐Bu_3_P	toluene	100	100 (97 : 3)
13^[c]^	1.2	–	toluene	100	trace
14^[d]^	1.2	*n*‐Bu_3_P	toluene	100	93 (97 : 3)
15^[e]^	1.2	*n*‐Bu_3_P	toluene	100	100 (95 : 5)
16^[f]^	1.2	*n*‐Bu_3_P	toluene	100	65 (97 : 3)
17 ^[g]^	2	PPh_3_	toluene	100	trace

^[a]^ Reaction conditions: **1 a** (0.1 mmol), catalyst (0.01 mmol), HBpin (0.12 mmol), solvent (0.5 mL, 0.2 M), 2 h. ^[b]^ Based on GC‐MS analysis. ^[c]^ With BH_3_. ^[d]^ 0.1 equiv of TMEDA.^[e]^ 1.0 equiv of *n*‐Bu_3_P. ^[f]^ 0.05 equiv of *n*‐Bu_3_P. ^[g]^ *Para*‐methoxy substrate **1 g** instead of **1 a**.

With optimized reaction conditions in hand, we surveyed the scope and limitation of the reaction. We first investigated electron donating groups on the aryl ring (Table 2). With model substrate **1 a**, the corresponding enyne borylated product **2 a** was isolated in 78 % yield, 97 : 3 (*E*/*Z*) selectivity, and no detected internal borylation product. Fortunately, increasing the scale of the reaction to 2 mmol had no effect on selectivity and the product was isolated in 62 % yield. Introduction of a methyl group at the *ortho*‐, *meta*‐, or *para*‐position of the aryl ring (**2 b**–**2 d**) resulted in good yields and selectivity. Increasing the steric bulk from a propyl to a *tert*‐butyl group likewise afforded **2 e** and **2 f**, respectively, in good yields and selectivity. Interestingly, when a methoxy group is placed closer to the reaction center (i. e., *para*→*meta*→*ortho*, **2 g**–**2 i**), a corresponding decrease in *E*/*Z* selectivity was observed, suggesting the sensitivity of the reaction to steric effects.^[19b]^ Other electron donating groups such as thioether **1 k** and amine **1 l** served as efficient substrates affording the products **2 k**–**2 l** in good yields and excellent *E*/*Z* selectivity. For substrates **1 g**, **1 j**, **1 k**, and **1 l**, we observed a decrease in **2 : 2‘** regioselectivity. Fortunately, further optimization revealed acetonitrile to be an efficient solvent. Phenyl substitution (**2 m**) at the 4‐position was tolerated whereas a larger naphthyl group (**2 n**) slightly decreased the *E*/*Z* selectivity while maintaining excellent regioselectivity for the external carbon of the 1,3‐diyne framework. We next investigated the effect of electron‐withdrawing substituents on the aryl ring. For these substrates, the borylation reaction proceeded rapidly and conversion of the starting material was complete within 1 h. In general, there is a slight decrease in *E*/*Z* selectivity while the overall yields remained consistent, and exclusive formation of the external borylation product **2** was observed. For example, acetophenone derivative **2 o** and methylester **2 p** were afforded in approximately 50 % yield and 80–90 % preference for the *E*‐isomer. Fluorine‐containing substrates such as trifluoromethyl (**2 q**), trifluoromethoxy (**2 r**), and fluoro (**2 s**) were produced in good to excellent yields and 90 : 10 *E*/*Z* selectivity. Decoration of the phenyl ring with chlorine at various positions (**2 t**–**2 v**) confirmed the sensitivity of the reaction to steric effects as the *ortho‐*chloro substituted product **2 t** had 67 : 33 *E*/*Z* selectivity. Among the halogens, 4‐bromo derivative **2 w** gave the lowest selectivity and we encountered difficulty in separating isomers. 1,3‐Diynes substituted with heterocycles, such as thiophene (**2 x**) and pyridine (**2 y**), were tolerated with yields of 63 % and 47 %, respectively, and up to >99 *E* selectivity.

**Table 2:** Substrate scope.^[a]^

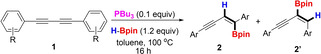				
Compound	R	Yield (%)^[b]^	**2** (*E*/*Z*) ^[c]^	**2** : **2’**
**2 a**	4‐H	78	97 : 3	>99 : 1
	(2 mmol scale)	62	97 : 3	>99 : 1
**2 b**	4‐CH_3_	66	92 : 8	>99 : 1
**2 c**	3‐CH_3_	71	91 : 9	>99 : 1
**2 d**	2‐CH_3_	59	86 : 14	>99 : 1
**2 e**	4‐*n*‐propyl	66	90 : 10	>99 : 1
**2 f**	4‐*t*‐butyl	50	90 : 10	>99 : 1
**2 g** ^[d]^	4‐OCH_3_	65	>99 : 1	>99 : 1
**2 h**	3‐OCH_3_	65	90 : 10	>99 : 1
**2 i**	2‐OCH_3_	70	75 : 25	>99 : 1
**2 j** ^[d]^	4‐OCH_2_CH_3_	78	>99 : 1	98 : 2
**2 k** ^[d]^	4‐SCH_3_	63	>99 : 1	>99 : 1
**2 l** ^[d]^	4‐N(CH_3_)_2_	50	>99 : 1	>99 : 1
**2 m**	4‐Ph	59	>99 : 1	97 : 3
**2 n** ^[e]^	2‐naphthyl	73	87 : 13	>99 : 1
**2 o**	4‐C(O)CH_3_	48	80 : 20	>99 : 1
**2 p**	4‐CO_2_CH_3_	53	90 : 10	>99 : 1
**2 q**	4‐CF_3_	84	89 : 11	>99 : 1
**2 r**	4‐OCF_3_	95	90 : 10	>99 : 1
**2 s**	4‐F	68	90 : 10	>99 : 1
**2 t**	2‐Cl	65	67 : 33	>99 : 1
**2 u**	3‐Cl	61	90 : 10	>99 : 1
**2 v**	4‐Cl	57	84 : 16	>99 : 1
**2 w**	4‐Br	62	60 : 40	>99 : 1
**2 x**	3‐thiophenyl	63	92 : 8	>99 : 1
**2 y**	2‐pyridyl	47	>99 : 1	>99 : 1

^[a]^ Reaction conditions: **1** (0.25 mmol), *n*‐Bu_3_P (0.025 mmol), HBpin (0.3 mmol), solvent, 16 h, 100 °C. ^[b]^ Isolated yields. ^[c]^ *E*/*Z* ratios based on GC‐MS. ^[d]^ MeCN as solvent. ^[e]^ 3 h and *E*/*Z* ratio based on ^1^H NMR.

**Figure 1:**
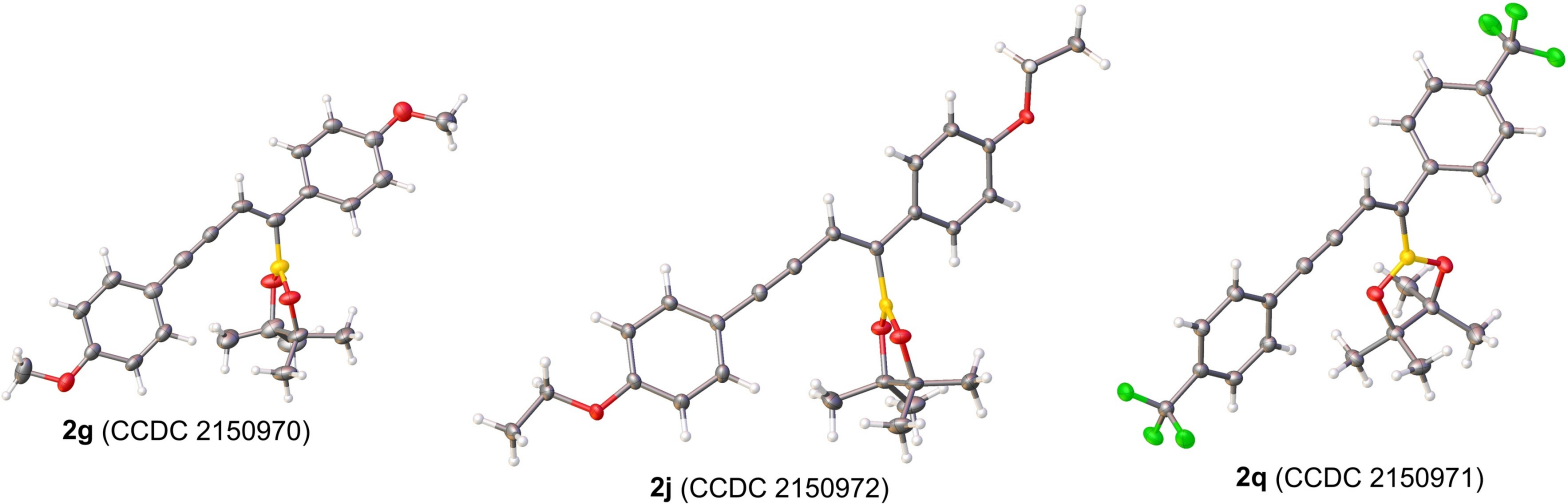
Molecular structures of enyne boronates **2 g**, **2 j**, and **2 q**.

To assign unambiguously the structure of the product enyne boronates, we attempted to cystallize several compounds for single crystal X‐ray diffraction studies. Fortunately, compounds bearing electron donating methoxy (**2 g**) and ethoxy (**2 j**) as well as electron withdrawing trifluoromethyl (**2 q**) groups yielded crystals suitable for analysis. As shown in Figure 1, the Bpin moiety was installed on the external carbon of the 1,3‐diyne framework with the alkynyl carbon positioned in a *cis* fashion relative to boron.

**Scheme 2:**
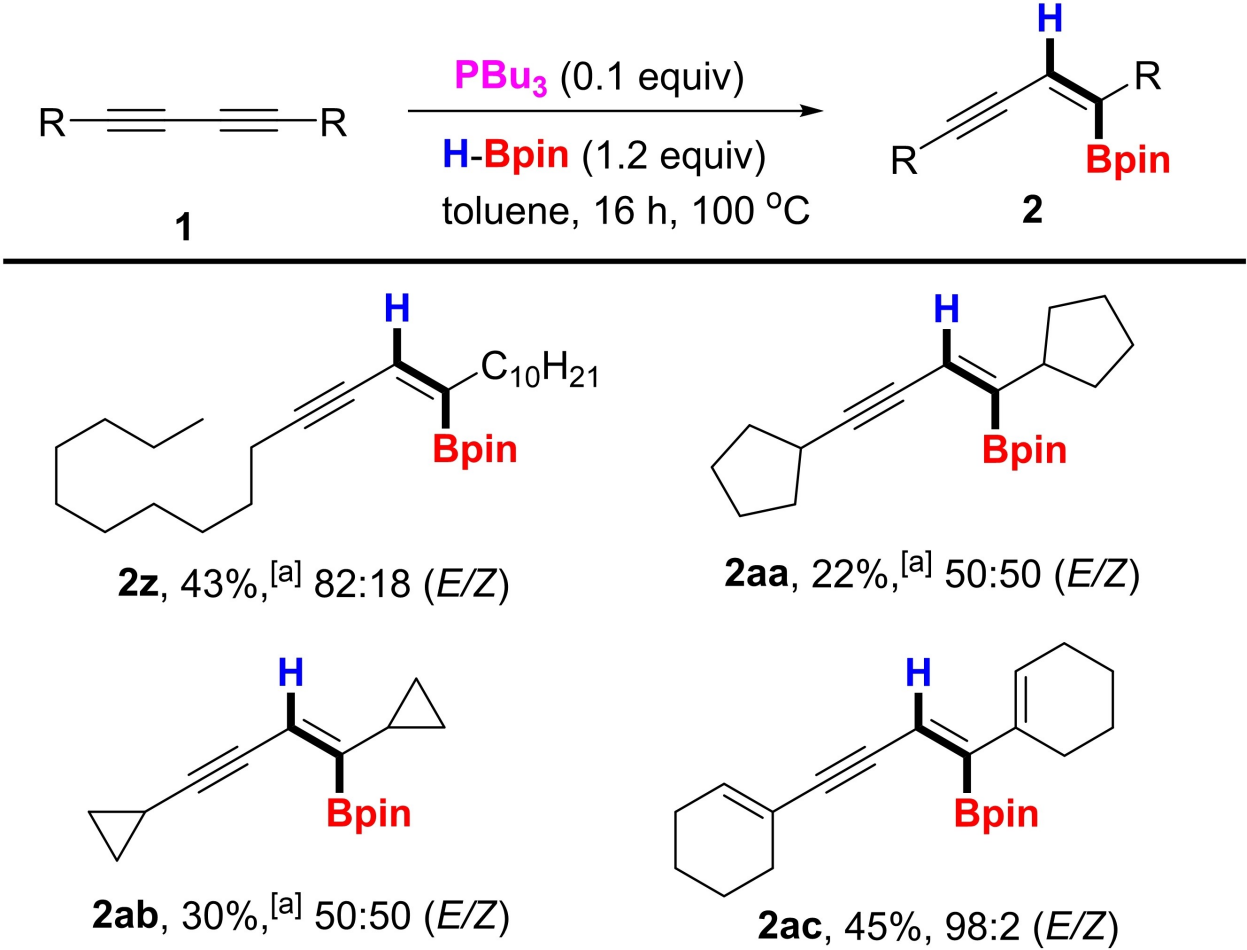
Substrate scope. Reaction conditions: **1** (0.25 mmol), n‐Bu_3_P (0.025 mmol), HBpin (0.3 mmol), solvent, 16 h, 100 °C. ^[a]^ 24 h. Isolated yields are reported and *E*/*Z* ratios based on GC‐MS.

**Scheme 3:**
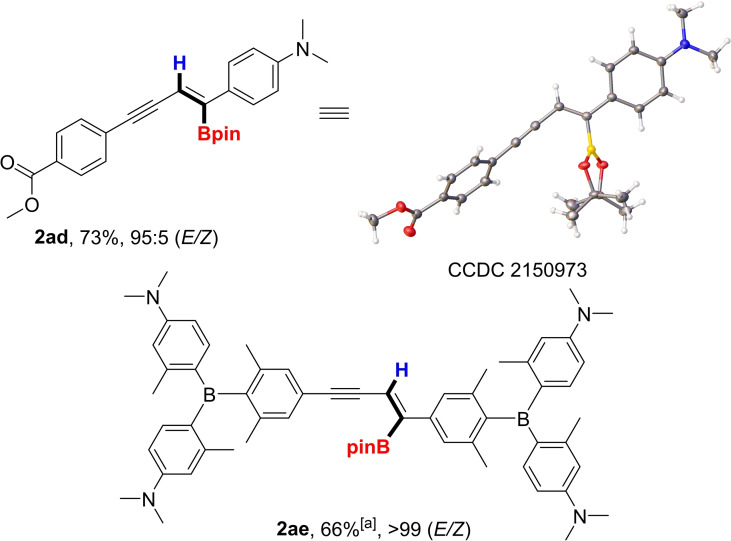
Substrate scope. Reaction conditions: **1** (0.25 mmol), n‐Bu_3_P (0.025 mmol), HBpin (0.3 mmol), solvent, 16 h, 100 °C. ^[a]^ *E*/*Z* ratio based on ^1^H NMR. Isolated yields are reported and *E*/*Z* ratios based on GC‐MS.

The utility of the new protocol prompted us to investigate the efficiency of the reaction conditions with alkyl substituted substrates (Scheme 2). While decyl (**1 z**), cyclopentyl (**1 aa**) and cyclopropyl (**1 ab**) diynes were converted to the corresponding enyne boronates **2 z** and **2 aa**–**2 ab**, the yields were moderate and selectivity was poor. These substrates suffered from low conversion and the presence of unreacted starting material as well as the *Z*‐isomer led to difficulties in purification. However, with the highly conjugated dicylohexenyldiyne **1 ac**, *E*/*Z*‐selectivity proved to be excellent with a yield of 45 % of **2 ac**, presumably because the alkenyl substituents provided similar electronic effects to those of aryl rings. Furthermore, we examined the regioselectivity of hydroboration with an unsymmetrical substrate bearing donor and acceptor moieties (Scheme 3). Thus, treatment of **1 ad**, a substrate bearing dimethylamino and methyl ester moieties on opposite ends of the diyne scaffold, afforded **2 ad** in 73 % yield with 95 : 5 *E*/*Z* selectivity. The regiochemistry observed in the product, which was confirmed by X‐ray crystallography, indicates that the reaction occurred on the external alkyne nearest the donor amine group. Finally, we investigated a fluorometric and Raman probe **1 ae**, a compound bearing 2 boron units used for simultaneous and selective sensing of various DNA, RNA, and proteins,^[5e]^ and **2 ae** was afforded in 66 % yield with excellent selectivity.

**Scheme 4:**
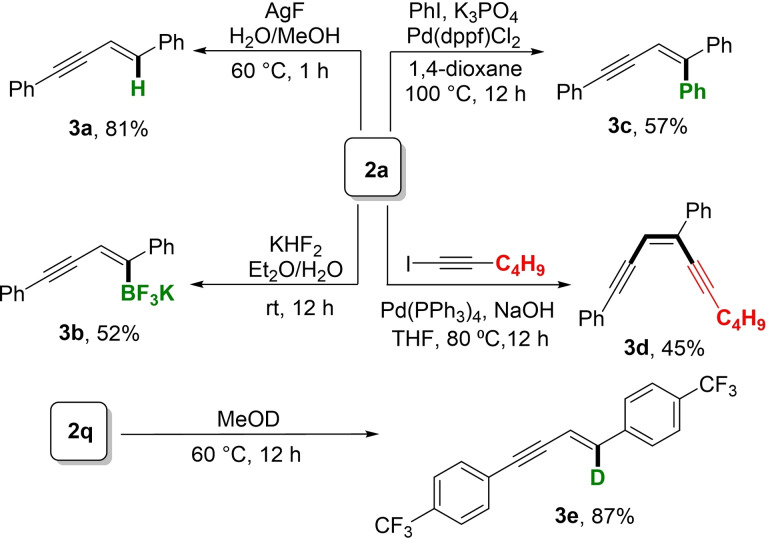
Synthetic transformation of enyne boronates.

To demonstrate utility of the reaction, we performed several boronate transformations (Scheme 4). Thus, treatment of **2 a** with AgF and H_2_O generated enyne **3 a** in 81 % yield with retention of the *trans* alkene geometry.[^21^, ^24^] Swapping of the boron substituents by reaction with KHF_2_ proceeded to afford the shelf‐stable enyne potassium trifluoroborate **3 b** in 52 % yield.^[25]^ In the presence of Pd(dppf)Cl_2_ and iodobenzene, **2 a** underwent a Suzuki‐Miyaura cross‐coupling reaction to afford **3 c** in 57 % yield.^[26]^ Alternatively, when **2 a** was treated with Pd(PPh_3_)_4_ and 1‐iodohex‐1‐yne, a Csp^2^‐Csp cross‐coupling occurred to generate *cis*‐enediyne **3 d** in 45 % yield.^[5a]^ Enediynes are priviledged scaffolds in the pharmaceutical industry with applications as potent antibiotic and anticancer agents.^[27]^ Isotopic labeling to form deuterated enynes can also be affected by exchanging the Bpin moiety with deuterium. Thus, treatment of **2 q** with *d_4_*‐methanol at elevated temperature produced deutero‐enyne **3 e** in 87 % yield.

**Scheme 5:**
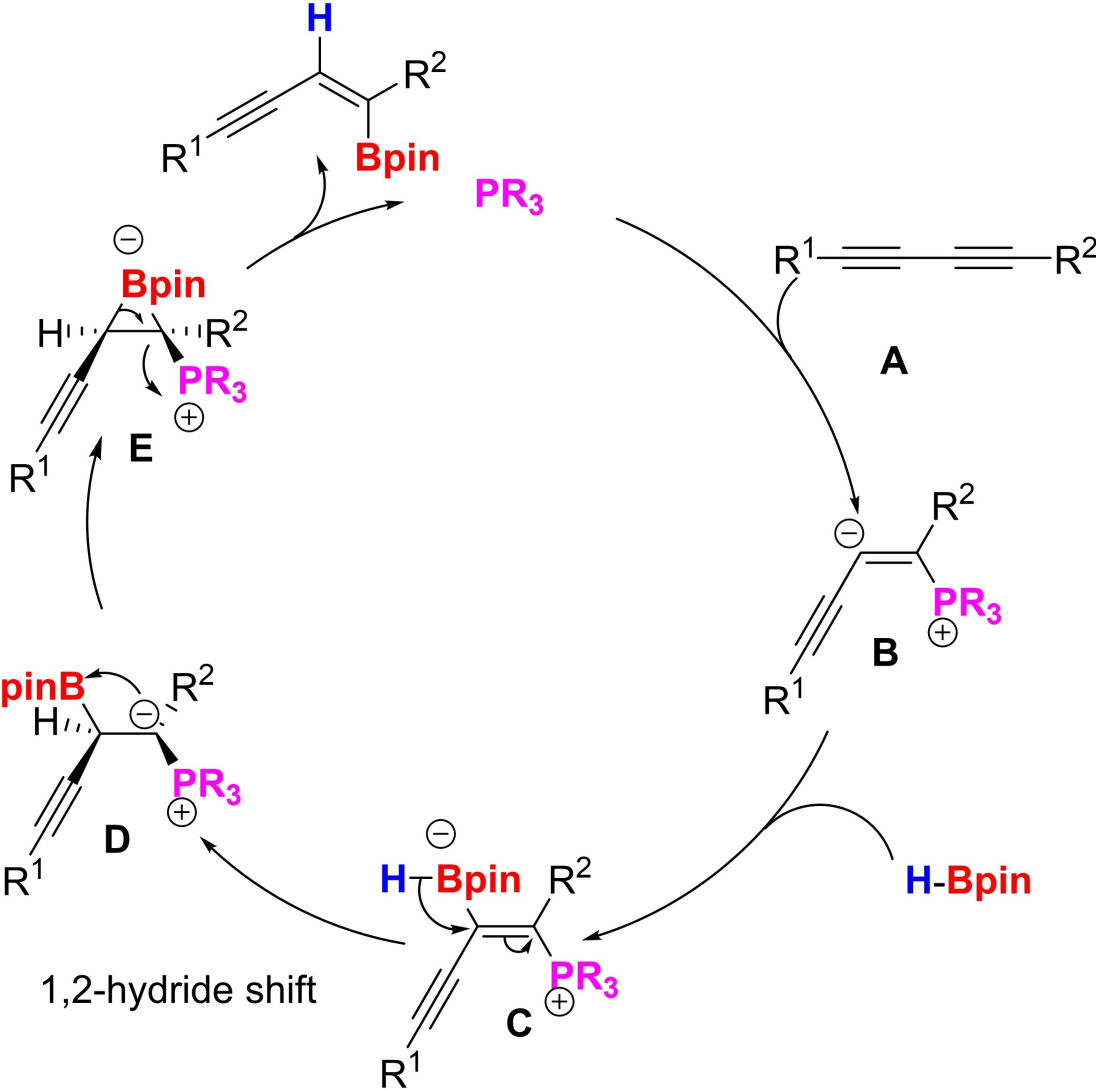
Proposed catalytic cycle.

**Scheme 6:**
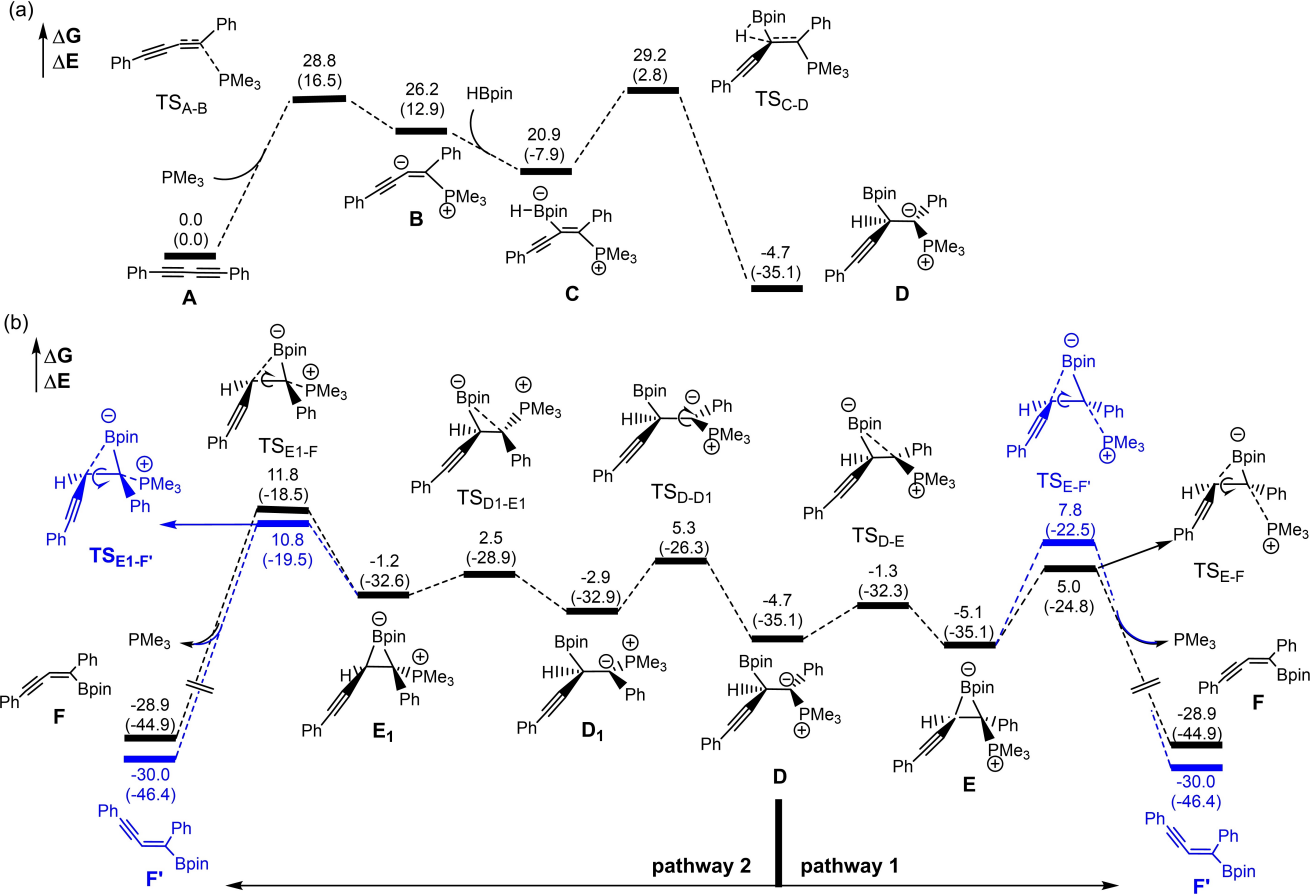
DFT calculations at the M06‐2x/6‐31(d,p) level of theory. (A) Pathway leading to key intermediate **D**. (B) Pathway that differentiates *trans* versus *cis* hydroboration products. Energy profiles were calculated for the phosphine‐catalyzed *trans* hydroboration of diyne **1 a** on the basis of the catalytic cycle shown in Scheme 5. Relative free energies and electronic energies (in parentheses) are given in kcal/mol. The black colored pathway is for the *trans* product and blue is for the *cis* product.

To gain insight into the catalytic cycle, we carried out density functional theory (DFT) calculations (see Supporting Information for details). The energy profile calculated for the reaction mechanism (Scheme 5) is illustrated in Scheme 6. Following previous reports on alkynoates,^[19a]^ we deduced that, in the first step of the reaction, the trialkyl phosphine adds to the external diyne carbon forming zwitterionic intermediate **B** (Scheme 6). **B** then reacts with HBpin to form adduct **C** that can undergo a 1,2‐hydride shift via transition state TS_C‐D_ to form intermediate **D**.^[28]^ The electron deficient boron can complex to the neighboring carbanion (TS_D‐E_) to form borirane intermediate **E** (pathway 1). The selectivity of the reaction comes into play in the next step. Intermediate **E**, through transition state TS_E‐F_, in which boron is on the external carbon, undergoes bond rotation in a counter‐clockwise direction forming the *trans* hydroborated product with the elimination of the phosphine catalyst. Alternatively, intermediate **E** can form product **F’** via *cis* hydroboration (marked in blue). TS_E‐F’_ (7.8 kcal/mol) and TS_E‐F_ (5.0 kcal/mol) have an energy difference of 2.8 kcal/mol, thus, favoring the formation of the *trans* product. In pathway 2, intermediate **D** is prone to bond rotation (TS_D‐D1_) to form intermediate **D1**. Again, boron can coordinate with the carbanion via TS_D1‐E1_ to form borirane **E1**. Clockwise bond rotation and elimination of PMe_3_ (TS_E1‐F_) can form the *trans* hydroborated product while counter‐clockwise bond rotation can form the *cis* product (TS_E1‐F’_). Pathway 1 is favored due to the energy difference of 5.8 kcal/mol between TS_E1‐F’_ (10.8 kcal/mol) and TS_E‐F_ (5.0 kcal/mol).

**Scheme 7:**
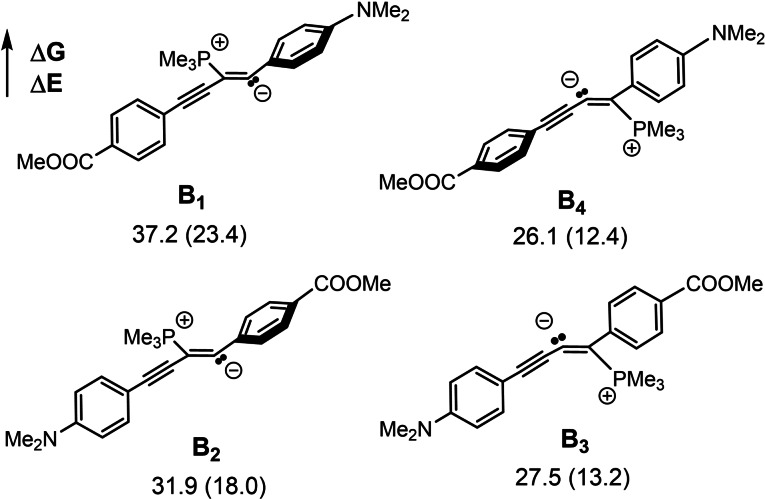
The relative stability of the four different isomers was calculated for the phosphine adduct of the unsymmetrical diyne **1 ad**. Relative free energies and electronic energies (in parentheses) are given in kcal/mol.

The highest energy points on the calculated reaction profile are TS_A‐B_ and TS_C‐D_. Experimentally, substrates with electron withdrawing groups on the aryl ring reacted faster than those with electron donating ones, and alkyl‐substituted diynes also reacted more slowly. These observations are consistent with TS_A‐B_ being stabilized by an electron withdrawing group, consistent with delocalization of negative charge along the π‐system. We calculated the energies of the four possible intermediates (**B_1_–B_4_**) in the hydroboration reaction of the donor‐acceptor substituted substrate **1 ad** resulting from phosphine attack (Scheme 7). Phosphine attack on the external carbon proximal to the donor group results in lowest energy isomer **B_4_**, which is an adduct that is most effective at delocalizing the negative charge, and is consistent with the regioselectivity observed experimentally (Scheme 3).

**Figure 2:**
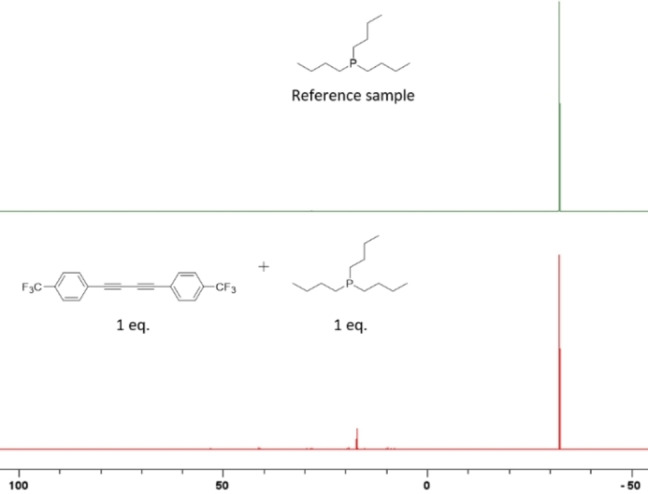
^31^P{^1^H} (121 MHz, scale in ppm) spectra of: (top) *n*‐Bu_3_P and (bottom) *n*‐Bu_3_P (0.1 mmol) and 1q (0.1 mmol) in toluene‐d_8_ after 3 days at rt.

**Figure 3:**
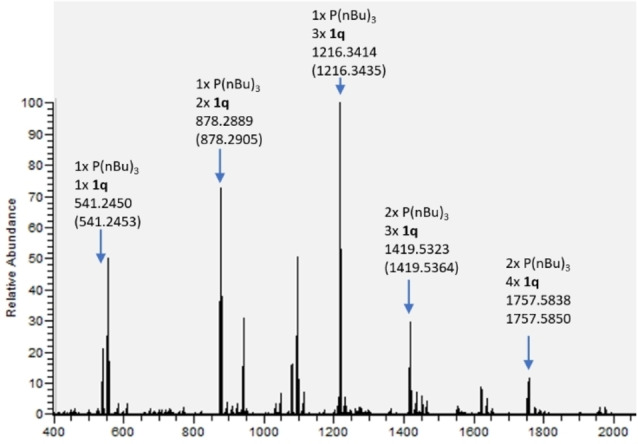
LIFDI‐HRMS (m/z) of *n*‐Bu_3_P (0.1 mmol) and **1 q** (0.1 mmol) *in* toluene‐d_8_ after 3 days at rt. The mass of several adducts of the substrates are marked with the calculated values in parentheses.

We conducted several experiments in toluene‐*d_8_* using *in situ* NMR and mass spectroscopy. First, under standard conditions without phosphine catalyst, *p*‐CF_3_‐diphenyl diyne **1 q** did not undergo hydroboration as expected (Supporting Information Figure VII_1–3). Second, no redistribution reaction or adduct formation was observed when HBpin was added to the *n*‐Bu_3_P catalyst, in contrast to previous observations with HBcat (Supporting Information Figure VII_4–6).^[22]^ Third, reaction of diyne **1 q** with *n*‐Bu_3_P for 3 days at r.t. led to a color change from yellow to black, together with a number of new signals, the major one being at 17 ppm in the ^31^P NMR spectra (Figure 2), and at −60 ppm in the ^19^F NMR spectra (Supporting Information Figure VII_7). We compared the ^31^P NMR signals with a tributyl(methyl)phosphonium salt (as an example of a formal P(V) cation) (peak at 31.4 ppm, Supporting Information Figure VII_8) to *n*‐Bu_3_P (−32.3 ppm). The significant and relatively similar downfield shifts of the signals from the reaction of **1 q** with *n*‐Bu_3_P and the vinyl phosphonium ion suggest that the new signals may belong to a zwitterionic adduct with a positive charge at the P‐atom, such as **B**. We also confirmed the formation of a phosphine‐diyne adduct, along with further adducts of varying number of phosphines and diynes 1q by *in situ* LIFDI‐HRMS (Figure 3). Furthermore, the Mulliken charges of intermediate **B**, determined by DFT calculations, support the zwitterionic nature of the adduct (Figure Supporting Information_VIII). The P‐atom has a signficant charge of +0.6 while the negative charge is delocalized onto the π‐system. Fourth, reaction of **1 q** with HBpin, using O=P(*n*Bu)_3_ instead of *n*‐Bu_3_P as the catalyst (Supporting Information Figure VII_10) also formed product **2 q**, but much more slowly. In this case, some of the phosphine oxide is reduced by HBpin *in situ* generating *n*‐Bu_3_P, which then catalyzes the reaction (Supporting Information VII_11). Taken together, the experimental evidence supports the catalytic cycle in Scheme 5.

## Conclusion

We developed a transition metal‐free method for the regio‐ and stereoselective *trans* hydroboration of 1,3‐diynes via phosphine catalysis. Interestingly, the boron moiety was installed on the external carbon of diyne framework. The reaction is compatible with a variety of substrates, including aryl, alkyl, heterocycles as well as symmetric and asymmetric 1,3‐diynes. The (*E*)‐1‐boryl‐1,3‐enynes were successfully transformed into other products including enediynes, which have potential in medicinal chemistry. Mechanistic and computational studies provided insight into the plausible mechanism for the reaction.

## Experimental Section

In a flame‐dried 2 dram vial, diyne **1 a** (50 mg, 0.25 mmol) was purged with argon, and toluene (1 mL) was added. Then, HBpin (44 μL, 0.3 mmol) and *n*‐Bu_3_P (6 μL, 0.025 mmol) were added. The vial was heated to 100 °C and stirred for 2 h. After completion of the reaction as followed by TLC, it was directly loaded onto silica and purified using a CombiFlash chromatography system (2.5 % EtOAc/hexane) to yield **2 a** as a transparent liquid (65 mg, 78 % yield). ^1^H NMR (400 MHz, CDCl_3_) δ 7.49–7.42 (m, 4H), 7.36–7.31 (m, 6H), 6.57 (s, 1H), 1.39 (s, 12H). ^13^C NMR (151 MHz, CDCl_3_) δ 140.8, 131.6, 128.6, 128.4, 128.4, 127.9, 126.9, 123.8, 119.6, 94.4, 89.4, 84.4, 25.1. ^11^B NMR (128 MHz, CDCl_3_) δ 30.7. HRMS: m/z calcd for C_22_H_24_BO_2_ [M+H]^+^ 331.1868; Found: 331.1870.

### Crystal Structures

Deposition Numbers 2150970 (for **2 g**), 2150972 (for **2 j**), 2150971 (for **2 q**), and 2150973 (for **2 ad**) contain the supplementary crystallographic data for this paper. These data are provided free of charge by the joint Cambridge Crystallographic Data Centre and Fachinformationszentrum Karlsruhe www.ccdc.cam.ac.uk/structures.

## Conflict of interest

The authors declare no conflict of interest.

1

## Supporting information

As a service to our authors and readers, this journal provides supporting information supplied by the authors. Such materials are peer reviewed and may be re‐organized for online delivery, but are not copy‐edited or typeset. Technical support issues arising from supporting information (other than missing files) should be addressed to the authors.

Supporting Information

## Data Availability

The data that support the findings of this study are available in the supplementary material of this article.
