# Supplementary material for: Transition Metal‐Free Regio‐ and Stereo‐Selective trans Hydroboration of 1,3‐Diynes: A Phosphine‐Catalyzed Access to (E)‐1‐Boryl‐1,3‐Enynes
Source: Chemistry. 2022 Sep 1;28(63):e202202349. doi: 10.1002/chem.202202349 (PMC9804376; doi:10.1002/chem.202202349)
Supplement: Supplementary file 1 — Supporting Information [file CHEM-28-0-s001.pdf]

Chemistry–A European Journal

Supporting Information

Transition Metal-Free Regio- and Stereo-Selective *trans* Hydroboration of 1,3-Diynes: A Phosphine-Catalyzed Access to (*E*)-1-Boryl-1,3-Enynes

Swetha Jos, Connor Szwetkowski, Carla Slebodnick, Robert Ricker, Ka Lok Chan, Wing Chun Chan, Udo Radius, Zhenyang Lin,* Todd B. Marder,* and Webster L. Santos*

Supporting Information

Table of Contents

I General Experimental Methods 1

II Synthesis of the substrates 2

III Synthesis and Characterization of the hydroborated products 2

IV Applications 16

V Confirmation of cis- and trans-isomers of 2a 20

VI Color changes during the reaction. 22

VII In situ NMR studies 23

VIII Computational Studies: 46

IX X-ray crystallography experiments 47

X References 50

I General Experimental Methods

Reactions were performed using the Schlenk techniques under argon or nitrogen atmosphere. All glassware used was flame-dried or oven-dried overnight. Chemicals were obtained from commercial sources unless otherwise noted. THF, toluene, MeCN, and CH_2Cl_2 were dried using the Innovative Technology Pure SolvMD solvent purification system. Column chromatography was performed using SiliaFlash P60 40-63 μm, 60 Å. TLC analyses were performed using Silicycle aluminum-backed silica gel F-254 plates and visualized by UV light or $KMnO_4$ stain. Silica gel column chromatography was performed using SiliaFlash P60 40-63 μm, 60 A silica from SiliCycle Inc. ^1H, ^{13}C, ^{11}B, ^{31}P, and ^{19}F spectra were recorded using an Agilent 400-MR 400 MHz, an Agilent U4-DD2 400 Hz, or a Bruker Avance II 500 MHz spectrometer. Chemical shifts are reported in δ ppm, ^1H, ^{13}C are referenced to an internal standard ($CDCl_3$, CD_2Cl_2, CD_3CN, or TMS) and ^{11}B, ^{31}P, ^{19}F referenced to external standards $BF_3.Et_2O$, H_3PO_4 and CCl_3F respectively. ^{13}C, ^{11}B, and ^{31}P are proton decoupled unless otherwise noted. Data are reported as follows: chemical shift, multiplicity (s = singlet, d = doublet, t = triplet, q = quartet, dd = doublet of doublets, dt = doublet of triplets, m = multiplet), coupling constants (Hz), and integration. GC−MS experiments were performed using an Agilent 7890 Series GC system coupled to an HP 5975 mass selective detector. ESI mass spectra were acquired with an Agilent 6220 LC-ESI-TOF or a Thermo Scientific Q-Exactive Orbitrap.

II Synthesis of the substrates

All diynes were prepared in accordance with previous literature.

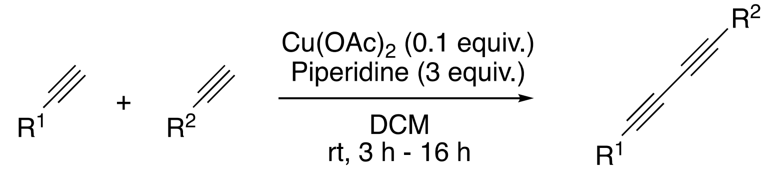

1a [1], **1b**[2], **1c**[3], **1d**[3], **1e**[2], **1f**[4], **1g**[5], **1h**[6], **1i**[7], **1j**[8], **1k**[6], **1l**[9], **1m**[7], **1n**[4], **1o**[7], **1p**[10], **1q**[11], **1r**[12], **1s**[4],**1t**[2], **1u**[3], **1v**[7], **1w**[2], **1x**[7], **1y**[13], **1z**[1], **1aa**[6], **1ab**[4], **1ac**[4], and **1ae**[14] were compared with the literature values for characterization.

methyl 4-((4-(dimethylamino)phenyl)buta-1,3-diyn-1-yl)benzoate (1ad)

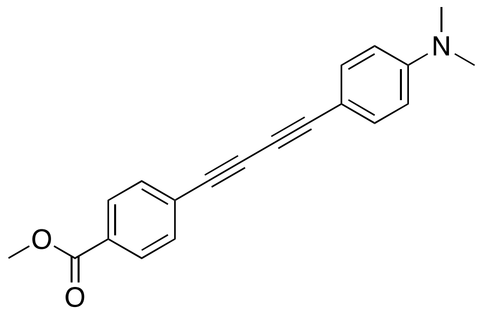

Yellow solid. **^{1}H NMR** ($CDCl_3$, 400 MHz) δ 8.01 – 7.96 (m, 2H), 7.59 – 7.54 (m, 2H), 7.43 – 7.37 (m, 2H), 6.66 – 6.61 (m, 2H), 3.90 (s, 3H), 2.99 (s, 6H). **^{13}C NMR** ($CDCl_3$, 100 MHz) δ 166.7, 151.5, 134.4, 132.7, 130.6, 129.9, 127.5, 112.1, 107.4, 85.8, 80.3, 78.0, 72.2, 52.7, 40.4. **HRMS** (ESI^+) m/z $[M+H]^+$ Calcd for $C_{20}H_{18}NO_2$ 304.1338; Found 304.1343.

III Synthesis and Characterization of the hydroborated products

To a flame-dried 7 mL vial, the diynes **1a-1z,** and **1aa-1ad** (0.25 mmol) were added and the vial was purged with argon. To this, the solvent was added (1 mL, 0.2 M). Then, the pinacol borane (0.3 mmol) and the tri-*n*-butyl phosphine catalyst (0.025 mmol) were added. This was heated to 100 °C and stirred for 1-16 h. After completion of the reaction, it was directly loaded onto silica and purified using a Combi Flash chromatograph system (0-60% EtOAc/hexanes).

Some of the compounds underwent protodeborylation during purification or in the NMR tube. CD_2Cl_2 was a better solvent compared to $CDCl_3$ to minimize protodeborylation.

Characterization of compounds

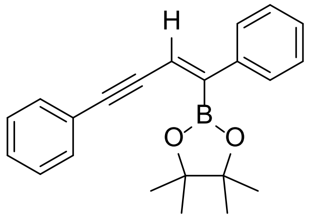

(*E*)-2-(1,4-diphenylbut-1-en-3-yn-1-yl)-4,4,5,5-tetramethyl-1,3,2-dioxaborolane (2a)

2 h reaction, transparent liquid, 64 mg, 78% yield (2.0 mmol scale, 405 mg, 62% yield). **^{1}H NMR** (400 MHz, $CDCl_3$) δ 7.49-7.42 (m, 4H), 7.36 – 7.31 (m, 6H), **6.57** (s, 1H), 1.39 (s, 12H). **^{13}C NMR** (151 MHz, $CDCl_3$) δ 140.8, 131.6, 128.6, 128.4, 128.4, 127.9, 126.9, 123.8, 119.6, 94.4, 89.4, 84.4, 25.1. **^{11}B NMR** (128 MHz, $CDCl_3$) δ 30.7. **HRMS**: (ESI^+) m/z calcd for $C_{22}H_{24}BO_2$ $[M+H]^+$ 331.1868; Found: 331.1870.

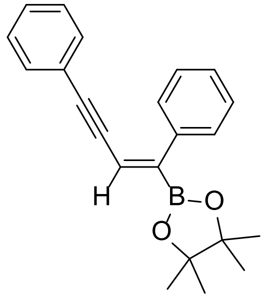

(*Z*)-2-(1,4-diphenylbut-1-en-3-yn-1-yl)-4,4,5,5-tetramethyl-1,3,2-dioxaborolane (2a- *Z* isomer)[15]

Spectroscopic data are consistent with the literature.

2 h reaction, white solid, **^{1}H NMR** (400 MHz, $CDCl_3$) δ 7.72 – 7.65 (m, 2H), 7.45 – 7.25 (m, 8H), **6.71** (s, 1H), 1.32 (s, 12H). **^{13}C NMR** (126 MHz, $CDCl_3$) δ 139.2, 131.7, 129.1, 128.4, 128.3, 127.6, 127.4, 123.3, 122.1, 96.8, 88.8, 84.0, 24.8. **^{11}B NMR** (128 MHz, $CDCl_3$) δ 30.4.

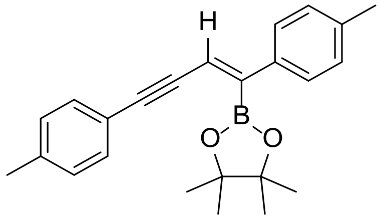

(*E*)-2-(1,4-di-p-tolylbut-1-en-3-yn-1-yl)-4,4,5,5-tetramethyl-1,3,2-dioxaborolane (2b)

16 h transparent liquid, 59 mg, 66% yield. **^{1}H NMR** (400 MHz, $CDCl_3$) δ 7.36 (d, *J* = 8.0 Hz, 2H), 7.32 (d, *J* = 8.0 Hz, 2H), 7.14 -7.12 (m, 4H), **6.53** (s, 1H), 2.35 (s, 3H), 2.34 (s, 3H), 1.38 (s, 12H). **^{13}C NMR** (101 MHz, $CDCl_3$) δ 138.3, 137.9, 137.6, 131.3, 129.2, 129.1, 126.7, 120.7, 118.7, 94.2,

88.8, 84.1, 25.0, 21.5, 21.2.[11]**B NMR** (128 MHz, $CDCl_3$) δ 30.8. **HRMS**: (ESI^+) m/z calcd for $C_{24}H_{28}BO_2$ $[M+H]^+$ 359.2181; Found: 359.2183.

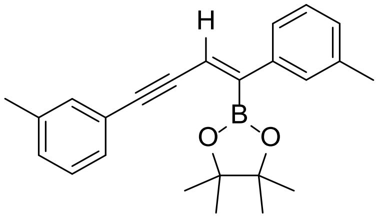

(*E*)-2-(1,4-di-m-tolylbut-1-en-3-yn-1-yl)-4,4,5,5-tetramethyl-1,3,2-dioxaborolane (2c)

16 h, transparent liquid, 64 mg, 71% yield. ^{1}**H NMR** (500 MHz, $CDCl_3$) δ 7.32 – 7.09 (m, 8H), **6.56** (s, 1H), 2.36 (s, 3H), 2.35 (s, 3H), 1.40 (s, 12H). ^{13}**C NMR** (126 MHz, $CDCl_3$) δ 140.8, 138.1, 138.0, 132.2, 129.2, 128.6, 128.6, 128.5, 128.3, 127.6, 124.1, 123.7, 119.3, 94.4, 89.1, 84.3, 25.0, 21.6, 21.4.^{11}**B NMR** (128 MHz, $CDCl_3$) δ 30.6. **HRMS:** (ESI^+) m/z calcd for $C_{24}H_{28}O_2B$ $[M+H]^+$ 359.2182; Found: 359.2169.

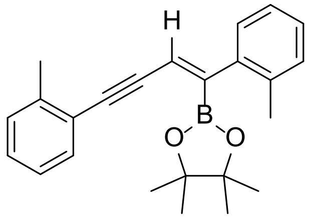

(*E*)-2-(1,4-di-o-tolylbut-1-en-3-yn-1-yl)-4,4,5,5-tetramethyl-1,3,2-dioxaborolane (2d)

16 h, transparent liquid, 53 mg, 59% yield. ^{1}**H NMR** (500 MHz, $CDCl_3$) δ 7.48 (d, *J* = 8 Hz, 1H), 7.24 – 7.21 (m, 2H), 7.19 – 7.14 (m, 5H), **6.41** (s, 1H), 2.53 (s, 3H), 2.32 (s, 3H), 1.32 (s, 12H). ^{13}**C NMR** (126 MHz, $CDCl_3$) δ 142.2, 140.3, 135.2, 131.9, 129.7, 129.3, 128.4, 128.2, 127.2, 125.8, 125.3, 124.0, 123.3, 93.8, 92.4, 83.8, 24.8, 20.8, 20.4. ^{11}**B NMR** (128 MHz, $CDCl_3$) δ 30.1. **HRMS:** (ESI^+) m/z calcd for $C_{24}H_{31}BNO_2$ $[M+NH_4]^+$ 376.2447; Found: 376.2453.

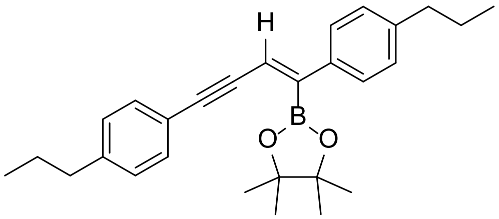

(*E*)-2-(1,4-bis(4-propylphenyl)but-1-en-3-yn-1-yl)-4,4,5,5-tetramethyl-1,3,2-dioxaborolane (2e)

16 h, pale yellow liquid, 68 mg, 66% yield. ^{1}**H NMR** (500 MHz, $CDCl_3$) δ 7.38 (d, *J* = 8 Hz, 2H), 7.34 (d, *J* = 8 Hz, 2H), 7.14 – 7.12 (m, 4H), **6.55** (s, 1H), 2.58 (t, *J* = 8 Hz, 4H), 1.69-1.39 (m, 4H), 1.39 (s, 12H), 0.96 - 0.92 (m, 6H). ^{13}**C NMR** (126 MHz, $CDCl_3$) δ 143.2, 142.5, 138.2, 131.5, 128.8, 128.6, 126.7, 121.1, 118.8, 94.4, 89.0, 84.3, 38.1, 37.9, 25.1, 24.6, 24.5, 14.1, 14.0. ^{11}**B NMR** (128 MHz, $CDCl_3$) δ 30.7. **HRMS**: (ESI^+) m/z calcd for $C_{28}H_{36}BO_2$ $[M+H]^+$ 415.2808; Found: 415.2809.

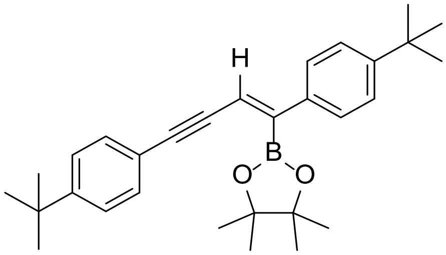

(E)-2-(1,4-bis(4-(tert-butyl)phenyl)but-1-en-3-yn-1-yl)-4,4,5,5-tetramethyl-1,3,2-dioxaborolane (2f)

16 h, transparent liquid, 50 mg, 50% yield. **^{1}H NMR** (400 MHz, $CDCl_3$) δ 7.46 – 7.29 (m, 8H), **6.57** (s, 1H), 1.39 (s, 12H), 1.32 (m, 18H). **^{13}C NMR** (126 MHz, CD_2Cl_2) δ 152.5, 151.8, 138.7, 131.9, 127.3, 126.3, 121.3, 119.4, 95.1, 89.6, 84.9, 35.5, 35.3, 31.8, 31.7, 25.6. **^{11}B NMR** (128 MHz, $CDCl_3$) δ 28.6. **HRMS**: (ESI^+) m/z calcd for $C_{60}H_{78}B_2NaO_4$ $[2M+Na]^+$ 907.8675; Found: 907.8715.

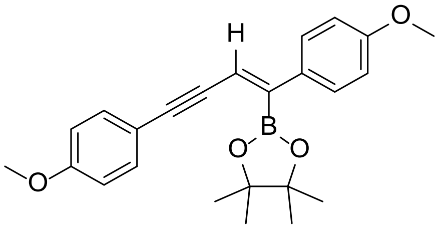

(E)-2-(1,4-bis(4-methoxyphenyl)but-1-en-3-yn-1-yl)-4,4,5,5-tetramethyl-1,3,2-dioxaborolane (2g)

16 h, white solid, 63 mg, 65% yield. **^{1}H NMR** (400 MHz, $CDCl_3$) δ 7.40 (d, J = 9 Hz, 2H), 7.37 (d, J = 9 Hz, 2H), 6.86 (d, J = 8.0 Hz, 2H), 6.85 (d, J = 8.0 Hz, 2H), **6.49** (s, 1H), 3.82 (s, 3H), 3.81 (s, 3H), 1.38 (s, 12H). **^{13}C NMR** (101 MHz, $CDCl_3$) δ 160.0, 159.9, 133.9, 133.3, 128.4, 118.2, 116.5, 114.4, 94.3, 88.8, 84.5, 55.8, 25.4. **^{11}B NMR** (128 MHz, $CDCl_3$) δ 30.8. **HRMS**: (ESI^+) m/z calcd for $C_{24}H_{28}BO_4$ $[M+H]^+$ 391.2081; Found: 391.2059.

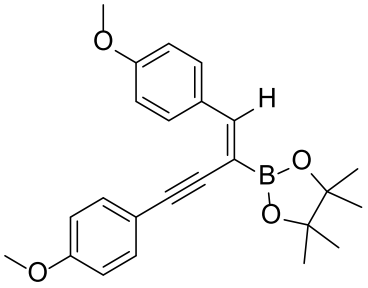

(E)-2-(1,4-bis(4-methoxyphenyl)but-1-en-3-yn-2-yl)-4,4,5,5-tetramethyl-1,3,2-dioxaborolane (2g') [16]

2 h, transparent liquid, 61 mg, 62% yield. **^{1}H NMR** (400 MHz, $CDCl_3$) δ 8.06 (d, J = 9 Hz, 2H), 7.47 (d, J = 9 Hz, 2H), **7.34** (s, 1H), 6.91 (d, J = 9 Hz, 2H), 6.87 (d, J = 9 Hz, 2H), 3.84 (s, 3H),

3.83 (s, 3H), 1.34 (s, 12H). **^{13}C NMR** (101 MHz, $CDCl_3$) δ 160.3, 159.3, 148.4, 133.0, 131.4, 130.4, 116.7, 113.9, 113.6, 96.8, 89.2, 84.1, 55.3, 24.8. **^{11}B NMR** (128 MHz, $CDCl_3$) δ 29.8.

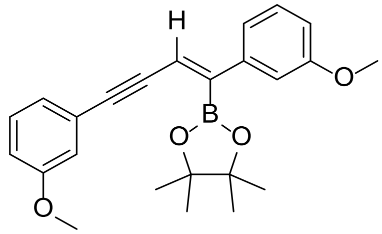

(*E*)-2-(1,4-bis(3-methoxyphenyl)but-1-en-3-yn-1-yl)-4,4,5,5-tetramethyl-1,3,2-dioxaborolane (2h)

16 h, sticky liquid, 48 mg, 65% yield. **^{1}H NMR** (500 MHz, $CDCl_3$) δ 7.26 – 7.21 (m, 2H), 7.07 (dt, *J* = 8 Hz, 1H), 7.04 – 6.96 (m, 3H), 6.89 – 6.82 (m, 2H), **6.57** (s, 1H), 3.81 (s, 3H), 3.81 (s, 3H), 1.39 (s, 12H).**^{13}C NMR** (126 MHz, $CDCl_3$) δ 159.6, 159.3, 142.0, 129.5, 129.4, 124.6, 124.1, 119.5, 119.3, 116.2, 114.9, 113.5, 112.2, 94.3, 88.9, 84.2, 55.2, 55.2 24.9. **^{11}B NMR** (128 MHz, $CDCl_3$) δ 30.5. **HRMS**: (ESI^+) m/z calcd for $C_{48}H_{54}B_2O_8$ $[2M]^+$ 780.4015; Found: 780.4024.

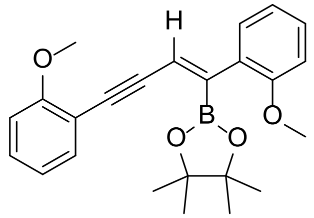

(*E*)-2-(1,4-bis(2-methoxyphenyl)but-1-en-3-yn-1-yl)-4,4,5,5-tetramethyl-1,3,2-dioxaborolane (2i)

16 h, transparent liquid, 68 mg, 70% yield. **^{1}H NMR** (500 MHz, $CDCl_3$) δ 7.49 (dd, *J* = 8, 1.7 Hz, 1H), 7.35 – 7.24 (m, 3H), 6.99 – 6.84 (m, 4H), **6.54** (s, 1H), 3.91 (s, 3H), 3.83 (s, 3H), 1.37 (s, 12H). **^{13}C NMR** (126 MHz, $CDCl_3$) δ 159.7, 156.2, 133.4, 131.0, 129.4, 129.1, 127.8, 121.2, 120.2, 119.5, 113.0, 110.4, 110.4, 93.1, 89.7, 83.6, 55.6, 55.3, 24.8. **^{11}B NMR** (128 MHz, $CDCl_3$) δ 30.4. **HRMS**: (ESI^+) m/z calcd for $C_{48}H_{54}B_2O_8$ $[2M]^+$ 780.4015; Found: 780.3941.

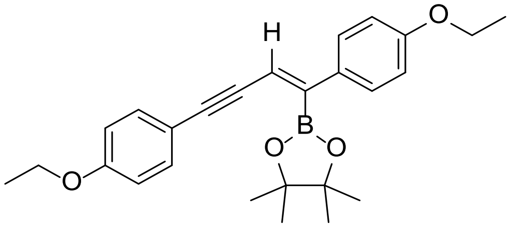

(*E*)-2-(1,4-bis(4-ethoxyphenyl)but-1-en-3-yn-1-yl)-4,4,5,5-tetramethyl-1,3,2-dioxaborolane (2j)

16 h, white solid, 56 mg, 78%,**^{1}H NMR** (500 MHz, $CDCl_3$) δ 7.38 (d, *J* = 9 Hz, 2H), 7.35 (d, *J* = 9 Hz, 2H), 6.84 (d, *J* = 8.0 Hz, 2H), 6.83 (d, *J* = 8.0 Hz, 2H), **6.48** (s, 1H), 4.06 - 4.01 (m, 4H), 1.43 – 1.39 (m, 6H), 1.38 (s, 12H). **^{13}C NMR** (126 MHz, $CDCl_3$) δ 158.8, 158.7, 133.9, 133.1, 132.7, 127.8, 117.4, 114.4, 114.3, 93.8, 88.2, 84.0, 63.4, 63.3, 24.9, 14.7, 14.7. **^{11}B NMR** (128 MHz, $CDCl_3$) δ 30.3. **HRMS**: (ESI^+) m/z calcd for $C_{26}H_{32}O_4B$ $[M+H]^+$ 419.2394; Found: 419.2407.

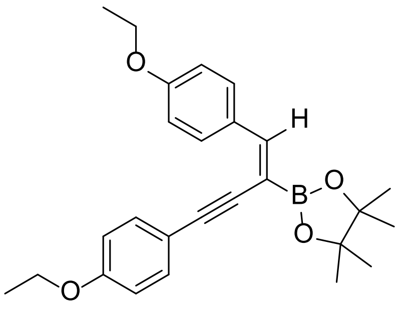

(*E*)-2-(1,4-bis(4-ethoxyphenyl)but-1-en-3-yn-2-yl)-4,4,5,5-tetramethyl-1,3,2-dioxaborolane (2j')

16 h, transparent liquid, 48 mg, 67% yield. **^{1}H NMR** (500 MHz, $CDCl_3$) δ 8.06 (d, *J* = 9 Hz, 2H), 7.46 (d, *J* = 9 Hz, 2H), **7.33** (s, 1H), 6.89 (d, *J* = 9 Hz, 2H), 6.86 (d, *J* = 9 Hz, 2H), 4.11 – 3.96 (m, 4H), 1.44 – 1.41 (m, 6H), 1.34 (s, 12H). **^{13}C NMR** (126 MHz, $CDCl_3$) δ 159.6, 158.7, 148.5, 133.0, 131.4, 130.2, 116.4, 114.3, 114.0, 96.8, 89.2, 84.0, 63.5, 63.4, 24.8, 14.8, 14.8. **^{11}B NMR** (128 MHz, $CDCl_3$) δ 30.07. **HRMS**: (ESI^+) m/z calcd for $C_{26}H_{31}O_4NaB$ $[M+Na]^+$ 441.2213; Found: 441.2206;

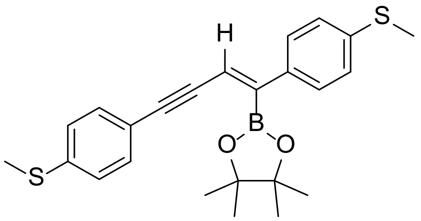

(*E*)-2-(1,4-bis(4-(methylthio)phenyl)but-1-en-3-yn-1-yl)-4,4,5,5-tetramethyl-1,3,2-dioxaborolane (2k)

16 h, white solid, 67 mg, 63% yield. **^{1}H NMR** (400 MHz, CD_2Cl_2) δ 7.37 (d, *J* = 8 Hz, 2H), 7.35 (d, *J* = 8 Hz, 2H), 7.21 (d, *J* = 8 Hz, 2H), 7.20 (d, *J* = 8 Hz, 2H)) **6.53** (s, 1H), 2.50 (s, 3H), 2.49 (s, 3H), 1.37 (s, 12H). **^{13}C NMR** (126 MHz, CD_2Cl_2) δ 140.1, 138.8, 137.6, 131.97, 127.5, 126.4, 125.9, 119.9, 118.8, 94.6, 89.8, 84.5, 25.1, 15.7, 15.3. **^{11}B NMR** (128 MHz, CD_2Cl_2) δ 31.0. **HRMS**: (ESI^+) m/z calcd for $C_{24}H_{28}BO_2S_2$ $[M+H]^+$: 423.1623; Found: 423.1648.

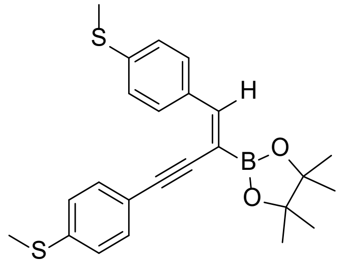

(*E*)-2-(1,4-bis(4-(methylthio)phenyl)but-1-en-3-yn-2-yl)-4,4,5,5-tetramethyl-1,3,2-dioxaborolane (2k')

2 h, sticky liquid, 45 mg, 43% yield. **Crude ^{1}H NMR** (400 MHz, CD_2Cl_2) δ 7.99 (d, *J* = 8 Hz, 2H), 7.41 (d, *J* = 8 Hz, 2H), **7.35** (s, 1H), 7.28 – 7.12 (m, 4H), 2.50 (s, 3H), 2.49 (s, 3H), 1.33 (s, 12H).

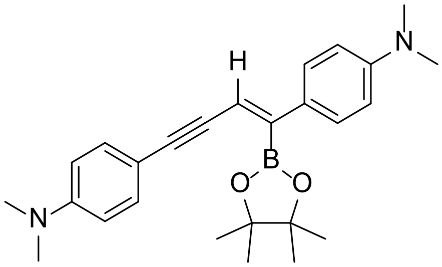

(*E*)-4,4'-(1-(4,4,5,5-tetramethyl-1,3,2-dioxaborolan-2-yl)but-1-en-3-yne-1,4-diyl)bis(N,N-dimethylaniline) (2l)

16 h, yellowish liquid, 52 mg, 50%. **Crude NMR**. Protodeborylation observed. **^{1}H NMR** (400 MHz, $CDCl_3$) δ 8.06 (d, *J* = 9 Hz, 2H), 7.42 (d, *J* = 9 Hz, 2H), **6.69 – 6.66** (m, 5H), 3.01 (s, 6H), 2.98 (s, 6H), 1.33 (s, 12H). **^{11}B NMR** (128 MHz, $CDCl_3$) δ 30.5. **HRMS**: (ESI^+) m/z calcd for $C_{26}H_{34}BN_2O_2$ $[M+H]^+$ 419.2772; Found: 419.2773.

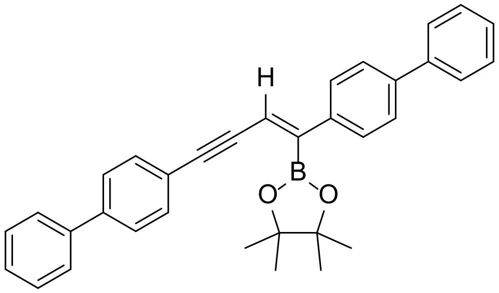

(*E*)-2-(1,4-di([1,1'-biphenyl]-4-yl)but-1-en-3-yn-1-yl)-4,4,5,5-tetramethyl-1,3,2-dioxaborolane (2m)

16 h, white solid, 71 mg, 59% yield. **^{1}H NMR** (400 MHz, CD_2Cl_2) δ 7.64 – 7.59 (m, 8H), 7.56 (d, *J* = 8 Hz, 2H), 7.52 (d, *J* = 8 Hz, 2H), 7.48 – 7.43 (m, 2H), 7.39 - 7.34 (m, 4H), **6.65** (s, 1H), 1.41 (s, 12H). **^{13}C NMR** (126 MHz, CD_2Cl_2) δ 141.2, 140.7, 140.6, 140.3, 140.0, 132.1, 129.1, 129.0, 128.0, 127.6, 127.5, 127.3, 127.2, 127.1, 127.1, 122.6, 119.4, 94.7, 90.3, 84.4, 25.0. **^{11}B NMR** (128

MHz, CD_2Cl_2) δ 31.1. **HRMS**: (ESI^+) m/z calcd for $C_{34}H_{32}BO_2$ $[M+H]^+$ 486.2589; Found: 486.2596.

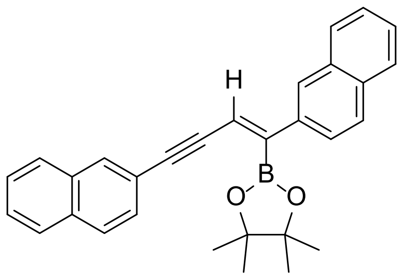

(*E*)-2-(1,4-di(naphthalen-2-yl)but-1-en-3-yn-1-yl)-4,4,5,5-tetramethyl-1,3,2-dioxaborolane (2n)

3 h, transparent liquid, 79 mg, 73% yield. **^{1}H NMR** (400 MHz, CD_2Cl_2) δ 8.61 (d, *J* = 8 Hz, 1H), 7.99 -7.96 (m, 1H), 7.92 – 7.88 (m, 3H), 7.84 – 7.76 (m, 2H), 7.67 – 7.61 (m, 1H), 7.59 – 7.56 (m, 1H), 7.51 – 7.48 (m, 4H), 7.38 (d, *J* = 7 Hz, 1H), **6.69** (s, 1H), 1.32 (s, 12H). **^{13}C NMR** (126 MHz, CD_2Cl_2) δ 141.6, 134.1, 134.0, 133.9, 132.1, 131.3, 129.6, 128.9, 128.8, 128.3, 127.4, 127.3, 127.1, 126.5, 126.5, 126.4, 126.3, 126.0, 125.92, 125.2, 121.8, 94.3, 93.7, 84.8, 25.2. **^{11}B NMR** (128 MHz, CD_2Cl_2) δ 30.3. **HRMS**: (ESI^+) m/z calcd for $C_{30}H_{28}BO_2$ $[M+H]^+$: 431.2182; Found: 431.2183.

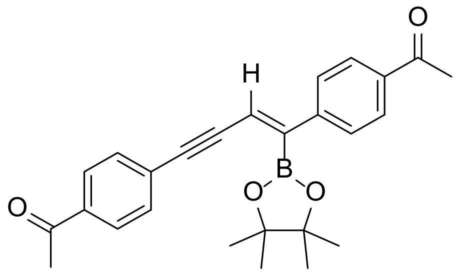

(*E*)-1,1'-((1-(4,4,5,5-tetramethyl-1,3,2-dioxaborolan-2-yl)but-1-en-3-yne-1,4-diyl)bis(4,1-phenylene))bis(ethan-1-one) (2o)

1 h, white solid, 50 mg, 48 % yield. **^{1}H NMR** (500 MHz, $CDCl_3$) δ 7.94 (d, *J* = 8 Hz, 4H), 7.56 (d, *J* = 8 Hz, 2H), 7.52 (d, *J* = 8 Hz, 2H), **6.66** (s, 1H), 2.62 (s, 3H), 2.60 (s, 3H), 1.39 (s, 12H). **^{13}C NMR** (126 MHz, $CDCl_3$) δ 197.8, 197.5, 145.4, 136.4, 136.3, 131.7, 128.8, 128.5, 128.3, 127.2, 121.6, 94.9, 92.2, 84.6, 26.8, 25.1.**^{11}B NMR** (128 MHz, $CDCl_3$) δ 30.8. **HRMS**: (ESI^+) m/z calcd for $C_{26}H_{28}BO_4$ $[M+H]^+$ 415.2081; Found: 415.2059.

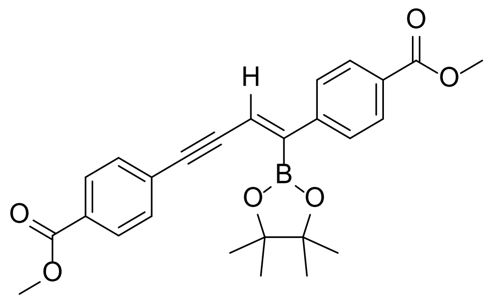

dimethyl 4,4'-(1-(4,4,5,5-tetramethyl-1,3,2-dioxaborolan-2-yl)but-1-en-3-yne-1,4-diyl)(*E*)-dibenzoate (2p)

1 h, sticky liquid, 59 mg, 53% yield (with 10% dimethyl 4,4'-(1-(4,4,5,5-tetramethyl-1,3,2-dioxaborolan-2-yl)but-1-en-3-yne-1,4-diyl)(*Z*)-dibenzoate) . ^{1}**H NMR** (400 MHz, CD_2Cl_2) δ 8.02 – 7.94 (m, 4H), 7.54 (d, J = 8 Hz, 2H), 7.50 (d, J = 8 Hz, 2H), **6.65** (s, 1H), 3.89 (s, 3H), 3.88 (s, 3H), 1.37 (s, 12H). ^{13}**C NMR** (101 MHz, CD_2Cl_2) δ 167.0, 166.7, 132.0, 131.8, 130.0, 129.9, 129.8, 129.2, 128.3, 127.3, 121.6, 95.0, 92.2, 84.8, 52.5, 52.4, 25.1. ^{11}**B NMR** (128 MHz, CD_2Cl_2) δ 30.3. **HRMS**: (ESI^+) m/z calcd for $C_{26}H_{28}BO_6$ $[M+H]^+$ 447.1979; Found: 447.1983.

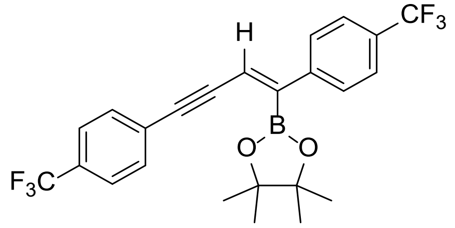

(*E*)-2-(1,4-bis(4-(trifluoromethyl)phenyl)but-1-en-3-yn-1-yl)-4,4,5,5-tetramethyl-1,3,2-dioxaborolane (2q)

1 h, yellow solid, 98 mg, 84% yield. ^{1}**H NMR** (400 MHz, $CDCl_3$) δ 7.64 – 7.52 (m, 8H), **6.61** (s, 1H), 1.38 (s, 12H). ^{13}**C NMR** (126 MHz, $CDCl_3$) δ 144.3, 132.1, 131.8, 130.6 – 130.0 (q, J = 33 Hz), 130.1 – 129.4 (q, J = 34 Hz), 127.5 – 121.1 (q, J = 271 Hz), 127.3, 126.7 – 120.8 (q, J = 263 Hz), 125.7 -125.6 (q, J = 4 Hz), 125.5 – 124.4 (q, J = 4 Hz), 121.8, 94.1, 91.1, 84.6, 25.1.^{19}**F NMR** (376 MHz, $CDCl_3$) δ -62.59, -62.86. ^{11}**B NMR** (128 MHz, $CDCl_3$) δ 30.4. **HRMS**: (ESI^+) m/z calcd for $C_{24}H_{25}BF_6NO_2$ $[M+NH_4]^+$ 484.1881; Found: 484.1885.

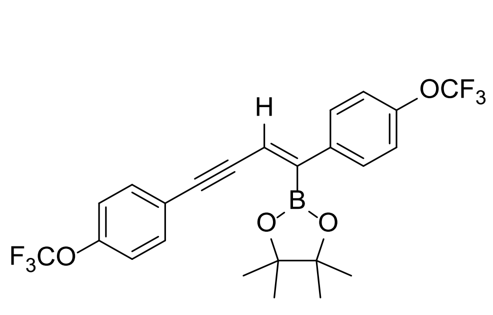

(*E*)-2-(1,4-bis(4-(trifluoromethoxy)phenyl)but-1-en-3-yn-1-yl)-4,4,5,5-tetramethyl-1,3,2-dioxaborolane (2r)

1 h, white solid, 118 mg, 95% yield. **^{1}H NMR** (400 MHz, CD_2Cl_2) δ 7.52 (d, *J* = 9 Hz, 2H), 7.47 (d, *J* = 9 Hz, 2H), 7.23 – 7.19 (m, 4H), **6.55** (s, 1H), 1.37 (s, 12H). **^{13}C NMR** (126 MHz, CD_2Cl_2) δ 149.4, 149.2, 140.1, 133.4, 128.8, 124.0 – 117.9 (q, *J* = 257.0 Hz), 123.9 – 117.8 (q, *J* = 258 Hz), 123.9, 121.4, 121.3, 121.0, 93.9, 90.2, 84.7, 25.1. **^{11}B NMR** (128 MHz, $CDCl_3$) δ 28.10.**^{19}F NMR** (376 MHz, $CDCl_3$) δ -57.79, -57.80. **HRMS**: (ESI^+) m/z calcd for $C_{24}H_{22}BF_6O_4$ $[M+H]^+$:499.1515; Found: 499.1541.

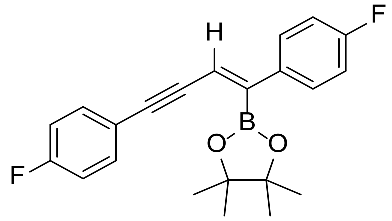

(*E*)-2-(1,4-bis(4-fluorophenyl)but-1-en-3-yn-1-yl)-4,4,5,5-tetramethyl-1,3,2-dioxaborolane (2s)

2 h, white solid, 62 mg, 68% yield. **^{1}H NMR** (400 MHz, $CDCl_3$) δ 7.46 - 7.38 (m, 4H), 7.05 -7.00 (m, 4H), 6.49 (s, 1H), 1.38 (s, 12H). **^{13}C NMR** (126 MHz, $CDCl_3$) δ 163.60 (d, *J* = 244 Hz), 163.50 (d, *J* = 251 Hz), 136.75 (d, *J* = 3 Hz), 133.30 (d, *J* = 8 Hz), 128.44 (d, *J* = 8 Hz), 119.70 (d, *J* = 4 Hz), 119.54, 115.76 (d, *J* = 32 Hz), 115.58 (d, *J* = 32 Hz), 93.37, 88.76, 84.26, 24.93. **^{11}B NMR** (128 MHz, $CDCl_3$) δ 30.6. **^{19}F NMR** (376 MHz, $CDCl_3$) δ -110.77 – -110.93 (m), -114.46 – -114.66 (m). **HRMS**: (ESI^+) m/z calcd for $C_{22}H_{22}O_2F_2B$ $[M+H]^+$ 367.1681; Found: 367.1705.

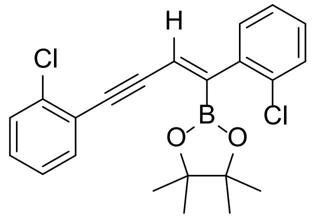

(*E*)-2-(1,4-bis(2-chlorophenyl)but-1-en-3-yn-1-yl)-4,4,5,5-tetramethyl-1,3,2-dioxaborolane (2t)

1 h, transparent liquid, 65 mg, 65% yield. **^{1}H NMR** (500 MHz, $CDCl_3$) δ 7.57 – 7.54 (m, 1H), 7.42 – 7.39 (m, 1H), 7.36 – 7.34 (m, 1H), 7.31 – 7.29 (m, 1H), 7.25 – 7.20 (m, 4H), **6.47** (s, 1H) 1.34 (s, 12H). **^{13}C NMR** (126 MHz, $CDCl_3$) δ 141.1, 135.7, 133.4, 132.7, 129.5, 129.3, 129.2, 129.1, 128.8, 127.1, 126.3, 123.4, 93.2, 91.7, 84.1, 24.8. **^{11}B NMR** (128 MHz, $CDCl_3$) δ 29.9. **HRMS**: (ESI^+) m/z calcd for $C_{22}H_{22}BCl_2O_2$ $[M+H]^+$ 400.1109; Found: 400.1108.

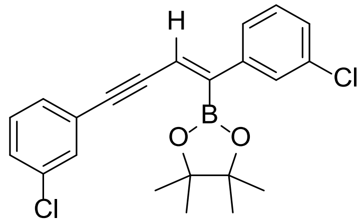

(*E*)-2-(1,4-bis(3-chlorophenyl)but-1-en-3-yn-1-yl)-4,4,5,5-tetramethyl-1,3,2-dioxaborolane (2u)

1 h, transparent liquid, 61 mg, 61% yield. **^{1}H NMR** (500 MHz, $CDCl_3$) δ 7.47 – 7.46 (m, 1H), 7.42 – 7.41 (m, 1H), 7.36 – 7.34 (m, 2H), 7.30 – 7.27 (m, 3H), 7.26 – 7.24 (m, 1H), **6.54** (s, 1H), 1.38 (s, 12H). **^{13}C NMR** (126 MHz, $CDCl_3$) δ 142.9, 134.8, 134.6, 131.8, 130.1, 130.0, 129.9, 129.0, 128.2, 127.3, 125.6, 125.5, 121.2, 94.0, 90.4, 84.8, 25.3. **^{11}B NMR** (128 MHz, $CDCl_3$) δ 30.0. **HRMS**: (ESI^+) m/z calcd for $C_{22}H_{22}BCl_2O_2$ $[M+H]^+$ 399.1088; Found: 399.1093.

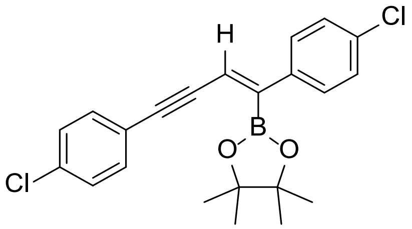

(*E*)-2-(1,4-bis(4-chlorophenyl)but-1-en-3-yn-1-yl)-4,4,5,5-tetramethyl-1,3,2-dioxaborolane (2v)

Solubilty issues with the starting material (**1v**).
1 h, white solid, 57 mg, 57% yield. **^{1}H NMR** (400 MHz, CD_2Cl_2) δ 7.45 – 7.22 (m, 8H), **6.53** (s, 1H), 1.36 (s, 12H). **^{13}C NMR** (126 MHz, CD_2Cl_2) δ 140.1, 135.2, 134.4, 133.5, 129.6, 129.4, 129.1, 122.8, 120.8, 94.5, 90.8, 85.1, 25.6. **^{11}B NMR** (128 MHz CD_2Cl_2) δ 30.5. **HRMS**: (ESI^+) m/z calcd for $C_{22}H_{22}BCl_2O_2$ $[M+H]^+$ 403.1044; Found: 403.1097.

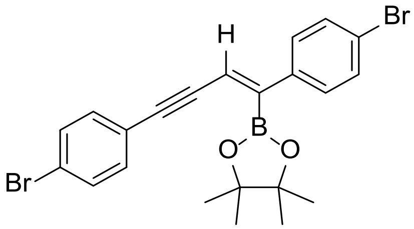

(*E*)-2-(1,4-bis(4-bromophenyl)but-1-en-3-yn-1-yl)-4,4,5,5-tetramethyl-1,3,2-dioxaborolane (2w)

1 h, white solid, 42 mg, 62% yield (with 12% *Z* isomer). **^{1}H NMR** (400 MHz, CD_2Cl_2) δ 7.51 – 7.46 (m, 4H), 7.36 – 7.30 (m, 4H), **6.53** (s, 1H), 1.35 (s, 12H). **^{13}C NMR** (126 MHz, CD_2Cl_2 δ 140.2, 133.3, 132.1, 131.9, 128.9, 123.0, 122.8, 122.2, 120.4, 94.2, 90.6, 84.7, 25.1. **^{11}B NMR** (128 MHz, CD_2Cl_2) δ 30.2. **HRMS**: (ESI^+) m/z calcd for $C_{22}H_{22}BBr_2O_2$ $[M+H]^+$ 487.0080; Found: 487.0002.

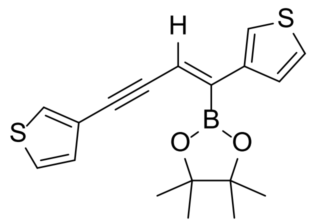

(*E*)-2-(1,4-di(thiophen-3-yl)but-1-en-3-yn-1-yl)-4,4,5,5-tetramethyl-1,3,2-dioxaborolane (2x)

1 h, white solid, 54 mg, 63% yield. **^{1}H NMR** (400 MHz, CD_2Cl_2) δ 7.33 (d, *J* = 5 Hz, 1H), 7.25 (d, *J* = 4 Hz, 1H), 7.22 (d, *J* = 4 Hz, 2H), 7.06 – 6.97 (m, 2H), **6.61** (s, 1H), 1.40 (s, 12H). **^{13}C NMR** (126 MHz, CD_2Cl_2) δ 145.1, 132.2, 128.3, 128.1, 127.8, 127.7, 125.6, 124.0, 116.8, 93.8, 88.8, 84.8, 25.1. **^{11}B NMR** (128 MHz, CD_2Cl_2) δ 30.0. **HRMS**: (ESI^{+}) m/z calcd for $C_{18}H_{20}BO_2S_2$ $[M+H]^{+}$ 343.0998; Found: 343.1012.

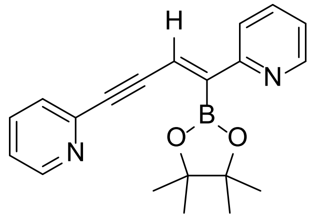

(*E*)-2,2'-(1-(4,4,5,5-tetramethyl-1,3,2-dioxaborolan-2-yl)but-1-en-3-yne-1,4-diyl)dipyridine (2y)

1 h, yellow solid, 38 mg, 47 % yield. **^{1}H NMR** (400 MHz, $CDCl_3$) δ 8.73 - 8.68 (m, 2H), 8.56 - 8.51 (m, 2H), 7.77 – 7.74 (m, 2H), 7.30 – 7.28 (m, 2H), **6.61** (s, 1H), 1.38 (s, 12H). **^{13}C NMR** (126 MHz, $CDCl_3$) δ 152.2, 148.9, 148.8, 148.2, 138.3, 136.4, 134.0, 123.3, 123.1, 121.6, 120.6, 92.0, 84.5, 84.2, 25.0. **^{11}B NMR** (128 MHz, $CDCl_3$) δ 30.2. **HRMS**: (ESI^{+}) m/z calcd for $C_{20}H_{22}BN_2O_2$ $[M+H]^{+}$ 333.1774; Found: 333.1776.

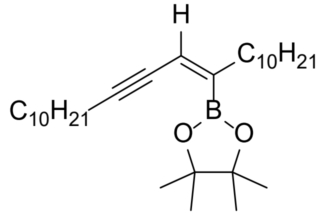

(*E*)-4,4,5,5-tetramethyl-2-(tetracos-11-en-13-yn-11-yl)-1,3,2-dioxaborolane (2z)

24 h, transparent liquid, 49 mg, 43% yield (Low conversion of starting material), (difficulty in separating isomers and multiple columns were run to separate the products). **^{1}H NMR** (400 MHz, $CDCl_3$) δ **6.07** (s, 1H), 2.39- 2.35 (m, 2H), 2.22 – 2.13 (m, 2H), 1.44 – 1.37 (m, 6H), 1.30 – 1.21 (m, 38H), 0.88 (t, *J* = 7 Hz, 6H). **^{13}C NMR** (126 MHz, $CDCl_3$) δ 174.6, 121.6, 97.3, 83.8, 82.5, 80.5, 36.4, 32.3, 32.3, 30.1, 30.0, 30.0, 29.9, 29.9, 29.8, 29.5, 29.3, 29.0, 28.5, 23.1, 19.9, 14.5. **^{11}B NMR** (128 MHz, $CDCl_3$) δ 28.0. **HRMS**: (ESI^{+}) m/z calcd for $C_{30}H_{55}BNaO_2$ $[M+Na]^{+}$ 482.4224; Found: 482.4249.

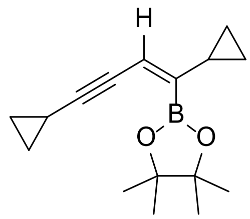

(*E*)-2-(1,4-dicyclopropylbut-1-en-3-yn-1-yl)-4,4,5,5-tetramethyl-1,3,2-dioxaborolane (2aa)

24 h, transparent liquid, 20 mg, 30% yield (difficulty in separating the isomers). **^{1}H NMR** (400 MHz, $CDCl_3$) δ **5.83** (s, 1H), 1.29 (s, 12H), 0.79 – 0.75 (m, 2H), 0.71 – 0.69 (m, 5H), 0.58 – 0.55 (m, 3H). **^{13}C NMR** (126 MHz, $CDCl_3$) δ 116.9, 95.8, 83.9, 83.5, 75.5, 31.4, 25.2, 17.4, 8.7, 7.7, 0.4. **^{11}B NMR** (128 MHz, $CDCl_3$) δ 29.8. **HRMS**: (ESI$^+$) m/z calcd for $C_{16}H_{24}BO_2$ $[M+H]^+$ 259.1867; Found: 259.1855.

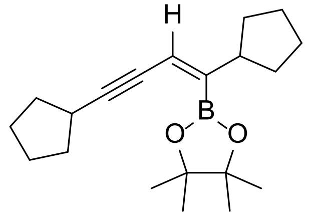

(*E*)-2-(1,4-dicyclopentylbut-1-en-3-yn-1-yl)-4,4,5,5-tetramethyl-1,3,2-dioxaborolane (2ab)

24 h, transparent liquid, 18 mg, 22% yield (difficulty in separating isomers).**^{1}H NMR** (500 MHz, $CDCl_3$) δ **6.15** (s, 1H), 3.20 – 3.01 (m, 1H), 2.84 – 2.71 (m, 1H), 1.45 – 1.38 (m, 10H), 1.23 (s, 12H), 0.94 – 0.92 (m, 6H). **^{13}C NMR** (101 MHz, $CDCl_3$) δ 179.2, 121.5, 83.1, 77.2, 76.7, 43.3, 34.0, 32.2, 26.2, 25.1, 24.9, 24.9, 24.7, 23.0, 22.7, 13.6. **^{11}B NMR** (128 MHz, $CDCl_3$) δ 30.15. **HRMS**: (ESI$^+$) m/z calcd for $C_{20}H_{32}BO_2$ $[M+H]^+$ 315.2532; Found: 315.2495.

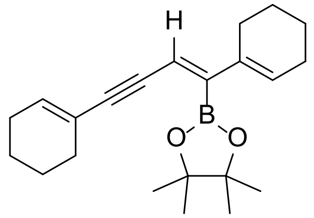

(*E*)-2-(1,4-di(cyclohex-1-en-1-yl)but-1-en-3-yn-1-yl)-4,4,5,5-tetramethyl-1,3,2-dioxaborolane (2ac)

16 h, transparent liquid, 38 mg, 45% yield. **^{1}H NMR** (500 MHz, $CDCl_3$) δ 6.07 – 6.05 (m, 1H), **6.02** (s, 1H), 5.86 – 5.84 (m, 1H), 2.20 – 2.05 (m, 9H), 1.70 – 1.59 (m, 7H), 1.36 (s, 12H). **^{13}C NMR** (126 MHz, $CDCl_3$) δ 137.8, 134.1, 130.8, 121.2, 112.0, 95.1, 87.0, 83.9, 29.1, 26.3, 25.7, 24.9, 24.8, 22.5, 22.3, 22.0, 21.4. **^{11}B NMR** (128 MHz, $CDCl_3$) δ 31.7. **HRMS**: (ESI$^+$) m/z calcd for $C_{22}H_{32}BO_2$ $[M+H]^+$ 339.2494; Found: 339.249.

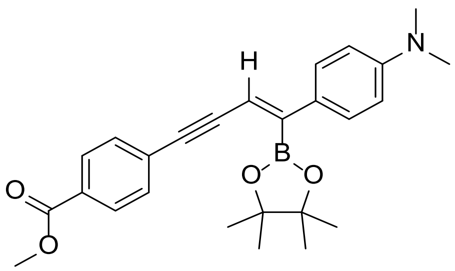

methyl (*E*)-4-(4-(4-(dimethylamino)phenyl)-1-(4,4,5,5-tetramethyl-1,3,2-dioxaborolan-2-yl)but-1-en-3-yn-1-yl)benzoate (2ad)

2 h, yellow solid, 78 mg, 73% yield. **^{1}H NMR** (500 MHz, CD_2Cl_2) δ 7.97 (d, *J* = 8 Hz, 2H), 7.50 (d, *J* = 8 Hz, 2H), 7.32 (d, *J* = 9 Hz, 2H), 6.68 (d, *J* = 9 Hz, 2H), **6.44** (s, 1H), 3.89 (s, 3H), 2.97 (s, 6H), 1.37 (s, 12H). **^{13}C NMR** (126 MHz, CD_2Cl_2) δ 166.3, 150.6, 131.1, 129.3, 129.2, 128.6, 127.8, 127.6, 112.5, 112.0, 93.3, 91.9, 84.1, 52.1, 40.1, 24.8. **^{11}B NMR** (128 MHz, CD_2Cl_2) δ 30.8. **HRMS**: (ESI$^+$) m/z calcd for $C_{26}H_{31}NO_4B$ $[M+H]^+$ 432.2346; Found: 432.2340.

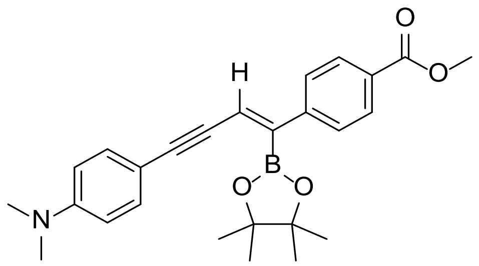

methyl (*E*)-4-(4-(4-(dimethylamino)phenyl)-1-(4,4,5,5-tetramethyl-1,3,2-dioxaborolan-2-yl)but-1-en-3-yn-1-yl)benzoate (2ad1)

1 h, yellow powdery solid, ^{1}H NMR (500 MHz, $CDCl_3$) δ 7.98 (d, *J* = 8 Hz, 2H), 7.47 (d, *J* = 8 Hz, 2H), 7.36 (d, *J* = 9 Hz, 2H), **6.67** (s, 1H), 6.65 (d, *J* = 9 Hz, 2H), 3.91 (s, 3H), 3.00 (s, 6H), 1.39 (s, 12H). **^{13}C NMR** (126 MHz, $CDCl_3$) δ 167.0, 150.2, 145.9, 132.8, 131.1, 129.7, 128.6, 126.7, 123.0, 112.2, 111.7, 98.0, 87.7, 84.2, 52.0, 40.2, 25.0. **^{11}B NMR** (128 MHz, $CDCl_3$) δ 30.3. **HRMS**: (ESI$^+$) m/z calcd for $C_{26}H_{31}NO_4B$ $[M+H]^+$ 432.2346; Found: 432.2340;

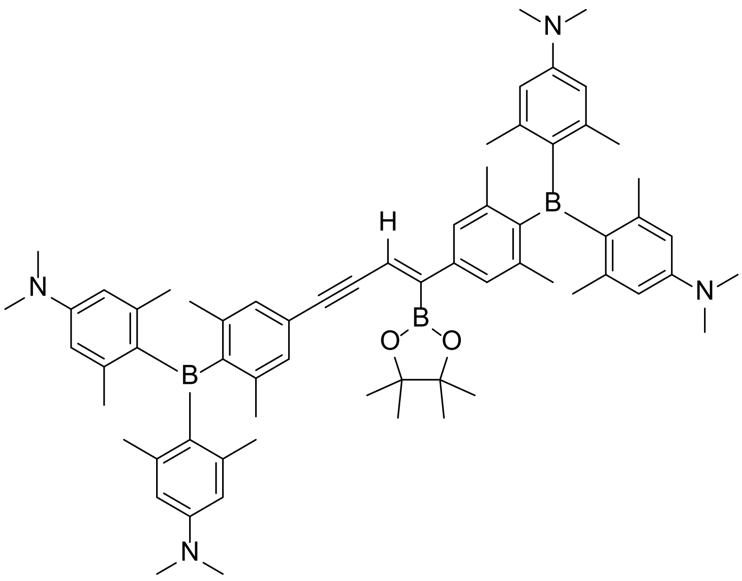

(E)-4,4',4'',4'''-(((1-(4,4,5,5-tetramethyl-1,3,2-dioxaborolan-2-yl)but-1-en-3-yne-1,4-diyl)bis(2,6-dimethyl-4,1-phenylene))bis(boranetriyl))tetrakis(N,N,3,5-tetramethylaniline) (2ae)

16 h (0.42 mmol reaction), yellow sticky solid, 27 mg, 61%. **^{1}H NMR** (500 MHz, CD_2Cl_2) δ 7.01 (s, 2H), 6.97 (s, 2H), **6.63** (s, 1H), 6.31 (s, 8H), 2.95 (s, 24H), 2.03 (s, 6H), 2.01 – 2.00 (m, 18H), 1.95 (s, 6H), 1.92 (s, 6H), 1.38 (s, 12H). **^{13}C NMR** (126 MHz, CD_2Cl_2) δ 151.7, 151.7, 143.4, 143.2, 142.9, 140.7, 140.6, 140.1, 136.1, 135.8, 130.3, 125.8, 123.3, 117.3, 111.8, 95.4, 89.9, 84.5, 40.2, 40.1, 25.1, 24.1, 24.0, 23.8, 23.7, 23.1, 22.8. **^{11}B NMR** (128 MHz, CD_2Cl_2) δ 71.5, 32.2. **HRMS**: (ESI$^+$) m/z calcd for $C_{66}H_{85}B_3N_4O_2K$ $[M+K]^+$ 1037.6589; Found: 1037.6660.

IV Applications

1) Protodeborylation[17]

To a 7 mL vial containing the borylated product (0.15 mmol), AgF (0.30 mmol), and the solvent THF (0.1 mL) were added. To this, methanol (0.67 mL, 1.7 mmol) and H_2O (0.45 mmol) were added at room temperature. The mixture was heated at 60 °C for 1 h. Upon completion, the reaction mixture was diluted with EtOAc (1 mL) and filtered through a short silica plug. After removing the solvent under reduced pressure, the residue was purified by flash column chromatography on silica gel (10% EtOAc/hexanes) to yield the corresponding protodeborylated product.

NMR of **3a** is consistent with the literature.[18]

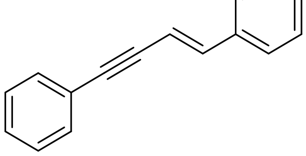

White solid, 25 mg, 81% yield. ^{1}**H NMR** (400 MHz, $CDCl_3$) δ 7.51 – 7.41 (m, 5H), 7.37 – 7.30 (m, 5H), 7.07 (d, *J* = 16 Hz, 1H), 6.39 (d, *J* = 16 Hz, 1H).

2) BF_3 salt formation (3b)[19]

To a 7 mL vial containing the boronate enyne **2a** (100 mg, 0.3 mmol), Et_2O (3 mL) was added. To this, 4.5 M of KHF_2 in water (1.05g of KHF_2, 3mL) was added and the mixture was stirred vigorously overnight at room temperature. The solvents were evaporated, and the residue was recrystallized using acetone and hexane.

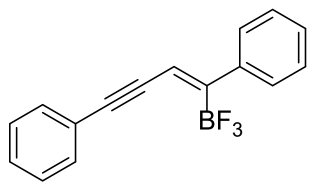

(*E*)-(1,4-diphenylbut-1-en-3-yn-1-yl)trifluoro-borane (3b)

White solid, 42 mg, 52%,^{1}**H NMR** (400 MHz, CD_3CN) δ 7.92 (d, *J* = 8 Hz, 1H), 7.46 – 7.41 (m, 4H), 7.37 – 7.29 (m, 3H), 7.26 – 7.23 (m, 2H), 5.93 (s, 1H). ^{13}**C NMR** (126 MHz, CD_3CN) δ 148.0, 131.9, 129.3, 128.4, 128.2, 128.0, 126.6, 125.9, 112.1, 93.6, 91.0. ^{11}**B NMR** (128 MHz, CD_3CN) δ 2.3 (q, *J* = 60, 58 Hz). **HRMS**: (ESI^-) m/z calcd for $C_{16}H_{11}BF_3$ $[M]^-$ 271.0906; Found: 271.0909.

3) Suzuki-Miyaura coupling[20]

To a flame-dried 7 mL vial, the enyne boronate (**2a**) (150 mg, 0.44 mmol) and 1-iodo-4-methoxy benzene (69 mg, 0.34 mmol) were added. To this, K_3PO_4 (1.0 mmol) and the solvent 1,4-dioxane (5 mL) were added under an argon atmosphere and the vial was purged for 10 min. To this, the catalyst, [1,1'-bis(diphenylphosphino)ferrocene]palladium(II) dichloride dichloromethane complex (1:1) (0.0068 mmol), was added, and the mixture was stirred for 12 h at 100 °C. After cooling to room temperature, water was added to quench the reaction and the mixture was extracted with EtOAc (3x 5 mL). The organic layer was washed with saturated aqueous sodium chloride and dried over anhydrous sodium sulfate, and then the solvent was evaporated under vacuum. This was then purified using flash column chromatography (1-5 % EtOAc/hexanes) to yield **3c**.

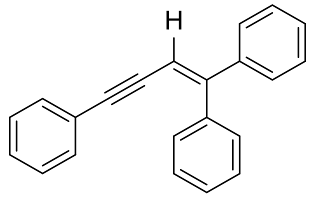

but-1-en-3-yne-1,1,4-triyltribenzene (3c)

Yellow liquid, 54 mg, 57% yield. ^{1}**H NMR** (500 MHz, $CDCl_3$) δ 7.58 – 7.51 (m, 3H), 7.46 – 7.35 (m, 4H), 7.30 – 7.25 (m, 8H), 6.24 (s, 1H). ^{13}**C NMR** (126 MHz, $CDCl_3$) δ 152.7, 141.4, 139.2, 131.4, 130.2, 128.3, 128.3, 128.3, 128.2, 128.1, 128.0, 127.8, 123.6, 107.1, 93.7, 89.2.

HRMS: (ESI^+) m/z calcd for $C_{22}H_{17}$ $[M+H]^+$ 281.1330; Found: 281.1345.

4) Enediyne synthesis (3d)[21]

To a 7 mL vial, containing 0.18 mmol (59 mg) of **2a**, tetrakis(triphenylphosphine) palladium(0) (10 mg, 0.0089 mmol), 1-iodohexyne (37 mg, 0.18 mmol) and NaOH (14 mg, 0.36 mmol) were added. To this, 0.2 mL of THF was added and the mixture was stirred overnight at 80 °C. After cooling to room temperature, water was added to quench the reaction and extracted with EtOAc (3x 5 ml). The organic layer was washed with saturated aqueous sodium chloride and dried over anhydrous sodium sulfate, and then the solvent was evaporated under vacuum. This was then purified using flash column chromatography (0 - 2.5% EtOAc/hexane) to yield the **3d**.

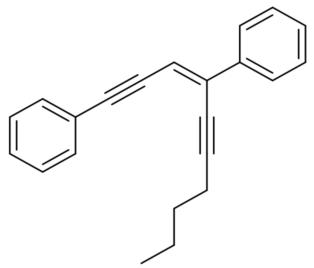

(*Z*)-deca-3-en-1,5-diyne-1,4-diyldibenzene (3d)

Transparent liquid, 23 mg, 45%. **^{1}H NMR** (500 MHz, $CDCl_3$) δ 7.70 – 7.69 (m, 2H), 7.54 – 7.52 (m, 2H), 7.42 – 7.31 (m, 6H), 6.49 (s, 1H), 2.60 (t, J = 7.0 Hz, 2H), 1.72 – 1.66 (m, 2H), 1.59 - 1.53 (m, 2H), 0.95 (t, J = 7 Hz, 3H). **^{13}C NMR** (126 MHz, $CDCl_3$) δ 137.7, 134.4, 132.1, 129.0, 128.9, 128.7, 126.5, 124.0, 112.9, 100.6, 97.5, 89.4, 79.1, 31.3, 22.5, 20.3, 14.1. **HRMS**: (ESI^-) m/z calcd for $C_{22}H_{19}$ $[M-H]^-$ 283.1486; Found: 283.1496.

5) Labeling

Following the procedure to synthesize the enyne boronate **2q**, to the reaction mixture, 10 equiv of MeOD was added and the reaction was heated at 60 °C for 12h. The reaction mixture was filtered through a short silica plug with EtOAc as the eluent and concentrated under reduced pressure. The resulting residue was purified using flash column chromatography on silica gel (10% EtOAc/hexanes) to yield **3e**.

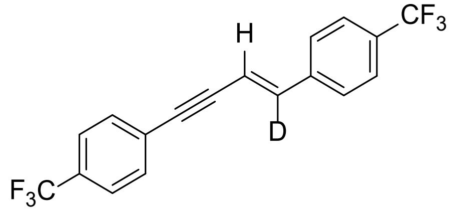

(*E*)-4,4'-(but-1-en-3-yne-1,4-diyl-1-d)bis((trifluoromethyl)benzene) (3e)

Pale yellow solid, 31 mg, 87% yield, **^{1}H NMR** (400 MHz, $CDCl_3$) δ 7.71 – 7.46 (m, 8H), 6.47 (s, 1H). **^{13}C NMR** (126 MHz, $CDCl_3$) δ141.4 - 141.02 (t, *J* = 25 Hz), 140.2, 132.7, 131.4 -130.7 (q, *J* = 32 Hz), 131.04 -130.27 (q, *J* = 32 Hz), 128.2 – 121.7 (q, *J* = 272 Hz), 128.1 – 121.6 (q, *J* = 273 Hz), 127.8, 127.5, 126.6 - 126.5 (q, *J* = 4 Hz), 126.2 - 125.9 (q, *J* = 4 Hz), 110.9, 92.1, 91.3. **^{19}F NMR** (376 MHz, $CDCl_3$) δ -62.72, -62.86. **HRMS**: (ESI^-) m/z calcd for $C_{36}H_{18}D_2ClF_{12}$ $[2M+Cl]^-$ 717.1187; Found : 717.1177.

Synthesis of the protodeborylated product of 2g'

Following the procedure to synthesize the enyne boronate **2g'**, to the reaction mixture, 10 equiv of MeOH were added and the mixture was heated at 60 °C for 12 h. The reaction mixture was filtered through a short silica plug with EtOAc as the eluent and concentrated under reduced pressure. The resulting residue was purified using flash column chromatography on silica gel (10% EtOAc/hexane) to yield **3g'**.

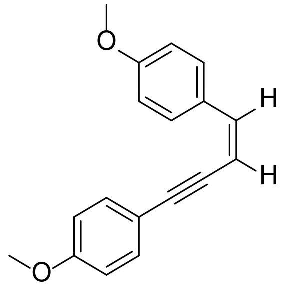

(*Z*)-2-(1,4-bis(4-methoxyphenyl)but-1-en-3-yn-2-yl)-4,4,5,5-tetramethyl-1,3,2-dioxaborolane (3g')[22]

^{1}H NMR (400 MHz, $CDCl_3$) δ 7.89 (d, *J* = 9 Hz, 2H), 7.43 (d, *J* = 9 Hz, 2H), 6.92 - 6.87 (m, 4H), 6.59 (d, *J* = 12 Hz, 1H), 5.78 (d, *J* = 12 Hz, 1H), 3.84 (s, 3H), 3.83 (s, 3H). **^{13}C NMR** (126 MHz, $CDCl_3$) δ 159.6, 137.4, 132.9, 130.2, 129.8, 115.8, 114.1, 113.6, 105.2, 95.4, 87.4, 55.3, 55.3.

V Confirmation of *cis*- and *trans*-isomers of 2a

Reaction:

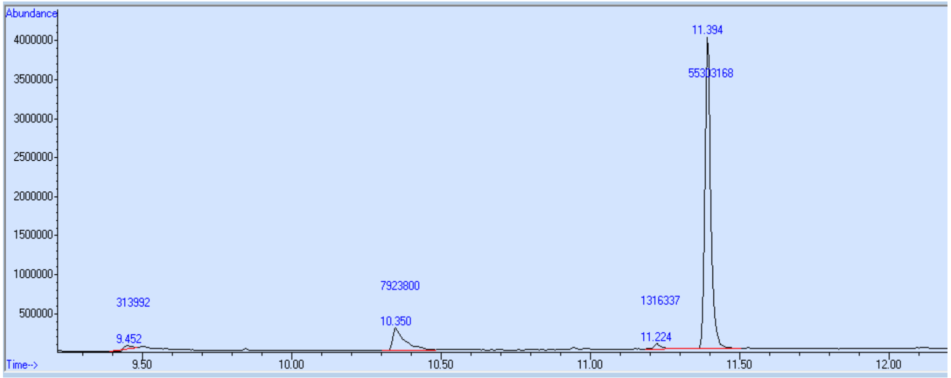

11.4 min : 2a, 11.2 : 2a – *Z* isomer, 10.4 min – protodeborylated product, 9.5 min- residual starting material.

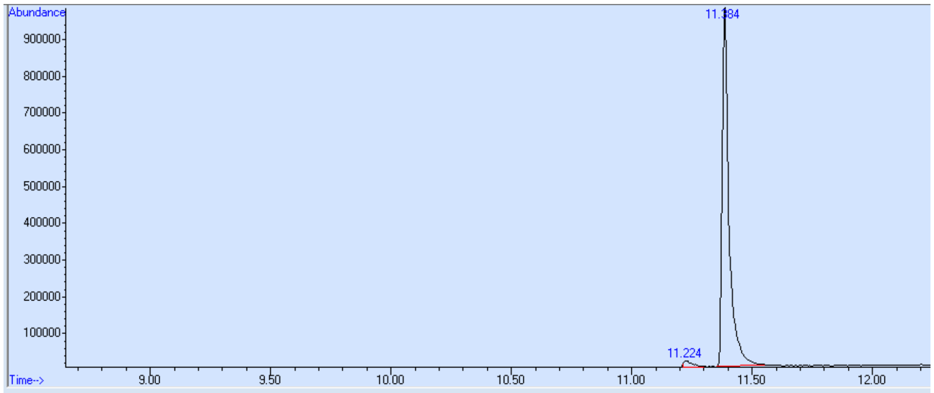

11.4 min - 2a (with 3% minor product (2a- *Z* isomer))

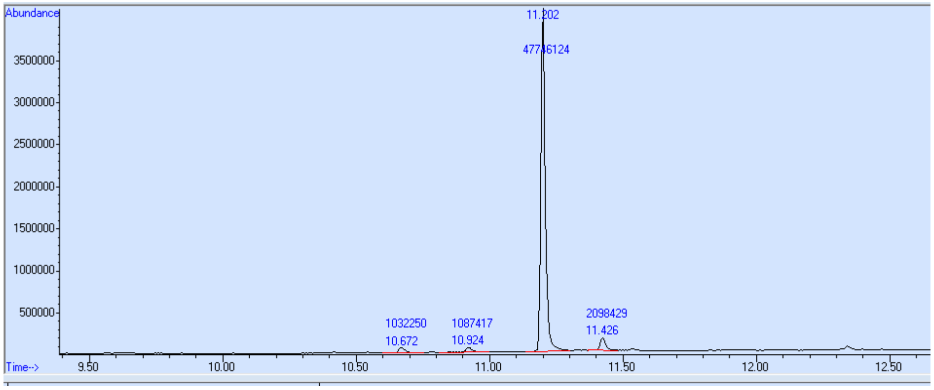

11.2 min – 2a- *Z* isomer (with 4% 2a and (residual protodeborylated product at 10.7 min))

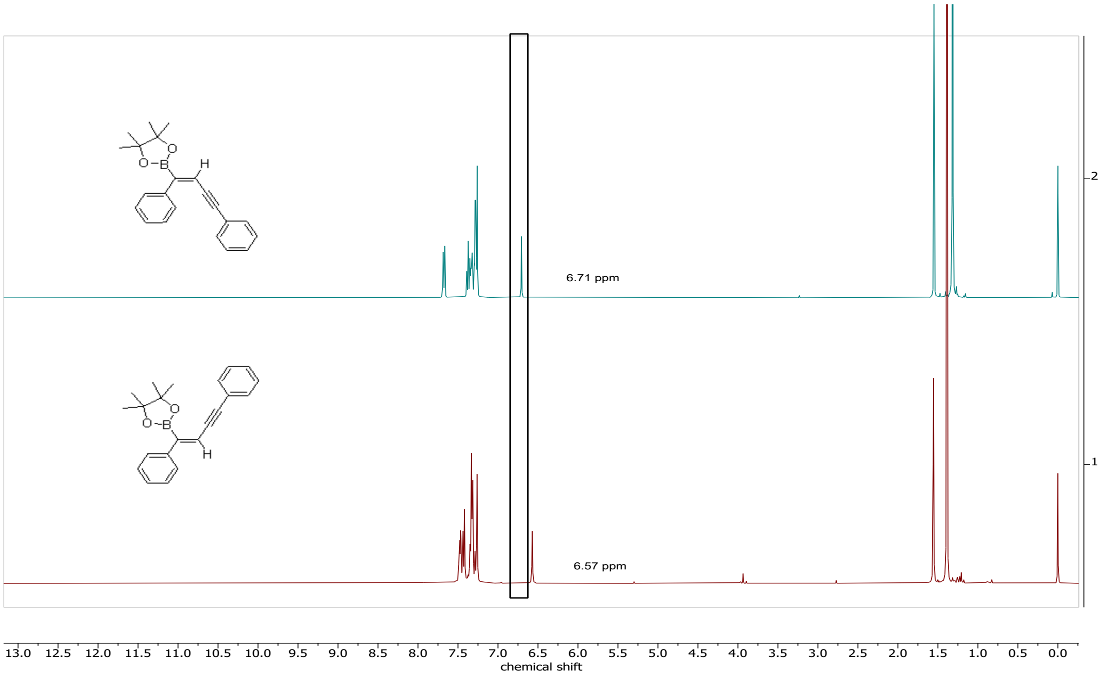

Selectivity during the reaction (^{1}H NMR)

Procedure: NMR experiments were conducted in toluene-d_8 at 70 °C in an NMR tube. The phenyl substrate **1a** (0.25 mmol) was used for the experiment. H-Bpin (0.3 mmol) and PBu_3 (0.025 mmol) was added. The NMR tube was heated to 70 °C in the spectrometer, and spectra were recorded at specific intervals.

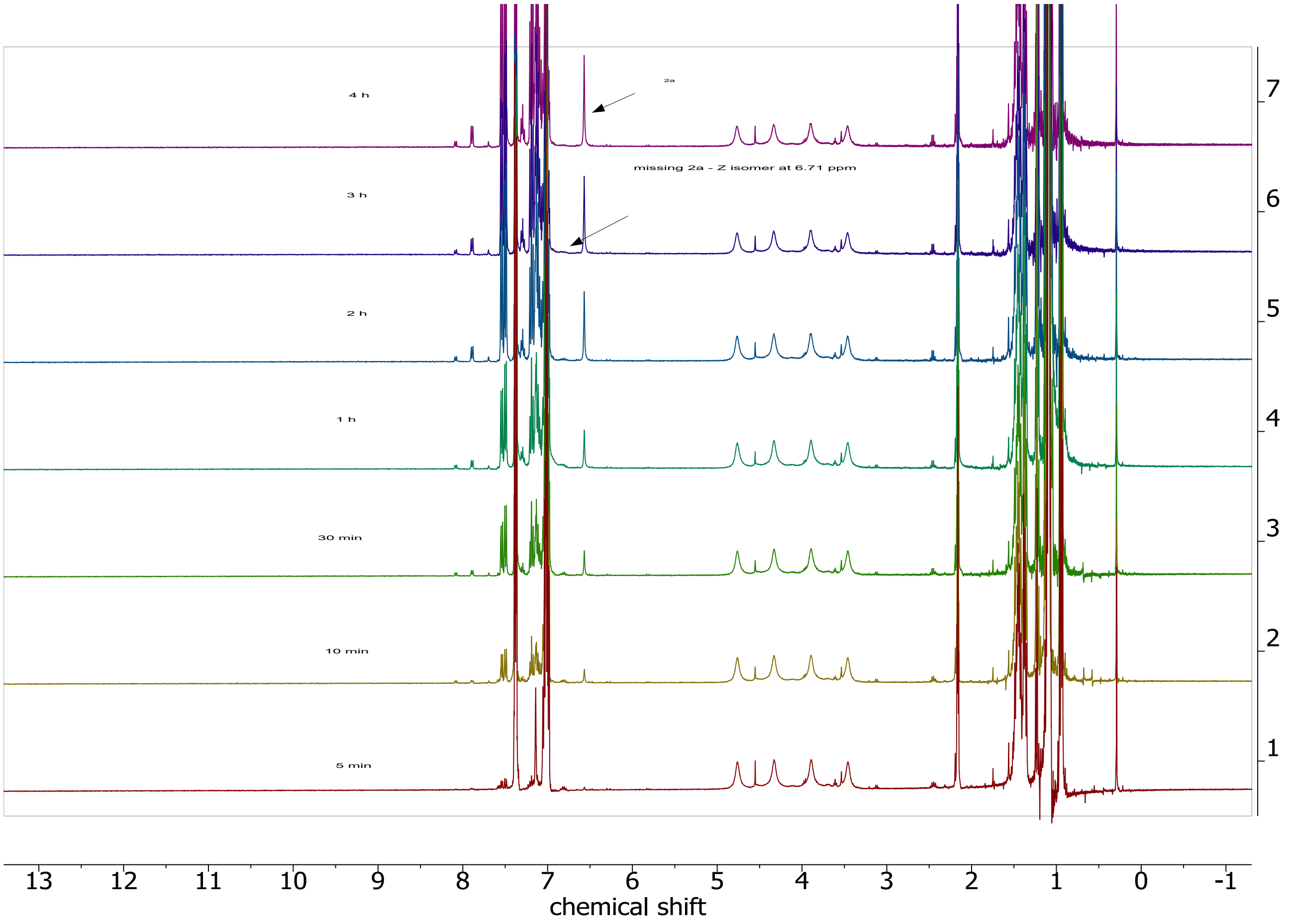

VI Color changes during the reaction.

Figure 1: Color changes during the reaction a) **2q**, b) **2e**

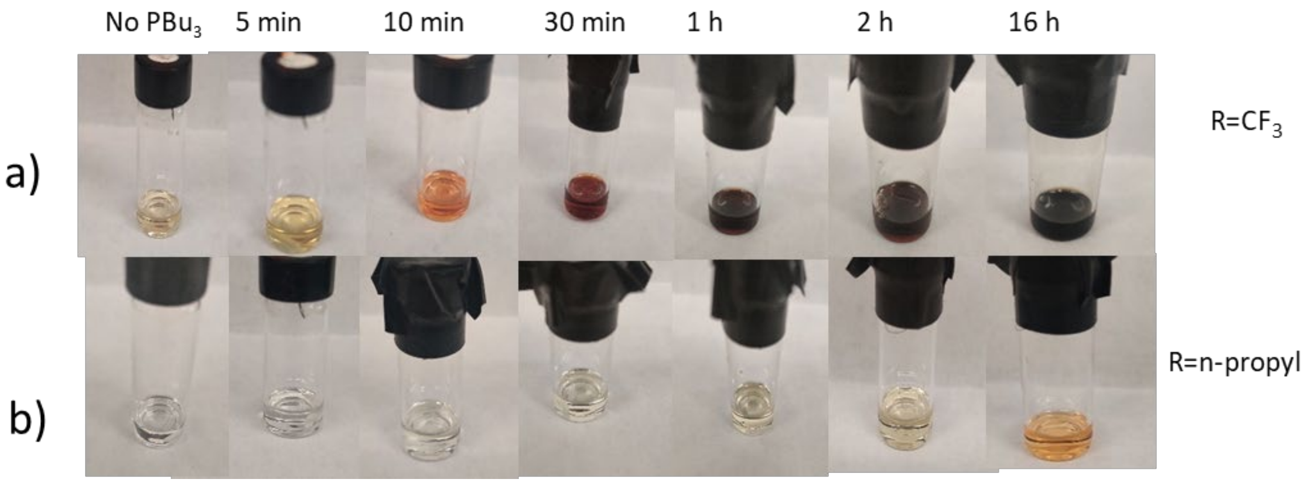

VII *In situ* NMR studies

Pinacolborane (HBpin) and tributyl phosphine oxide (O=PnBu$_3$) were purchased from Sigma Aldrich and PnBu$_3$ from abcr, all were used without further purification. Toluene-d$_8$ was purchased from Sigma Aldrich, dried by storing over molecular sieves for several days, and then degassed under vacuum in an ultrasonic bath. All reactions were conducted, and all NMR-spectra were recorded in 0.5 mL of toluene-d$_8$ under argon atmosphere. NMR spectra were recorded on a Bruker Avance 500 MHz (^{1}H: 500 MHz, ^{13}C: 125 MHz, ^{11}B: 160 MHz, ^{31}P: 202 MHz, ^{19}F: 470 MHz), on a Bruker Avance 300 MHz (^{1}H: 300 MHz, ^{11}B: 96 MHz, ^{13}C: 75 MHz, ^{31}P: 121 MHz) and on a Bruker Avance Neo I 600 (^{1}H: 600 MHz, ^{11}B: 192 MHz, ^{19}F: 564 MHz, ^{13}C: 150 MHz, ^{31}P: 242 MHz). All ^{1}H- and ^{13}C{^{1}H}-NMR spectra are reported relative to TMS, using the residual signals of toluene-d$_8$. The ^{19}F{^{1}H}-NMR spectra are reported relative to the external standard CFCl$_3$. The ^{31}P{^{1}H}-NMR spectra are reported relative to the external standard H$_3$PO$_4$. The ^{11}B{^{1}H}-NMR spectra are reported relative to the external standard BF$_3$·Et$_2$O. All scales of the depicted NMR spectra are given in *ppm*. High resolution mass spectrometry (HRMS) was performed using a Thermo Fischer Scientific Exactive Plus Orbitrap system with a LIDFI (Liquid Injection Field Desorption Ionization) or APCI (atmospheric pressure chemical ionization) probe. GCMS analyses were performed on an Agilent Technologies GCMS system (GC 7890A, EI-MS 5975C). UV/Vis absorption spectra were recorded in n-hexane in standard quartz cuvettes (1 cm x 1 cm cross-section) using an Agilent 8453 diode array UV-visible spectrophotometer.

Spectra of starting material

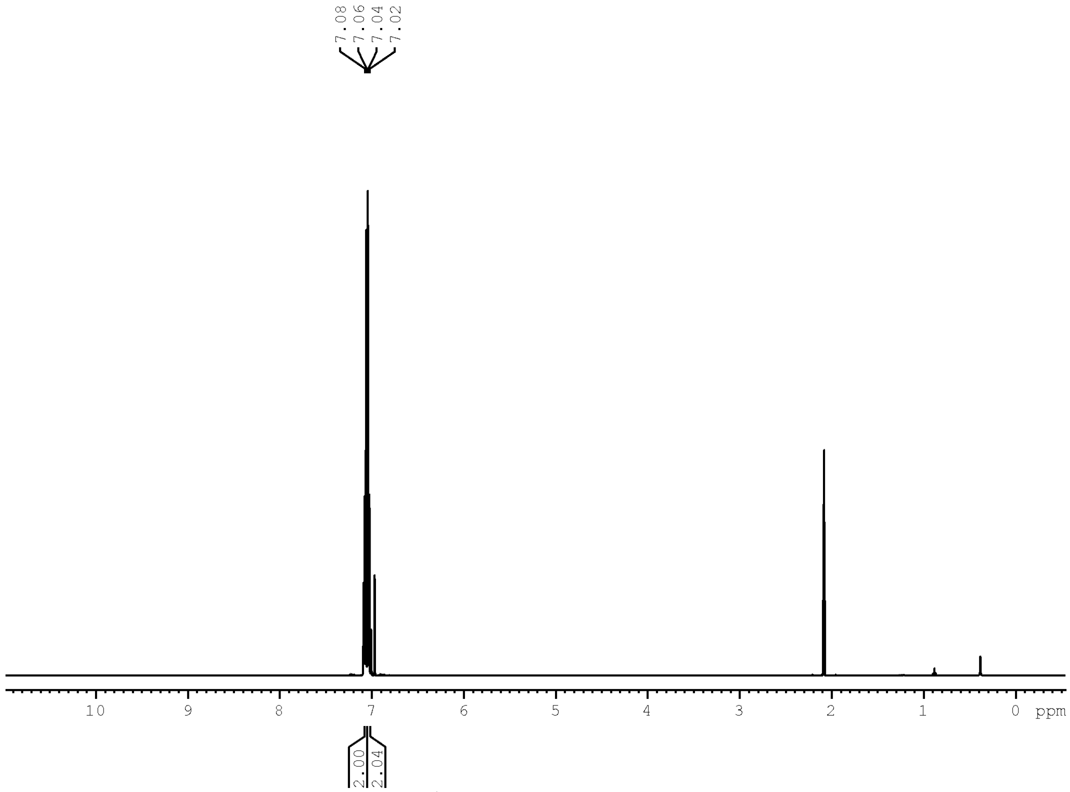

Figure 1: ^{1}H-NMR (500 MHz) of **1q**.

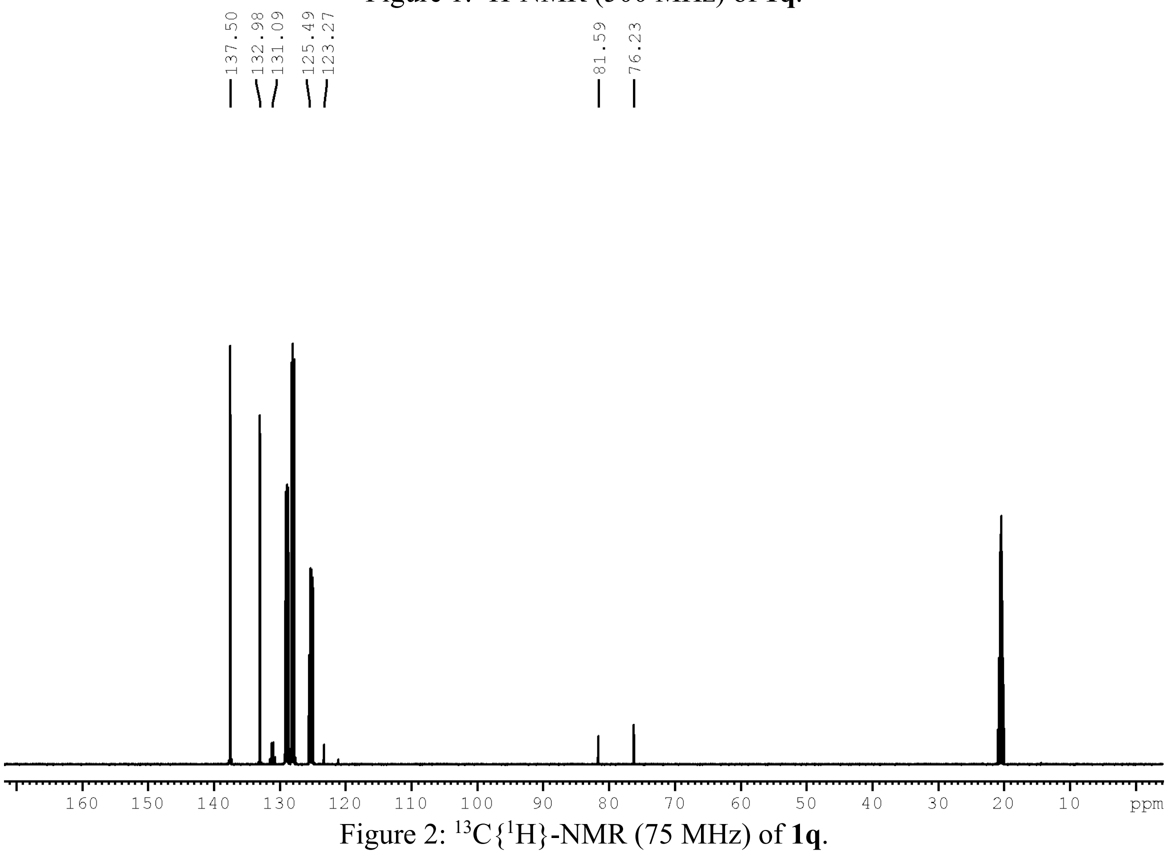

Figure 2: ^{13}C{^{1}H}-NMR (75 MHz) of **1q**.

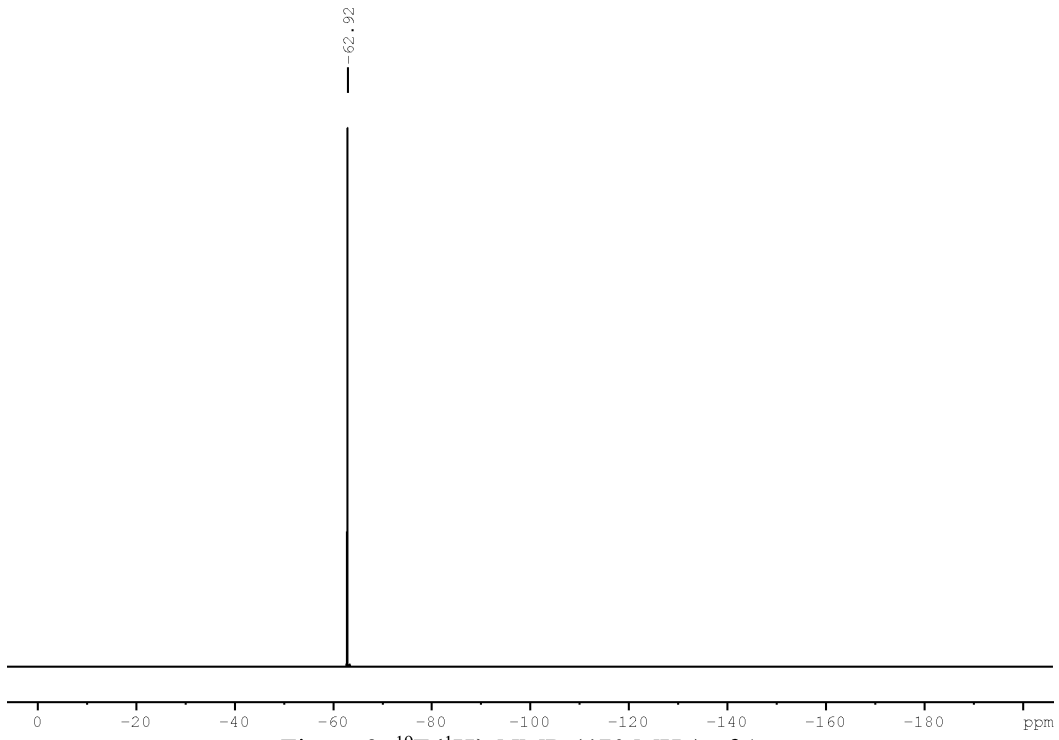

Figure 3: $^{19}F\{^1H\}$-NMR (470 MHz) of **1q**.

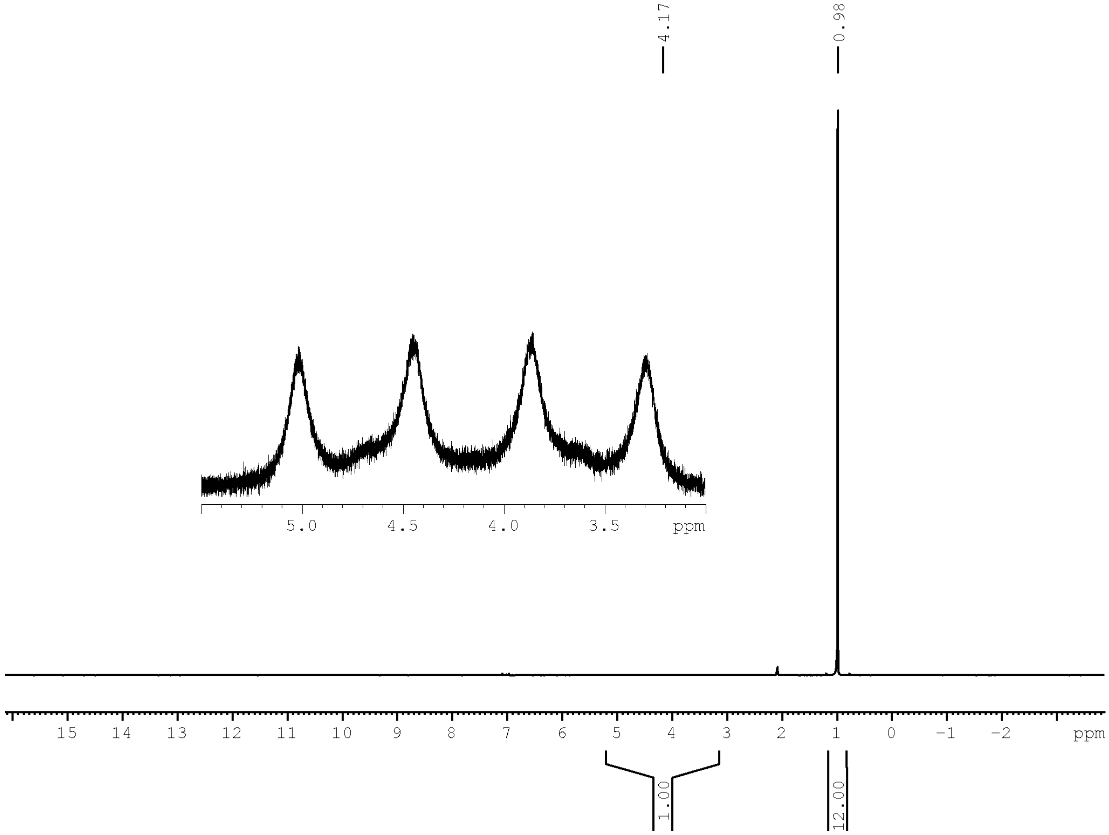

Figure 4: ^1H-NMR (300 MHz) of HBpin.

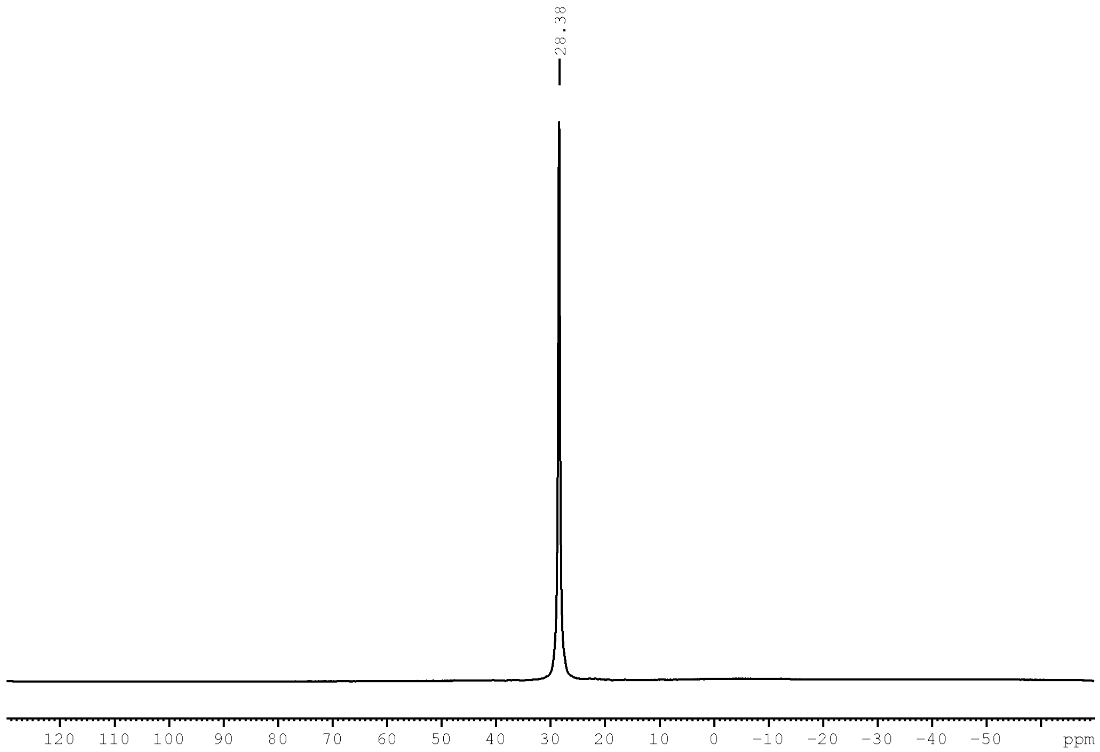

Figure 5: $^{11}B\{^{1}H\}$-NMR (96 MHz) of HBpin.

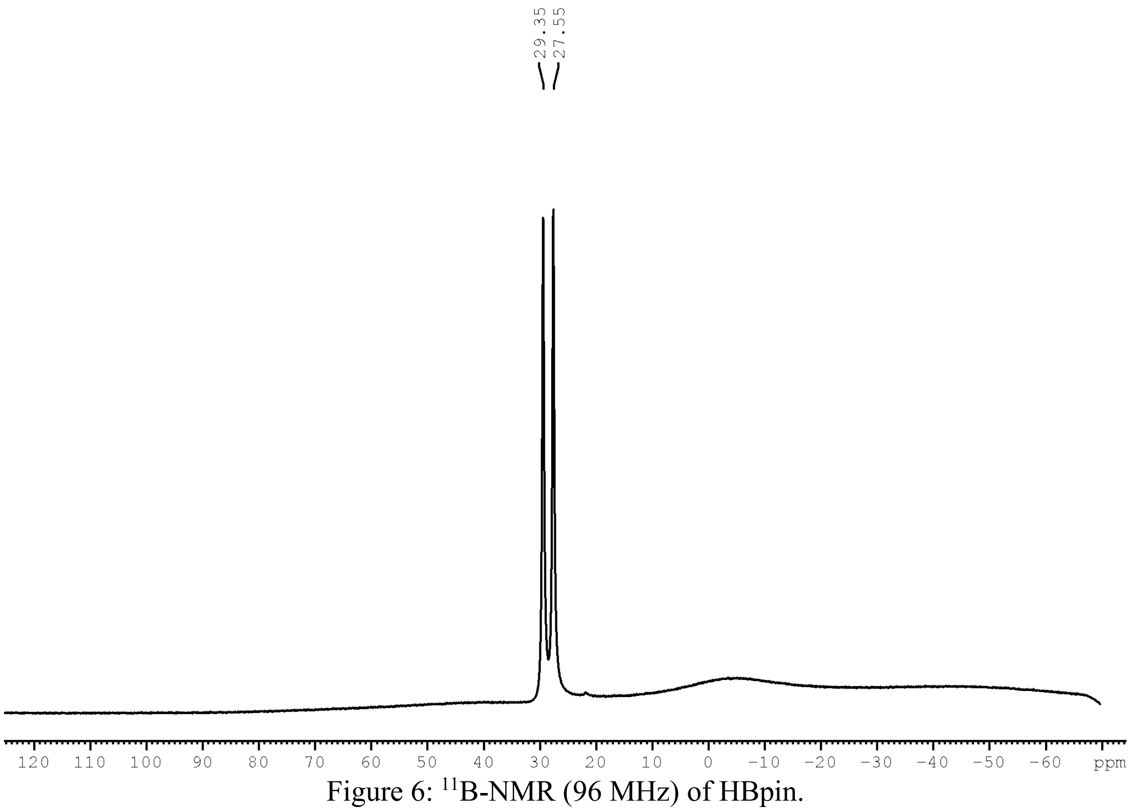

Figure 6: ^{11}B-NMR (96 MHz) of HBpin.

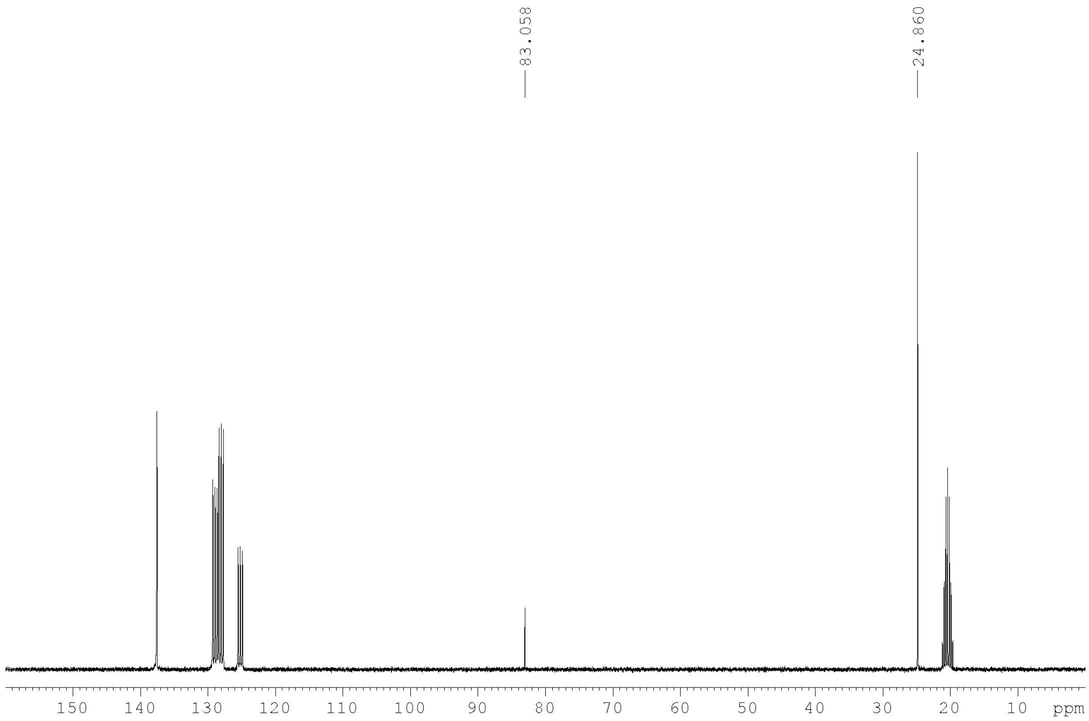

Figure 7: $^{13}C\{^{1}H\}$-NMR (75 MHz) of HBpin.

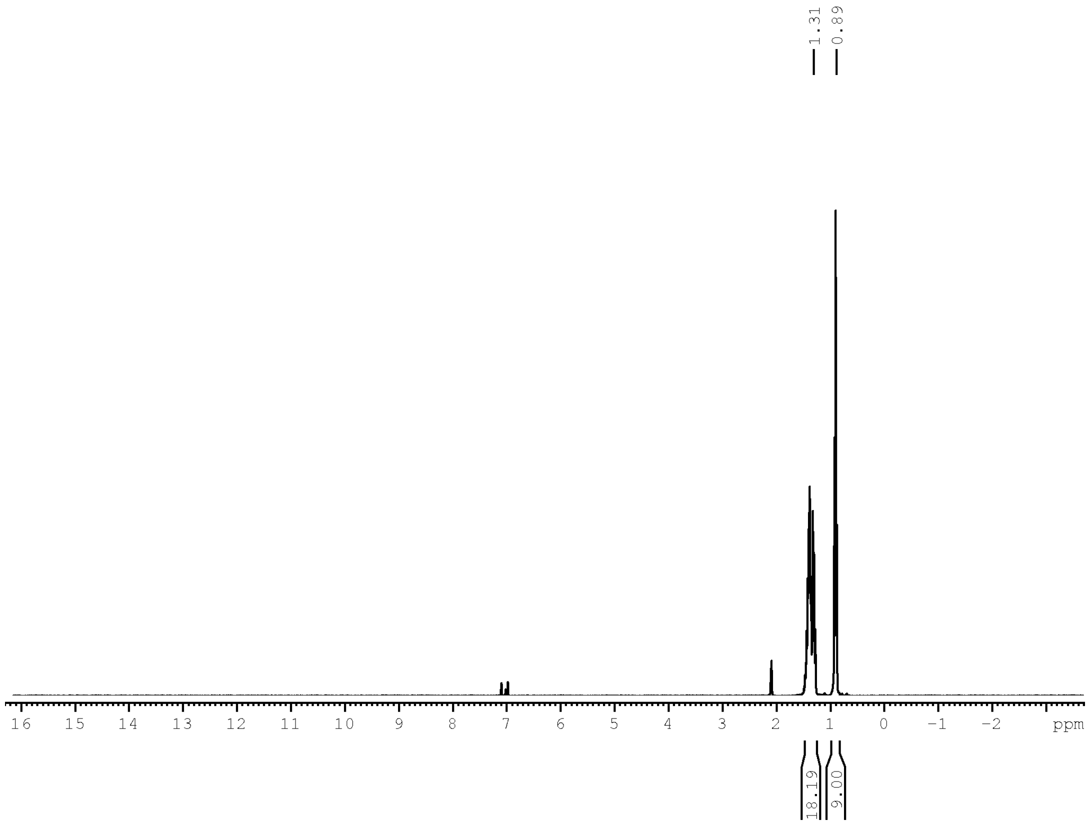

Figure 8: ^{1}H-NMR (300 MHz) of $PnBu_3$.

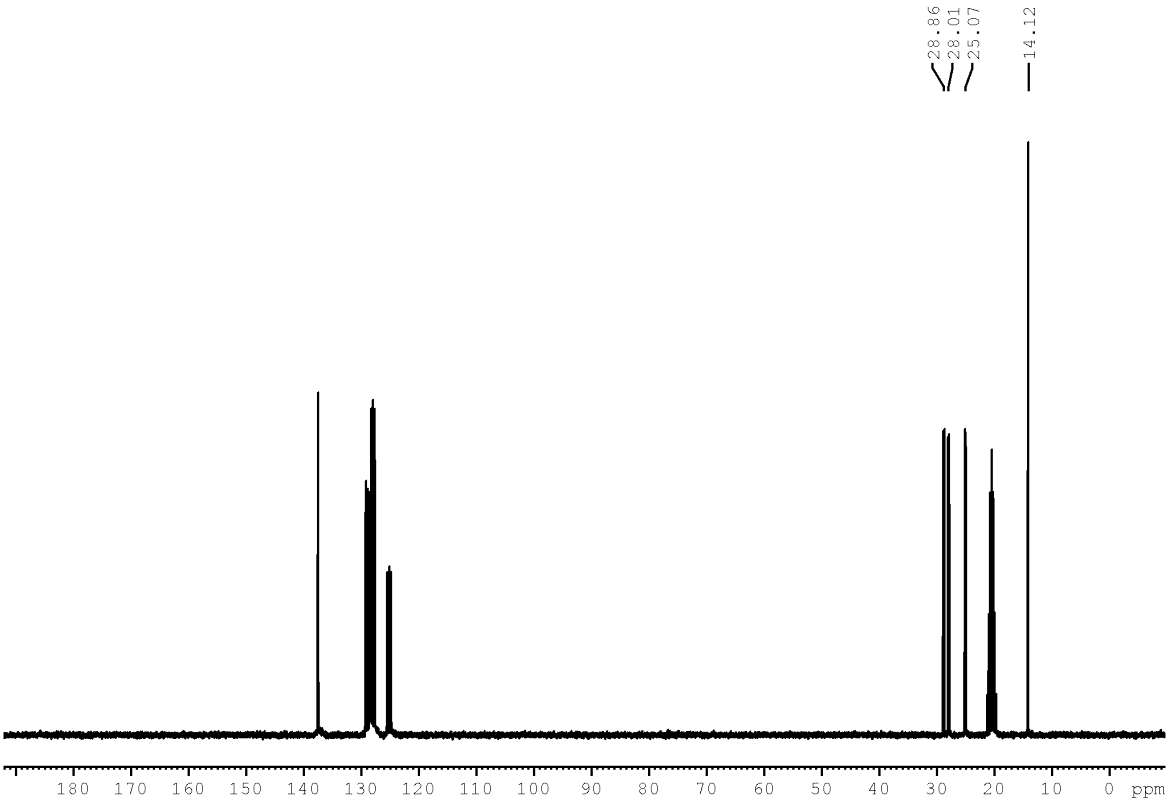

Figure 9: $^{13}C\{^{1}H\}$-NMR (125 MHz) of $PnBu_3$.

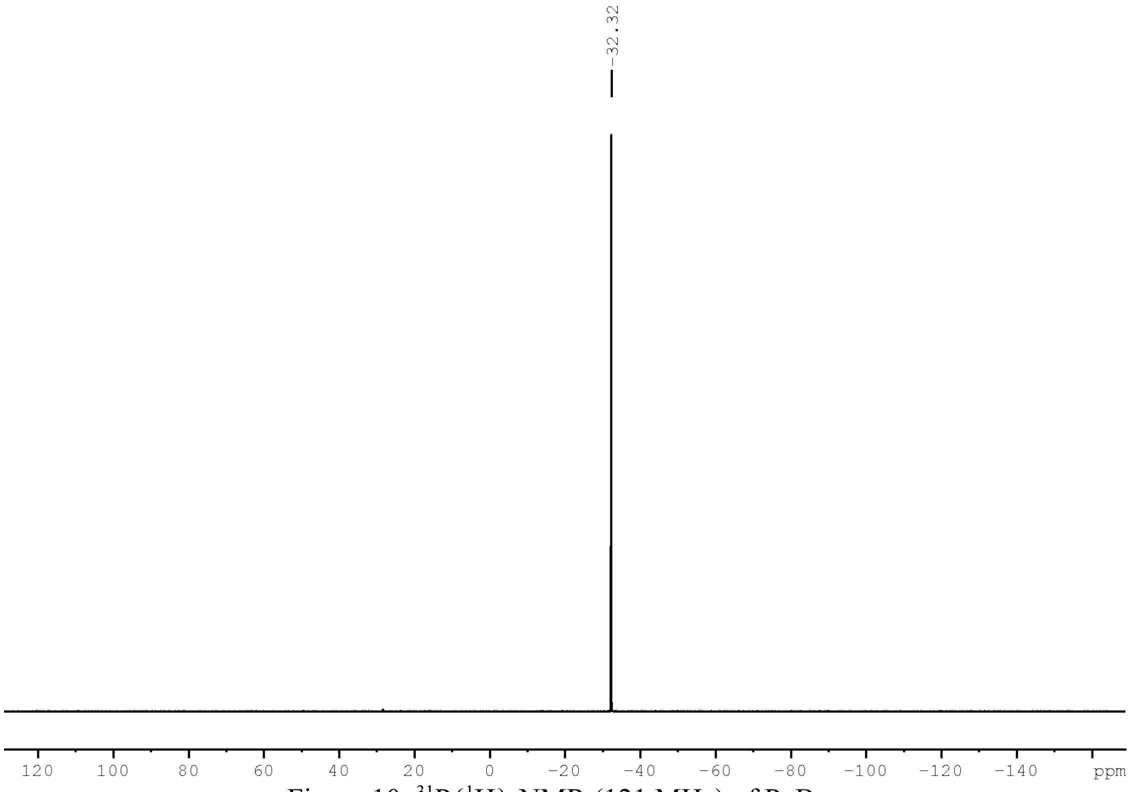

Figure 10: $^{31}P\{^{1}H\}$-NMR (121 MHz) of $PnBu_3$.

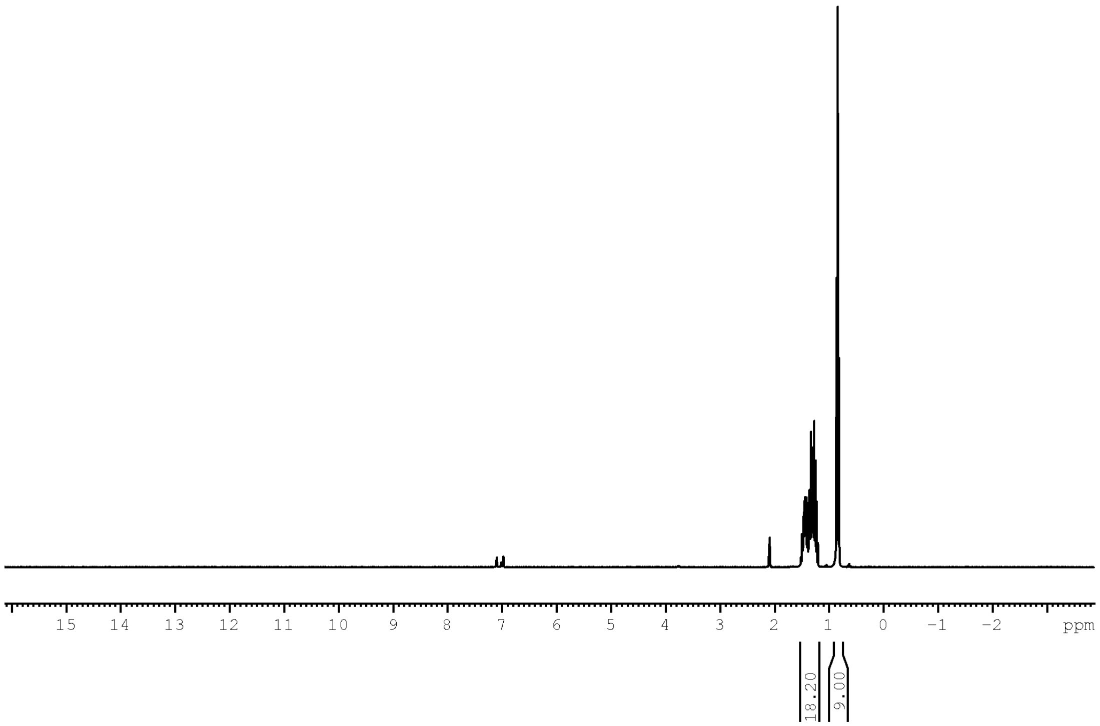

Figure 11: ^{1}H-NMR (300 MHz) of O=PnBu$_3$.

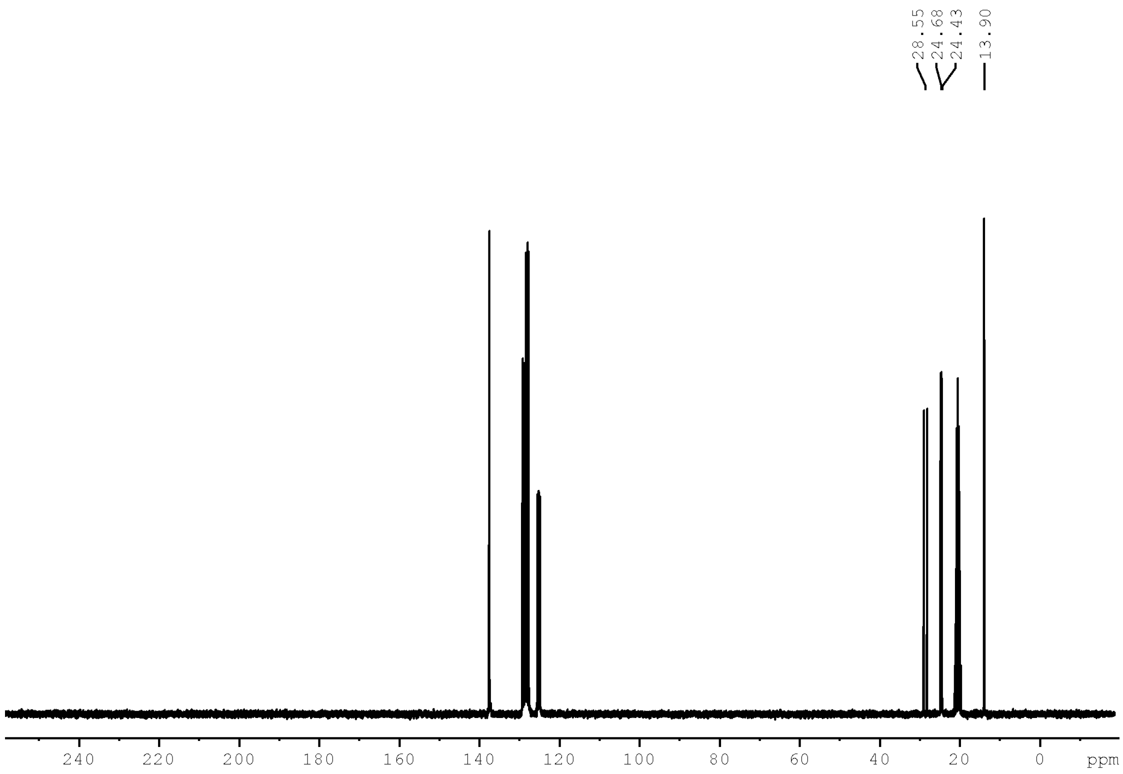

Figure 12: ^{13}C{^{1}H}-NMR (125 MHz) of O=PnBu$_3$.

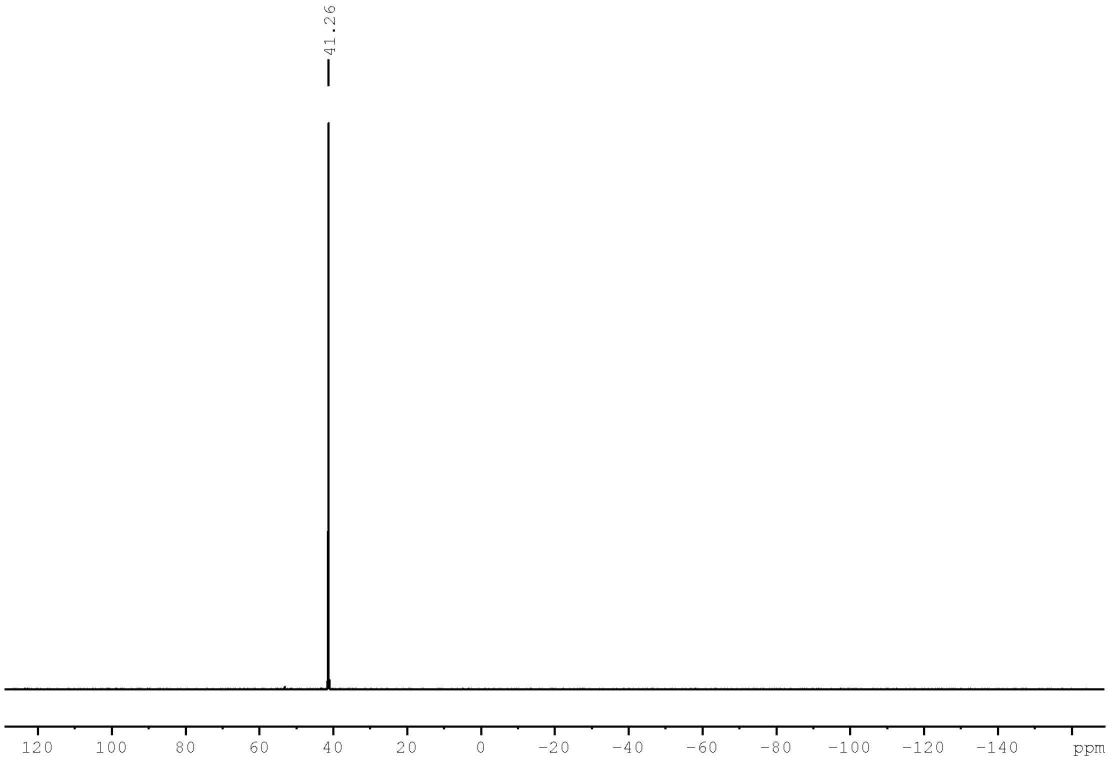

Figure 13: $^{31}P\{^{1}H\}$-NMR (121 MHz) of O=PnBu$_3$.

Diyne with HBpin, without catalyst

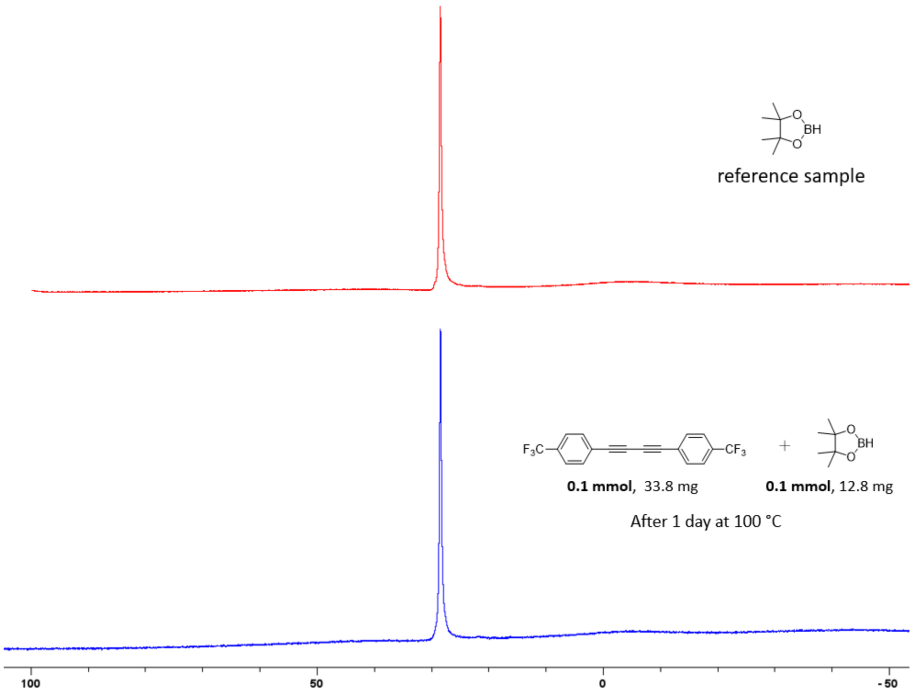

Figure 14: $^{11}B\{^{1}H\}$-NMR (96 MHz) of a mixture of diyne **1q** (0.1 mmol) with HBpin (0.1 mmol) after heating at 100 °C for 1 d (blue) and a reference spectrum of HBpin (red).

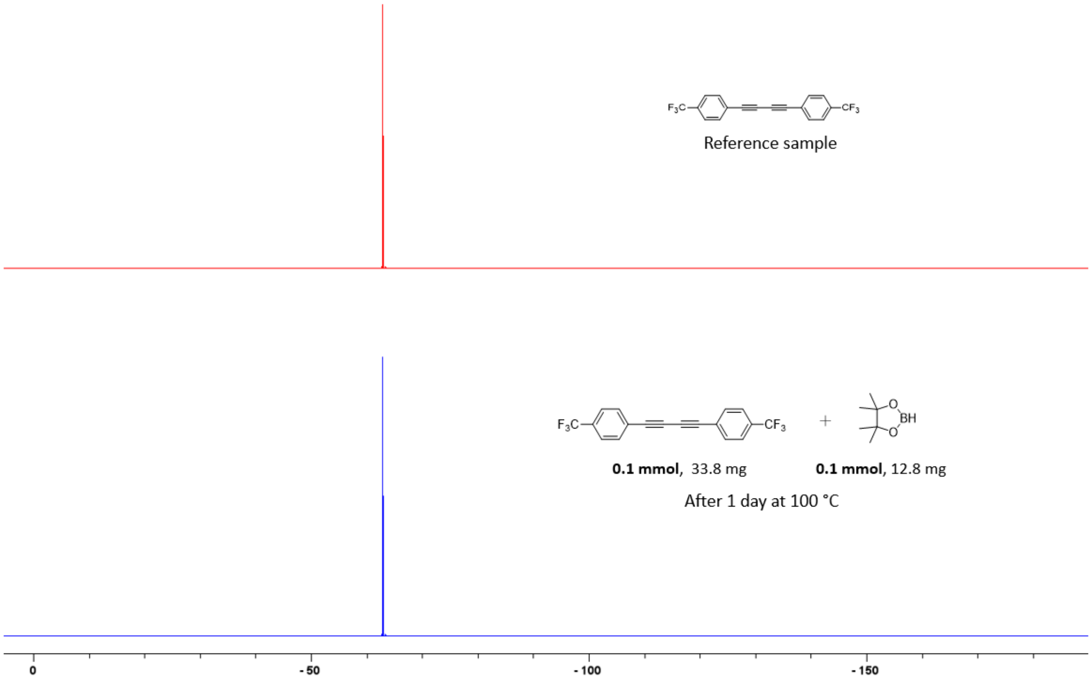

Figure 15: $^{19}F\{^{1}H\}$-NMR (470 MHz) of a mixture of **1q** (0.1 mmol) and HBpin (0.1 mmol) after heating at 100 °C for 1 d (blue) and a reference spectrum of **1q** (red).

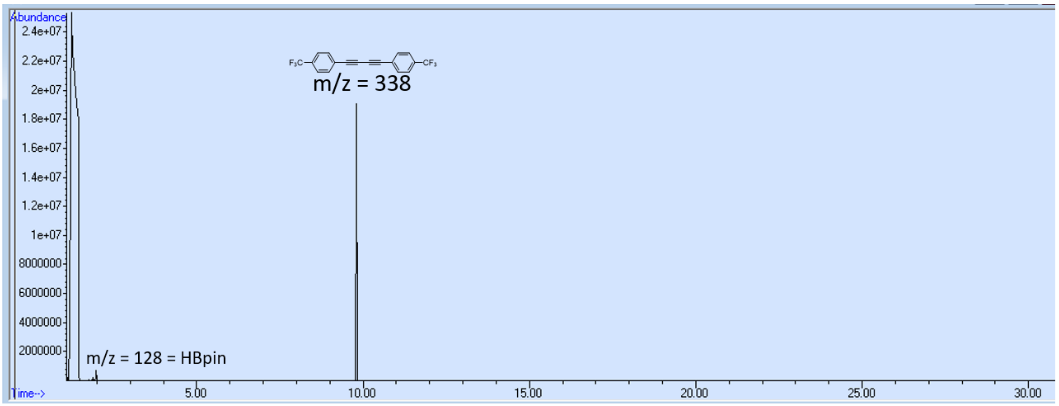

Figure 16: GCMS of a mixture of **1q** (0.1 mmol) and HBpin (0.1 mmol) after heating at 100 °C for 1 d.

<u>**HBpin thermal stability control**</u>

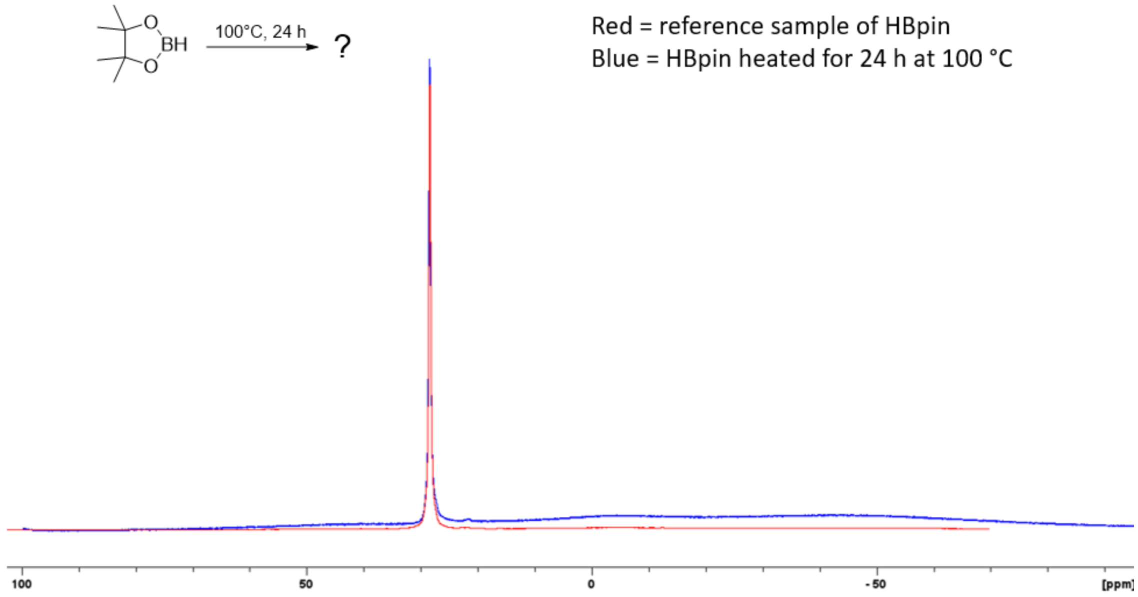

Figure 17: $^{11}B\{^{1}H\}$-NMR (96 MHz) of HBpin (0.1 mmol, 12.8 mg) after 24 h at 100 °C (blue) and of a HBpin reference sample (red).

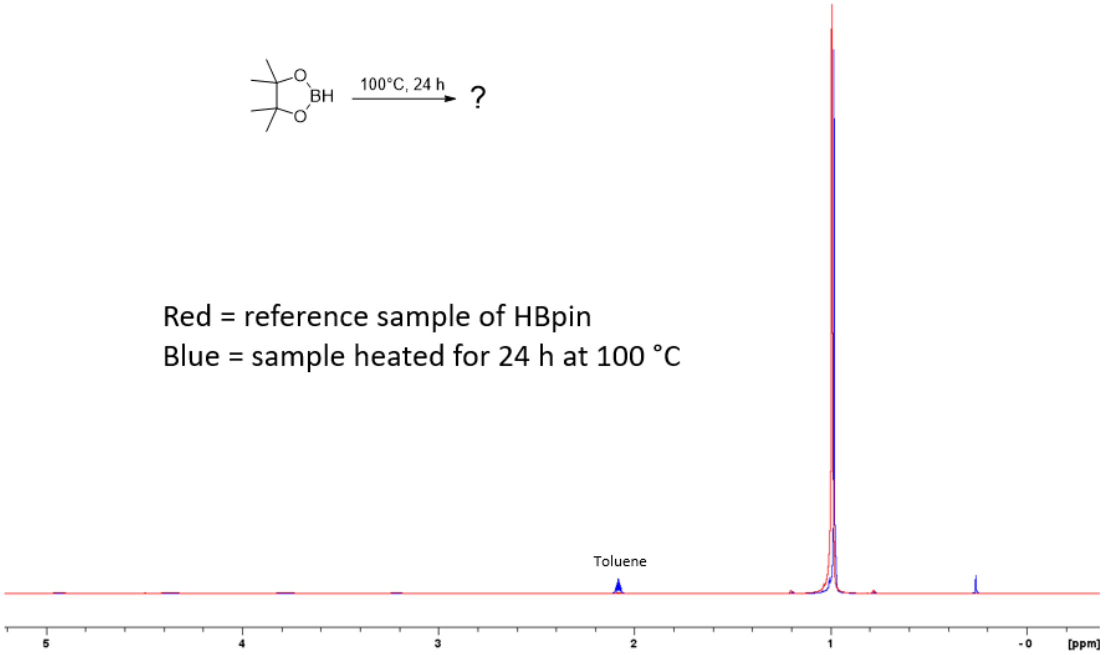

Figure 18: ^{1}H-NMR (300 MHz) of HBpin (0.1 mmol, 12.8 mg) after 24 h at 100 °C (blue) and of a HBpin reference sample (red).

HBpin with Phosphine

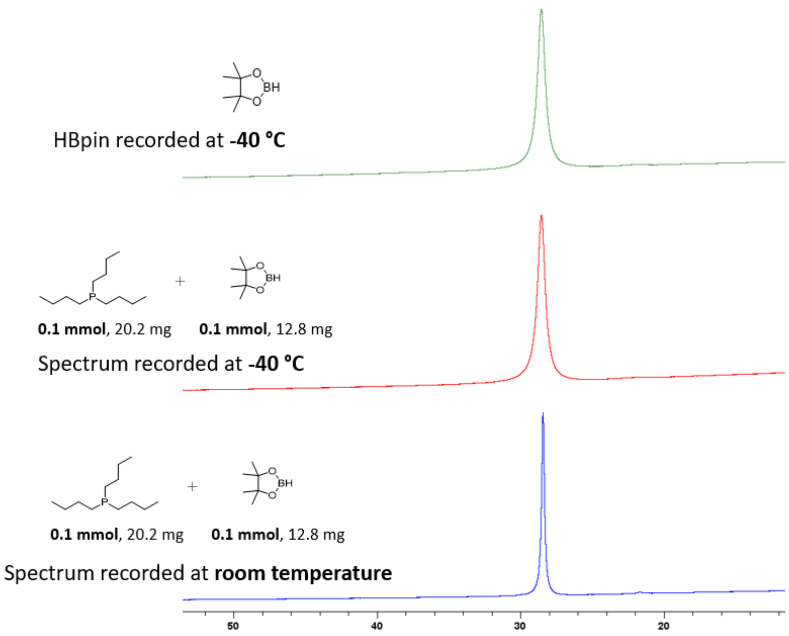

Figure 19: $^{11}B\{^{1}H\}$ (160 MHz) NMR spectra of a mixture of $PnBu_3$ and HBpin, after 1 d at room temperature, recorded at room temperature (blue) and at -40 °C (red) and a reference spectrum of HBpin (-40 °C, green).

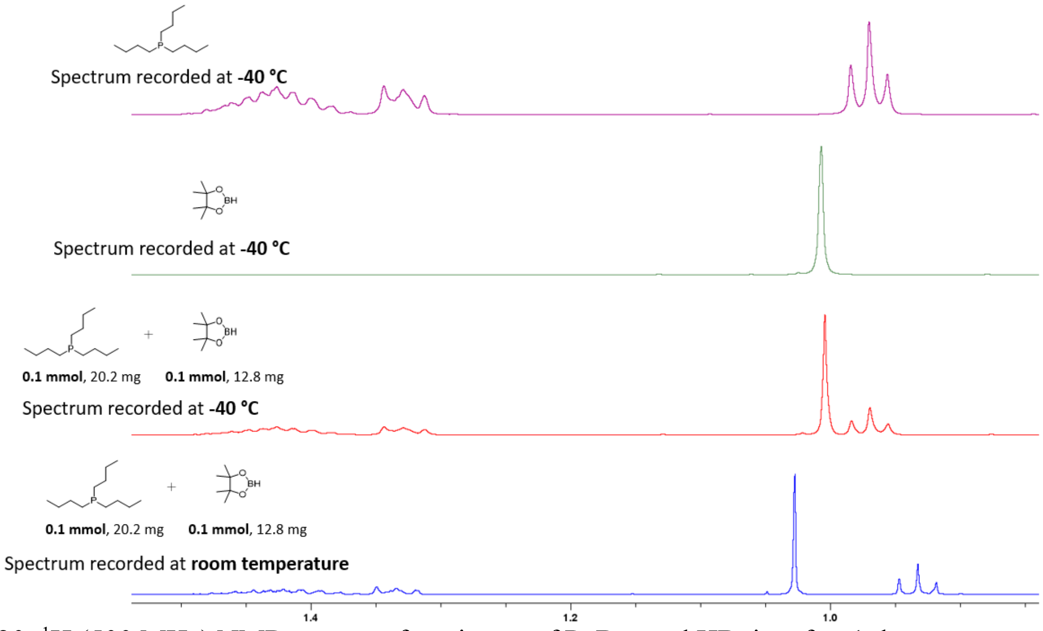

Figure 20: ^{1}H (500 MHz) NMR spectra of a mixture of $PnBu_3$ and HBpin, after 1 d at room temperature, recorded at room temperature (blue) and at -40 °C (red) and reference spectra of HBpin (-40 °C, green) and $PnBu_3$ (-40 °C, purple).

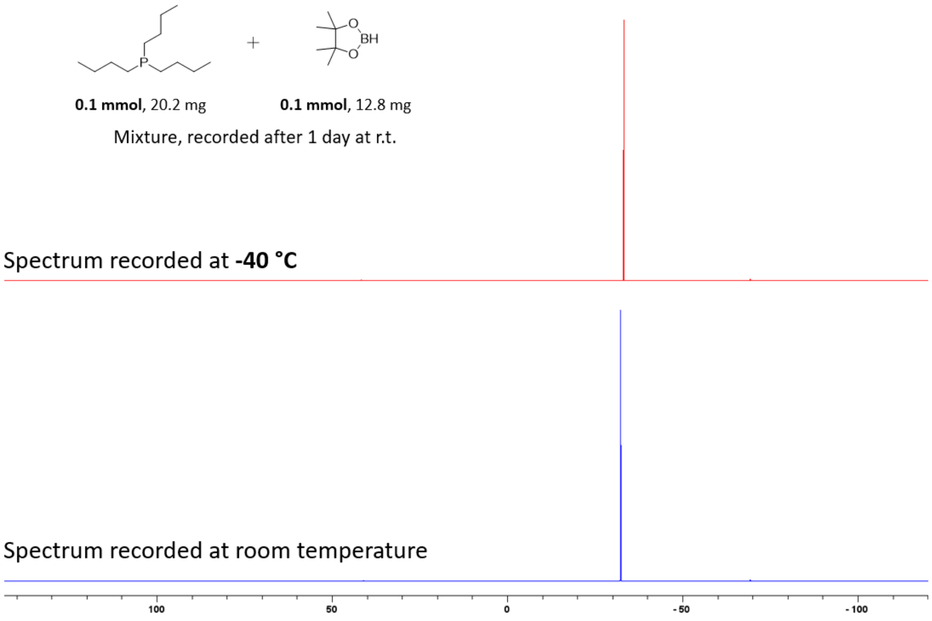

Figure 21: $^{31}P\{^{1}H\}$ (202 MHz)-NMR of a mixture of $PnBu_3$ and HBpin after 1 d at room temperature. Spectra were recorded at room temperature (blue) and at -40 °C (red). The signal is the unchanged phosphine.

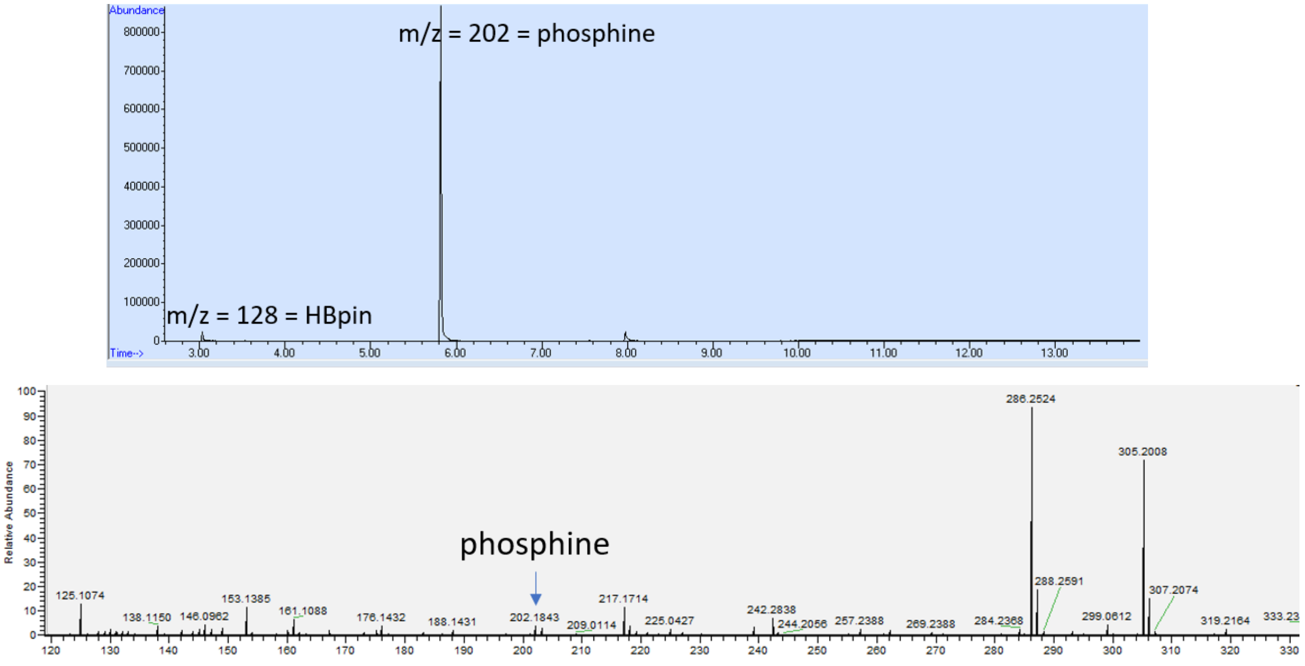

Figure 22: GCMS (above) and ESI+ HRMS (below) of a mixture of $PnBu_3$ and HBpin, after 1 d at room temperature. No adduct was detected.

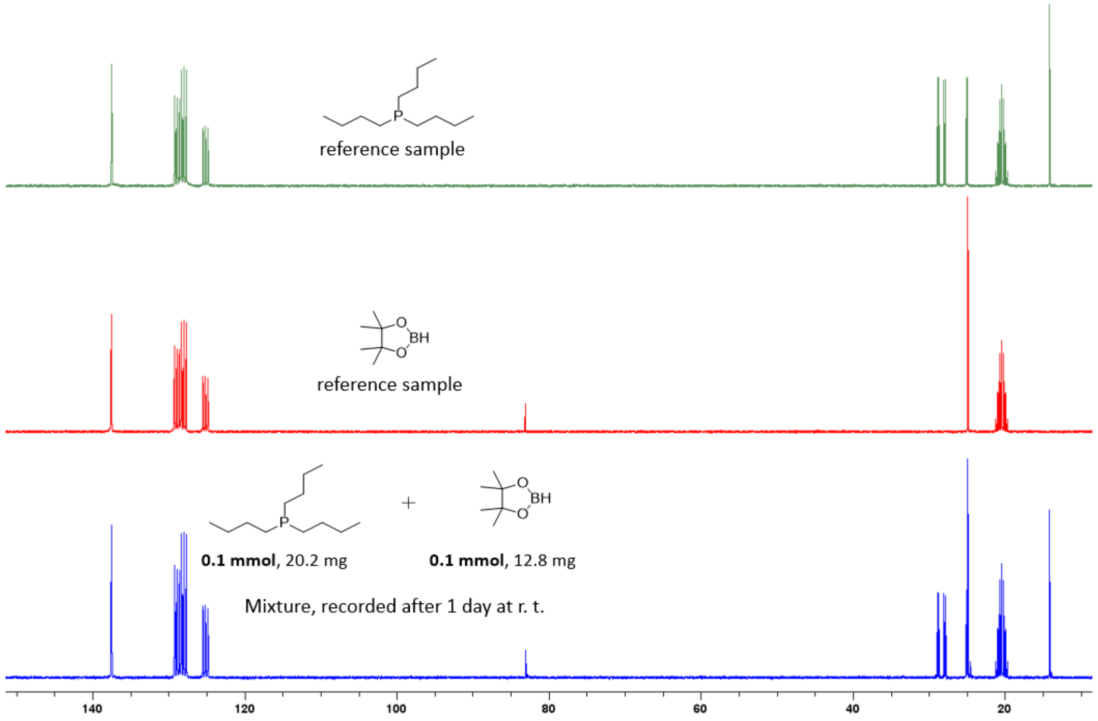

Figure 23: $^{13}C\{^1H\}$-NMR (75 MHz) of a 1:1 mixture of $PnBu_3$ and HBpin (blue) and reference samples of the components.

Diyne with Phosphine

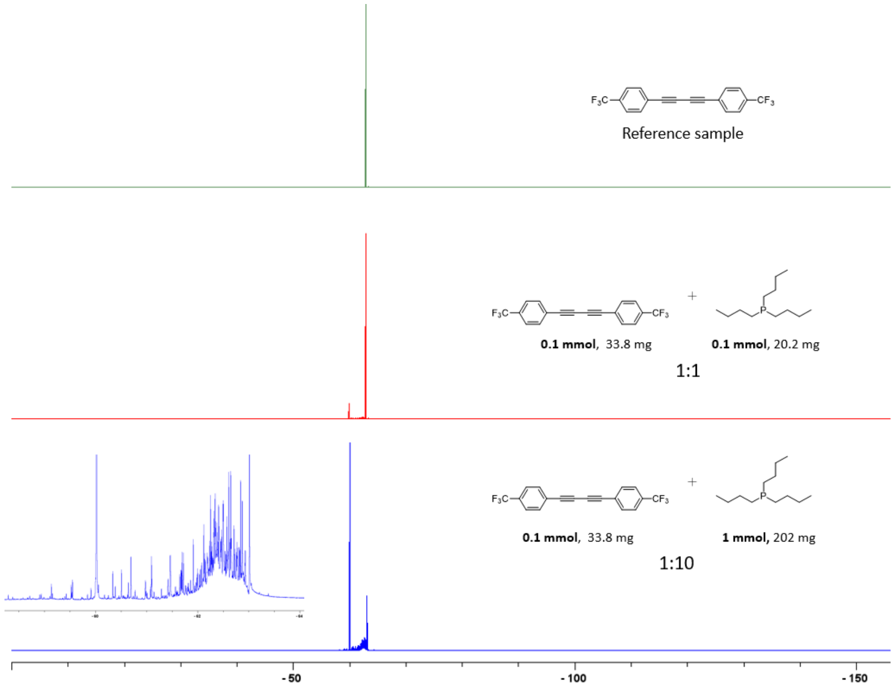

Figure 24: $^{19}F\{^1H\}$-NMR of a mixture of diyne **1q** and $PnBu_3$ after 3 d at room temperature in 1:1 ratio (red, middle spectrum) and in a 1:10 ratio (blue, bottom spectrum, 564 MHz) and a diyne **1q** reference spectrum (green, top spectrum, 470 MHz).

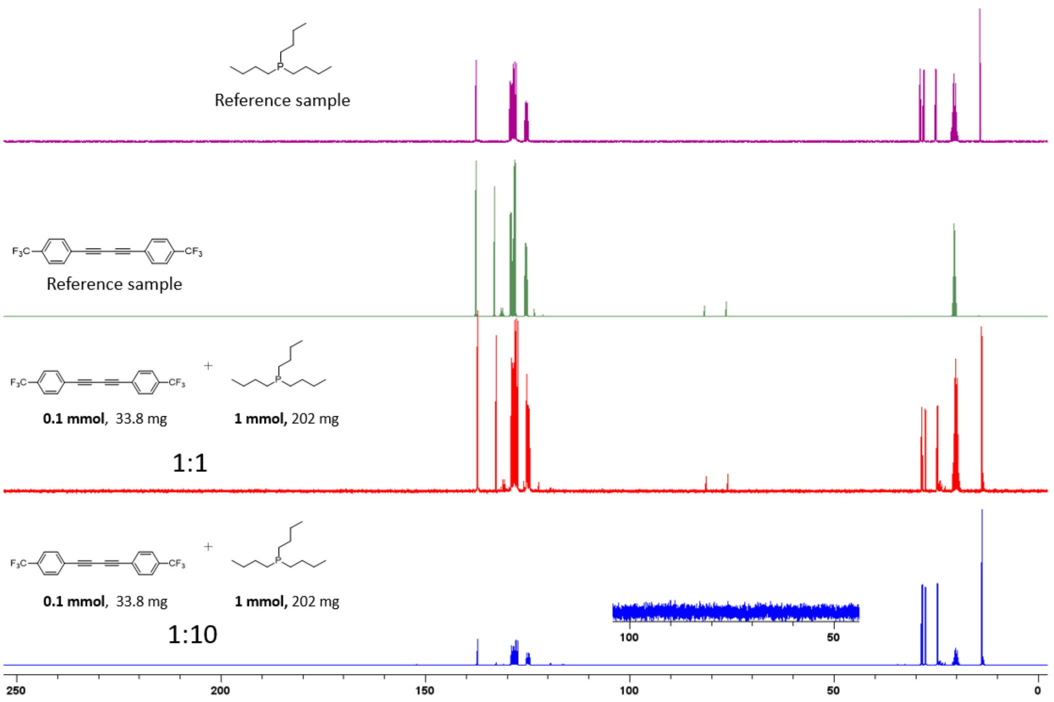

Figure 25: $^{13}C\{^{1}H\}$-NMR (75 MHz) of diyne **1q** and $PnBu_3$, after 3 d at room temperature in 1:1 ratio (red) and in a 1:10 ratio (blue) and spectra of a diyne **1q** reference sample (green) and a $PnBu_3$ reference sample (purple).

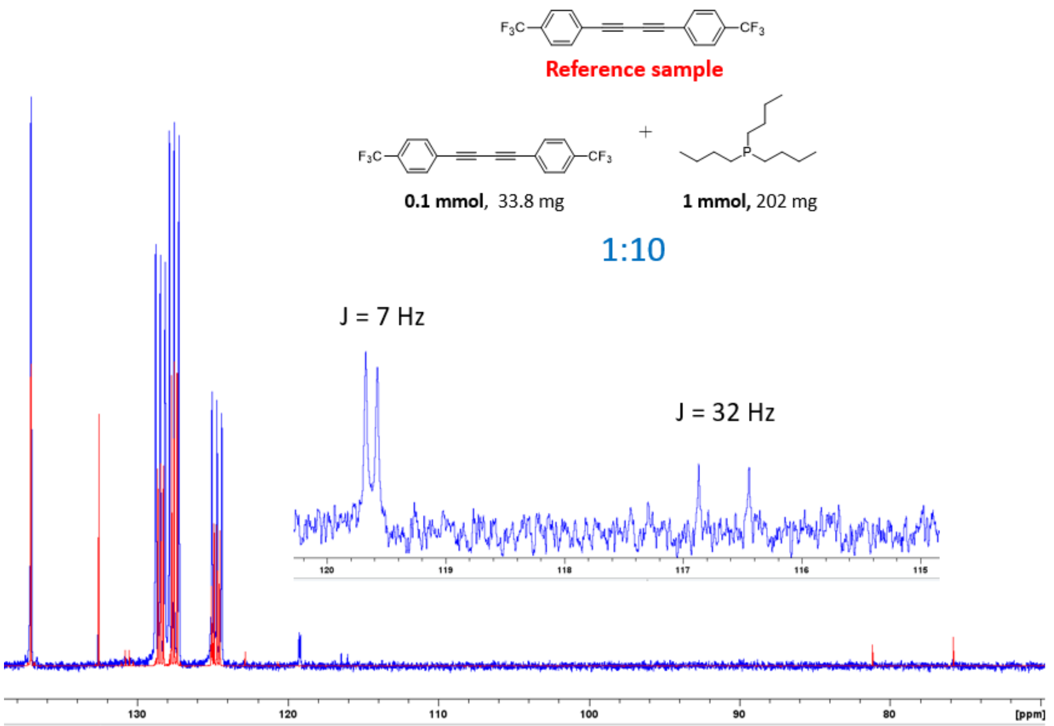

Figure 26: $^{13}C\{^{1}H\}$-NMR (75 MHz, enlarged) of diyne **1q** and $PnBu_3$, after 3 d at room temperature in a 1:10 ratio (blue) compared to a reference spectrum of diyne **1q** (red).

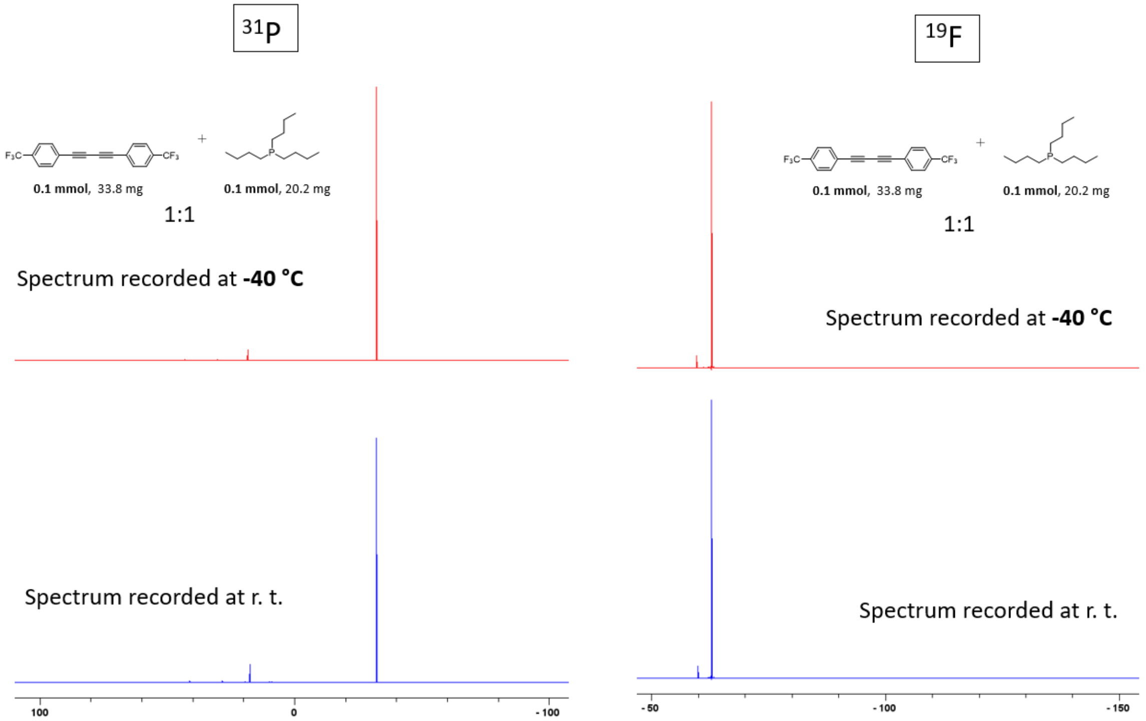

Figure 27: Comparison of $^{31}P\{^{1}H\}$ (220 MHz)- (left) and $^{19}F\{^{1}H\}$(470 MHz)- (right) NMR spectra of a 1:1 mixture of diyne **1q** and $PnBu_3$ after 3 d at room temperature. The bottom spectra were recorded at room temperature, the ones on top at -40 °C. There is no change visible when measuring the sample at lowered temperature.

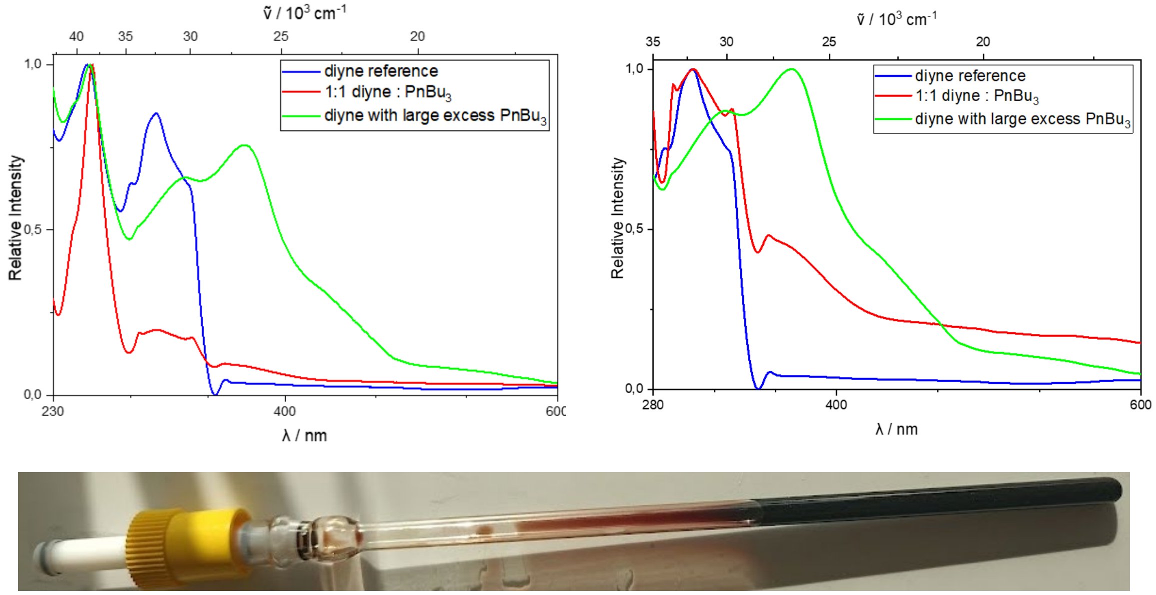

Figure 28: Above: UV/VIS-Spectra recorded after 7 d at room temperature of a reference sample of diyne **1q,** of a 1:1 mixture of diyne **1q,** and of $PnBu_3$, and of **1q** with a large excess of $PnBu_3$. Below: NMR tube of a 1:1 mixture of diyne **1q** and $PnBu_3$. The absorption of diyne **1q** in the visible region of the spectrum is potentially caused by aggregation.

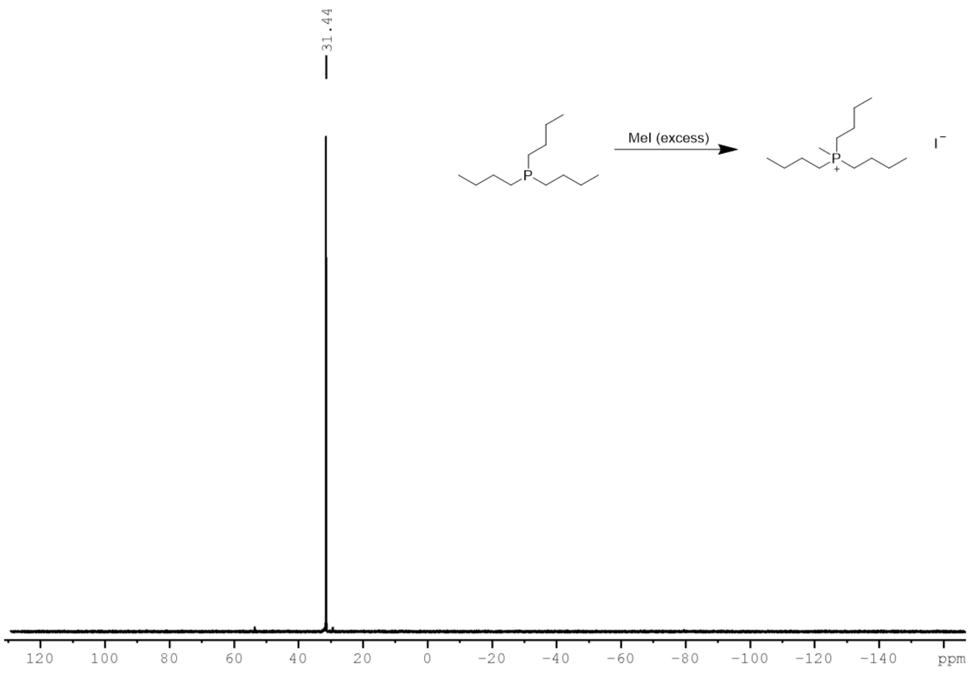

Figure 29: $^{31}P\{^{1}H\}$-NMR (121MHz) of methyl(tri-n-butyl)phosphonium iodide, used as a reference, which was obtained by the methylation of $PnBu_3$ with methyl iodide.

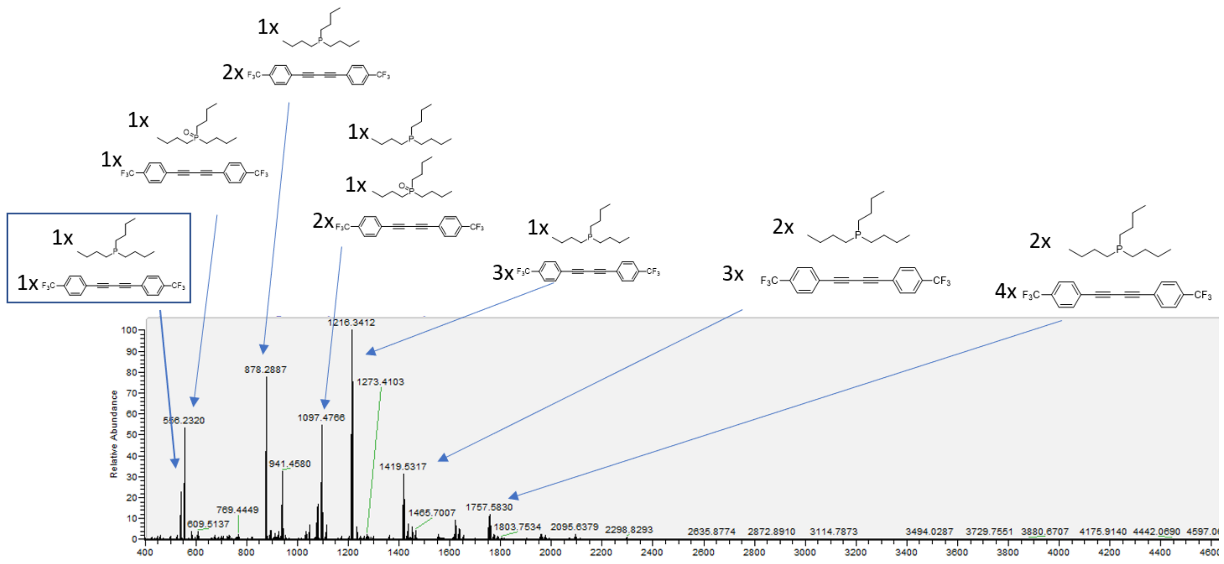

Figure 30: LIFDI-HRMS of a 1:1 mixture of $PnBu_3$ and diyne **1q**, after 3 d at room temperature. The 1:1 adduct of the substrates is marked on the left, there are several other adducts of different composition.

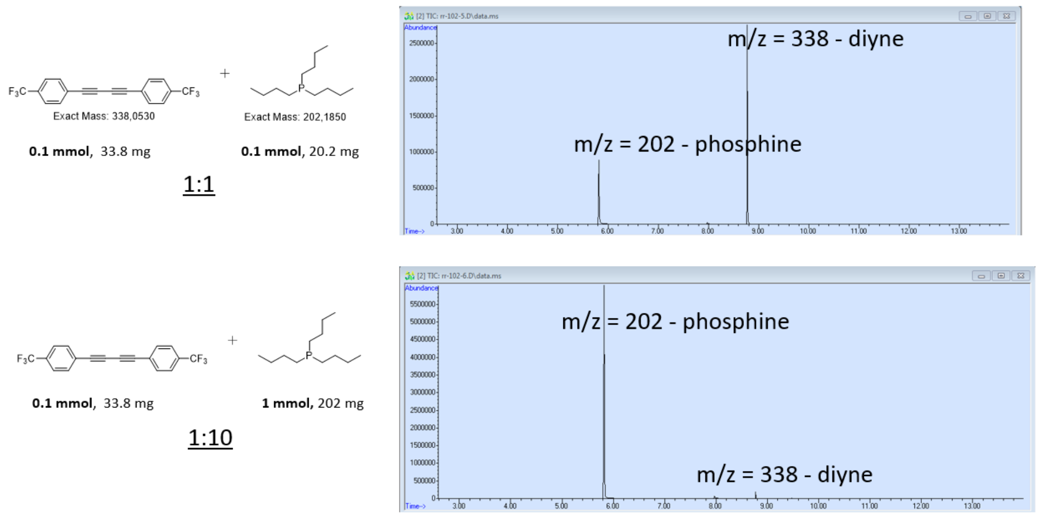

Figure 31: GCMS chromatograms of mixtures of diyne **1q** and $PnBu_3$ after 3 d at room temperature in ratios of 1:1 (top) and 1:10 (bottom). For the 1:10 ratio experiment, the diyne **1q** gets consumes almost completely. The resulting products could not be detected with the method.

Diyne with HBpin, using O=$PnBu_3$ as the catalyst

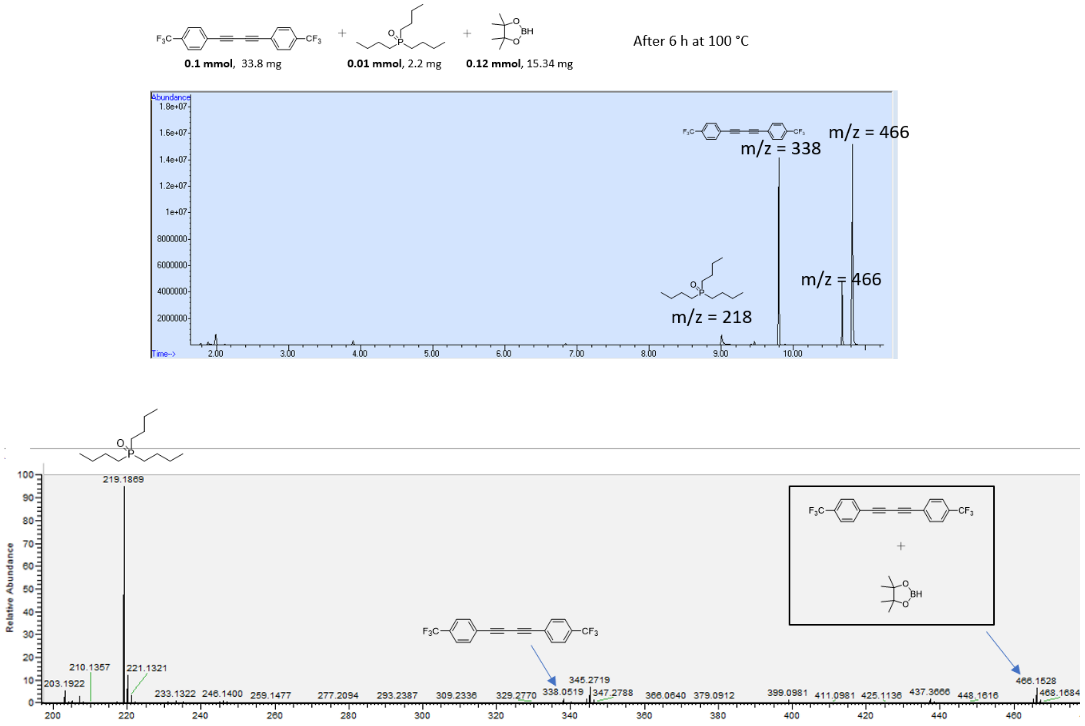

Figure 32: GCMS (above) and HRMS (below, APCI+) of a 1 : 0.1 : 1.2 mixture of diyne **1q**, O=$PnBu_3$ and HBpin after 6 h at 100 °C . The signals of the hydroboration products (m/z = 466) and of the starting material are marked.

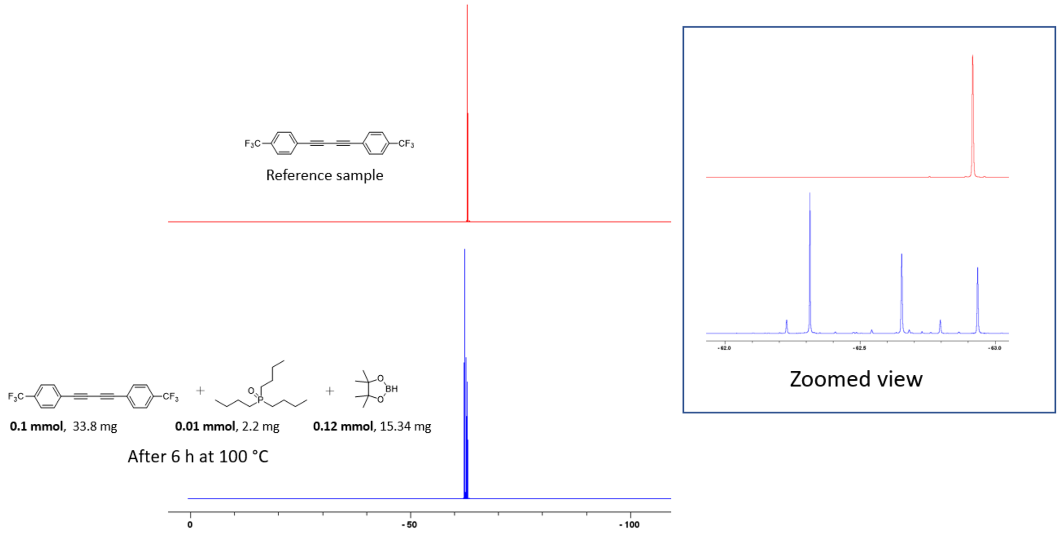

Figure 33: $^{19}F\{^1H\}$ (470 MHz blue, 564 MHz red) NMR of a mixture of diyne **1q**, HBpin and O=PnBu3 as the catalyst after 6 h at 100 °C, in comparison with a reference sample of diyne **1q**.

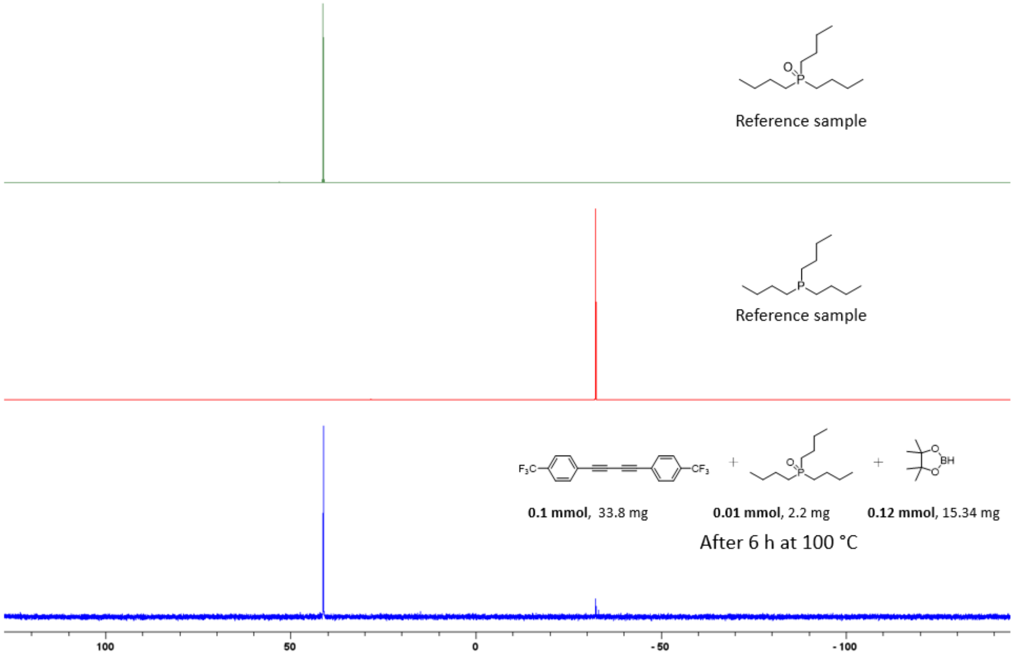

Figure 34: $^{31}P\{^1H\}$-NMR (121 MHz) of a mixture of diyne **1q**, HBpin and O=PnBu3 as the catalyst after 6 h at 100 °C. (blue), and PnBu3 (red) and O=PnBu3 (green) reference samples. A peak corresponding to PnBu3 can be found in the bottom spectrum of the reaction mixture.

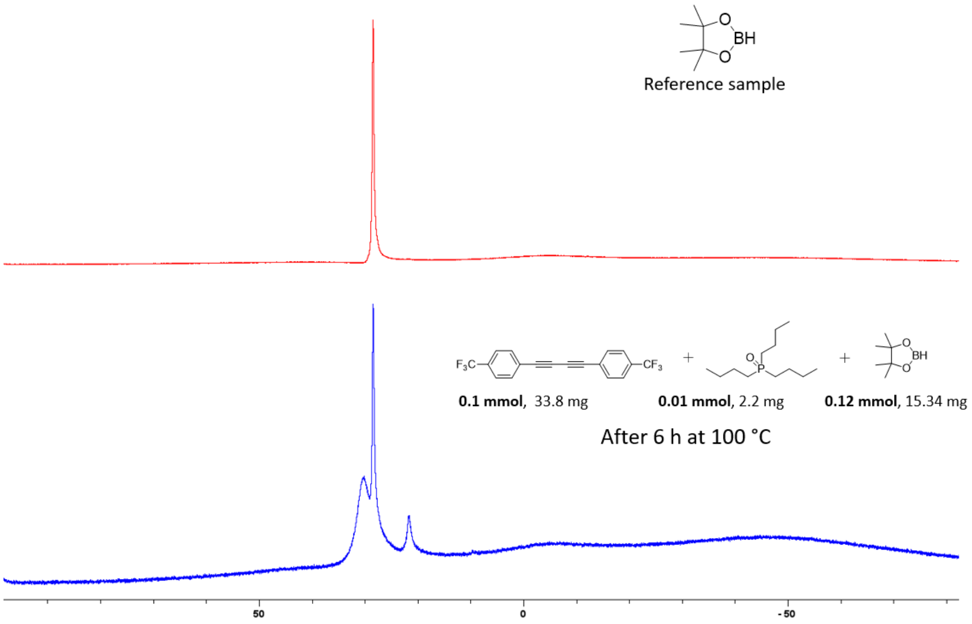

Figure 35: $^{11}B\{^{1}H\}$-NMR (121 MHz) of a mixture of diyne **1q**, HBpin and O=PnBu$_3$ as the catalyst after 6 h at 100 °C. (blue), and of a HBpin (red) reference sample. Two new signals are detected at 30.3 and 21.7 ppm in the mixture.

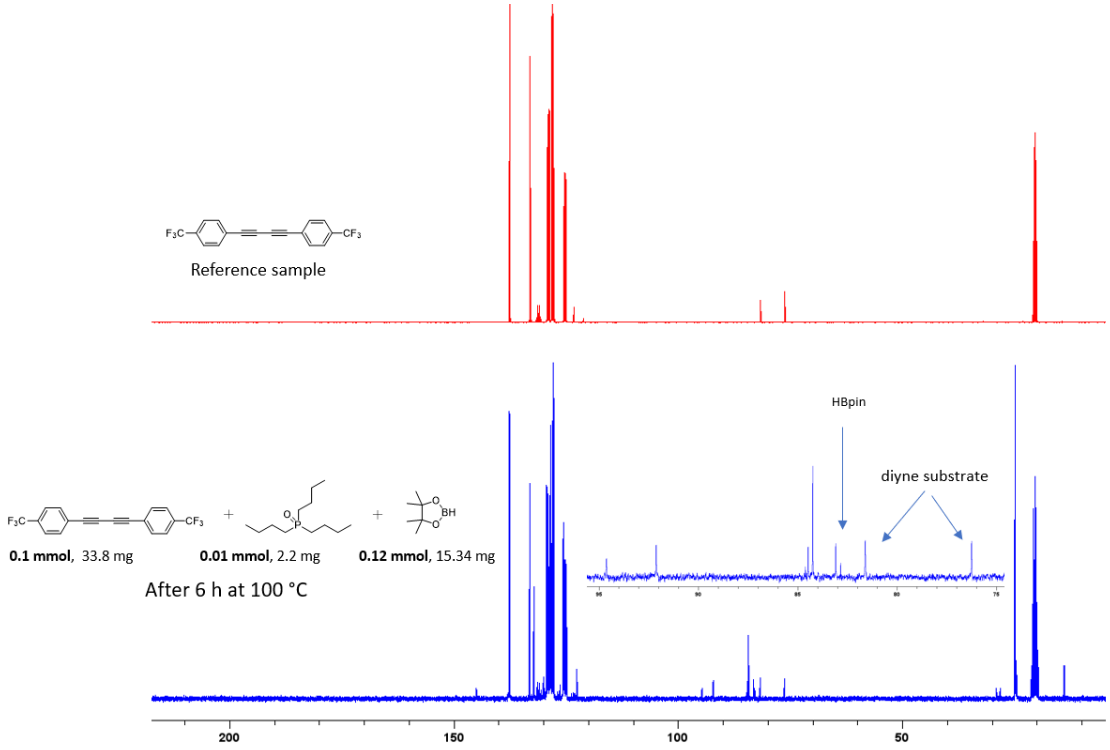

Figure 36: $^{13}C\{^{1}H\}$-NMR (75 MHz) of a mixture of diyne **1q**, HBpin and O=PnBu$_3$ after 6 h at 100 °C. (blue), and of a diyne **1q** (red) reference sample.

HBpin with O=PnBu$_3$

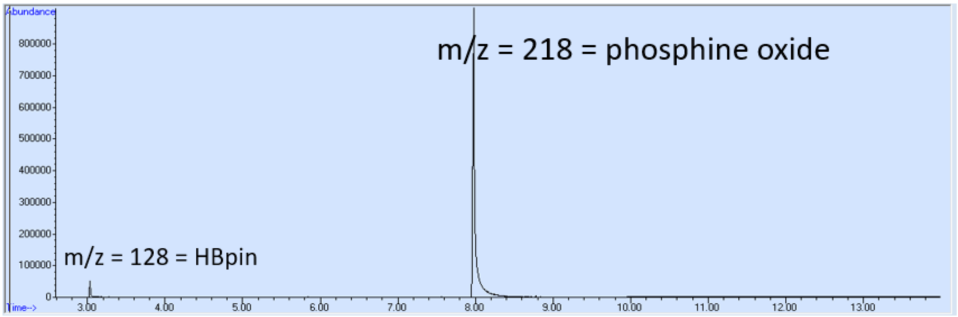

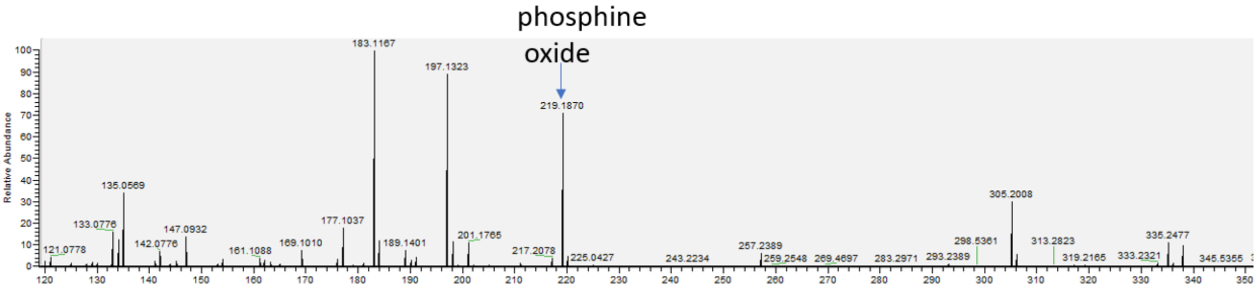

Figure 37: GCMS (above) and ESI+ HRMS (below) of a mixture of O=PnBu$_3$ and HBpin, after 1 d at room temperature. No adduct was detected.

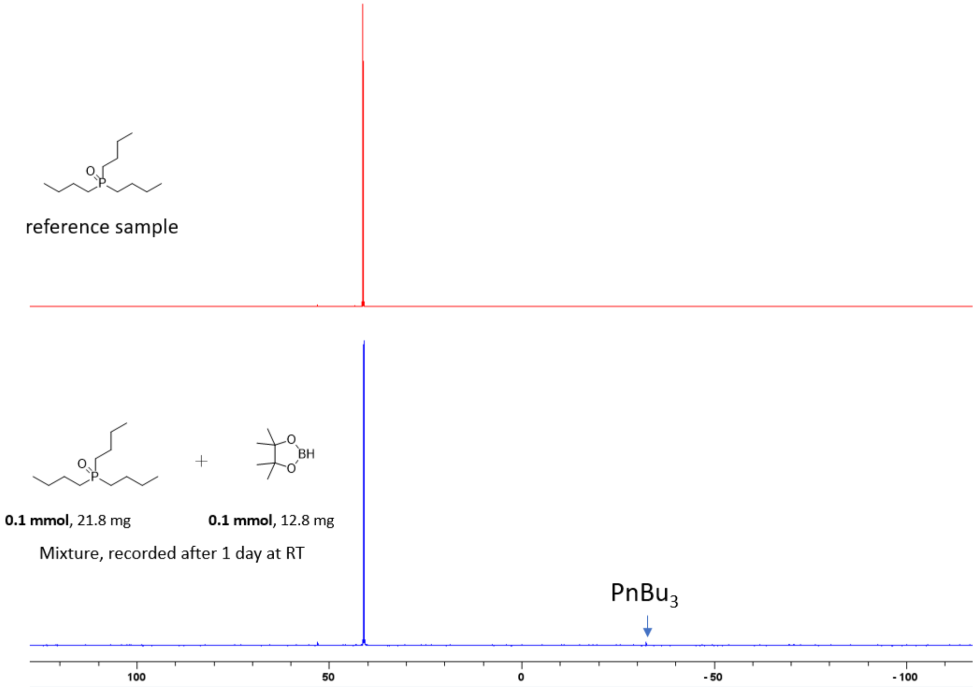

Figure 38: $^{31}P\{^{1}H\}$-NMR (121MHz) of a mixture of O=PnBu$_3$ and HBpin after 1d at r.t. (blue) and a O=PnBu$_3$ reference spectrum. The signal of the newly formed PnBu$_3$ is marked in the bottom spectrum.

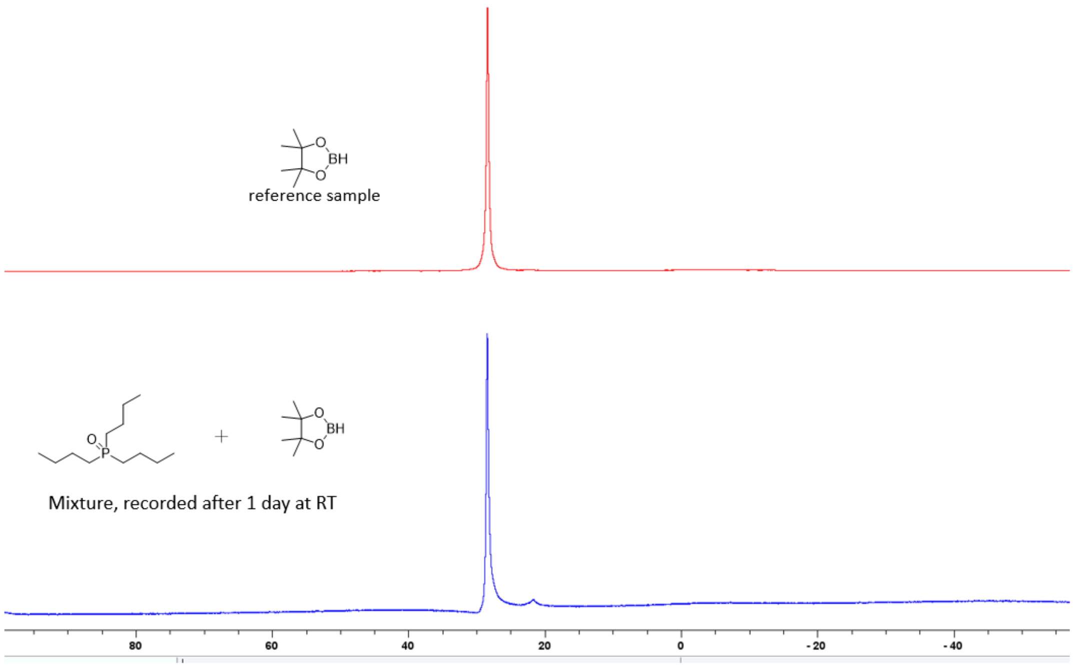

Figure 39: $^{11}B\{^1H\}$ (96 MHz) NMR spectra of a mixture of O=PnBu$_3$ (0.1 mmol) and HBpin (0.1 mmol), after 1 d at room temperature (blue) and a HBpin reference spectrum (red).

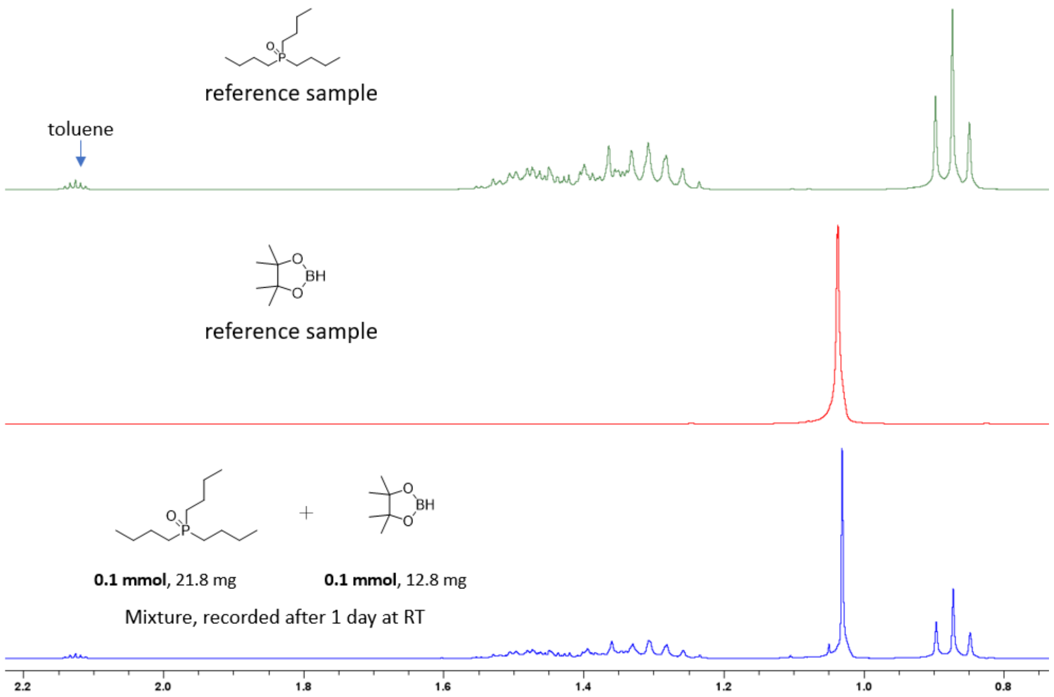

Figure 40: ^1H (300 MHz) NMR spectra of a mixture of O=PnBu$_3$ (0.1 mmol) and HBpin (0.1 mmol) after 1 d at room temperature (blue), and of a HBpin reference (red), and a O=PnBu$_3$ reference spectrum (green).

Diyne with O=PnBu$_3$

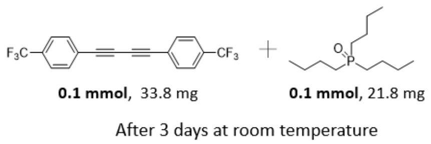

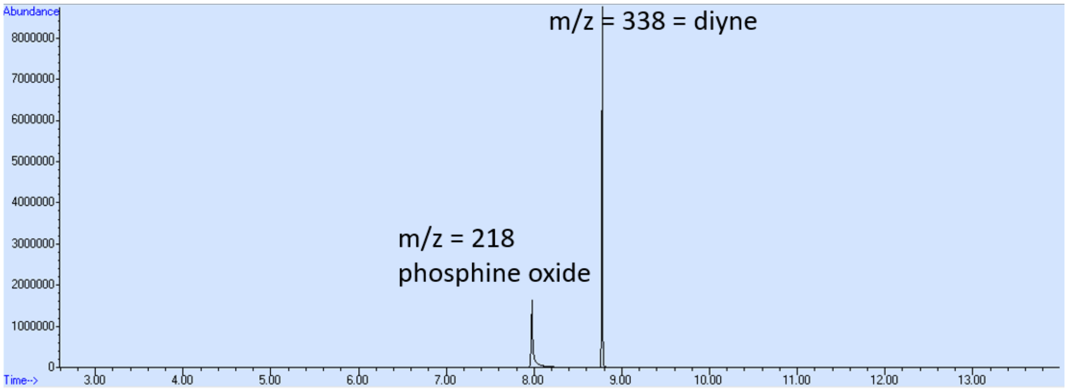

Figure 41: GCMS of a mixture of diyne **1q** and O=PnBu$_3$ (0.1 mmol) after 3 d at room temperature. Only the starting material was detected in the mixture. The mixture did not turn dark, like the mixture of PnBu$_3$ and diyne **1q** did.

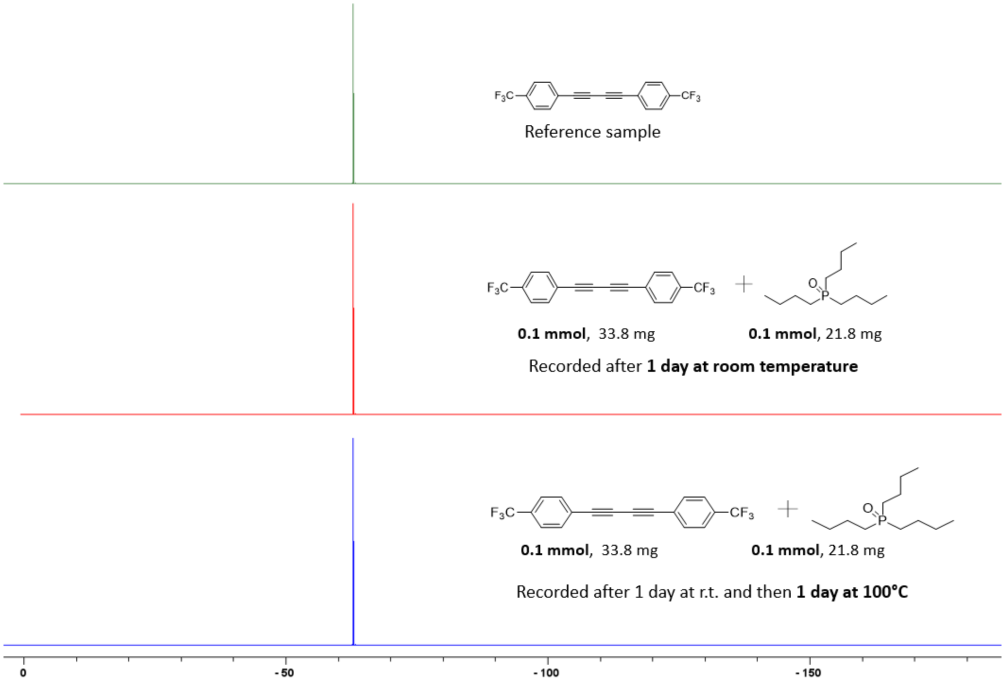

Figure 42: $^{19}F\{^{1}H\}$-NMR of mixtures of O=PnBu$_3$ (0.1 mmol) and diyne **1q** (0.1 mmol) after 1 d at room temperature (470 MHz, red) and the same sample after another 1 d at 100 °C (470 MHz, blue) and a reference spectrum of diyne **1q** (564 MHz, green). No reaction was observed.

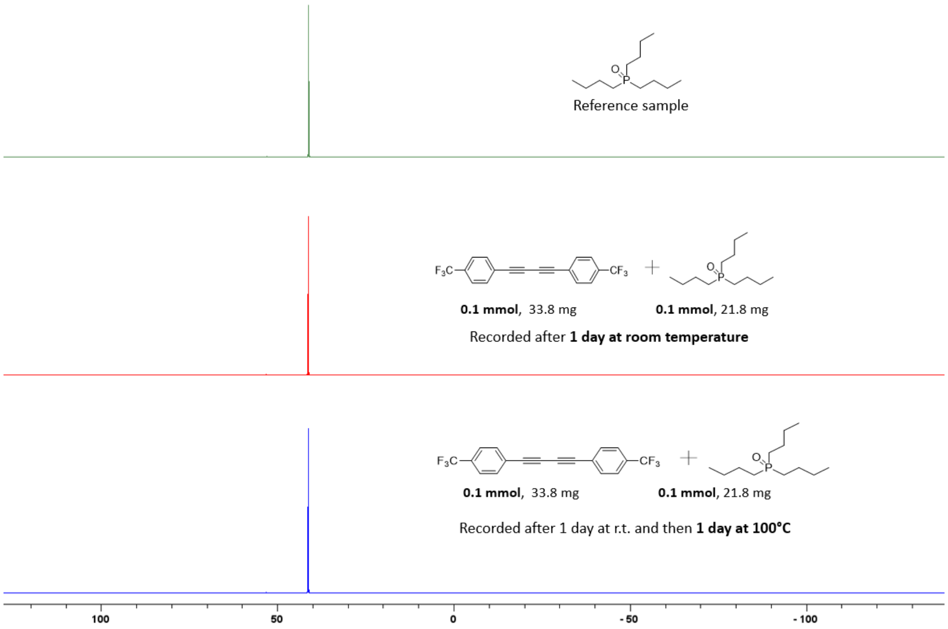

Figure 43: $^{31}P\{^{1}H\}$ (121 MHz) NMR of mixtures of O=PnBu$_3$ (0.1 mmol) and diyne **1q** (0.1 mmol) after 1 d at room temperature (red) and the same sample after another d at 100 °C (blue) and a reference spectrum of O=PnBu$_3$ (green). No reaction was observed.

VIII Computational Studies:

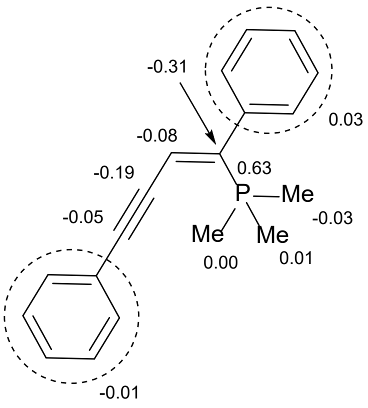

Figure SI_VIII_1: Mulliken charge analysis of intermediate **B.**

Computational Details [23-25]

All DFT calculations were conducted with the Gaussian 09 package.[1] All of the structures were optimized with the M06-2x functional[2] using the 6-31G(d,p) basis set[3] for all atoms in gas phase. For the stationary points, frequency calculations were performed at the same level of theory to ensure that the intermediates had no imaginary frequencies and transition states had only one imaginary frequency, and to obtain the relative Gibbs free energies at 298K. Transition states were further confirmed by intrinsic reaction coordinate (IRC) calculations.

Table S1 Energies of calculated structures (Hartrees)

	E(gas)	G(gas)
**PMe3**	-460.9904	-460.9060
**A**	-615.3640	-615.2018
**TSA-B**	-1076.3281	-1076.0619
**B**	-1076.3339	-1076.0661
**HBpin**	-411.7014	-411.5415
**C**	-1488.0684	-1487.6159
**TSC-D**	-1488.0514	-1487.6027
**D**	-1488.1117	-1487.6568
**TSD-E**	-1488.1072	-1487.6513
**E**	-1488.1117	-1487.6574
**TSE-F**	-1488.0954	-1487.6413
**F**	-1027.1365	-1026.7891

**TSE-F'**	-1488.0916	-1487.6368
**F'**	-1027.1393	-1026.7911
**TSD-D1**	-1488.0977	-1487.6408
**D1**	-1488.1082	-1487.6538
**TSD1-E1**	-1488.1020	-1487.6452
**E1**	-1488.1078	-1487.6511
**TSE1-F**	-1488.0852	-1487.6305
**TSE1-F'**	-1488.0869	-1487.6321
**B_1**	-1438.0273	-1437.6535
**B_2**	-1438.0359	-1437.662
**B_3**	-1438.0434	-1437.6691
**B_4**	-1438.0447	-1437.6711

IX X-ray crystallography experimental [26]

The crystals were grown slowly in 7 mL vials from DCM/hexane.

For **2g**: A colorless plate (0.03 x 0.11 x 0.19 mm^3) was centered on the goniometer of a Rigaku Oxford Diffraction Synergy-S diffractometer equipped with a HyPix6000HE detector and operating with CuKα radiation. The data collection routine, unit cell refinement, and data processing were carried out with the program CrysAlisPro.[26a] The Laue symmetry and systematic absences were consistent with the monoclinic space groups $P2/n$ and Pn. Only the noncentrosymmetric space group, Pn, gave a reasonable structure solution. In addition, $P2/n$ would have required Z' = ½ which is not reasonable for this molecule; nor does the disorder (vide infra) mimic a 2-fold parallel to [010]. The structure was solved using SHELXS and refined using SHELXL via Olex2. Olex2 was used for molecular graphics generation.

Special notes for **2g**: The structure exhibits whole-molecule disorder, with the relative occupancies of the 2 conformations refining to 0.754(4) and 0.246(4). The two orientations of the molecule have nearly identical 'footprints' with overlap in the pinacolato boron group and the two methoxy phenyl groups. To maintain a chemically reasonable structure model, especially for the minor conformation, multiple constraints and restraints were used. An AFIX 66 command was used to restrain the two phenyl groups of the minor conformation to be hexagonal. A SIMU restraint was used to keep anisotropic displacement parameters of the minor conformation from going non-positive definite. Finally, the anisotropic displacement parameters of atoms from the two conformations that were very near each other were constrained to be equal with EADP. The final refinement model involved anisotropic displacement parameters for non-hydrogen atoms and a riding model for all hydrogen atoms.

For **2j**: A colorless plate (0.04 x 0.12 x 0.30 mm^3)/ **2q**: a colorless rod (0.10 x 0.11 x 0.66 mm^3)/ **2ad**: yellow plate (0.05 x 0.20 x 0.37 mm^3) were centered on the goniometer of a Rigaku Oxford Diffraction Synergy-S diffractometer equipped with a HyPix6000HE detector and operating with CuKα radiation. The data collection routine, unit cell refinement, and data processing were carried out with the program CrysAlisPro. The Laue symmetry and systematic absences were consistent with the monoclinic space group $P2_1/c$ for **2j** / The Laue symmetry was consistent with the triclinic space groups P1 and P-1(**2q**), The

centrosymmetric space group P-1 was chosen and gave a satisfactory structure solution/ The Laue symmetry and systematic absences were consistent with the monoclinic space group $P2_1/c$ for **2ad**. The structure was solved using SHELXT[26b] and refined using SHELXL[26c] via Olex2.[26d] The final refinement model involved anisotropic displacement parameters for non-hydrogen atoms and a riding model for all hydrogen atoms. Olex2 was used for molecular graphics generation.

CCDC numbers : **2g** (CCDC 2150970), **2j** (CCDC 2150972), **2q** (CCDC 2150971), **2ad** (CCDC 2150973)

Single-crystal X-ray diffraction data and structure refinements.

Identification code	2g	2j	2q	2ad
CCDC #	2150970	2150972	2150971	2150973
Empirical formula	$C_{24}H_{27}BO_4$	$C_{26}H_{31}BO_4$	$C_{24}H_{21}BF_6O_2$	$C_{26}H_{30}BNO_4$
Formula weight	390.26	418.32	466.22	431.32
Temperature/K	100.00(12)	100.00(10)	100.00(12)	99.99(13)
Crystal system	monoclinic	monoclinic	triclinic	monoclinic
Space group	*Pn*	$P2_1/c$	P-1	$P2_1/c$
a/Å	7.98308(10)	18.5356(2)	6.87760(10)	13.92470(10)
b/Å	12.49107(14)	9.97500(10)	11.5850(2)	11.09910(10)
c/Å	10.87444(10)	13.48040(10)	14.9735(3)	16.0593(2)
α/°	90	90	110.376(2)	90
β/°	99.8032(11)	104.1810(10)	91.714(2)	110.1060(10)
γ/°	90	90	104.877(2)	90
Volume/Å^3	1068.54(2)	2416.47(4)	1071.49(4)	2330.73(4)
Z	2	4	2	4
ρ_{calc}g/cm^3	1.213	1.150	1.445	1.229
μ/mm^{-1}	0.643	0.598	1.084	0.650
F(000)	416.0	896.0	480.0	920.0
Crystal size/mm^3	0.19 × 0.11 × 0.03	0.3 × 0.12 × 0.04	0.66 × 0.11 × 0.1	0.37 × 0.205 × 0.05
Radiation	Cu Kα (λ = 1.54184)	Cu Kα (λ = 1.54184)	Cu Kα (λ = 1.54184)	Cu Kα (λ = 1.54184)
2Θ range for data collection/°	7.076 to 154.57	4.918 to 154.764	6.352 to 154.89	6.76 to 154.462
Index ranges	$-10 \le h \le 10$, $-15 \le k \le 15$, $-13 \le l \le 13$	$-22 \le h \le 23$, $-12 \le k \le 12$, $-17 \le l \le 16$	$-7 \le h \le 8$, $-14 \le k \le 14$, $-18 \le l \le 18$	$-17 \le h \le 17$, $-13 \le k \le 14$, $-20 \le l \le 19$
Reflections collected	22548	43887	27038	38240
Independent reflections	4186 [R_{int} = 0.0379, R_σ = 0.0279]	5138 [R_{int} = 0.0481, R_σ = 0.0291]	4503 [R_{int} = 0.0482, R_σ = 0.0326]	4930 [R_{int} = 0.0375, R_σ = 0.0255]
Data/restraints/parameters	4186/354/384	5138/0/286	4503/0/302	4930/0/296
Goodness-of-fit on F^2	1.114	1.052	1.028	1.047
Final R indexes [I>=2σ (I)]	R_1 = 0.0406, wR_2 = 0.0974	R_1 = 0.0377, wR_2 = 0.0967	R_1 = 0.0394, wR_2 = 0.1067	R_1 = 0.0404, wR_2 = 0.1100
Final R indexes [all data]	R_1 = 0.0412, wR_2 = 0.0977	R_1 = 0.0408, wR_2 = 0.0990	R_1 = 0.0416, wR_2 = 0.1086	R_1 = 0.0430, wR_2 = 0.1121
Largest diff. peak/hole / e Å^{-3}	0.13/-0.17	0.19/-0.23	0.26/-0.29	0.33/-0.21
Flack parameter	-0.02(8)			

X References

[1] T.-P. Cheng, B.-S. Liao, Y.-H. Liu, S.-M. Peng, S.-T. Liu, *Dalton Trans.* **2012**, *41*, 3468-3473.

[2] Y. Liu, C. Wang, X. Wang, J.-P. Wan, *Tetrahedron Lett.* **2013**, *54*, 3953-3955.

[3] J. Xue, M.-T. Luo, Y.-L. Wen, M. Ye, L.-X. Liu, Z.-W. Chen, *Synthesis* **2014**, *46*, 3191-3198.

[4] P. Wang, X. Liu, S. Zhang, *Chin. J. Chem.* **2013**, *31*, 187-194.

[5] S. Parshamoni, S. Sanda, H. S. Jena, S. Konar, *Dalton Trans.* **2014**, *43*, 7191-7199.

[6] L. Feng, T. Hu, S. Zhang, H.-Y. Xiong, G. Zhang, *Org. Lett.* **2019**, *21*, 9487-9492.

[7] D.-X. Liu, F.-L. Li, H.-X. Li, W.-J. Gong, J. Gao, J.-P. Lang, *Eur. J. Org. Chem.* **2014**, *2014*, 4817-4822.

[8] Y. Arakawa, S. Nakajima, R. Ishige, M. Uchimura, S. Kang, G.-i. Konishi, J. Watanabe, *J. Mater. Chem.* **2012**, *22*, 8394-8398.

[9] S. Kaur, A. Mukhopadhyaya, A. Selim, V. Gowri, K. M. Neethu, A. H. Dar, S. Sartaliya, M. E. Ali, G. Jayamurugan, *Chem. Comm.* **2020**, *56*, 2582-2585.

[10] A. S. Batsanov, J. C. Collings, I. J. S. Fairlamb, J. P. Holland, J. A. K. Howard, Z. Lin, T. B. Marder, A. C. Parsons, R. M. Ward, J. Zhu, *J. Org. Chem.* **2005**, *70*, 703-706.

[11] X. Chen, H. Zhang, J. Chen, H. Gong, *Chem. Lett.* **2014**, *44*, 129-131.

[12] X. Li, X. Liu, H. Chen, W. Wu, C. Qi, H. Jiang, *Angew. Chem. Int. Ed.* **2014**, *53*, 14485-14489; *Angew. Chem.* **2014**, *126*, 14713 –14717.

[13] S. Li, X. Chen, J. Chen, H. Gong, *Bull. Chem. Soc. Jpn.* **2016**, *89*, 794-797.

[14] H. Amini, Ž. Ban, M. Ferger, S. Lorenzen, F. Rauch, A. Friedrich, I. Crnolatac, A. Kenđel, S. Miljanić, I. Piantanida, T. B. Marder, *Chem. Eur. J.* **2020**, *26*, 6017-6028.

[15] D. Li, Y. E. Kim, J. Yun, *Org. Lett.* **2015**, *17*, 860-863.

[16] T. Sokolnicki, J. Szyling, A. Franczyk, J. Walkowiak, *Adv. Syn. Catal.* **2020**, *362*, 177-183.

[17] B. Sundararaju, A. Fürstner, *Angew. Chem. Int. Ed.* **2013**, *52*, 14050-14054; *Angew. Chem.* **2013**,125, 14300 –1430.

[18] S. Ventre, E. Derat, M. Amatore, C. Aubert, M. Petit, *Adv. Syn. Catal.* **2013**, *355*, 2584-2590.

[19] R. A. Batey, T. D. Quach, *Tetrahedron Lett.* **2001**, *42*, 9099-9103.

[20] K. M. Junichiro Yamamoto, Tomohiro Era, Yoshisuke Nakasto, Kenji Uchida, *US2011112299 (A1)* **2011**.

[21] K. Li, S.-H. Yu, K.-F. Zhuo, X. Lu, B. Xiao, T.-J. Gong, Y. Fu, *Adv. Syn. Catal.* **2019**, *361*, 3937-3942.

[22] J. Q. Sebastian M. Weber, Gerhard Hilt, *Chem. Eur. J.* **2020**, *26*, 12129-12133.

[23] Frisch, M. J.; Trucks, G. W.; Schlegel, H. B.; Scuseria, G. E.; Robb, M. A.; Cheeseman, J. R.; Scalmani, G.; Barone, V.; Mennucci, B.; Petersson, G. A.; Nakatsuji, H.; Caricato, M.; Li, X.; Hratchian, H. P.; Izmaylov, A. F.; Bloino, J.; Zheng, G.; Sonnenberg, J. L.; Hada, M.; Ehara, M.; Toyota, K.; Fukuda, R.; Hasegawa, J.; Ishida, M.; Nakajima, T.; Honda, Y.; Kitao, O.; Nakai, H.; Vreven, T.; Montgomery, J. A., Jr.; Peralta, J. E.; Ogliaro, F.; Bearpark, M.; Heyd, J. J.; Brothers, E.; Kudin, K. N.; Staroverov, V. N.; Kobayashi, R.; Normand, J.; Raghavachari, K.; Rendell, A.; Burant, J. C.; Iyengar, S. S.; Tomasi, J.; Cossi, M.; Rega, N.; Millam, J. M.; Klene, M.; Knox, J. E.; Cross, J. B.; Bakken, V.; Adamo, C.; Jaramillo, J.; Gomperts, R.; Stratmann, R. E.; Yazyev, O.; Austin, A. J.; Cammi, R.; Pomelli, C.; Ochterski, J. W.; Martin, R. L.; Morokuma, K.; Zakrzewski, V. G.; Voth, G. A.; Salvador, P.; Dannenberg, J. J.; Dapprich, S.; Daniels, A. D.; Farkas, Ö.; Foresman, J. B.; Ortiz, J. V.; Cioslowski, J.; Fox, D. J., Gaussian09, Revision D.01. Gaussian, Inc.: Wallingford, CT, **2009**.

[24] Zhao, Y.; Truhlar, D. G. *Theor. Chem. Acc.* **2008**, *120*, 215—241.

[25] a) Ditchfield, R.; Hehre, W. J.; Pople, J. A. *J. Chem. Phys.* **1971**, *54*, 724-728; b) Hehre, W. J.; Ditchfield, R.; Pople, J. A. *J. Chem. Phys.* **1972**, *56*, 2257-2261; c) Hariharan, P. C.; Pople, J. A. *Theor. Chem. Acc.* **1973**, *28*, 213-222; d) Hariharan, P.C.; Pople, J.A. *Mol. Phys.* **1974**, *27*, 209-214.

[26] a) CrysAlisPro Software System, v1.171.41.105a, Rigaku Oxford Diffraction, **2021**, Rigaku Corporation, Oxford, UK; b) Sheldrick, G. M. *Acta Cryst*. **2015**, *A71*, 3–8; c) Sheldrick, G. M. *Acta Cryst.* **2015**, *C71*, 3-8; d) Dolomanov, O.V.; Bourhis, L. J.; Gildea, R. J.; Howard, J. A. K.; Puschmann, H. *J. Appl. Cryst.* **2009**, ***42***, 339–341; e) Olomanov, O.V.; Bourhis, L. J.; Gildea, R. J.; Howard, J. A. K.; Puschmann, H. *J. Appl. Cryst.* **2009**, ***42***, 339–341; f) DMacrae, C. F.; Sovago, I.; Cottrell, S. J.; Galek, P. T. A.; McCabe, P.; Pidcock, E.; Platings, M.; Shields, G. P.; Stevens, J. S.; Towler M.; Wood, P. A. *J. Appl. Cryst.* **2020**, *53*, 226-235.

X NMR spectra

2a-^{1}H

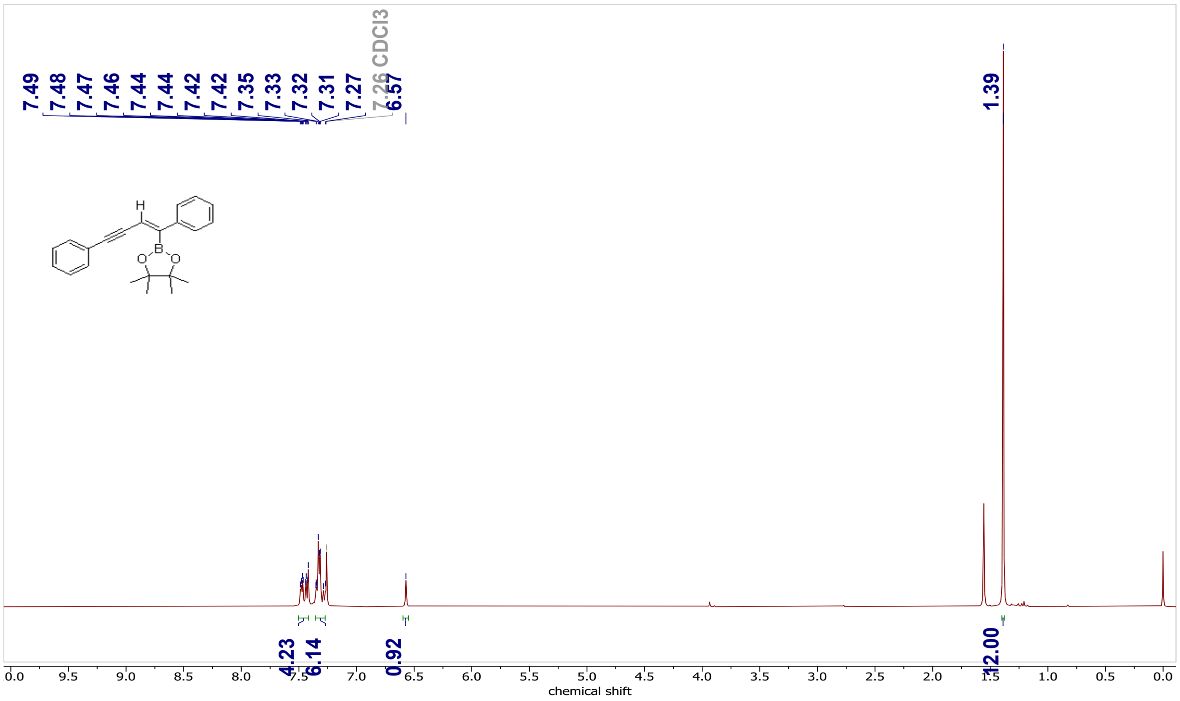

2a-^{13}C

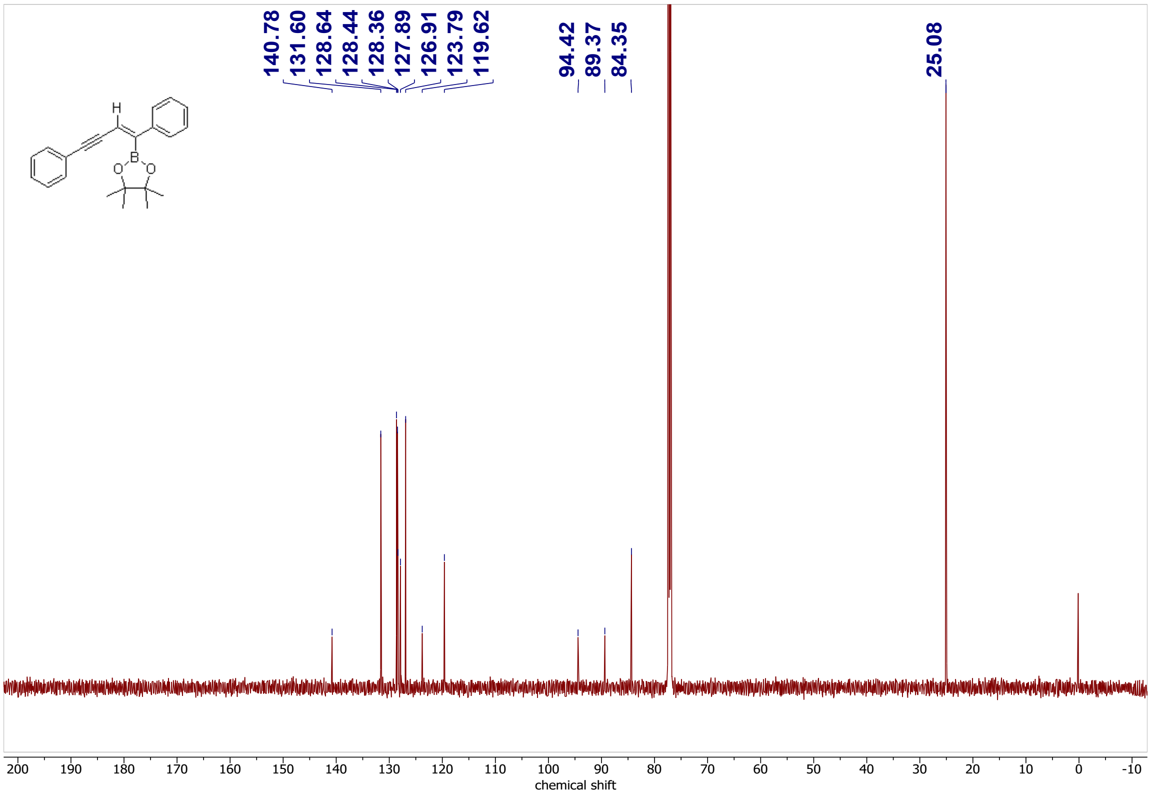

2a-^{11}B

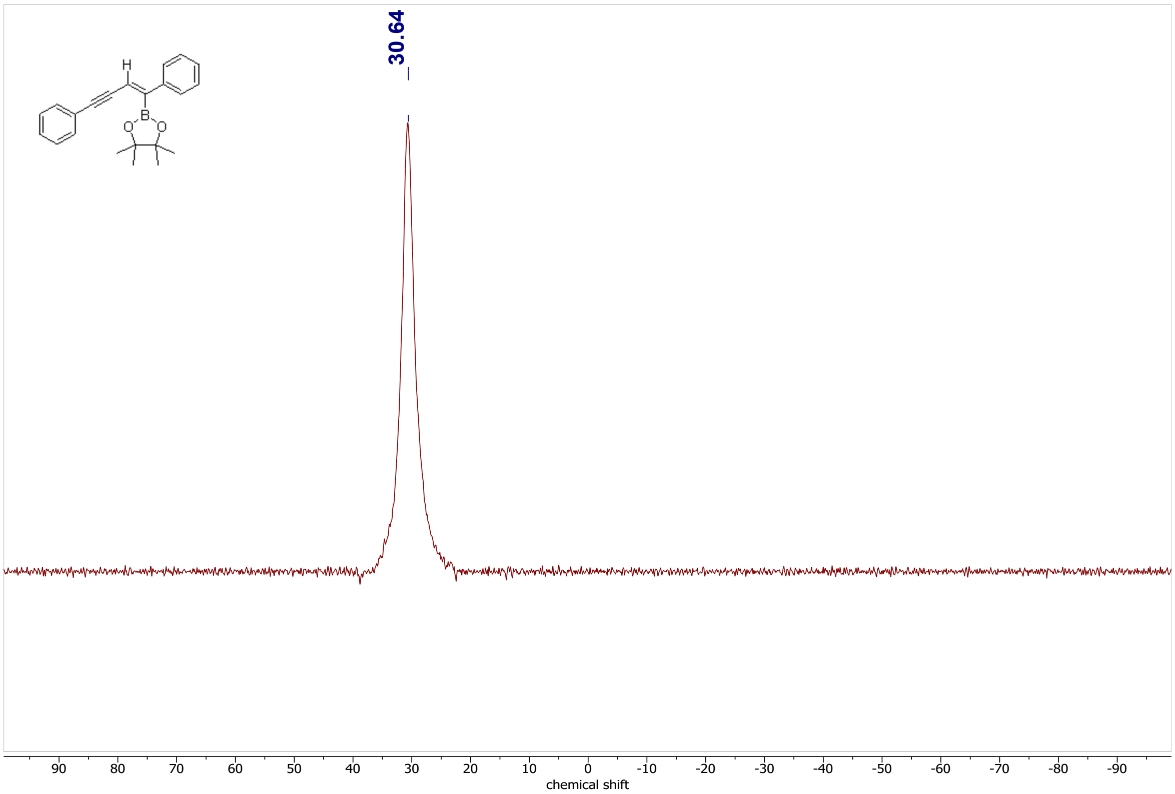

30.64
chemical shift

2b-^{1}H

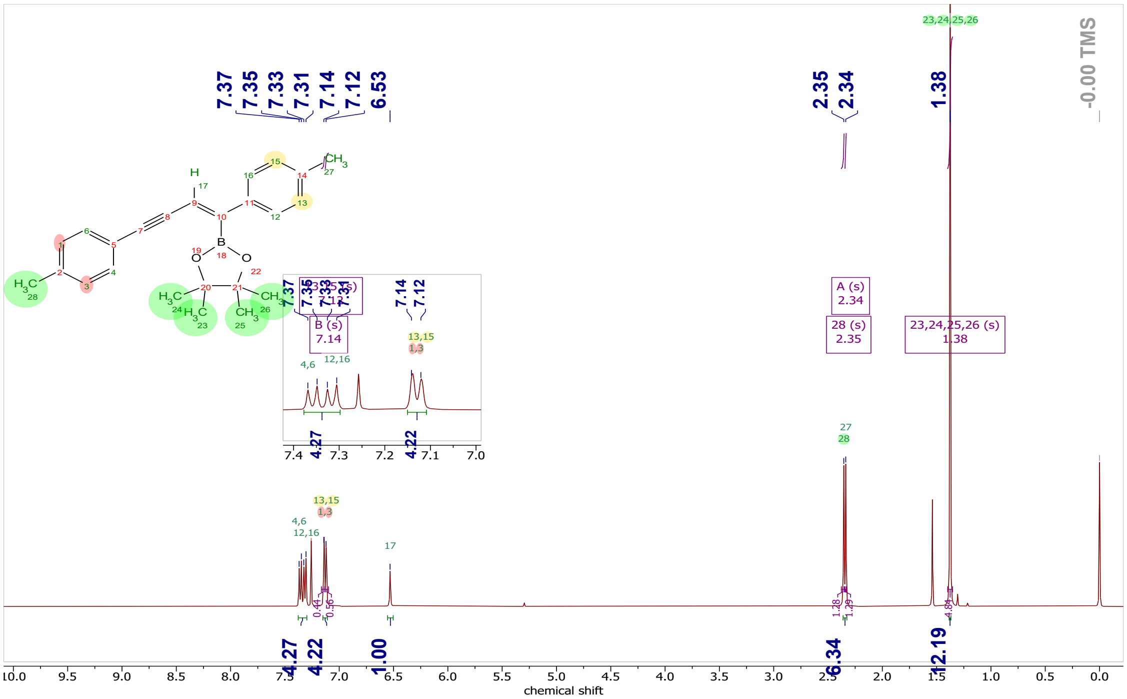

7.37 7.35 7.33 7.31 7.14 7.12 6.53
2.35 2.34
1.38
-0.00 TMS
A (s)
2.34
B (s)
7.14
28 (s)
2.35
23,24,25,26 (s)
1.38
4.27
4.22
1.00
6.34
12.19
chemical shift

2b-^{13}C

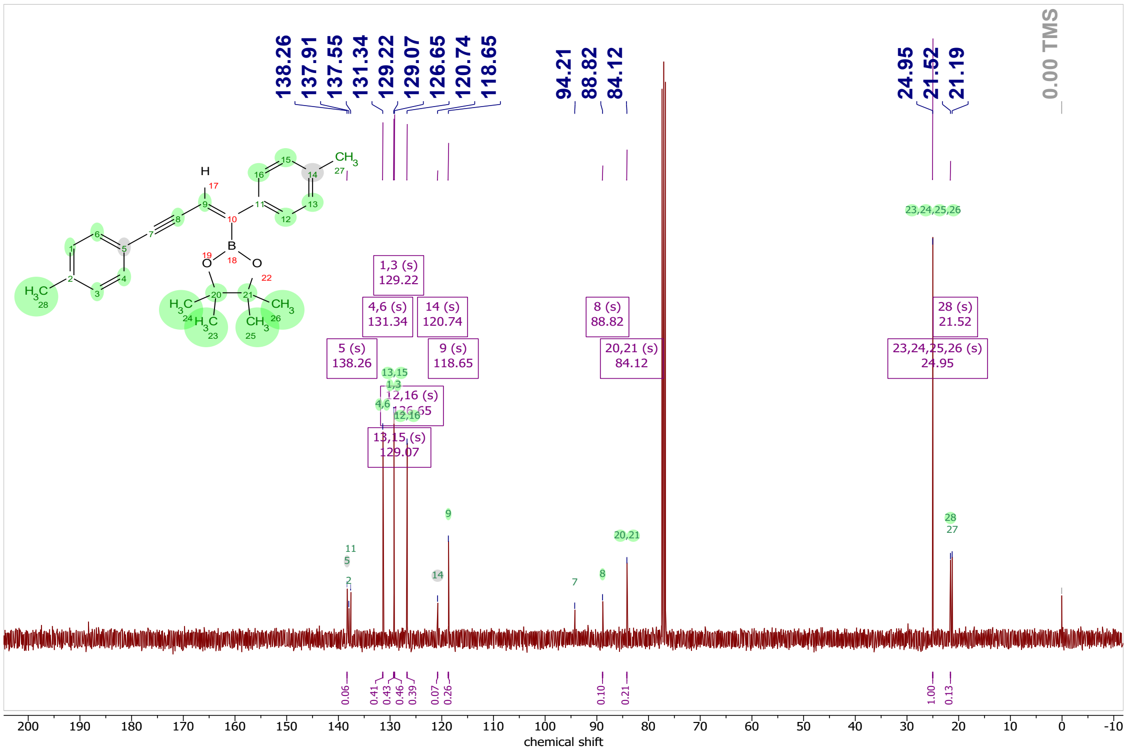
138.26
137.91
137.55
131.34
129.22
129.07
126.65
120.74
118.65
94.21
88.82
84.12
24.95
21.52
21.19
0.00 TMS
1,3 (s)
129.22
4,6 (s)
131.34
14 (s)
120.74
5 (s)
138.26
9 (s)
118.65
12,16 (s)
126.65
13,15 (s)
129.07
8 (s)
88.82
20,21 (s)
84.12
28 (s)
21.52
23,24,25,26 (s)
24.95
chemical shift

2b-^{11}B

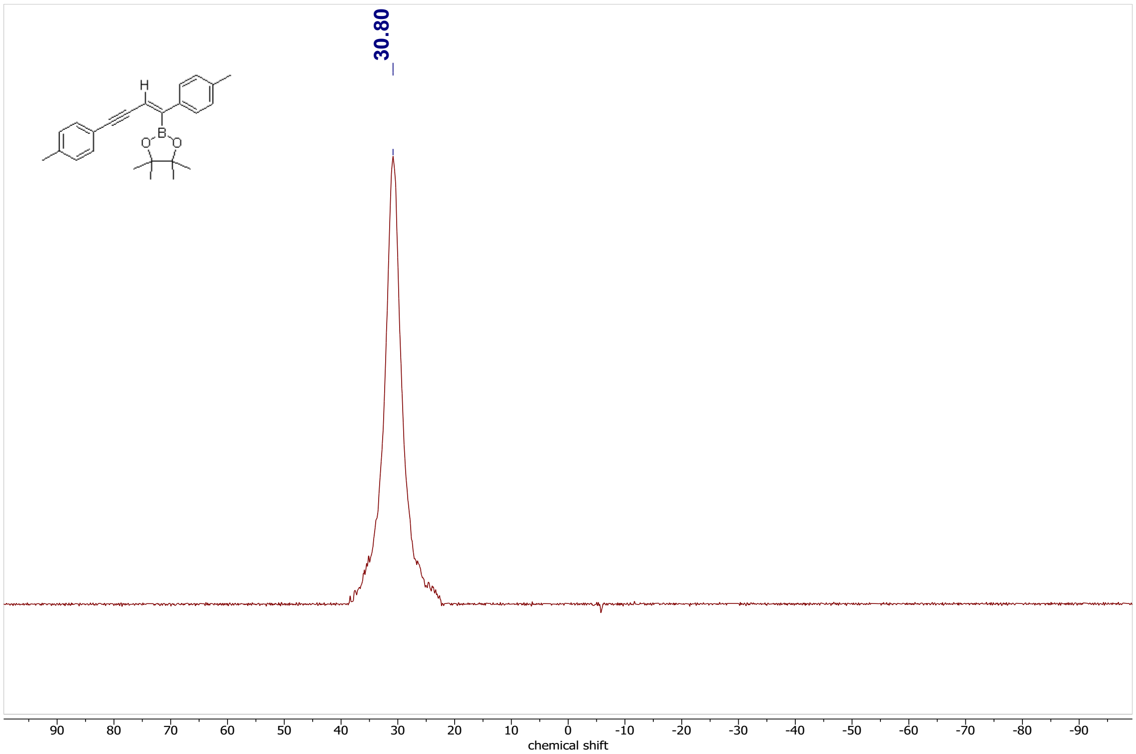
30.80
chemical shift

2b-HSQC

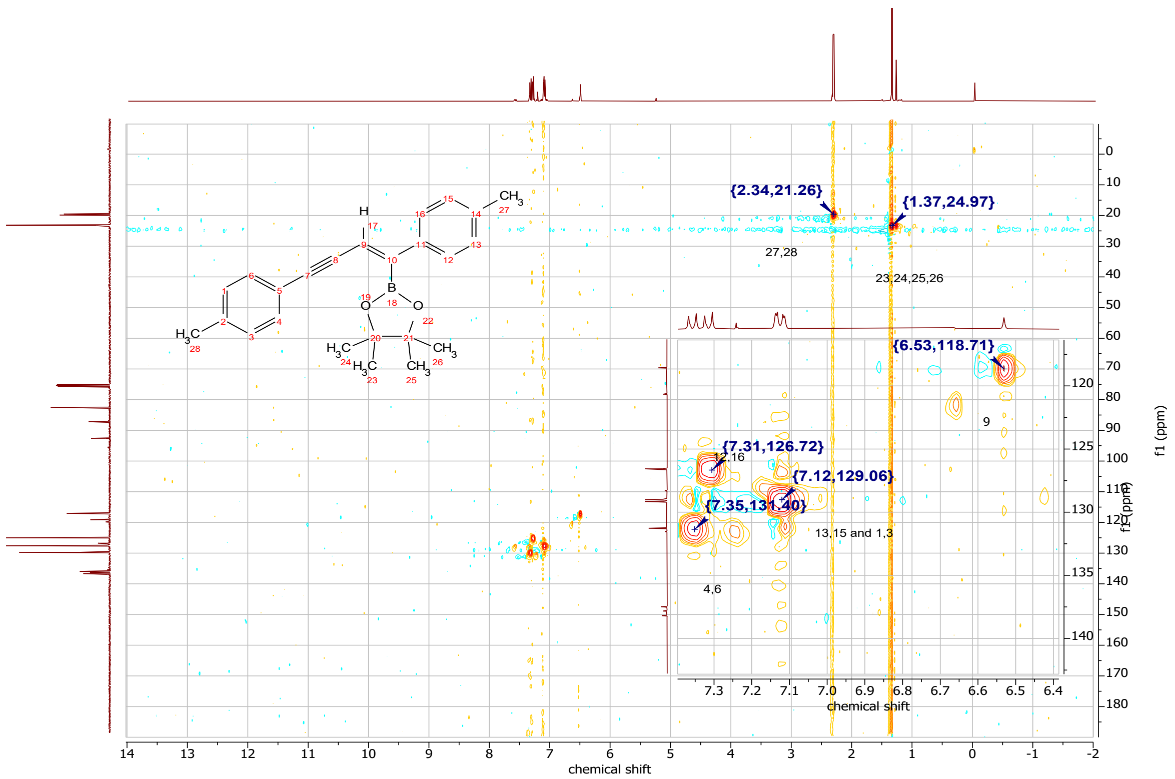

2b-HMBC

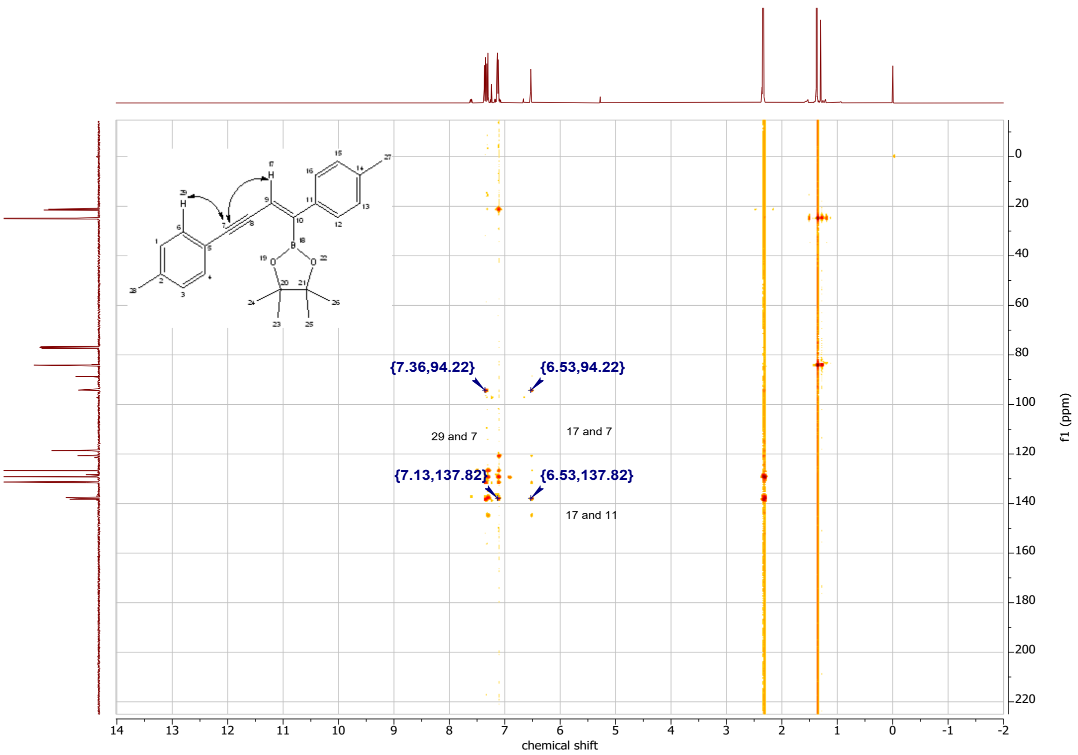

2b' -1H

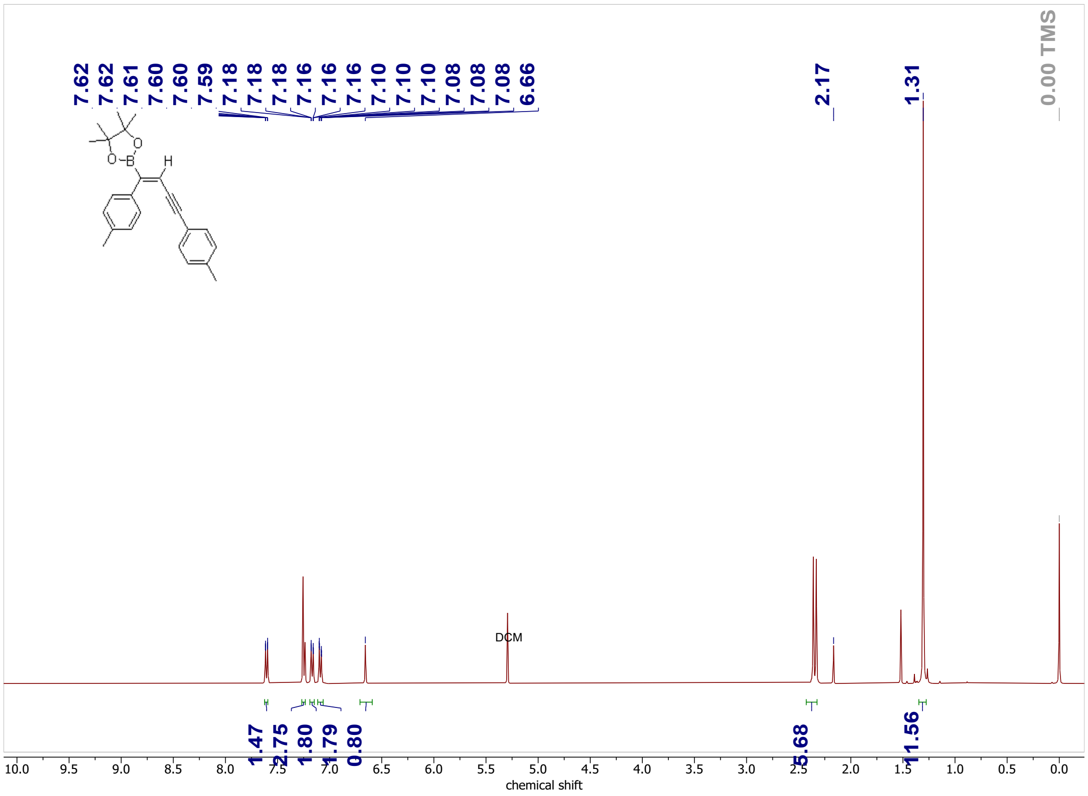

7.62
7.62
7.61
7.60
7.60
7.59
7.18
7.18
7.18
7.16
7.16
7.16
7.10
7.10
7.10
7.08
7.08
7.08
6.66
2.17
1.31
0.00 TMS
DCM
1.47
2.75
1.80
1.79
0.80
5.68
11.56
chemical shift

2c-1H

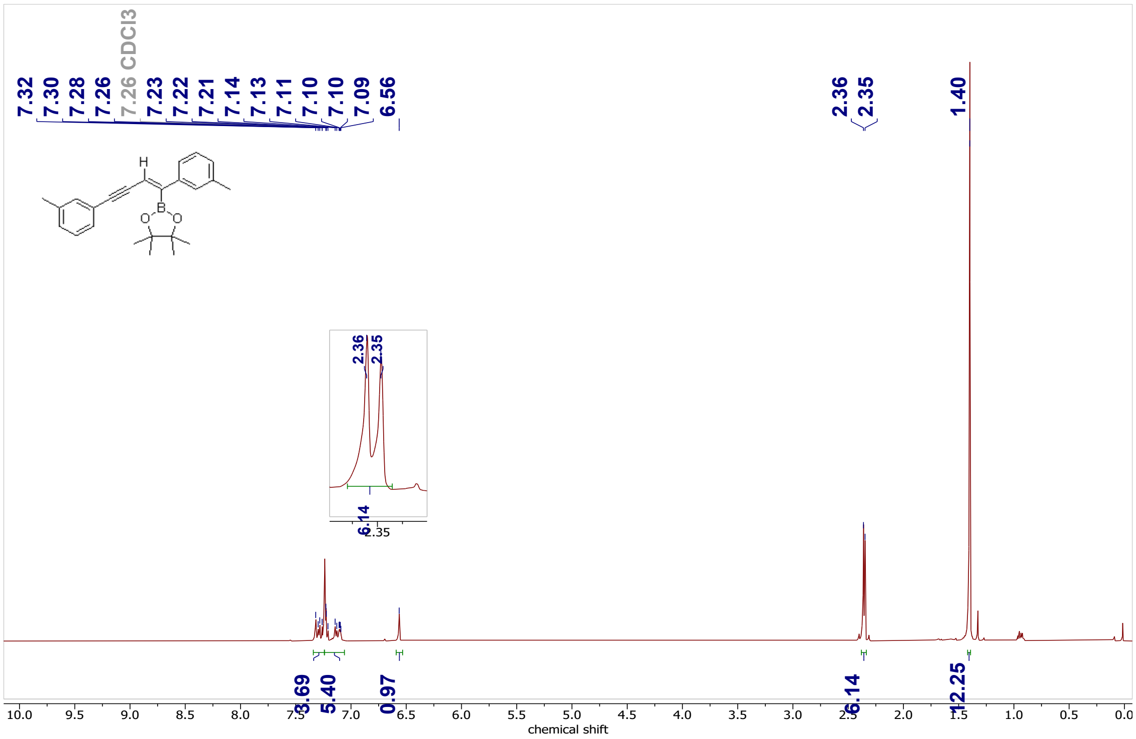

7.32
7.30
7.28
7.26
7.26 CDCl3
7.23
7.22
7.21
7.14
7.13
7.11
7.10
7.10
7.09
6.56
2.36
2.35
1.40
6.14
3.69
5.40
0.97
12.25
chemical shift

2c-^{13}C

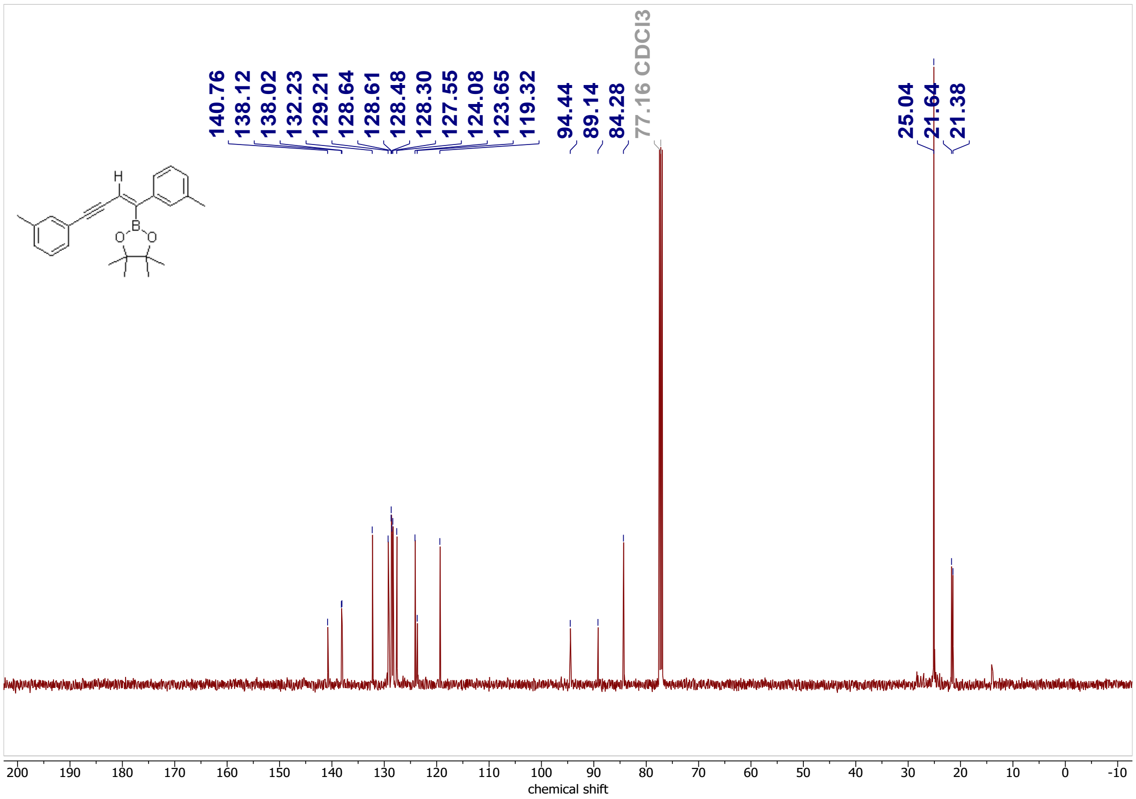
140.76
138.12
138.02
132.23
129.21
128.64
128.61
128.48
128.30
127.55
124.08
123.65
119.32
94.44
89.14
84.28
77.16 CDCl3
25.04
21.64
21.38
H
O
B
O
chemical shift

2c-^{11}B

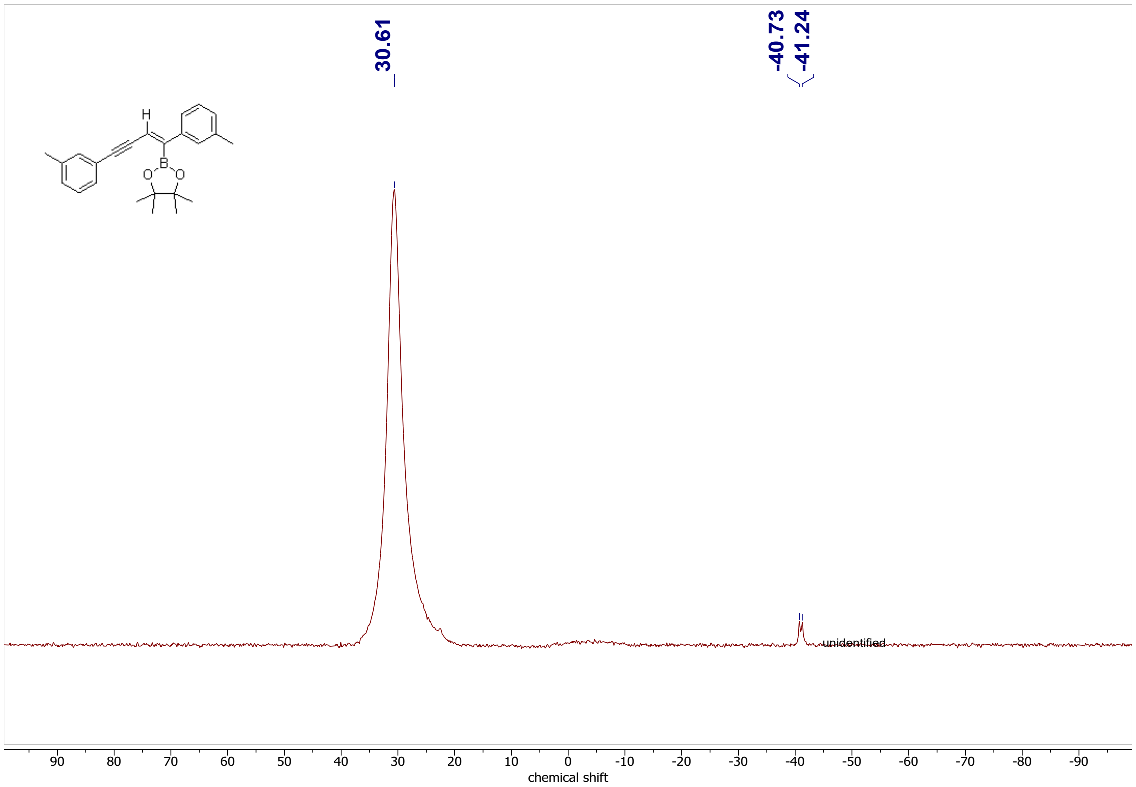
30.61
-40.73
-41.24
H
O
B
O
unidentified
chemical shift

2d-^{1}H

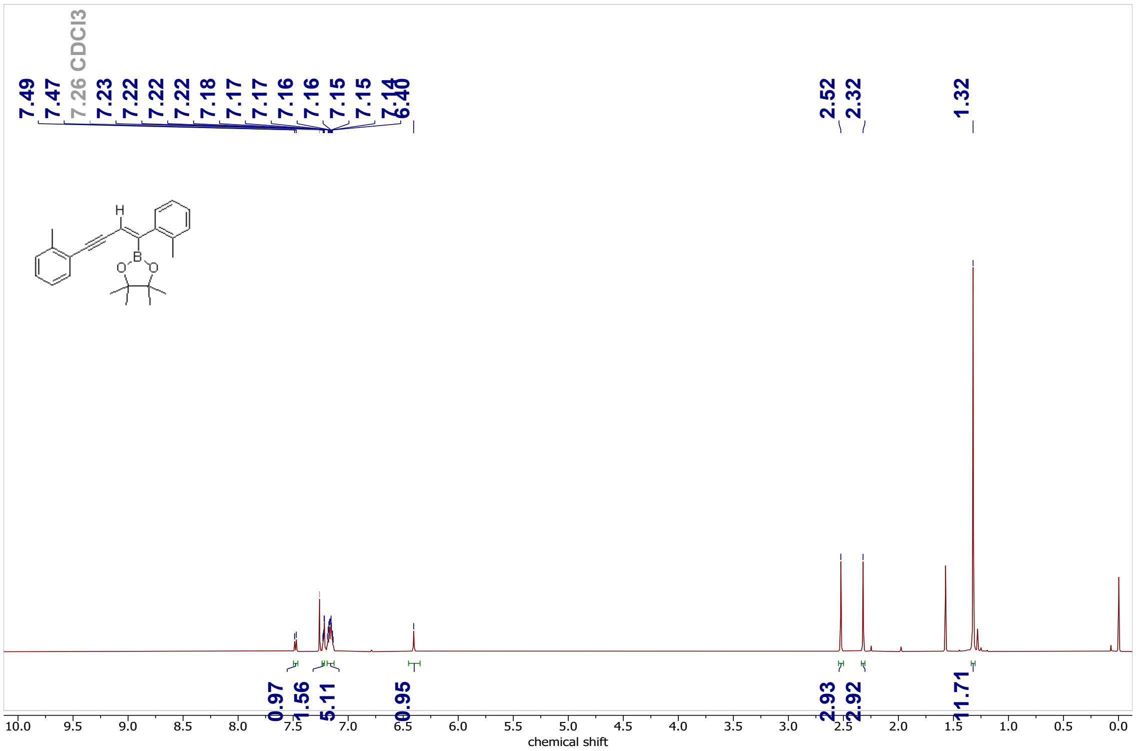
7.49
7.47
7.26 CDCl3
7.23
7.22
7.22
7.22
7.18
7.17
7.17
7.16
7.16
7.15
7.15
7.14
6.40
2.52
2.32
1.32
0.97
1.56
5.11
0.95
2.93
2.92
11.71
chemical shift

2d-^{13}C

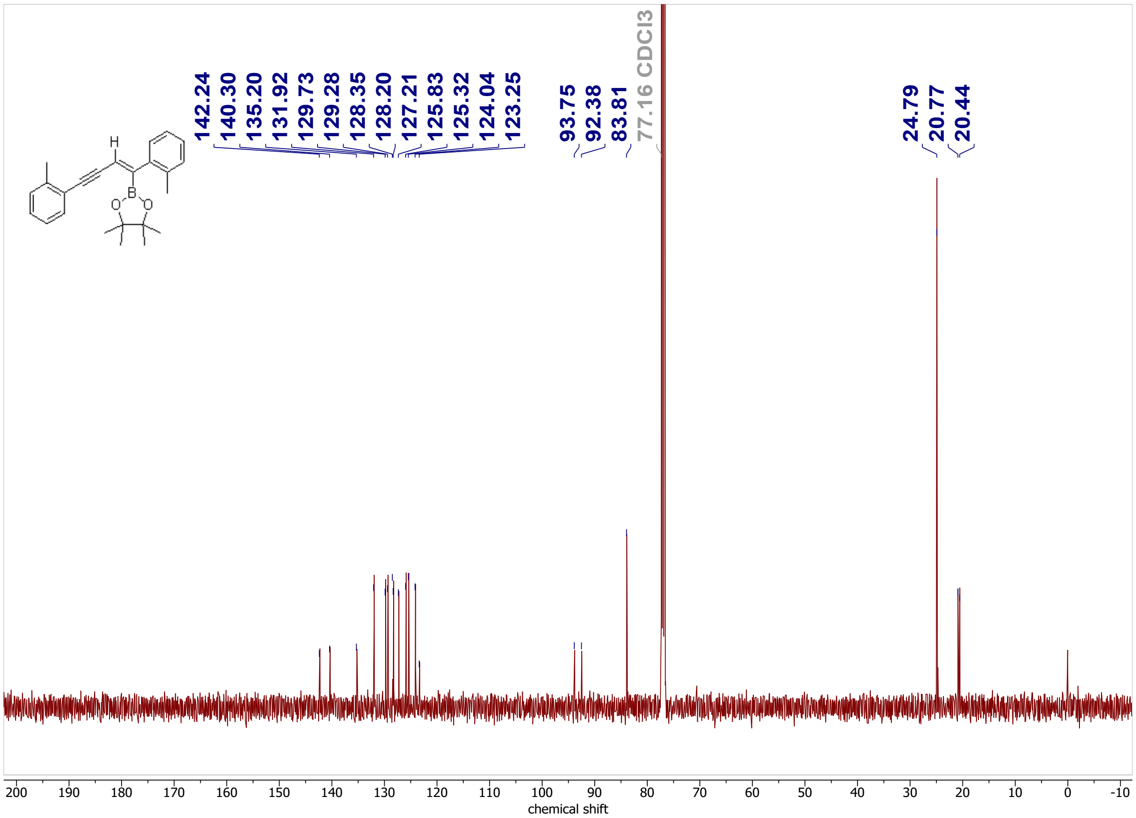
142.24
140.30
135.20
131.92
129.73
129.28
128.35
128.20
127.21
125.83
125.32
124.04
123.25
93.75
92.38
83.81
77.16 CDCl3
24.79
20.77
20.44
chemical shift

2d-^{11}B

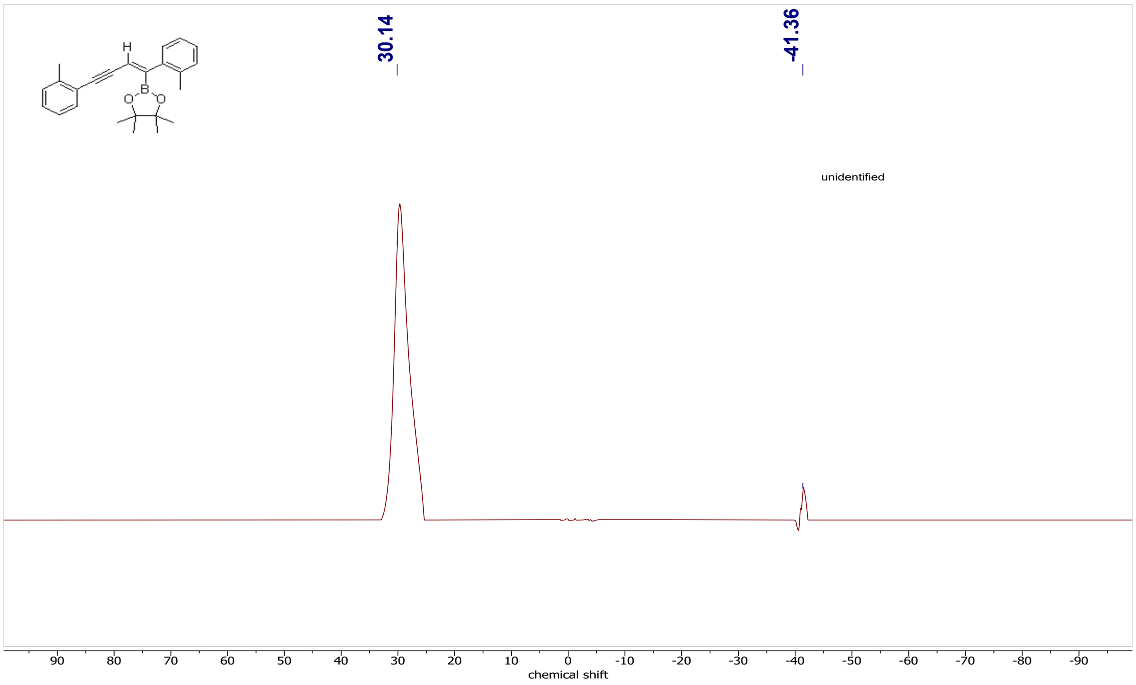
30.14
-41.36
unidentified
chemical shift

2e-^{1}H

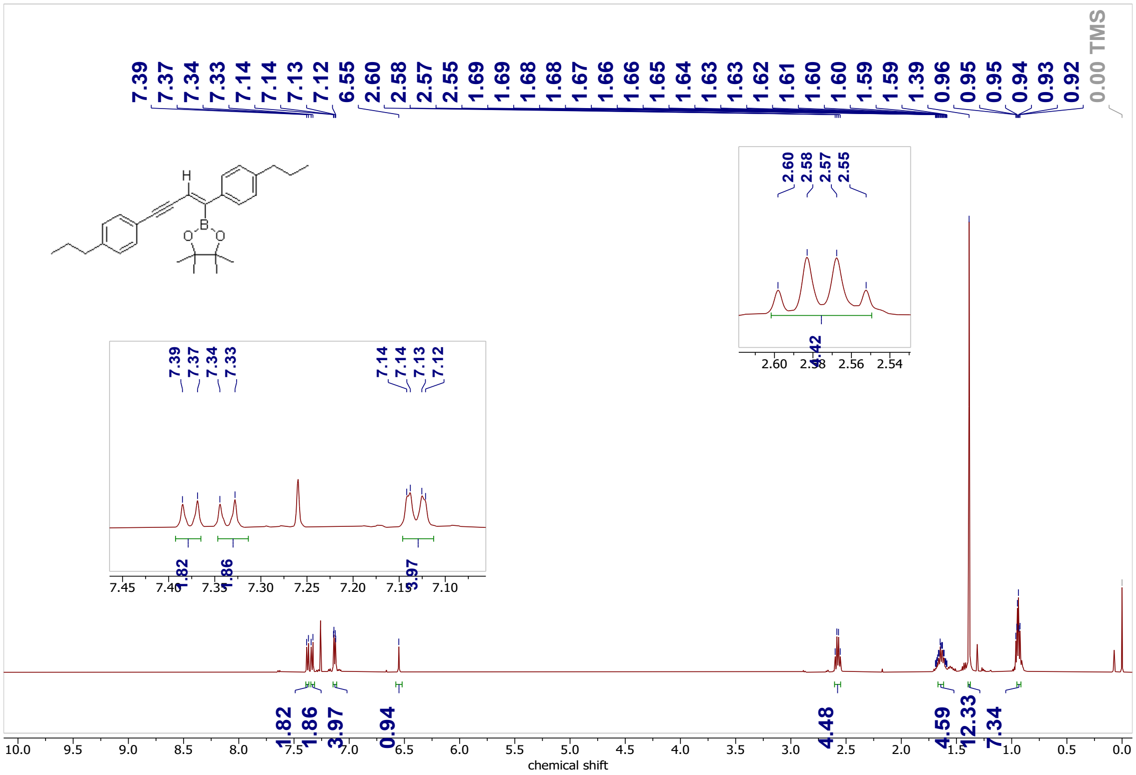
7.39
7.37
7.34
7.33
7.14
7.14
7.13
7.12
6.55
2.60
2.58
2.57
2.55
1.69
1.69
1.68
1.68
1.67
1.66
1.66
1.65
1.64
1.63
1.63
1.62
1.61
1.60
1.60
1.59
1.59
1.39
0.96
0.95
0.95
0.94
0.93
0.92
0.00 TMS
1.82
1.86
3.97
0.94
4.48
4.59
12.33
7.34
4.42
chemical shift

2e-^{13}C

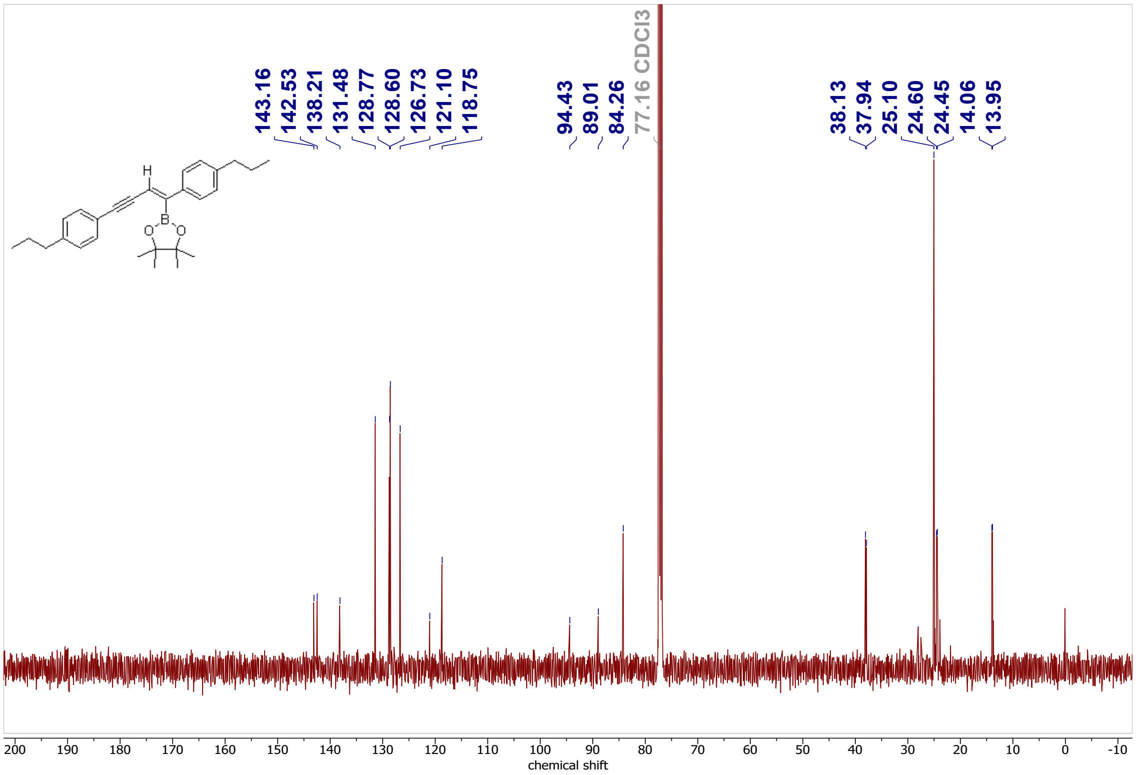
143.16
142.53
138.21
131.48
128.77
128.60
126.73
121.10
118.75
94.43
89.01
84.26
77.16 CDCl3
38.13
37.94
25.10
24.60
24.45
14.06
13.95
H
B
O
O
200 190 180 170 160 150 140 130 120 110 100 90 80 70 60 50 40 30 20 10 0 -10
chemical shift

2e-^{11}B

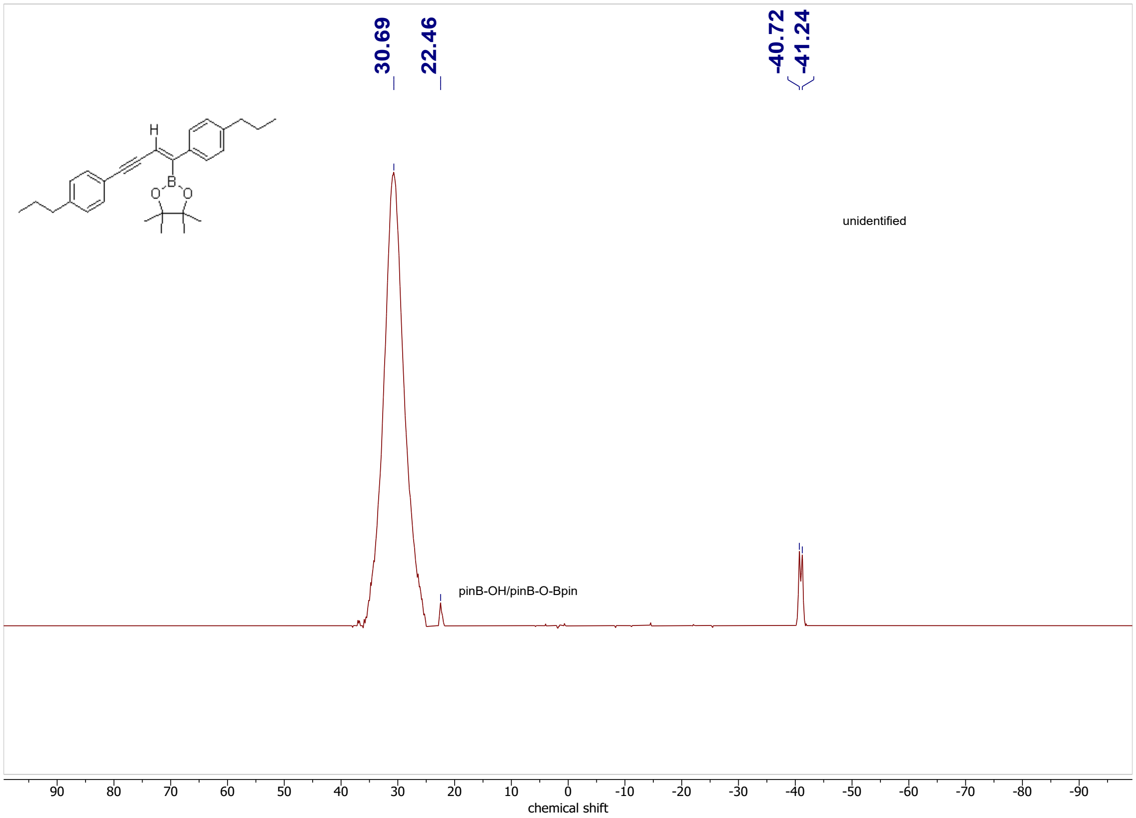
30.69
22.46
-40.72
-41.24
H
B
O
O
unidentified
pinB-OH/pinB-O-Bpin
90 80 70 60 50 40 30 20 10 0 -10 -20 -30 -40 -50 -60 -70 -80 -90
chemical shift

2f-^{1}H

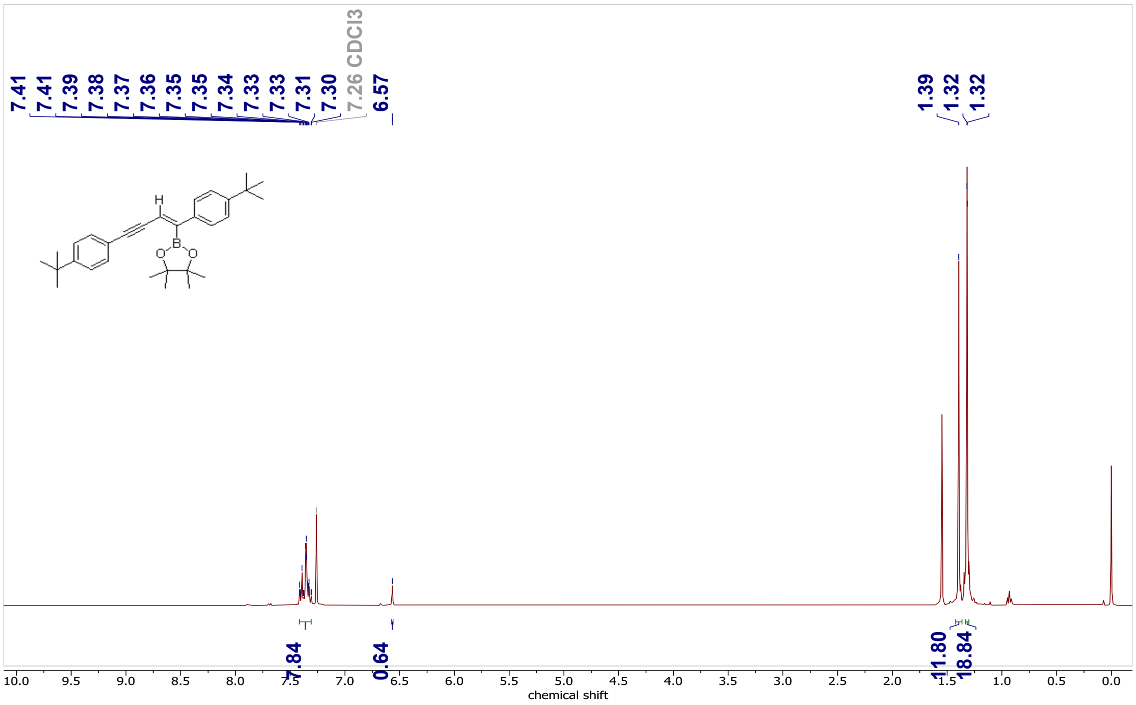
7.41
7.41
7.39
7.38
7.37
7.36
7.35
7.35
7.34
7.33
7.33
7.31
7.30
7.26 CDCl3
6.57
1.39
1.32
1.32
H
B
O
O
7.84
0.64
11.80
18.84
10.0 9.5 9.0 8.5 8.0 7.5 7.0 6.5 6.0 5.5 5.0 4.5 4.0 3.5 3.0 2.5 2.0 1.5 1.0 0.5 0.0
chemical shift

2f-^{13}C

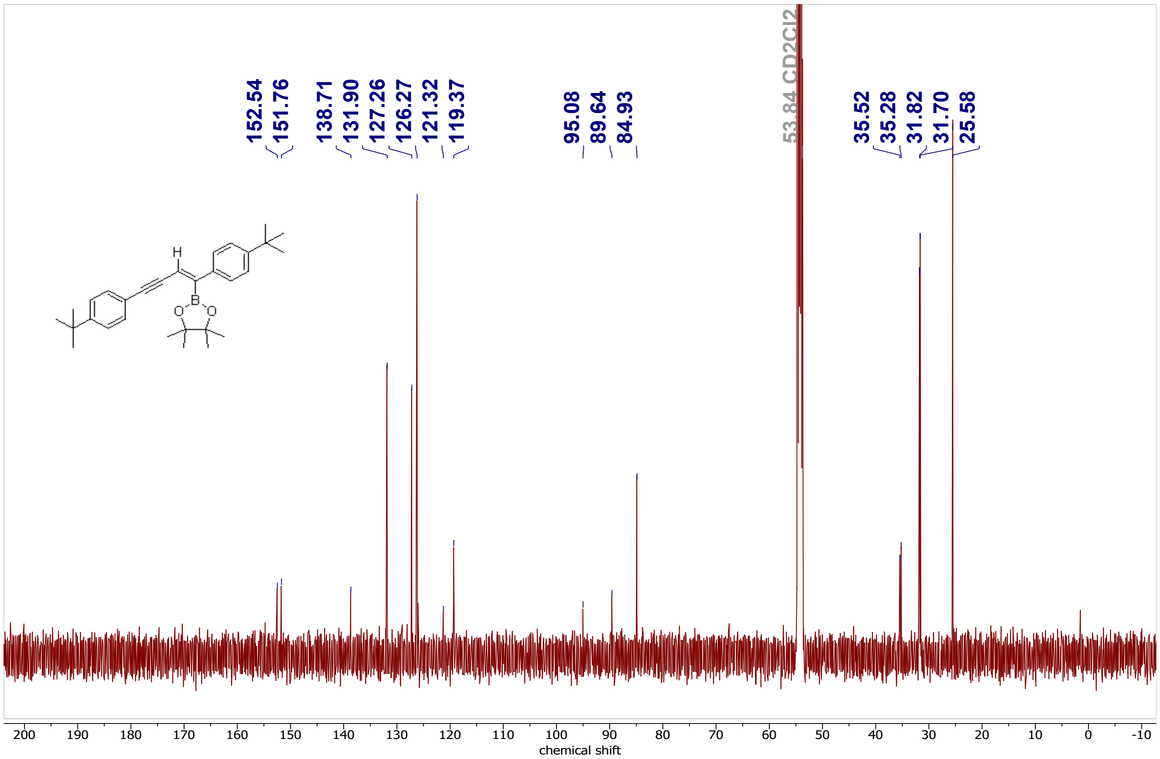
152.54
151.76
138.71
131.90
127.26
126.27
121.32
119.37
95.08
89.64
84.93
53.84 CD2Cl2
35.52
35.28
31.82
31.70
25.58
H
B
O
O
200 190 180 170 160 150 140 130 120 110 100 90 80 70 60 50 40 30 20 10 0 -10
chemical shift

2f-[11]B

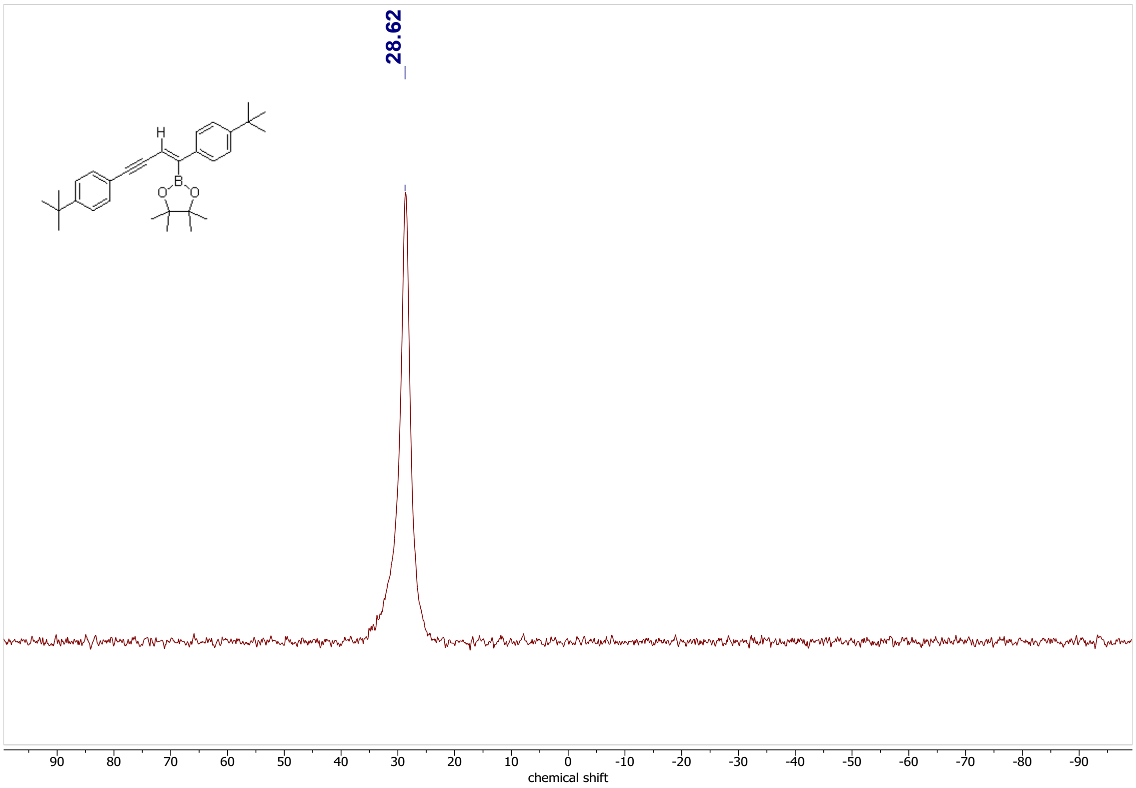

28.62
H
B
O
O
90
80
70
60
50
40
30
20
10
0
-10
-20
-30
-40
-50
-60
-70
-80
-90
chemical shift

2g-[1]H

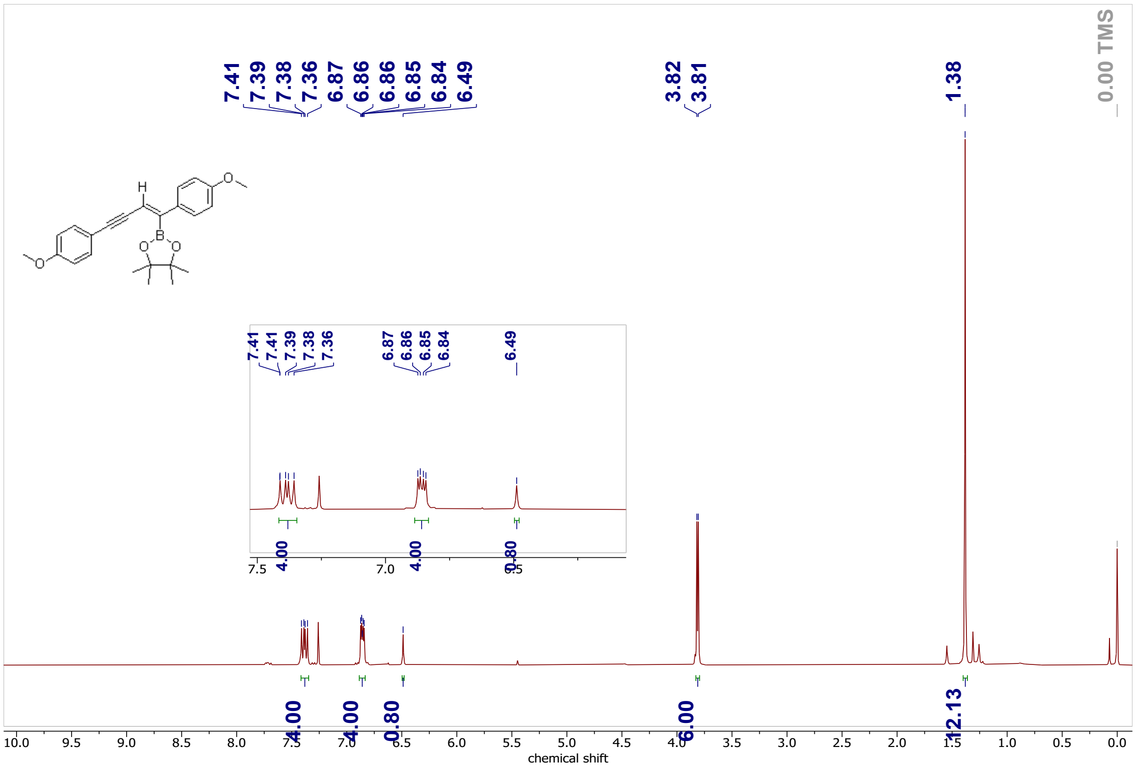

7.41
7.39
7.38
7.36
6.87
6.86
6.86
6.85
6.84
6.49
3.82
3.81
1.38
0.00 TMS
H
B
O
O
O
O
4.00
4.00
0.80
6.00
12.13
chemical shift

2g-^{13}C

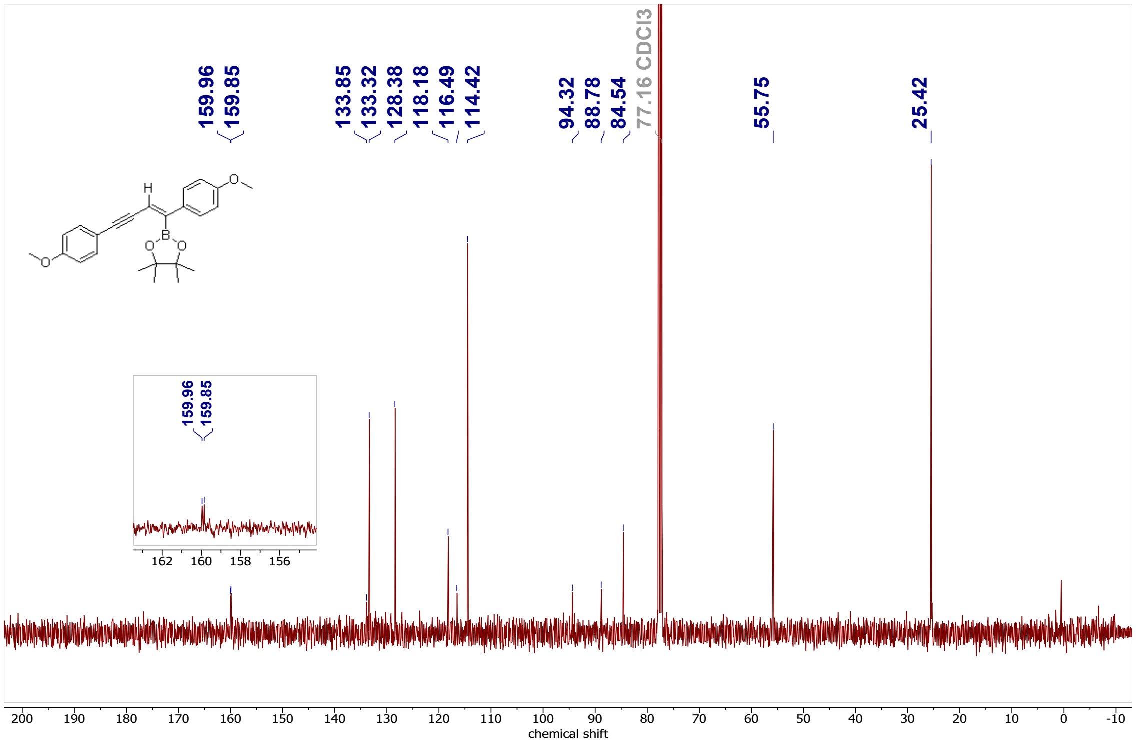

159.96
159.85
133.85
133.32
128.38
118.18
116.49
114.42
94.32
88.78
84.54
77.16 CDCl3
55.75
25.42
162
160
158
156
200
190
180
170
160
150
140
130
120
110
100
90
80
70
60
50
40
30
20
10
0
-10
chemical shift

2g-^{11}B

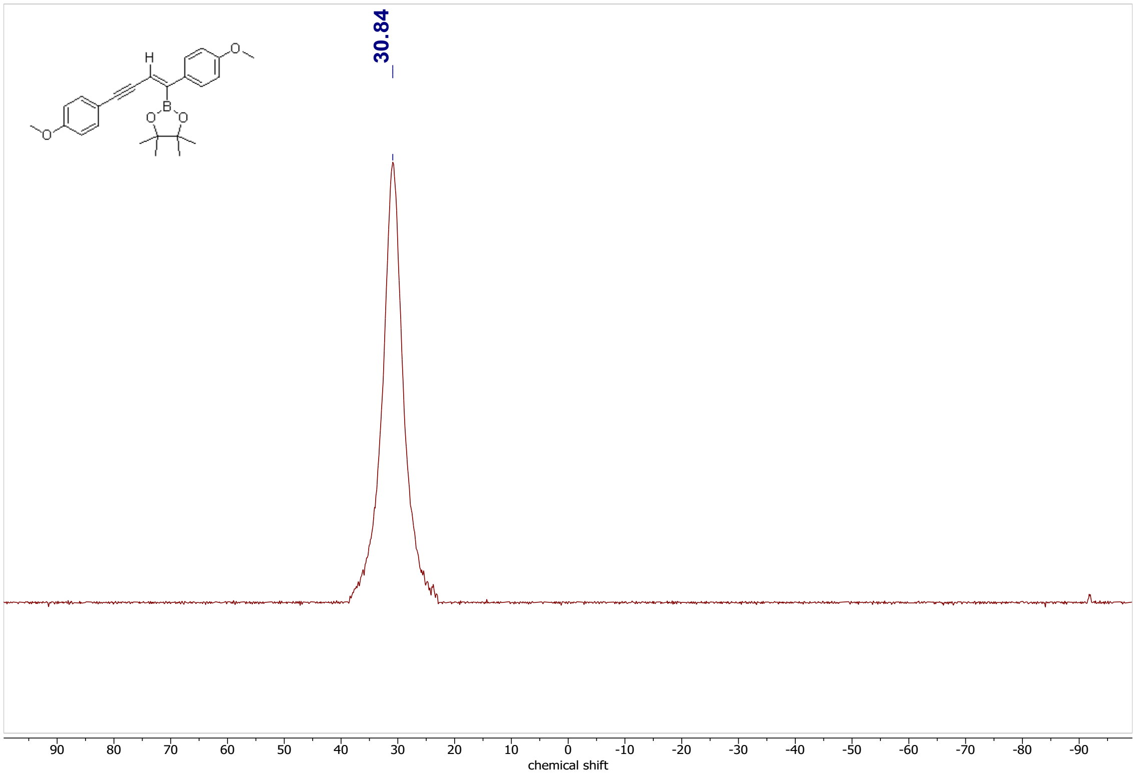

30.84
90
80
70
60
50
40
30
20
10
0
-10
-20
-30
-40
-50
-60
-70
-80
-90
chemical shift

2g1-¹H

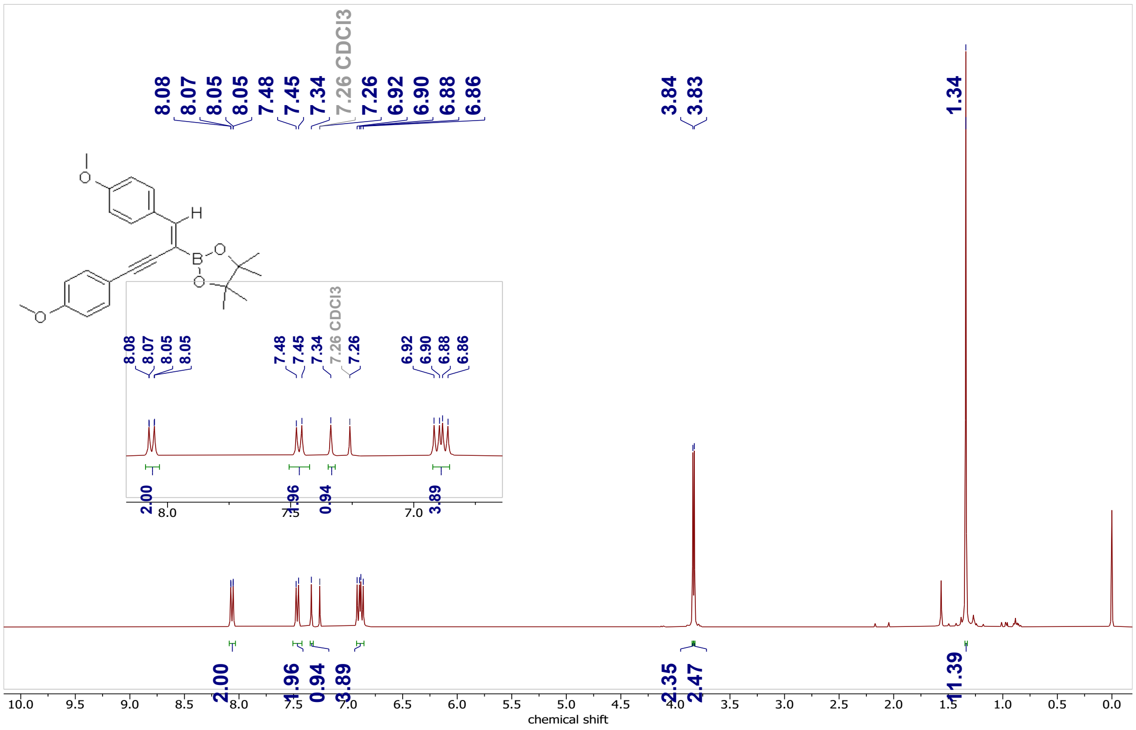

2g1-¹³C

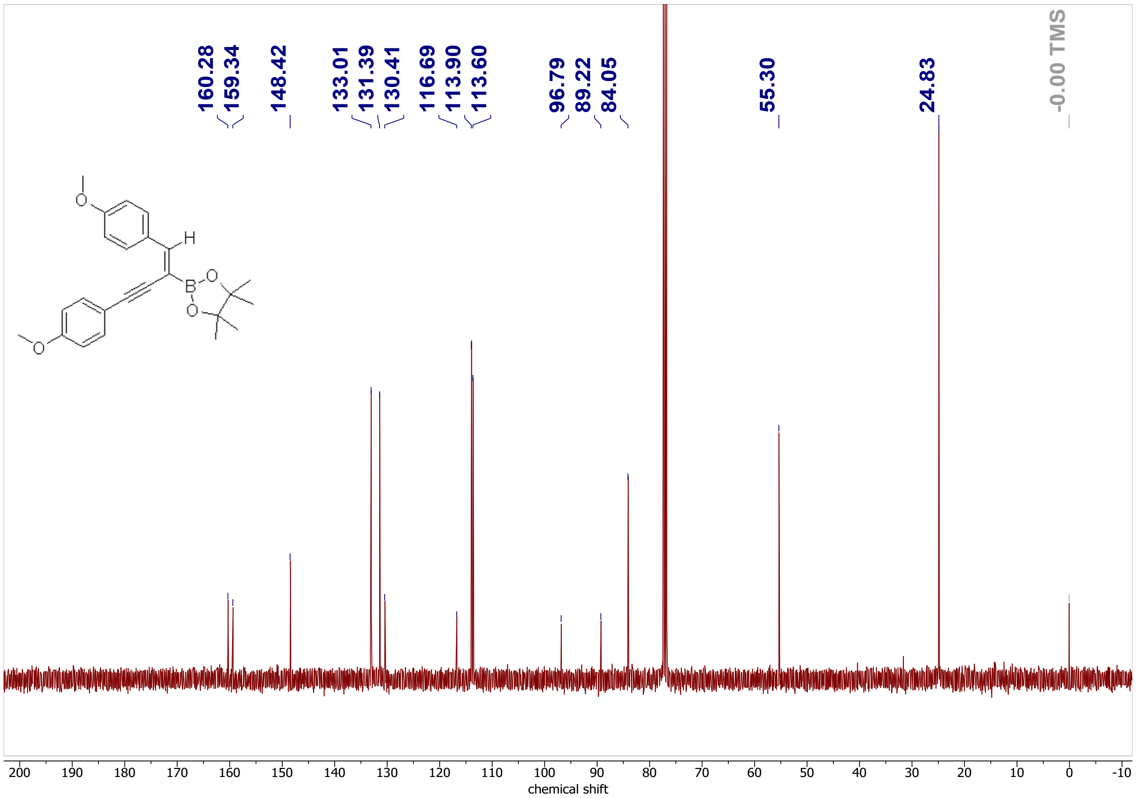

2g1-^{11}B

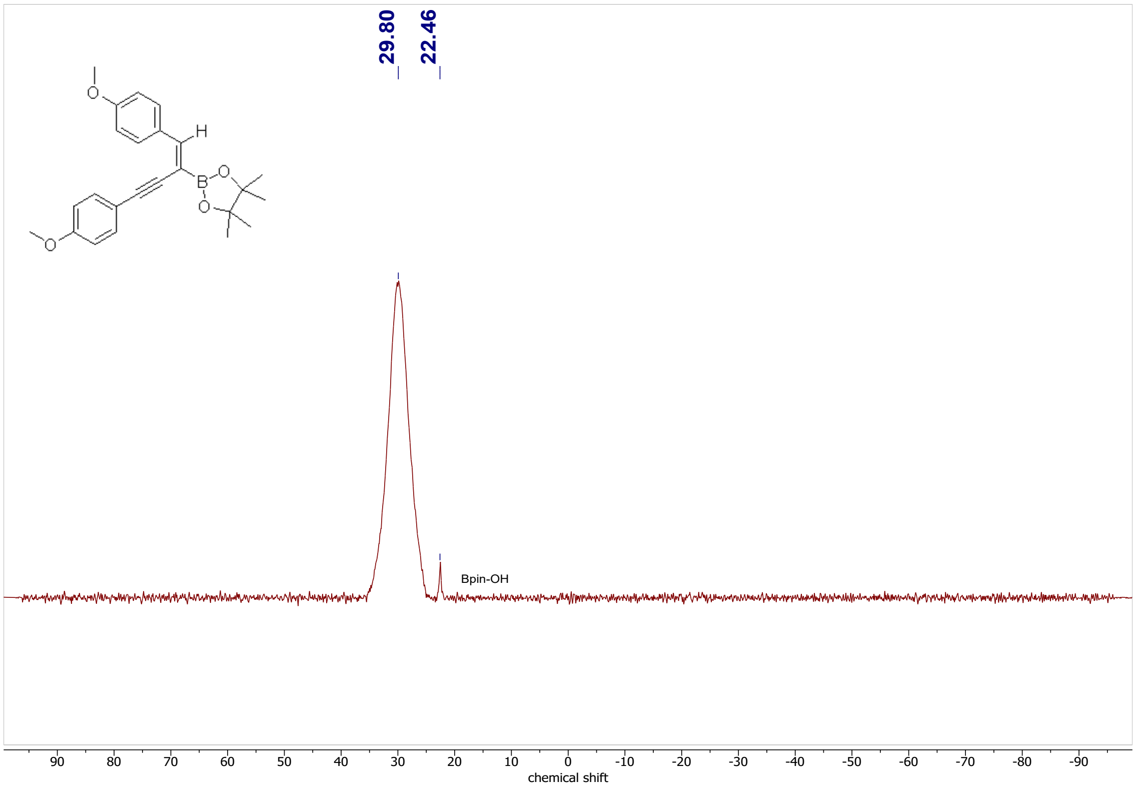

2h-^{1}H

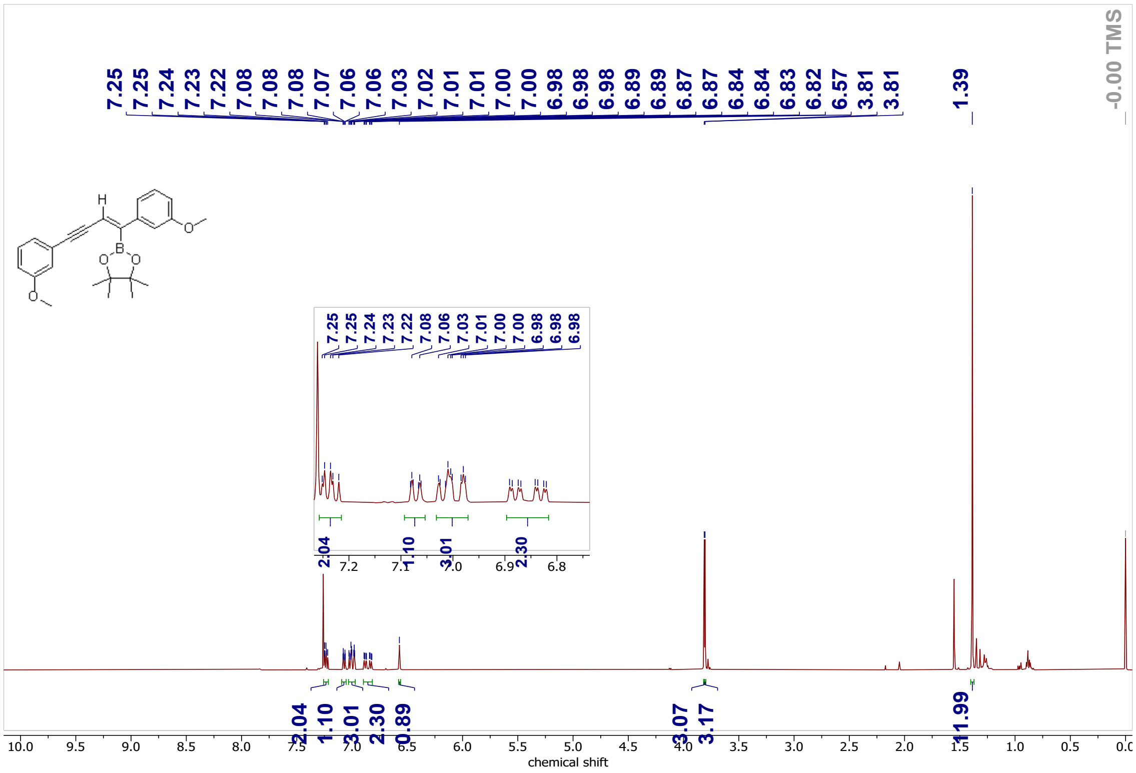

2h-^{13}C

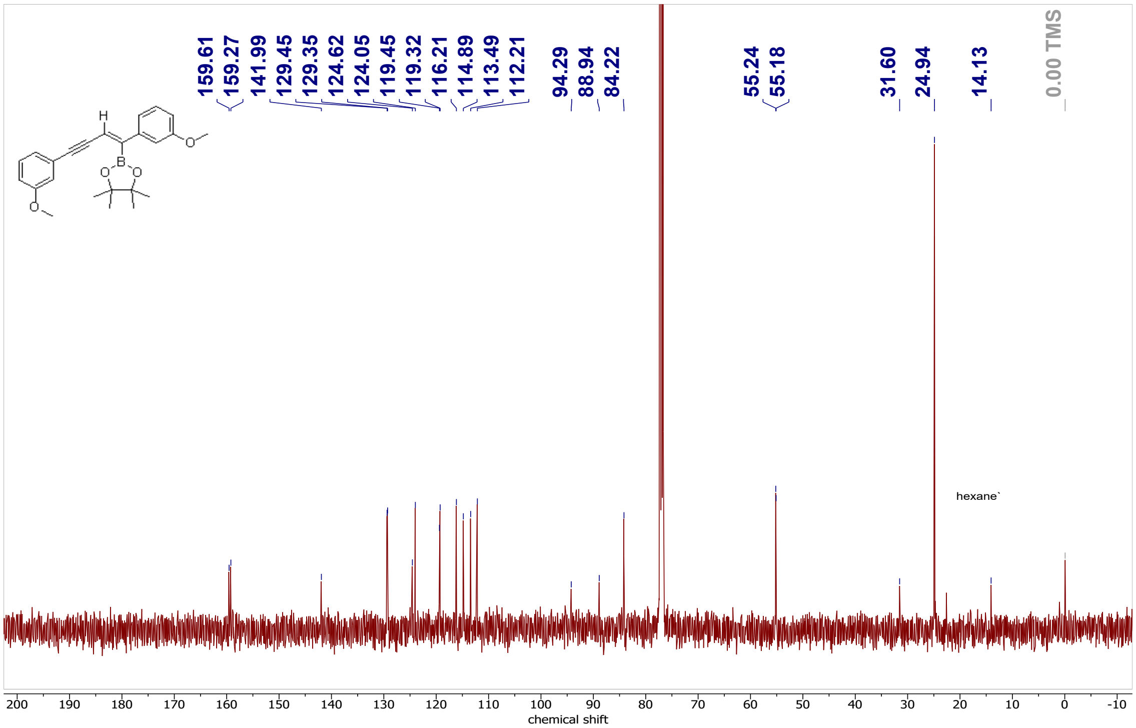
159.61
159.27
141.99
129.45
129.35
124.62
124.05
119.45
119.32
116.21
114.89
113.49
112.21
94.29
88.94
84.22
55.24
55.18
31.60
24.94
14.13
0.00 TMS
H
O
B
O
O
O
hexane`
200
190
180
170
160
150
140
130
120
110
100
90
80
70
60
50
40
30
20
10
0
-10
chemical shift

2h-^{11}B

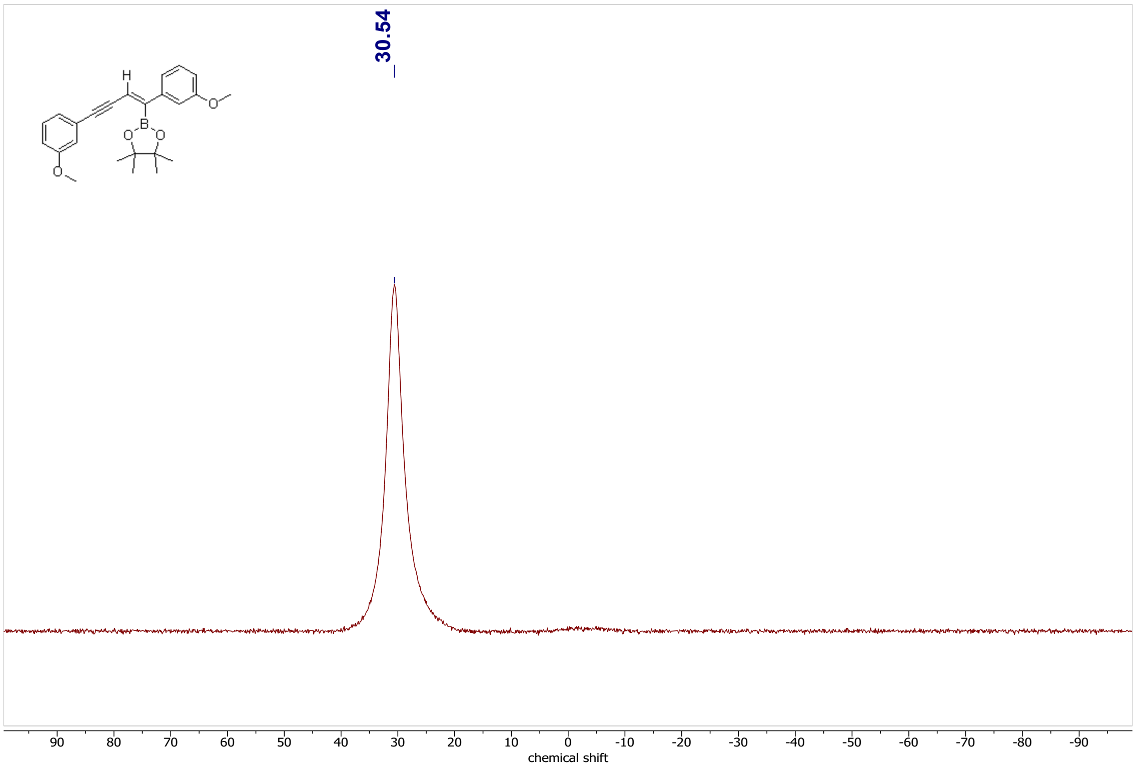
30.54
H
O
B
O
O
O
90
80
70
60
50
40
30
20
10
0
-10
-20
-30
-40
-50
-60
-70
-80
-90
chemical shift

2i-^{1}H

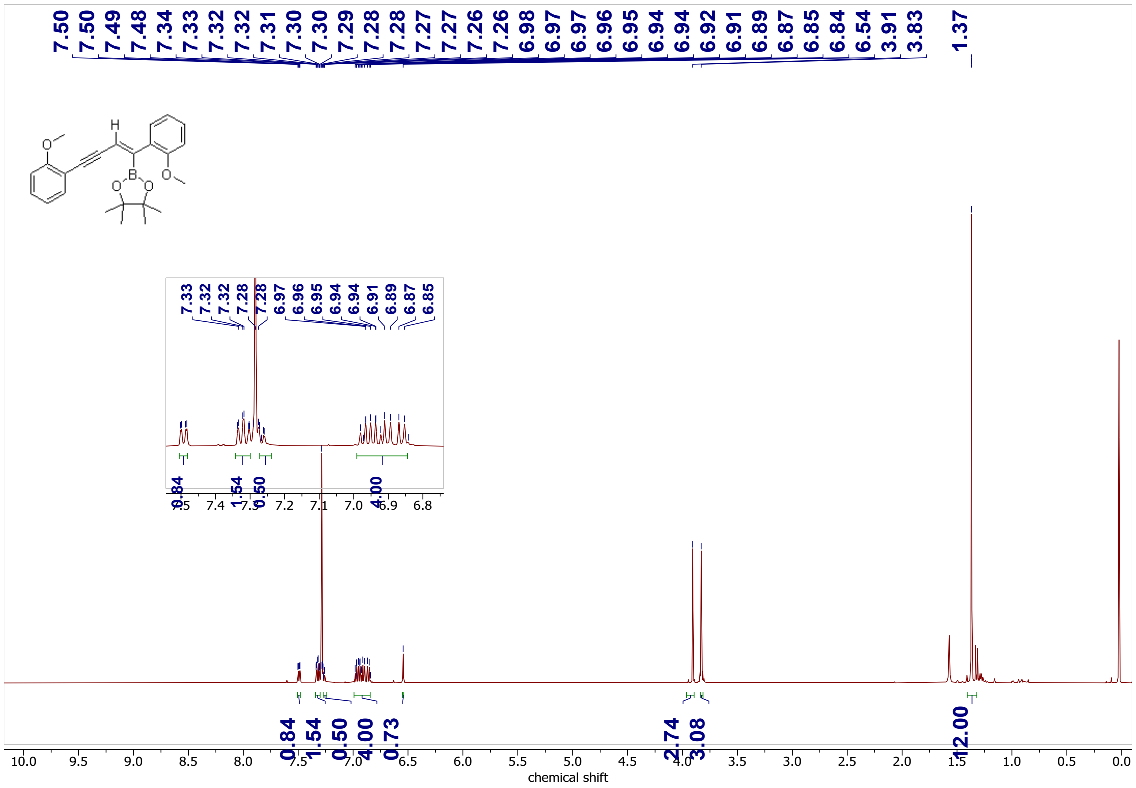

2i-^{13}C

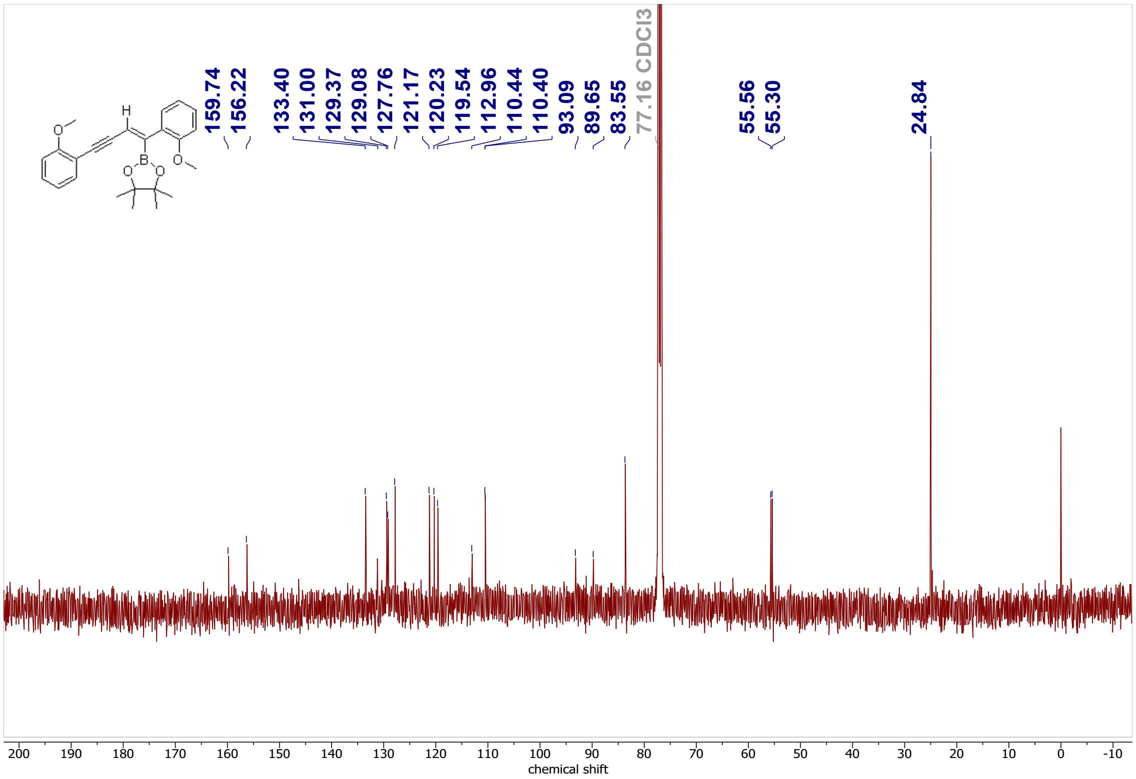

2i-^{11}B

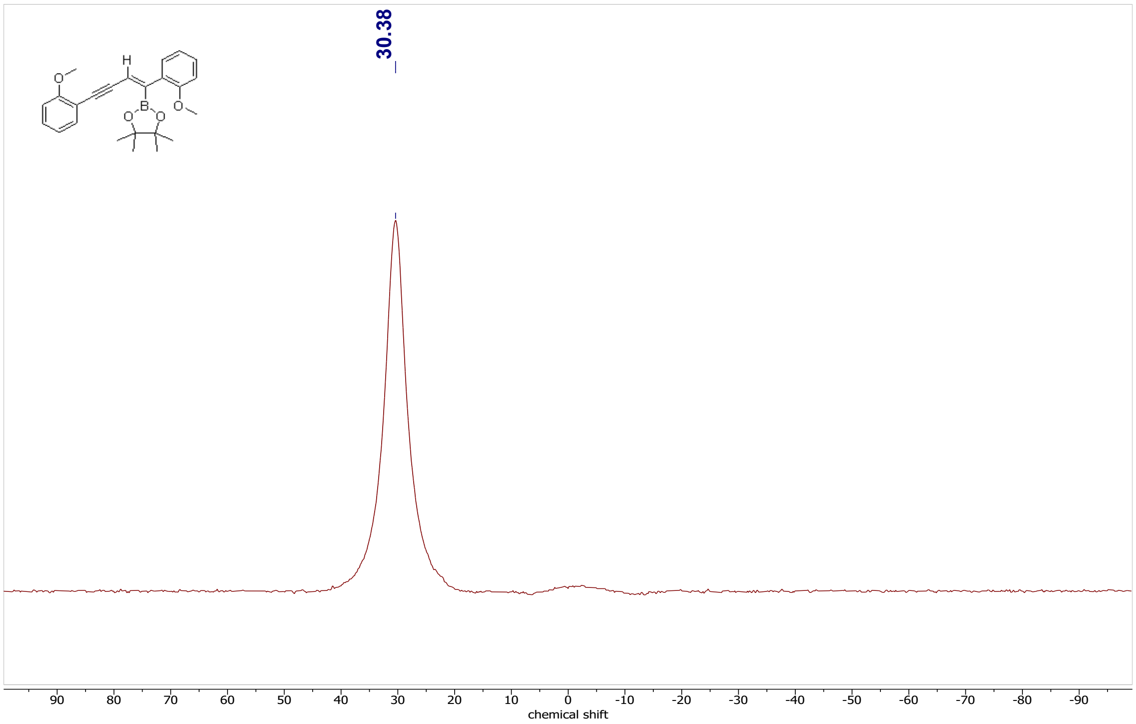
30.38
chemical shift

2j-^{1}H

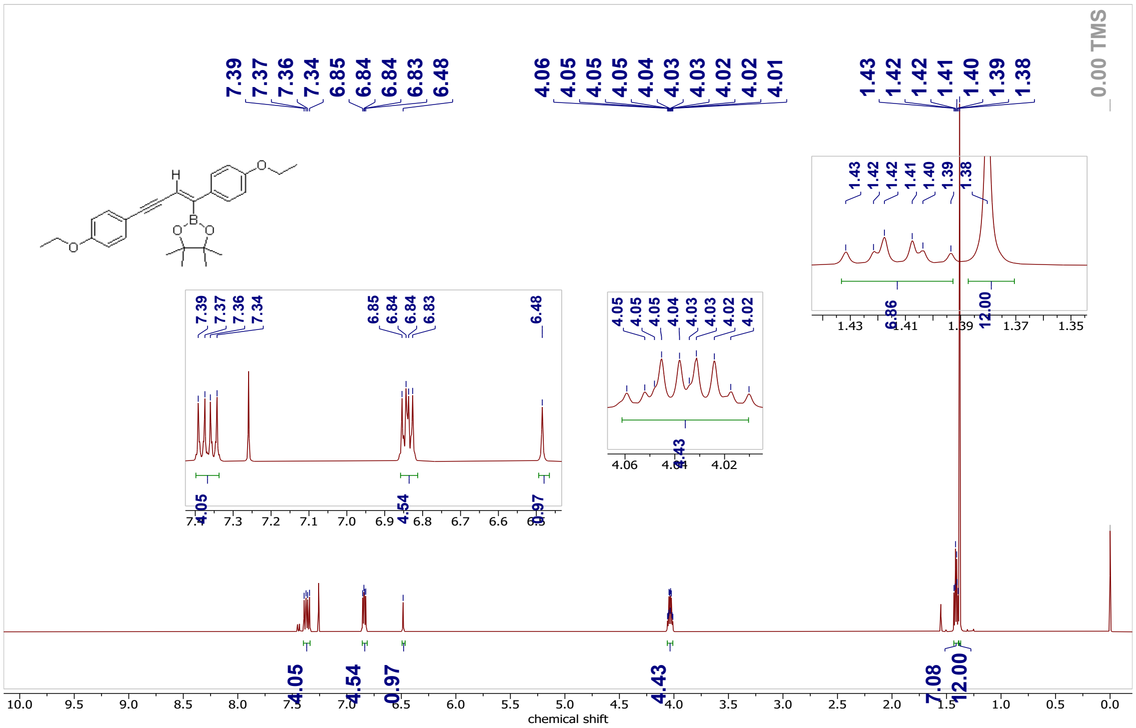
7.39
7.37
7.36
7.34
6.85
6.84
6.84
6.83
6.48
4.06
4.05
4.05
4.05
4.04
4.03
4.03
4.02
4.02
4.01
1.43
1.42
1.42
1.41
1.40
1.39
1.38
0.00 TMS
4.05
4.54
0.97
4.43
7.08
12.00
chemical shift

2j-^{13}C

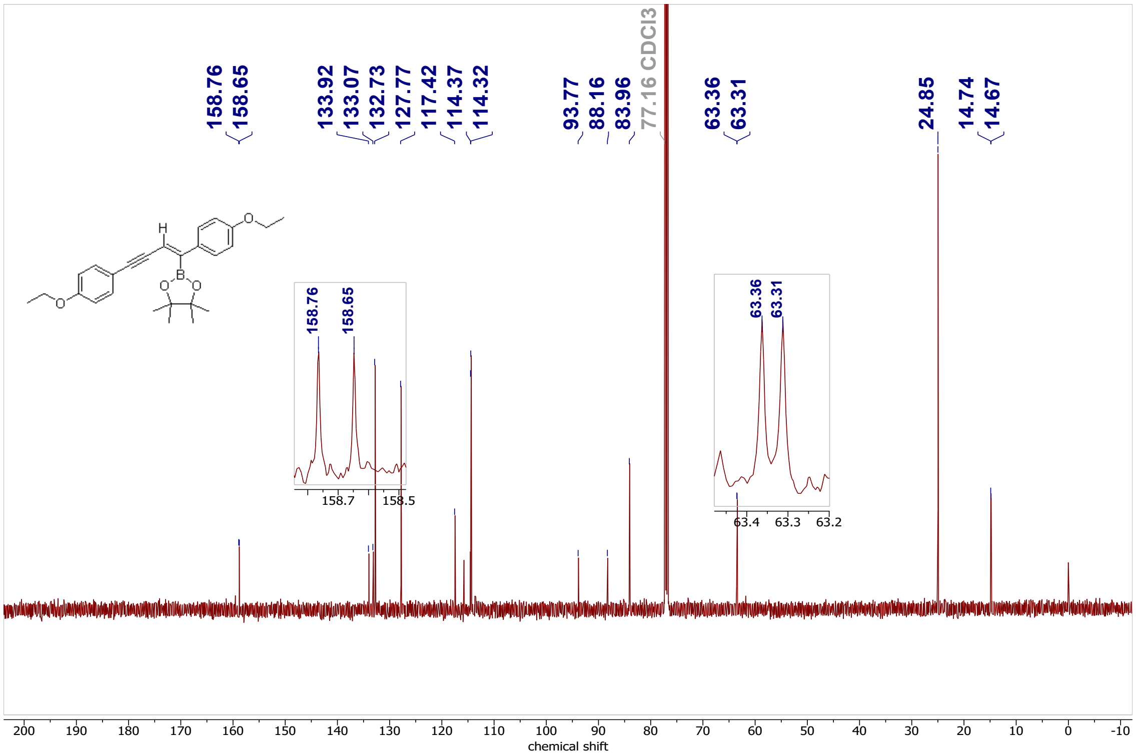
158.76
158.65
133.92
133.07
132.73
127.77
117.42
114.37
114.32
93.77
88.16
83.96
77.16 CDCl3
63.36
63.31
24.85
14.74
14.67
H
O
B
chemical shift

2j-^{11}B

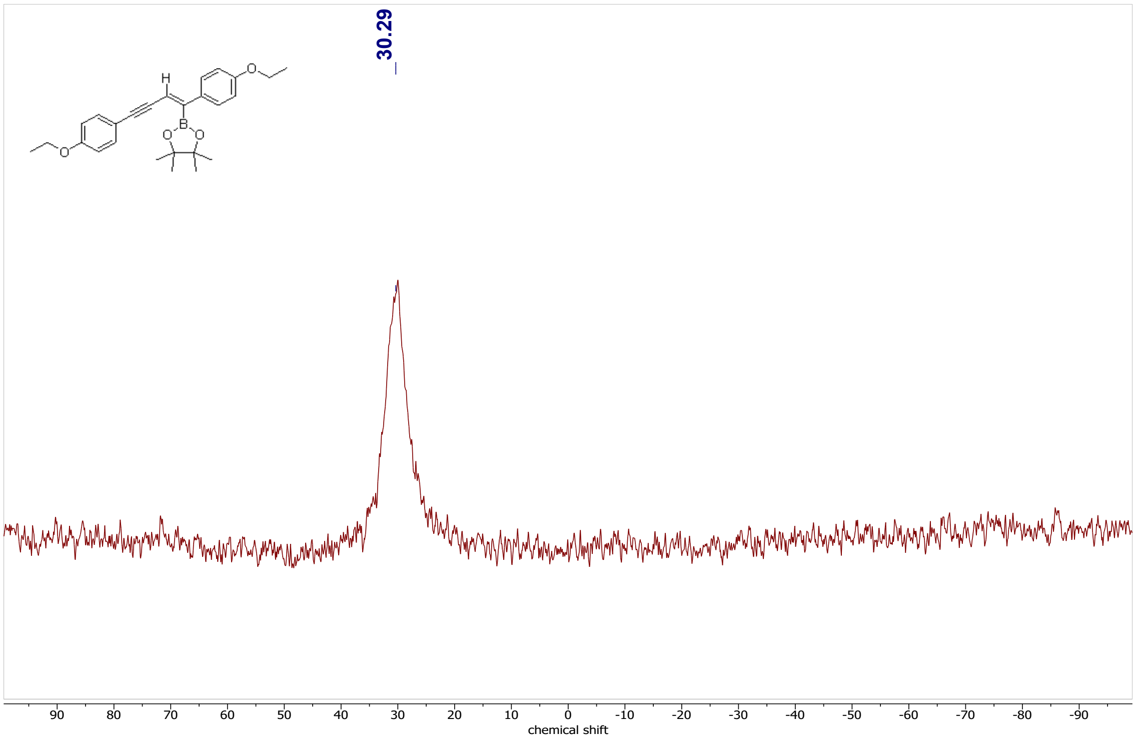
30.29
H
O
B
chemical shift

2j1-^{1}H

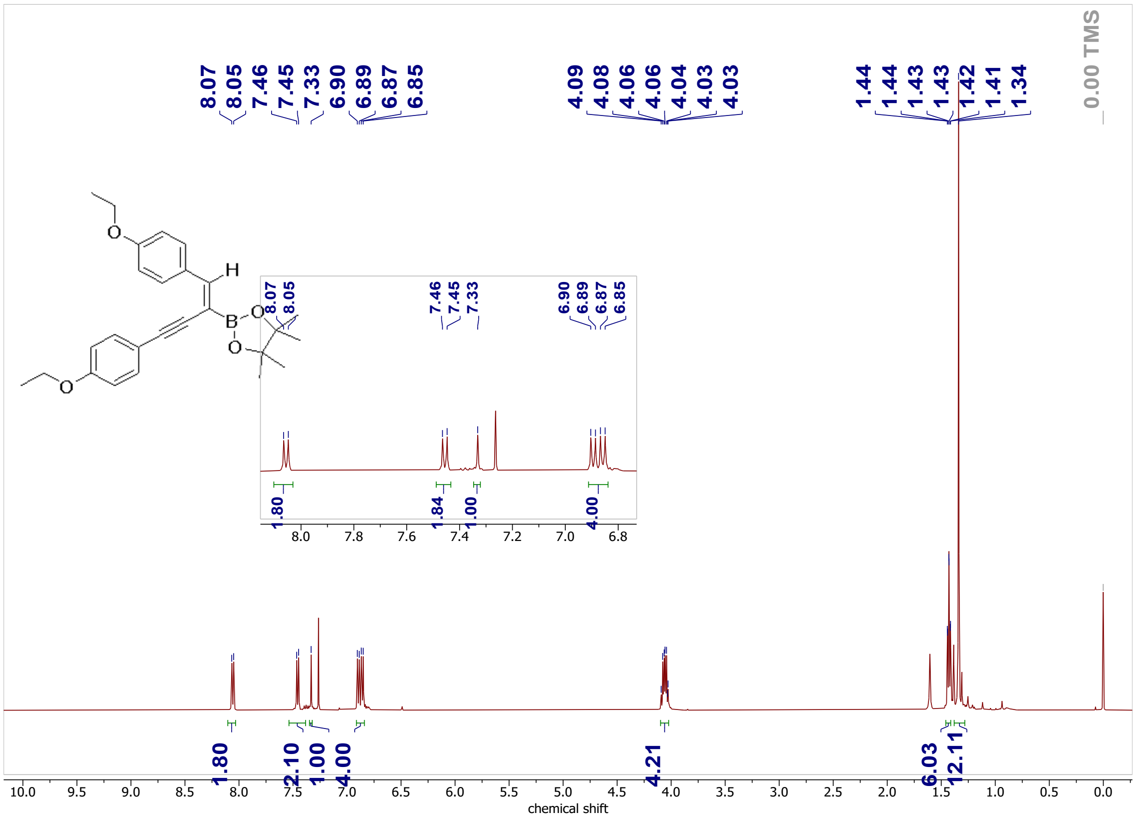

2j1-^{13}C

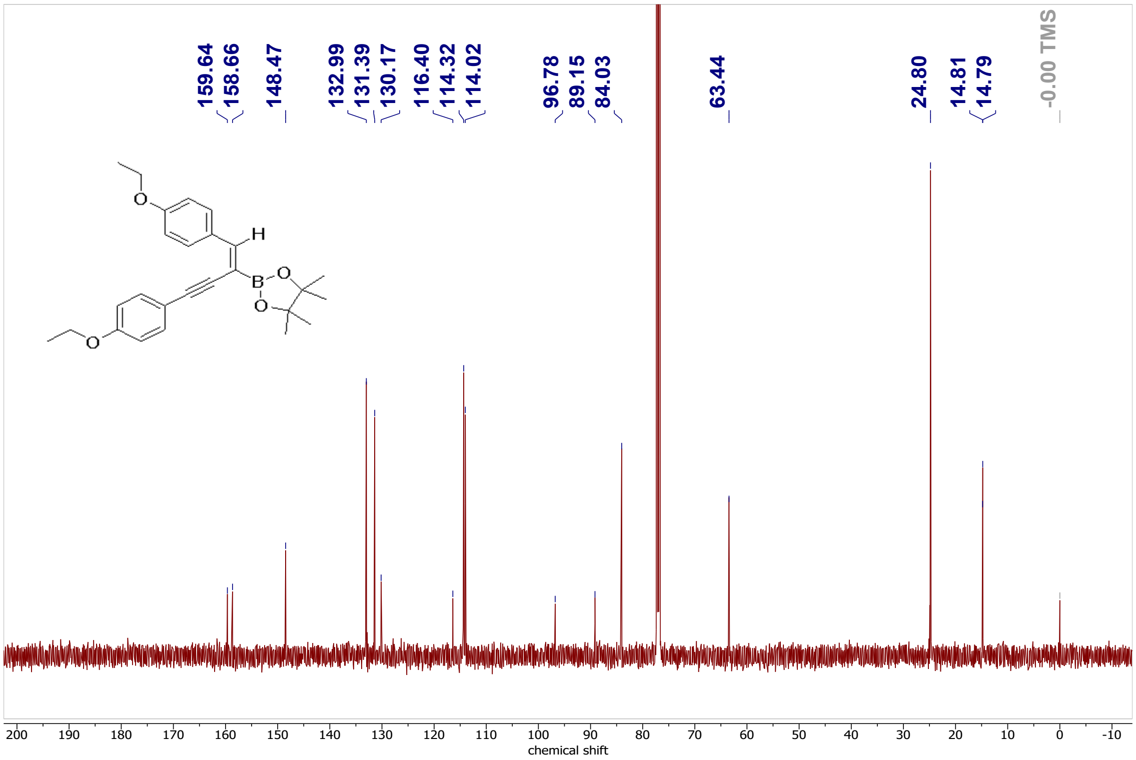

2j1-^{11}B

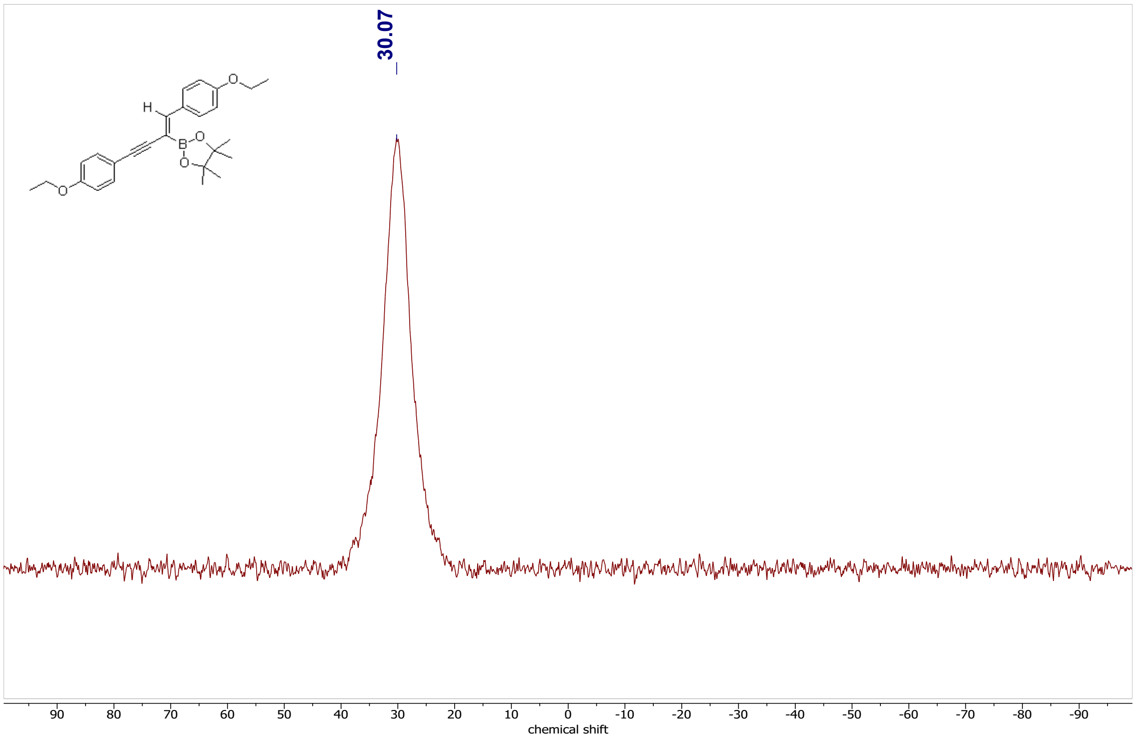

30.07
H
O
B
O
O
O
chemical shift
90
80
70
60
50
40
30
20
10
0
-10
-20
-30
-40
-50
-60
-70
-80
-90

2k-^{1}H

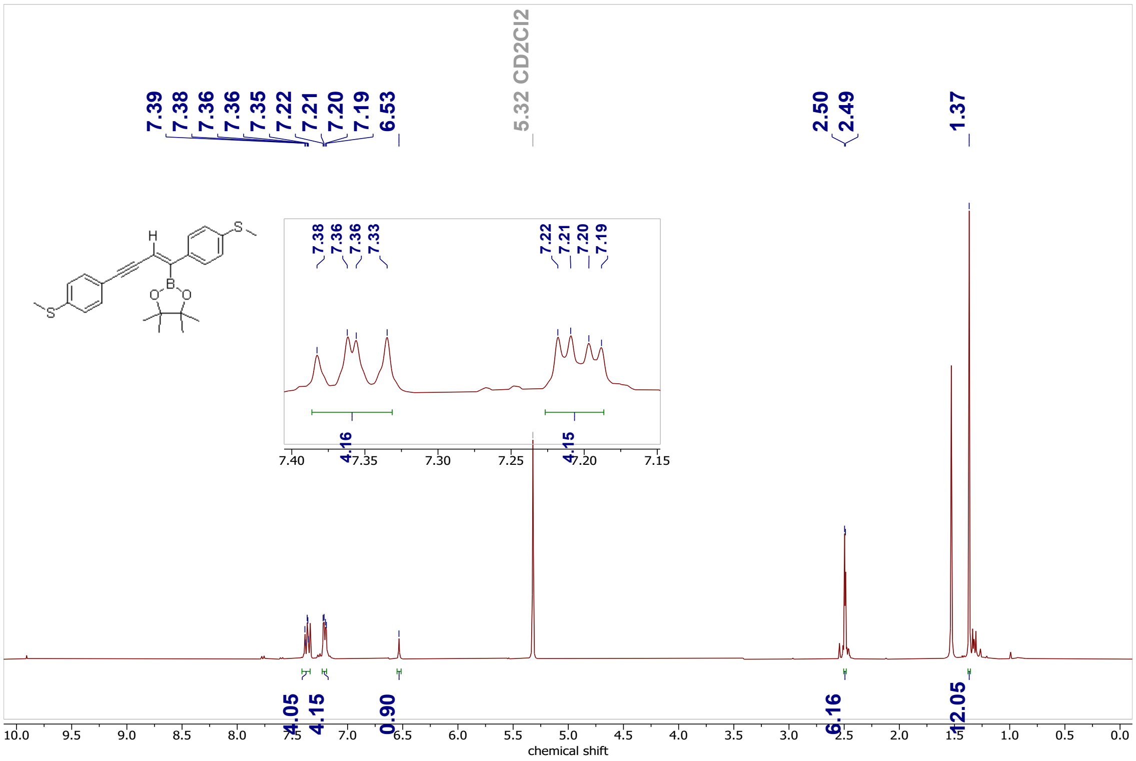

7.39
7.38
7.36
7.36
7.35
7.22
7.21
7.20
7.19
6.53
5.32 CD2Cl2
2.50
2.49
1.37
H
S
S
B
O
O
7.38
7.36
7.36
7.33
7.22
7.21
7.20
7.19
4.16
4.15
7.40
7.35
7.30
7.25
7.20
7.15
4.05
4.15
0.90
6.16
12.05
chemical shift

2k-^{13}C

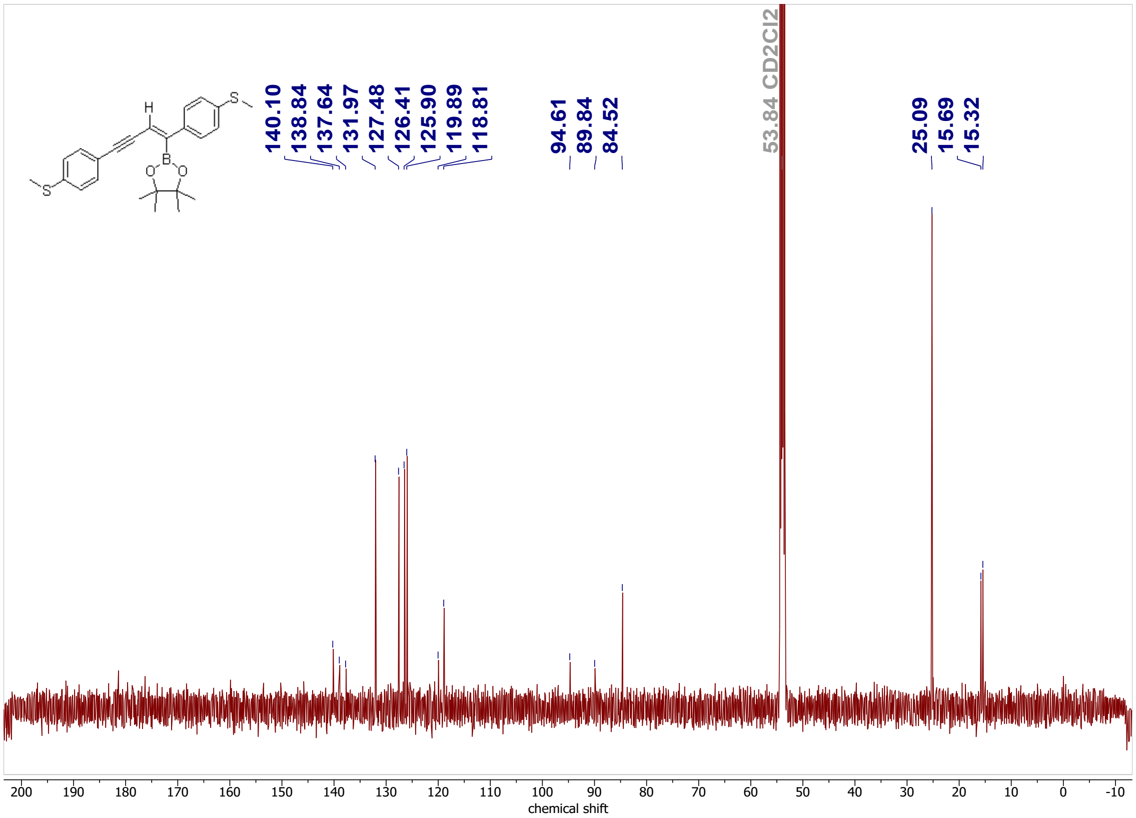
140.10
138.84
137.64
131.97
127.48
126.41
125.90
119.89
118.81
94.61
89.84
84.52
53.84 CD2Cl2
25.09
15.69
15.32
200 190 180 170 160 150 140 130 120 110 100 90 80 70 60 50 40 30 20 10 0 -10
chemical shift

2k-^{11}B

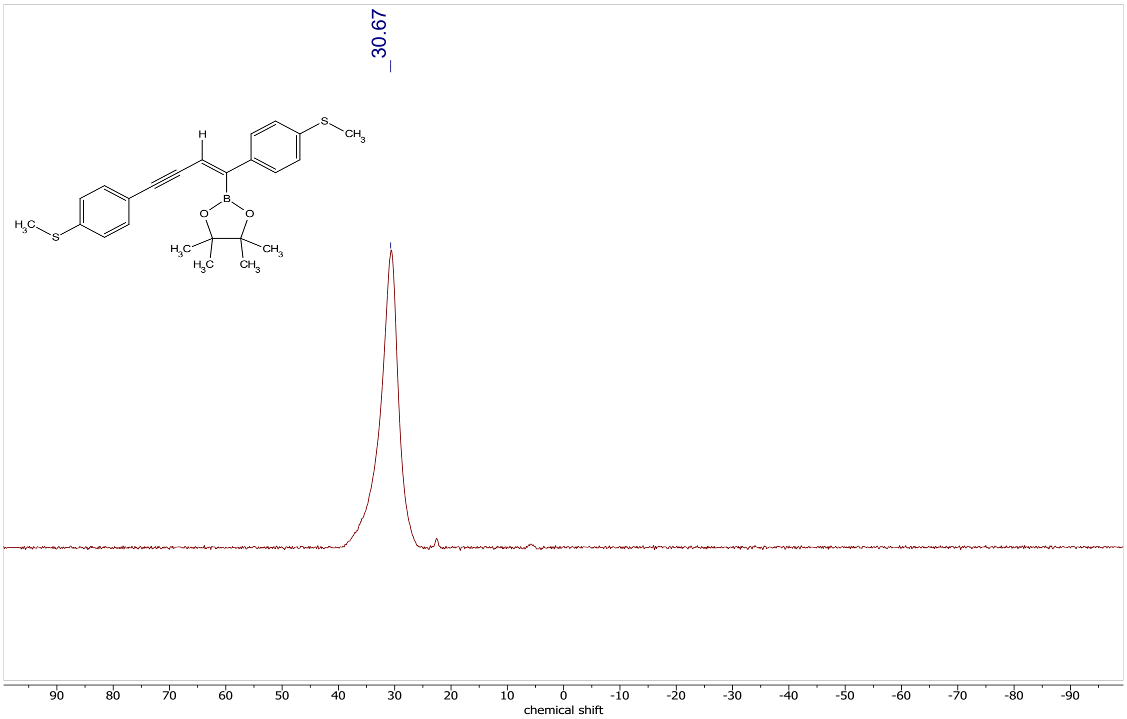
30.67
90 80 70 60 50 40 30 20 10 0 -10 -20 -30 -40 -50 -60 -70 -80 -90
chemical shift

2k1-^{1}H (crude NMR)

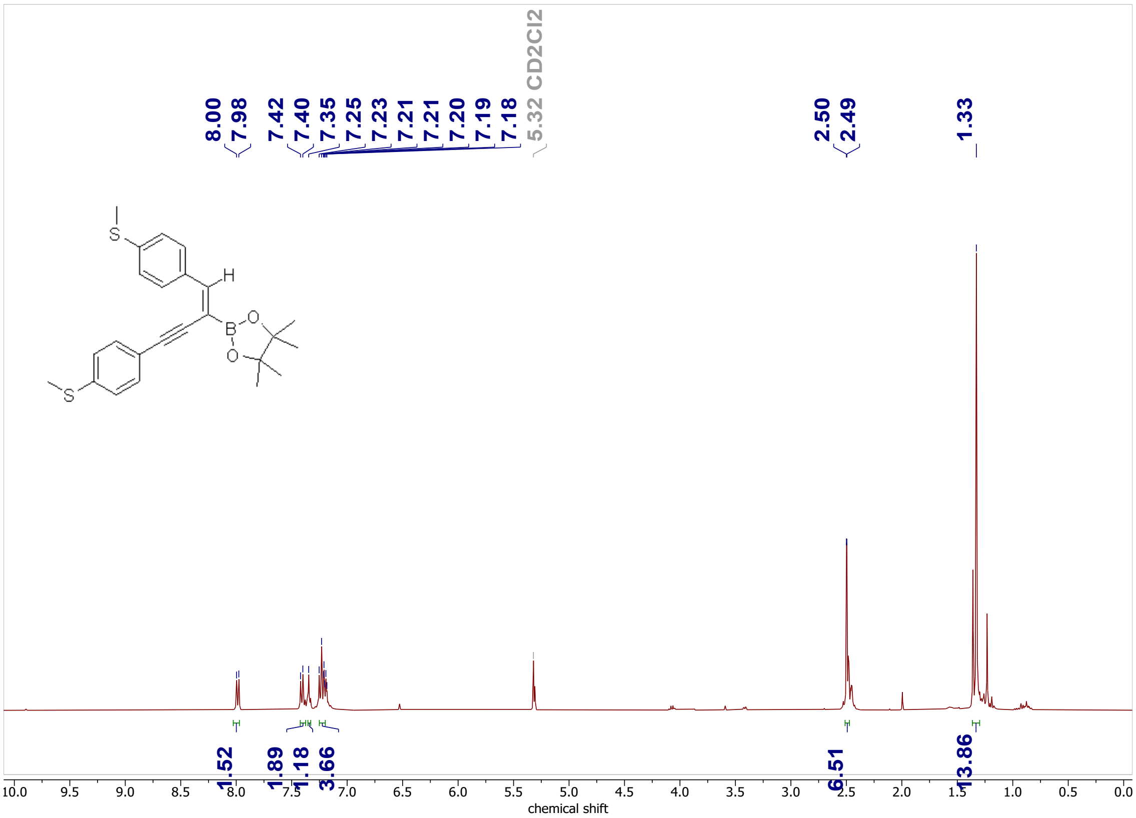

2l-^{1}H (crude NMR)

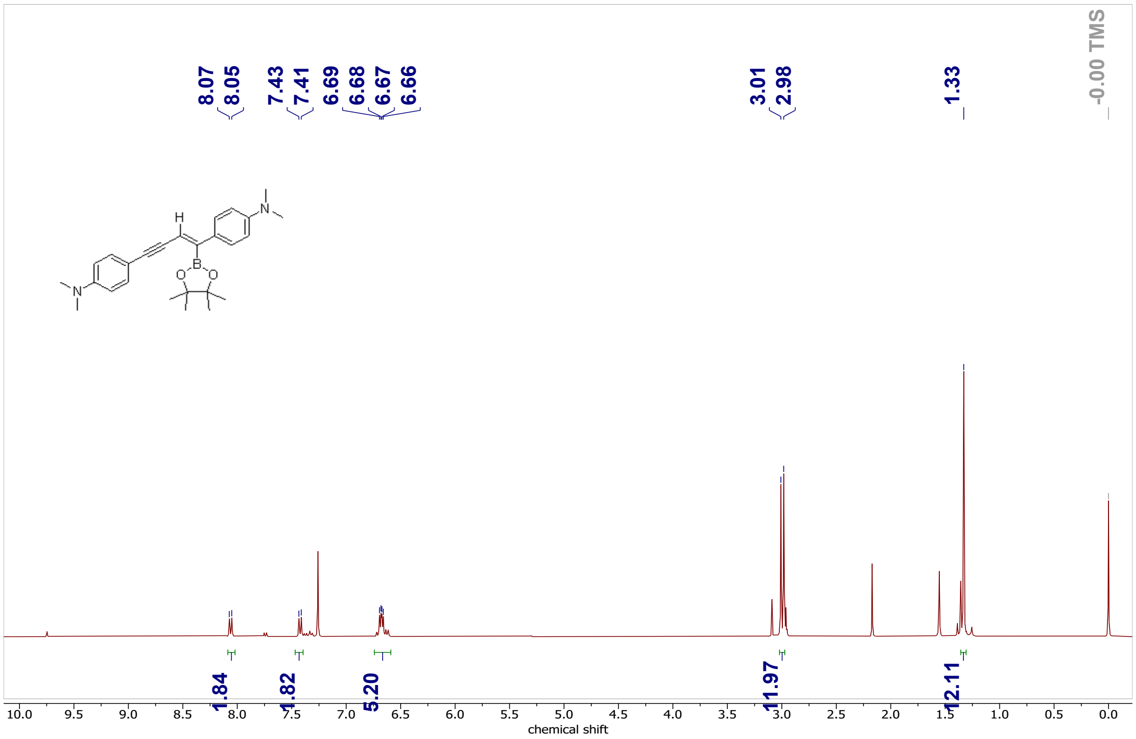

2l-^{11}B

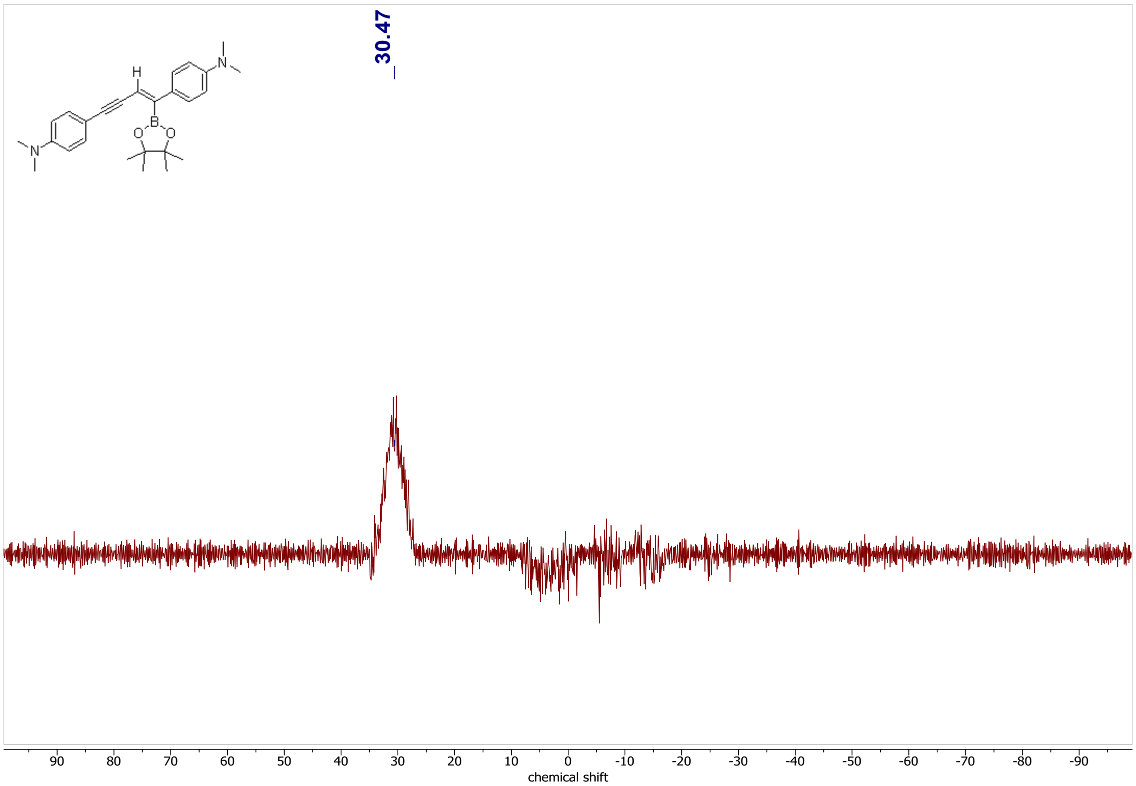
30.47
chemical shift

2m-^{1}H

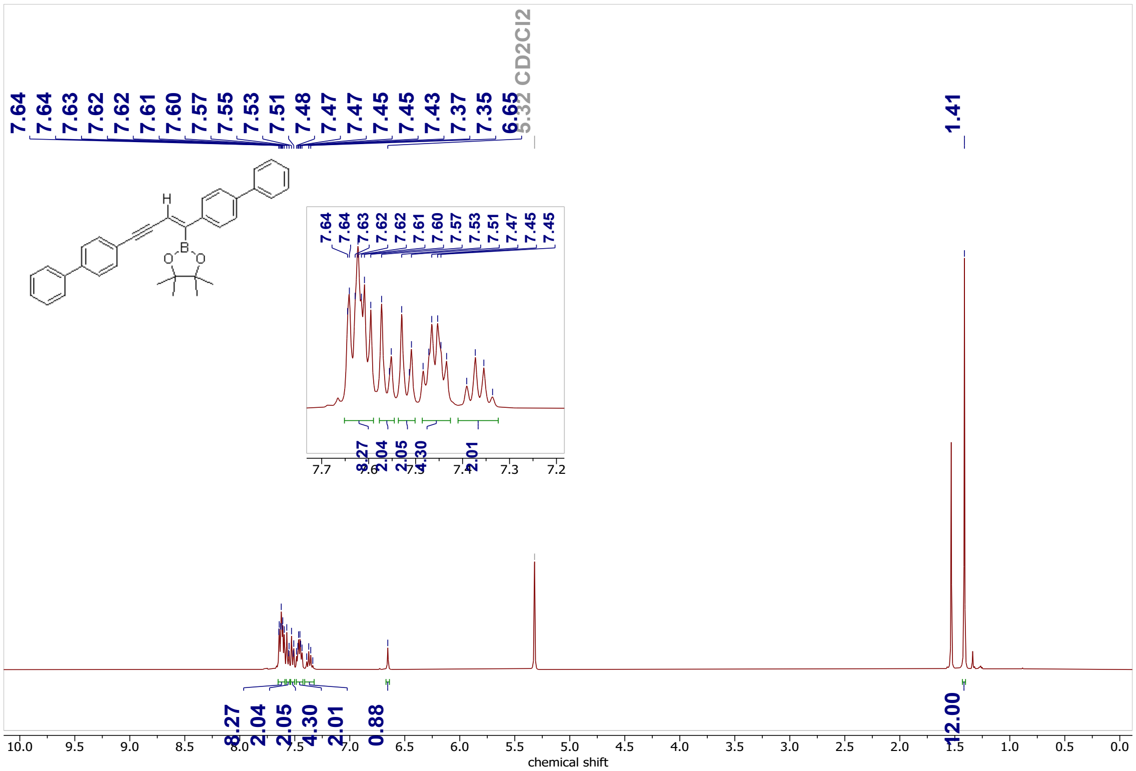
7.64
7.64
7.63
7.62
7.62
7.61
7.60
7.57
7.55
7.53
7.51
7.48
7.47
7.47
7.45
7.45
7.43
7.37
7.35
6.65
5.32 CD2Cl2
1.41
8.27
2.04
2.05
4.30
2.01
0.88
12.00
chemical shift

2m-^{13}C

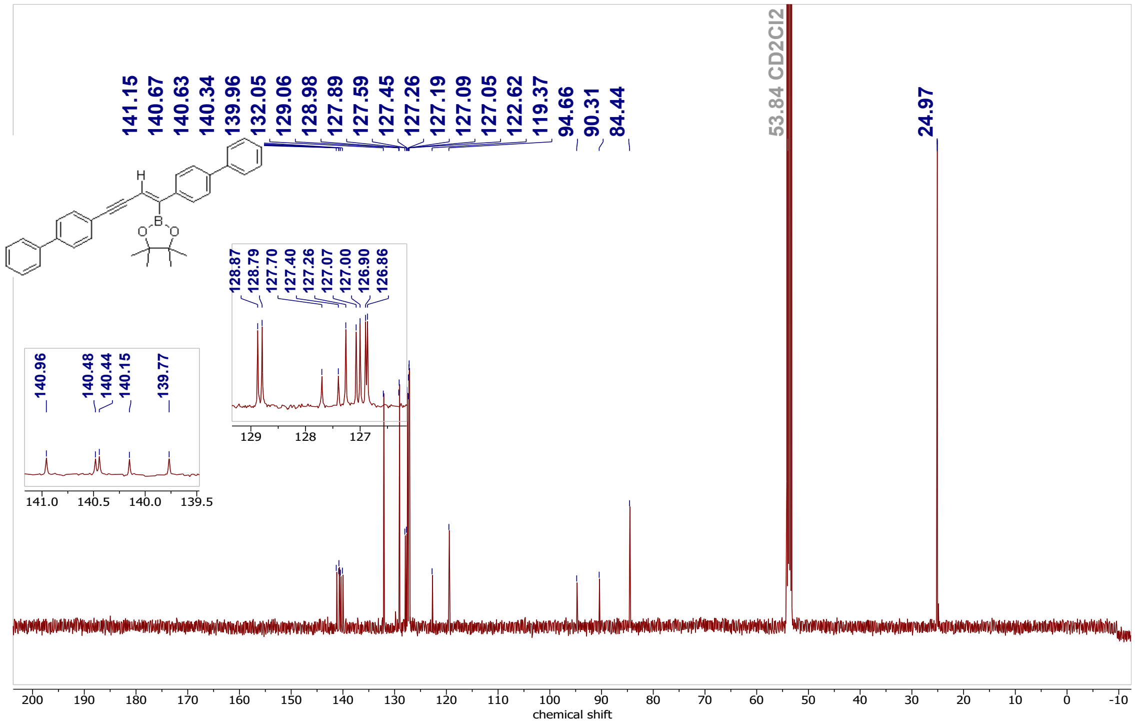

141.15
140.67
140.63
140.34
139.96
132.05
129.06
128.98
127.89
127.59
127.45
127.26
127.19
127.09
127.05
122.62
119.37
94.66
90.31
84.44
53.84 CD2Cl2
24.97
140.96
140.48
140.44
140.15
139.77
128.87
128.79
127.70
127.40
127.26
127.07
127.00
126.90
126.86
chemical shift

2m-^{11}B

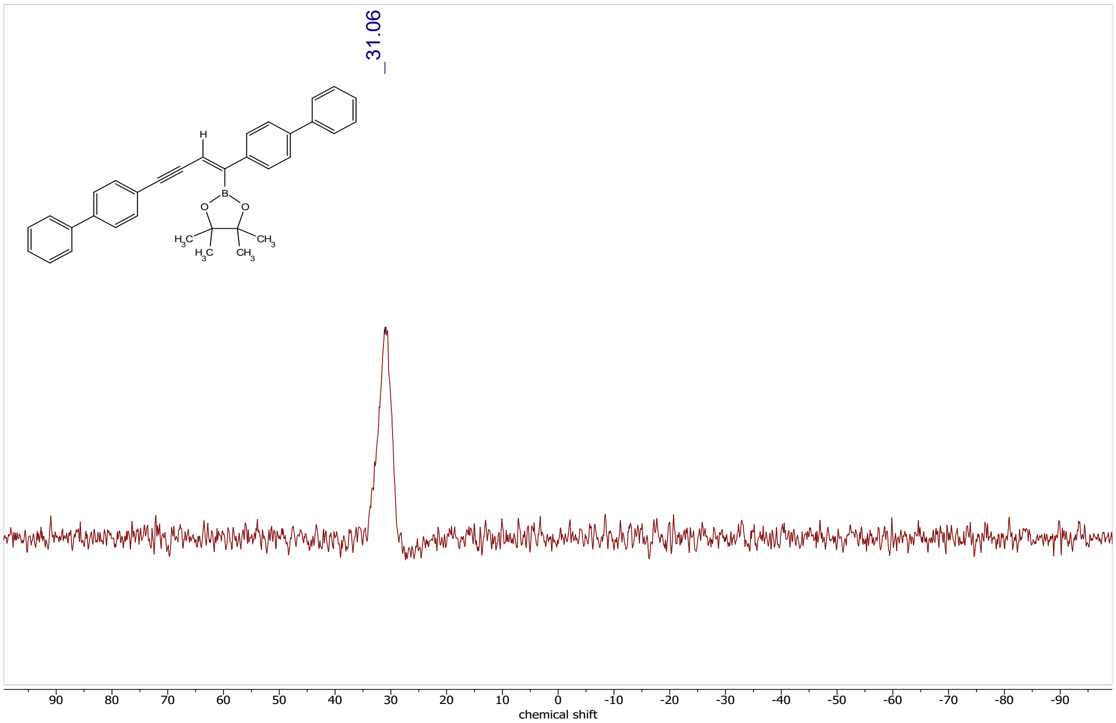

31.06
chemical shift

2n-^{1}H

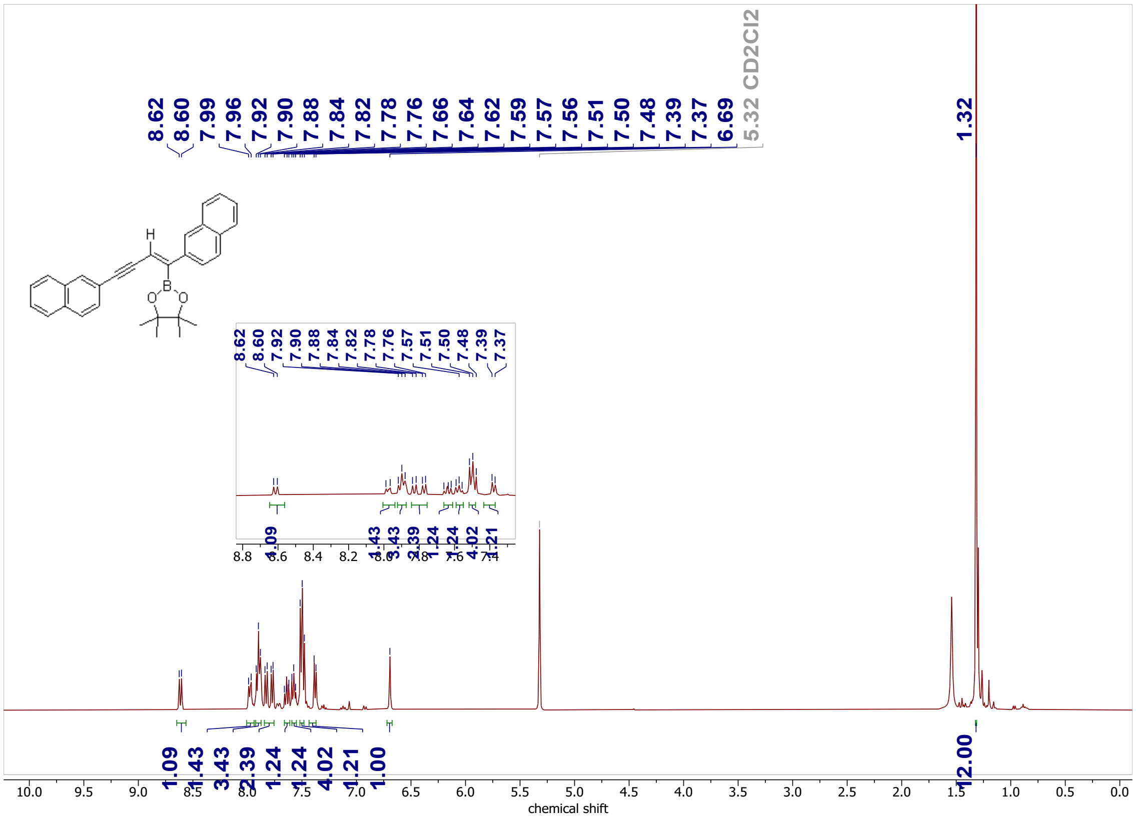

2n-^{13}C

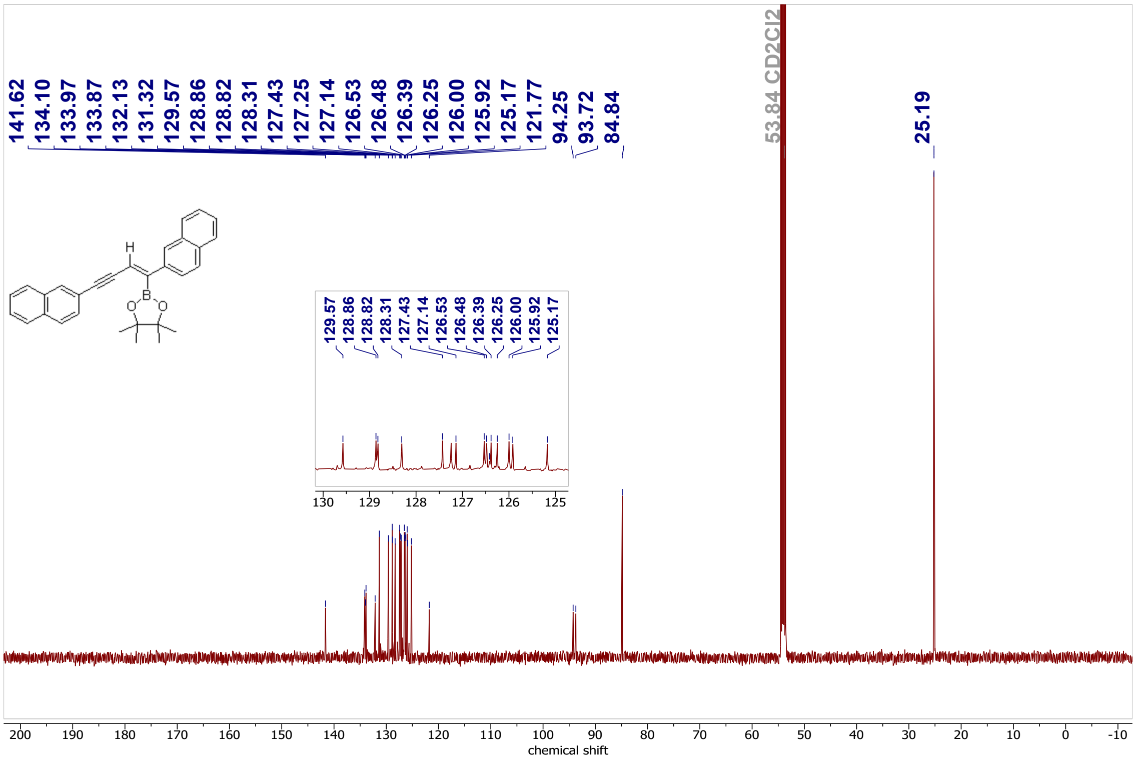

2n-^{11}B

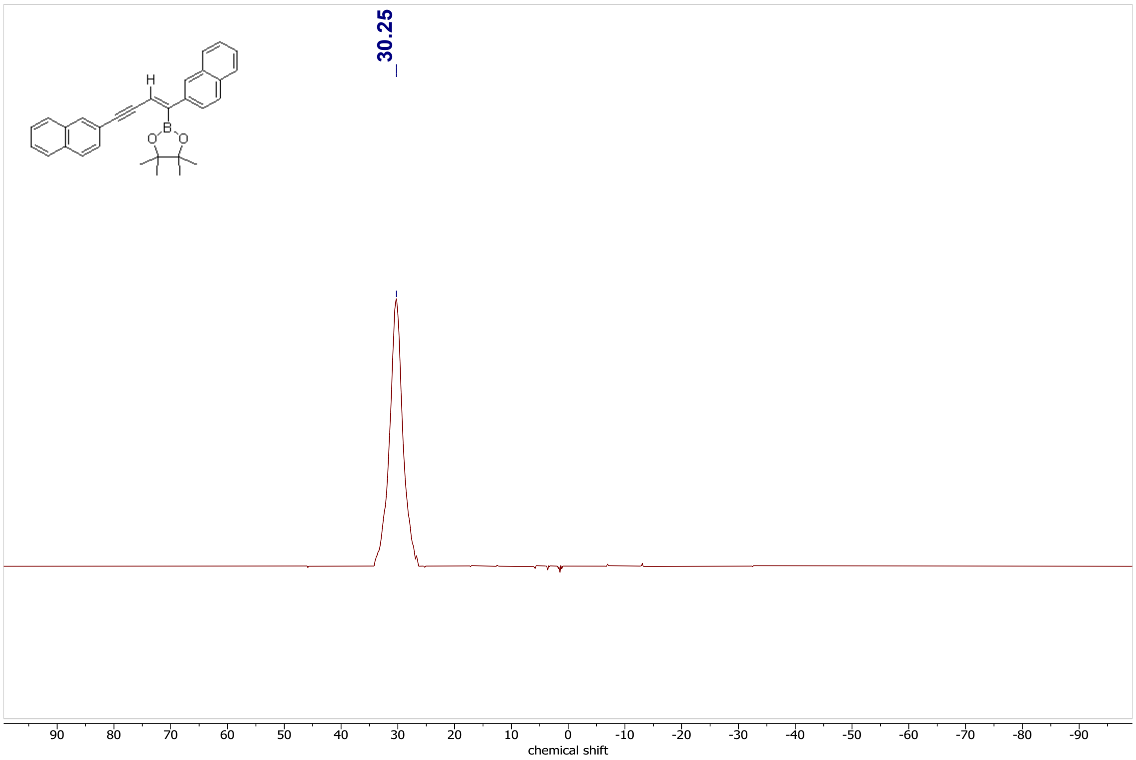

30.25
H
B
O
O
chemical shift

2o-^{1}H

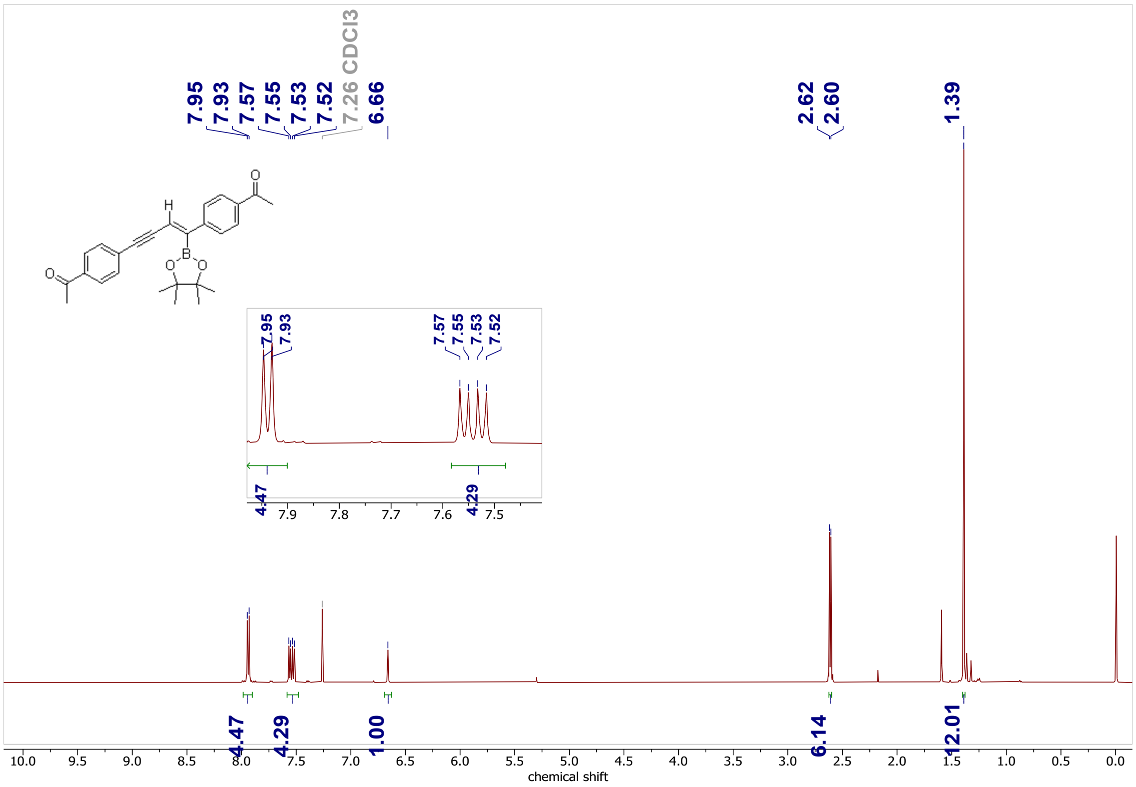

7.95
7.93
7.57
7.55
7.53
7.52
7.26 CDCl3
6.66
2.62
2.60
1.39
H
O
B
O
O
O
4.47
4.29
1.00
6.14
12.01
chemical shift

2o-^{13}C

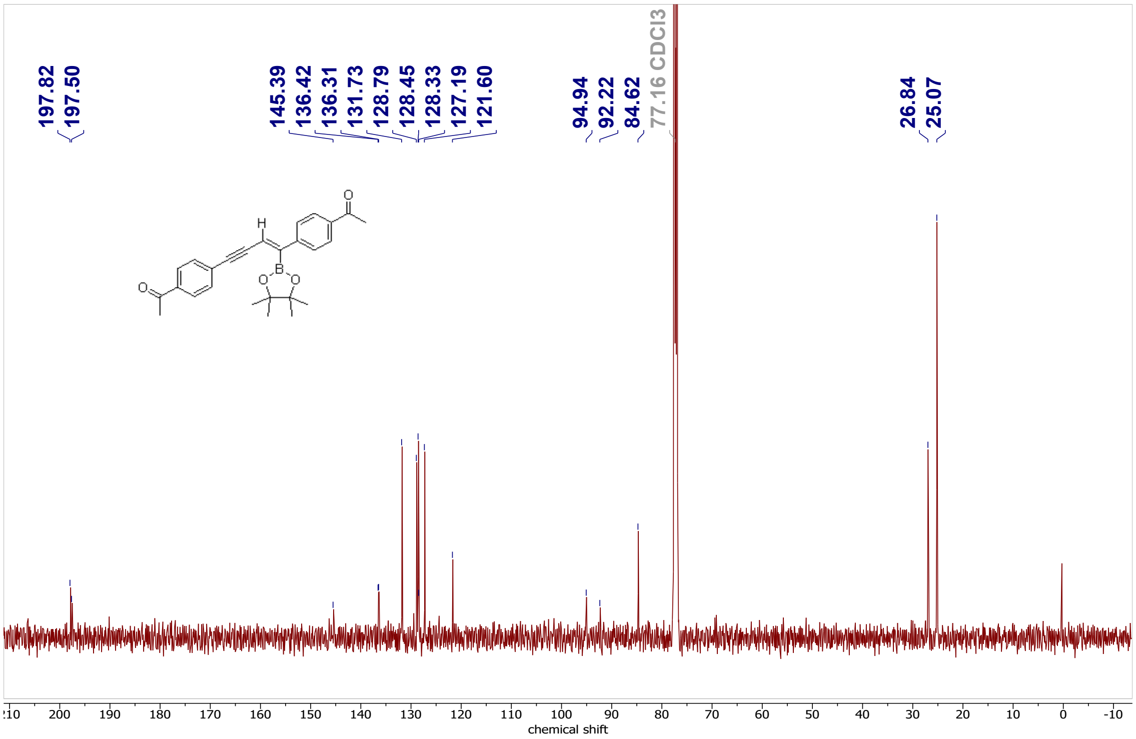
197.82
197.50
145.39
136.42
136.31
131.73
128.79
128.45
128.33
127.19
121.60
94.94
92.22
84.62
77.16 CDCl3
26.84
25.07
chemical shift

2o-^{11}B

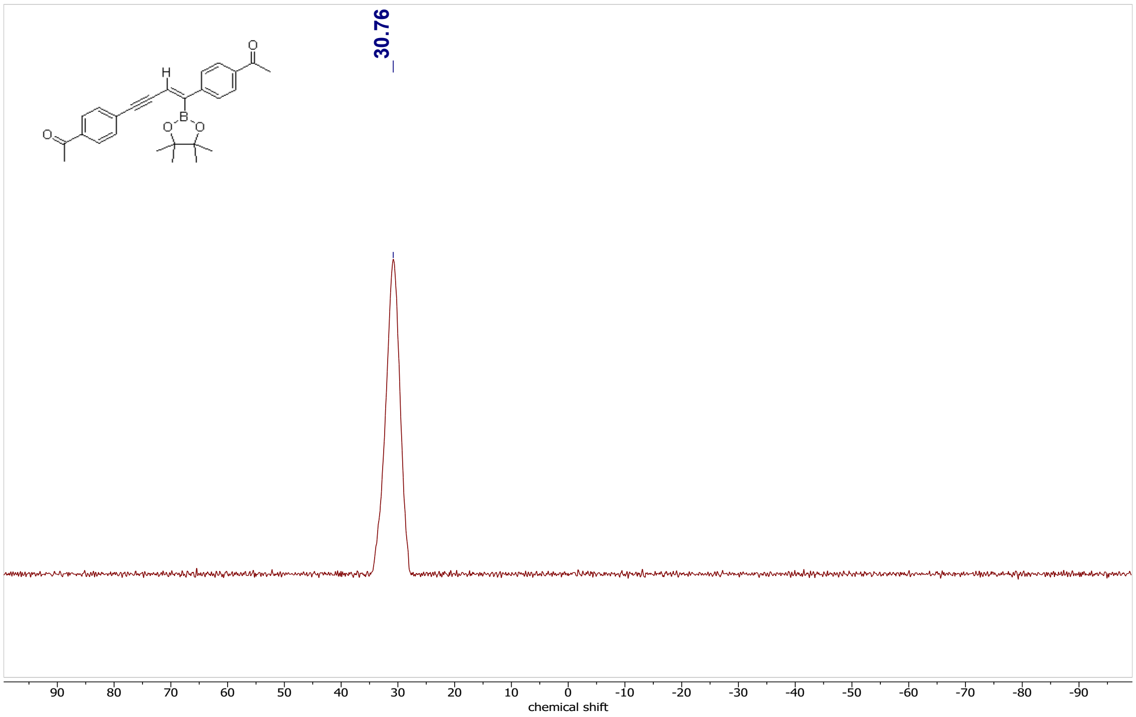
30.76
chemical shift

2p-^{1}H-contaminated with 10% *Z* isomer

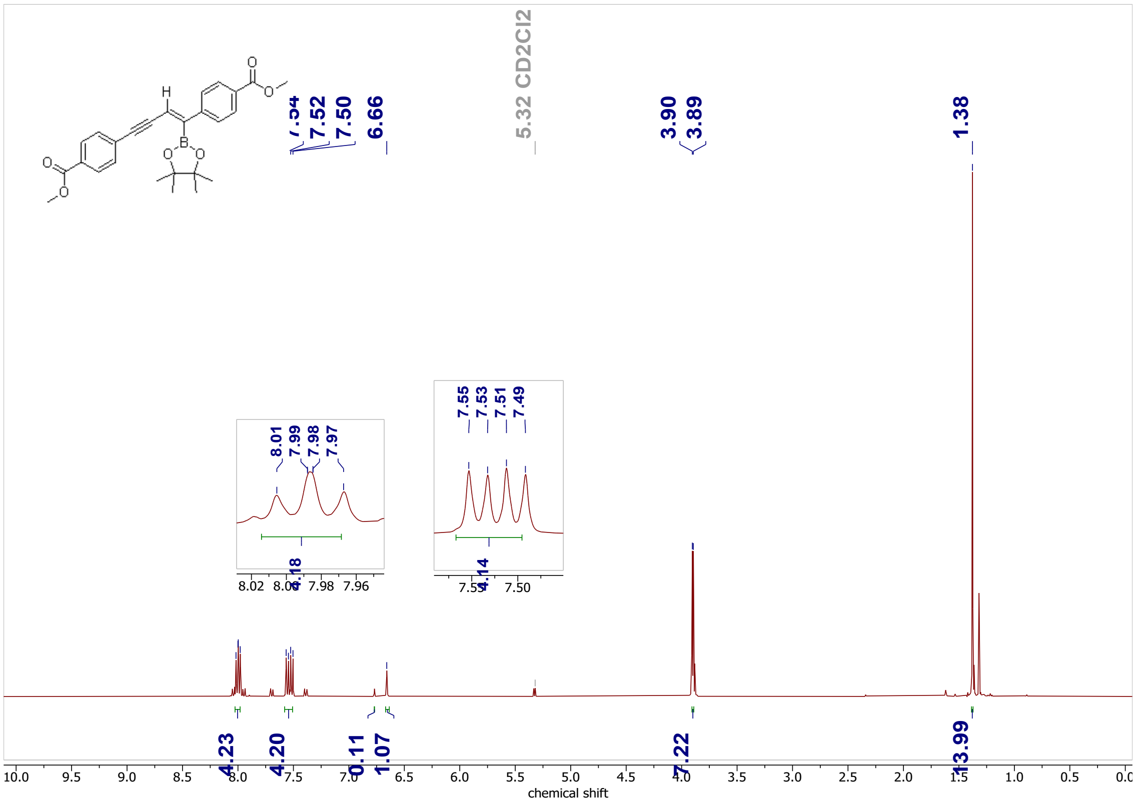

2p-^{13}C

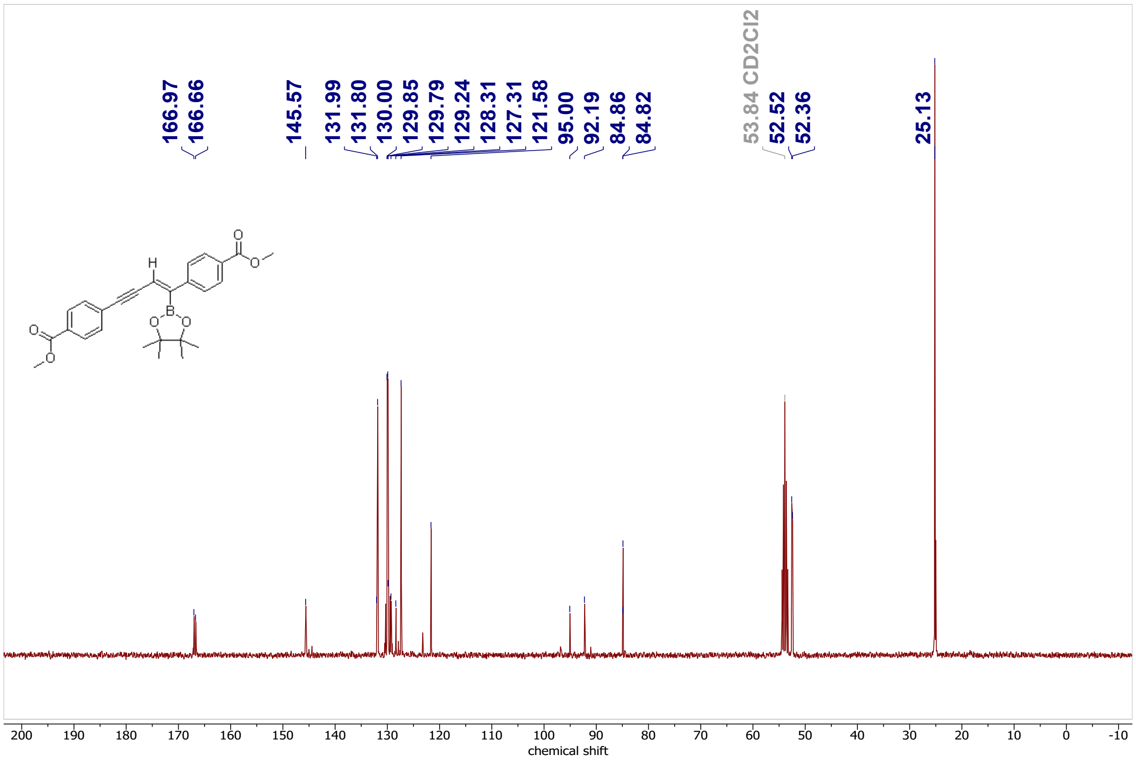

2p-^{11}B

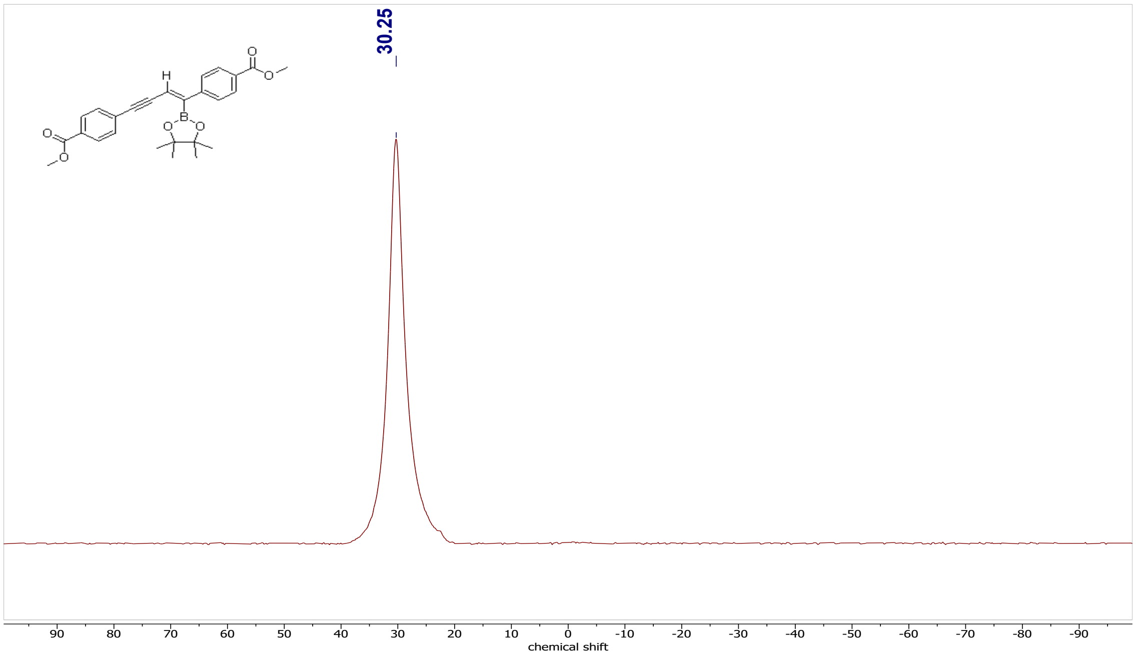

30.25
chemical shift

2q-^{1}H

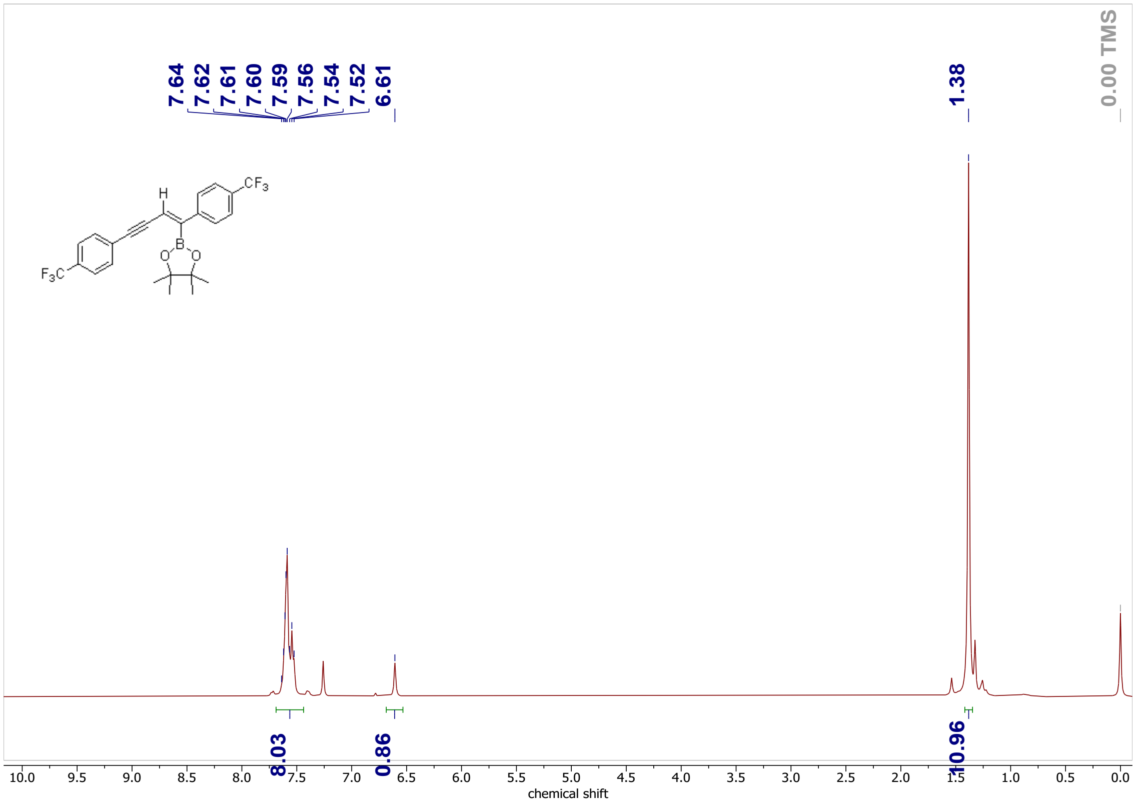

7.64
7.62
7.61
7.60
7.59
7.56
7.54
7.52
6.61
1.38
0.00 TMS
8.03
0.86
10.96
chemical shift

2q-^{13}C

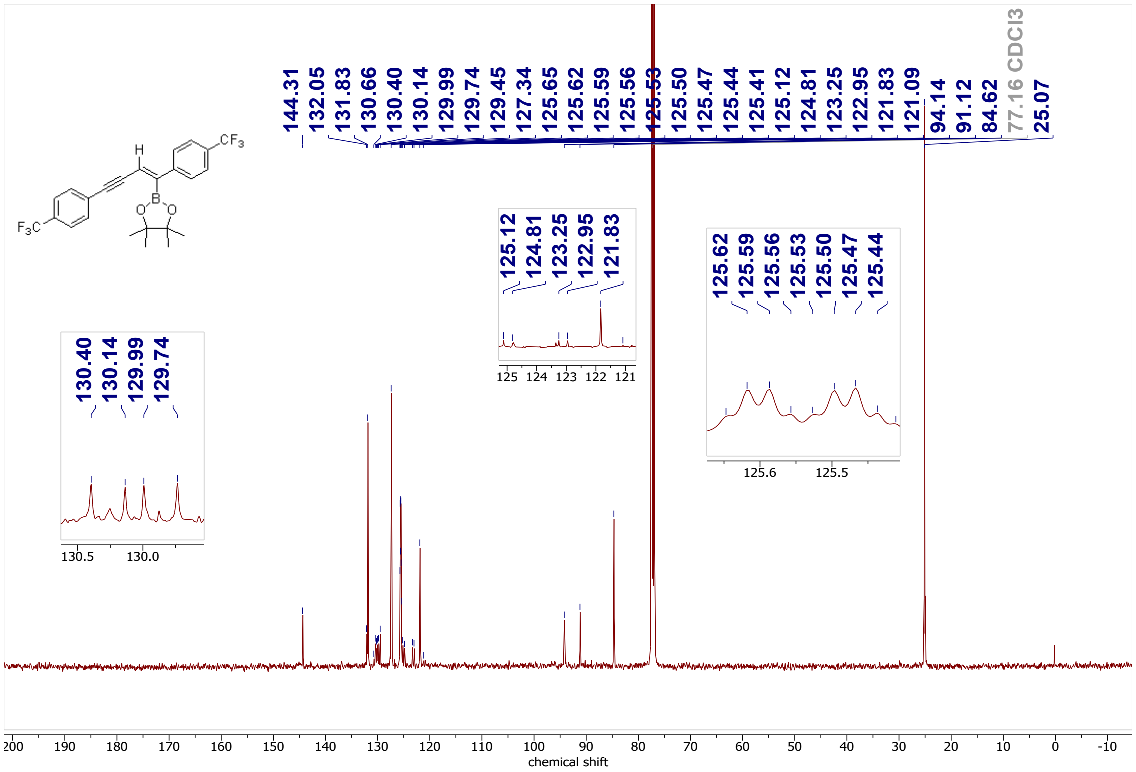
144.31
132.05
131.83
130.66
130.40
130.14
129.99
129.74
129.45
127.34
125.65
125.62
125.59
125.56
125.53
125.50
125.47
125.44
125.41
125.12
124.81
123.25
122.95
121.83
121.09
94.14
91.12
84.62
77.16 CDCl3
25.07
F3C
CF3
H
B
O
chemical shift

2q-^{11}B

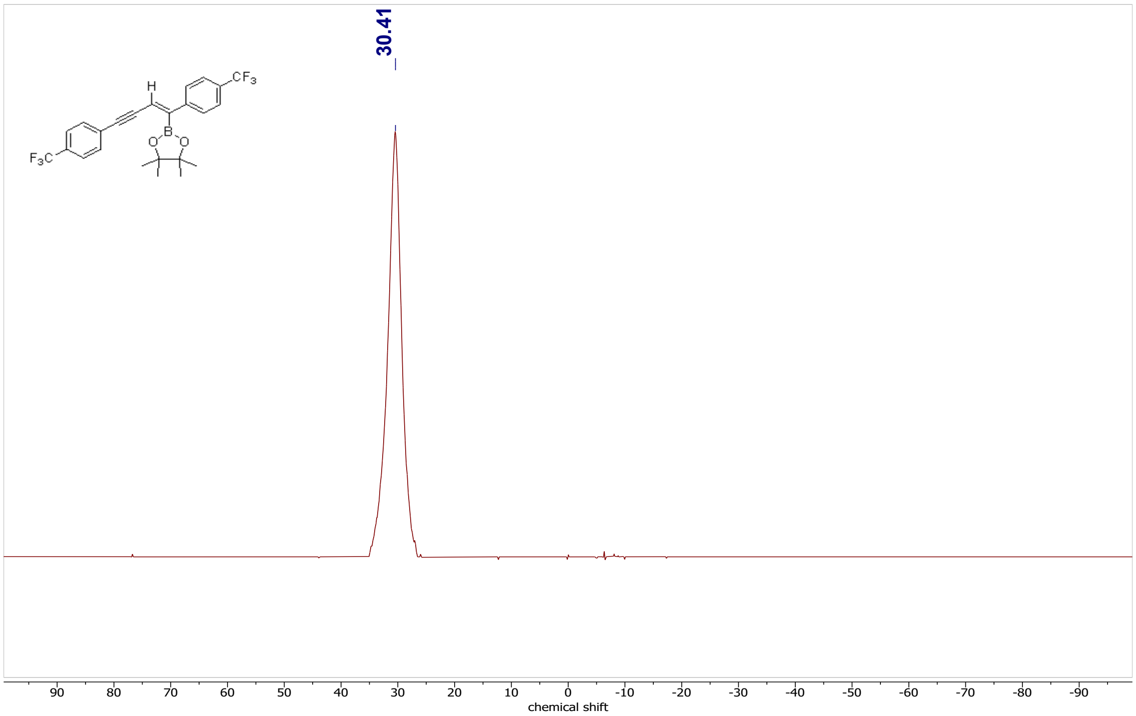
30.41
F3C
CF3
H
B
O
chemical shift

2q-^{19}F

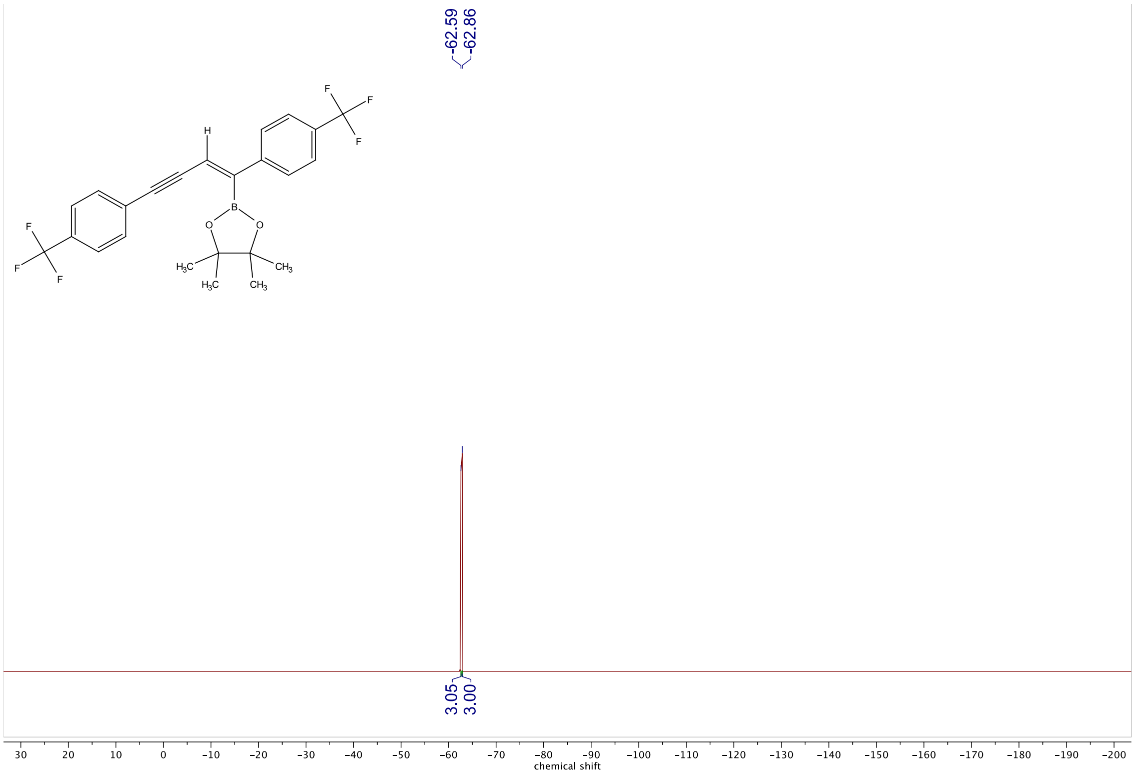

-62.59
-62.86
3.05
3.00
30 20 10 0 −10 −20 −30 −40 −50 −60 −70 −80 −90 −100 −110 −120 −130 −140 −150 −160 −170 −180 −190 −200
chemical shift

2r-^{1}H

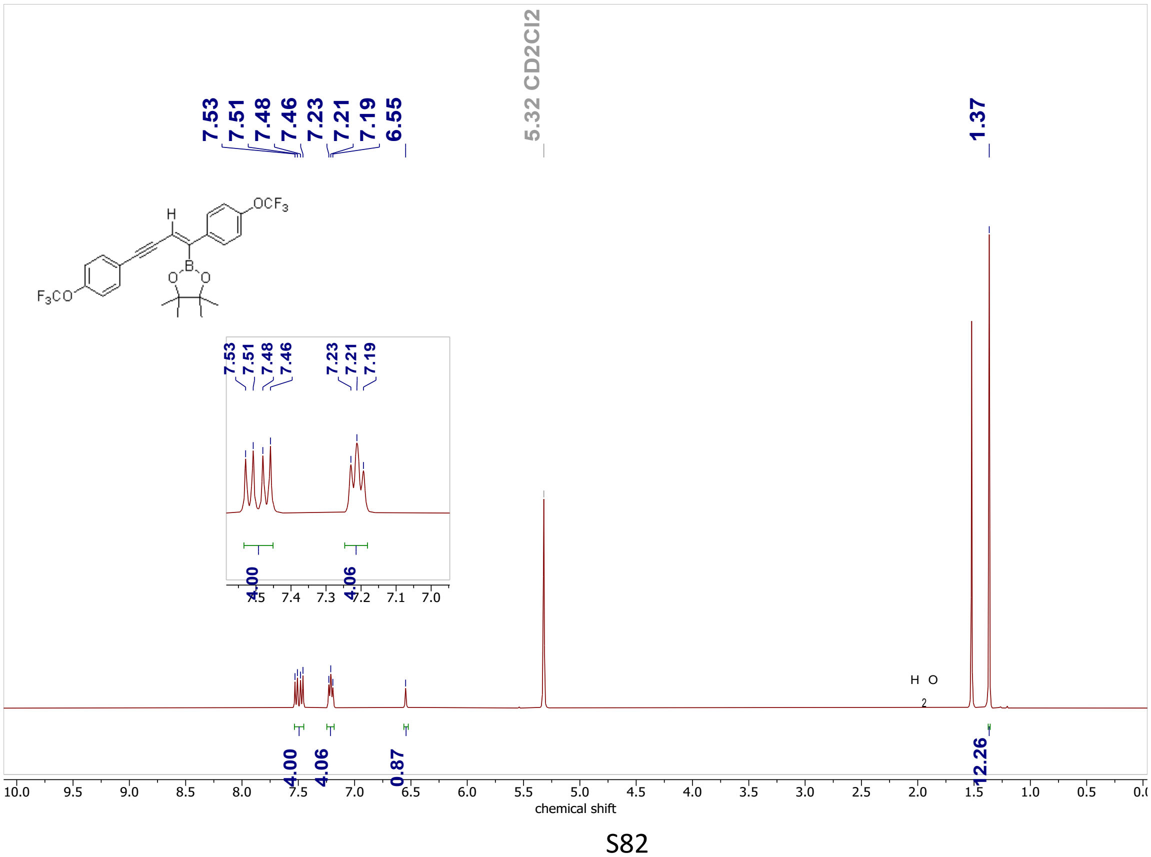

7.53
7.51
7.48
7.46
7.23
7.21
7.19
6.55
5.32 CD2Cl2
1.37
OCF3
F3CO
H O
2
4.00
4.06
0.87
12.26
10.0 9.5 9.0 8.5 8.0 7.5 7.0 6.5 6.0 5.5 5.0 4.5 4.0 3.5 3.0 2.5 2.0 1.5 1.0 0.5
chemical shift

2r-^{13}C

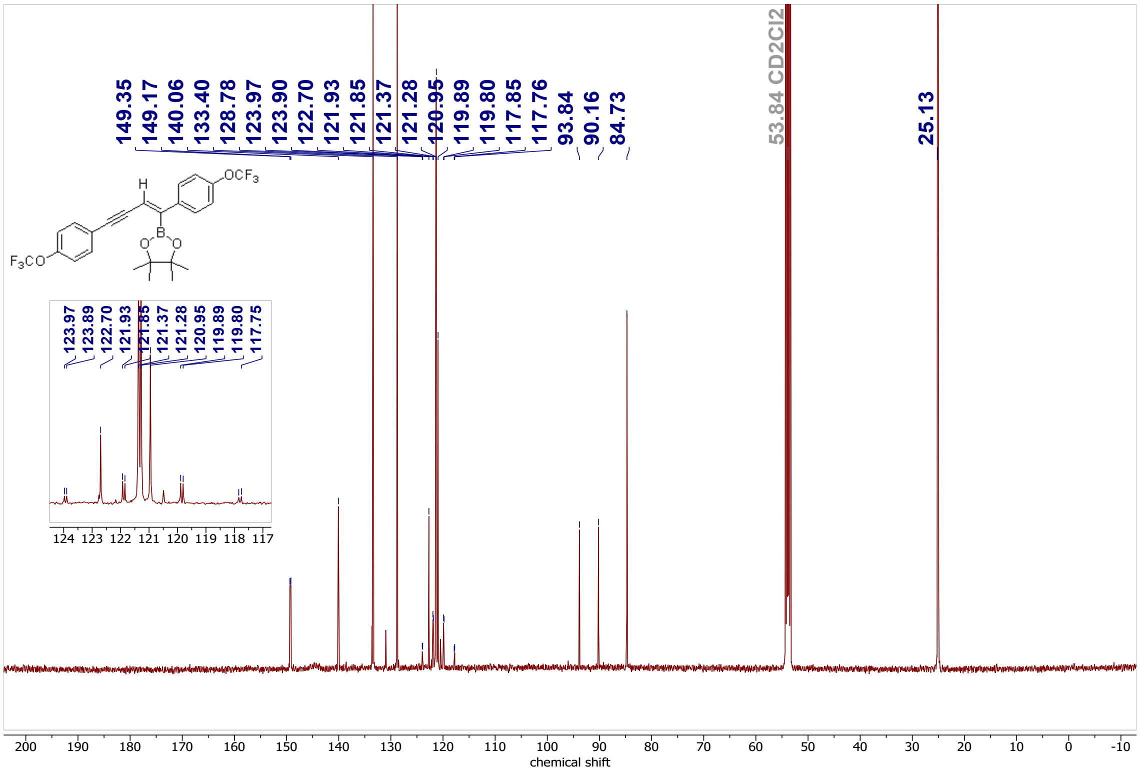

149.35
149.17
140.06
133.40
128.78
123.97
123.90
122.70
121.93
121.85
121.37
121.28
120.95
119.89
119.80
117.85
117.76
93.84
90.16
84.73
53.84 CD2Cl2
25.13
OCF3
H
B
O
F3CO
123.97
123.89
122.70
121.93
121.85
121.37
121.28
120.95
119.89
119.80
117.75
124 123 122 121 120 119 118 117
200 190 180 170 160 150 140 130 120 110 100 90 80 70 60 50 40 30 20 10 0 -10
chemical shift

2r-^{11}B

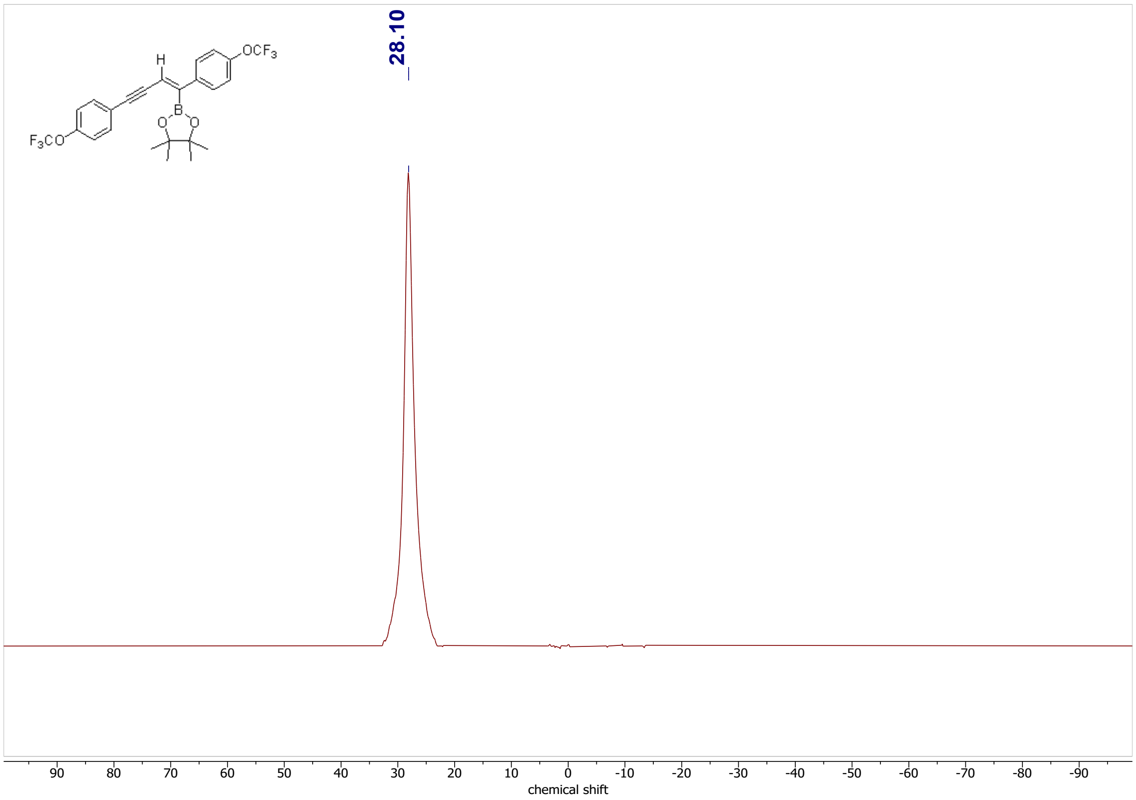

28.10
OCF3
H
B
O
F3CO
90 80 70 60 50 40 30 20 10 0 -10 -20 -30 -40 -50 -60 -70 -80 -90
chemical shift

2r-^{19}F

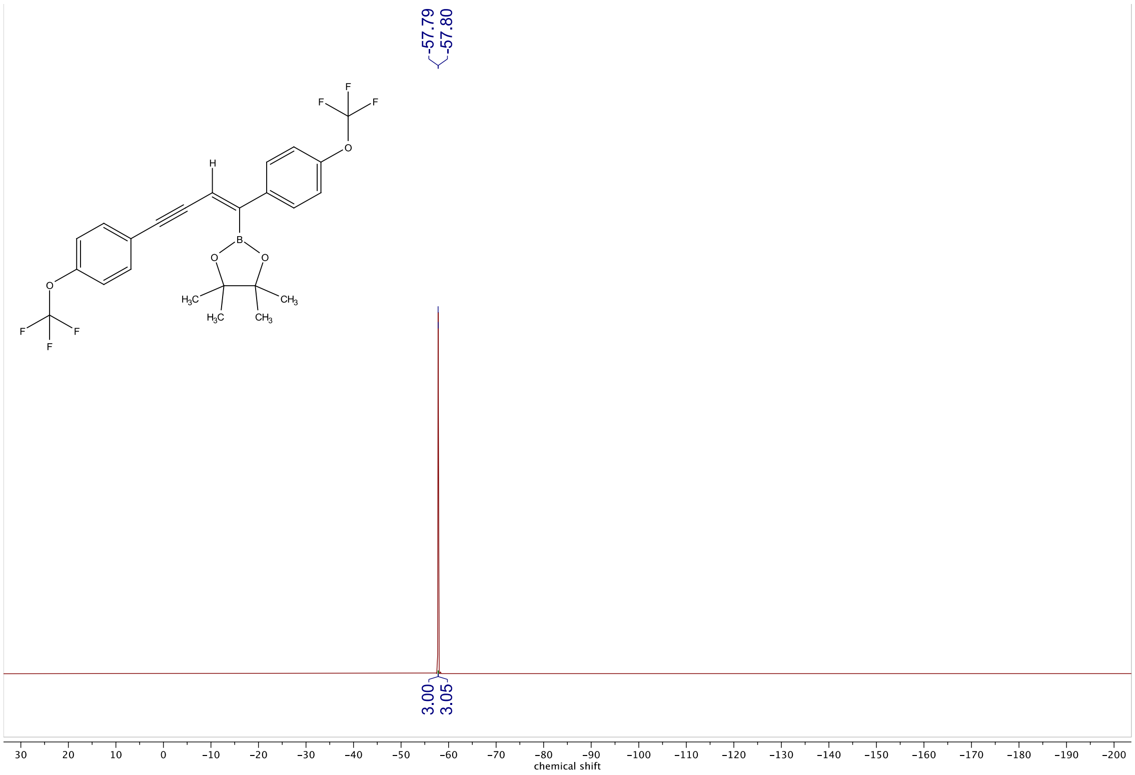

2s-^{1}H

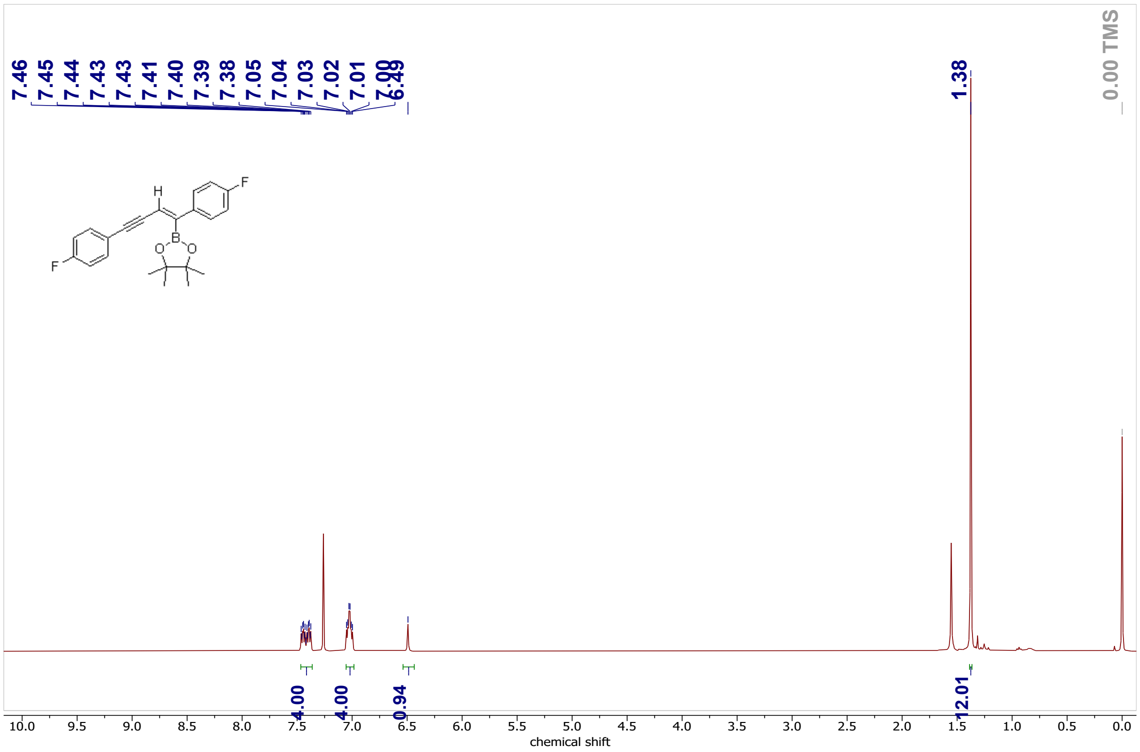

2s-^{13}C

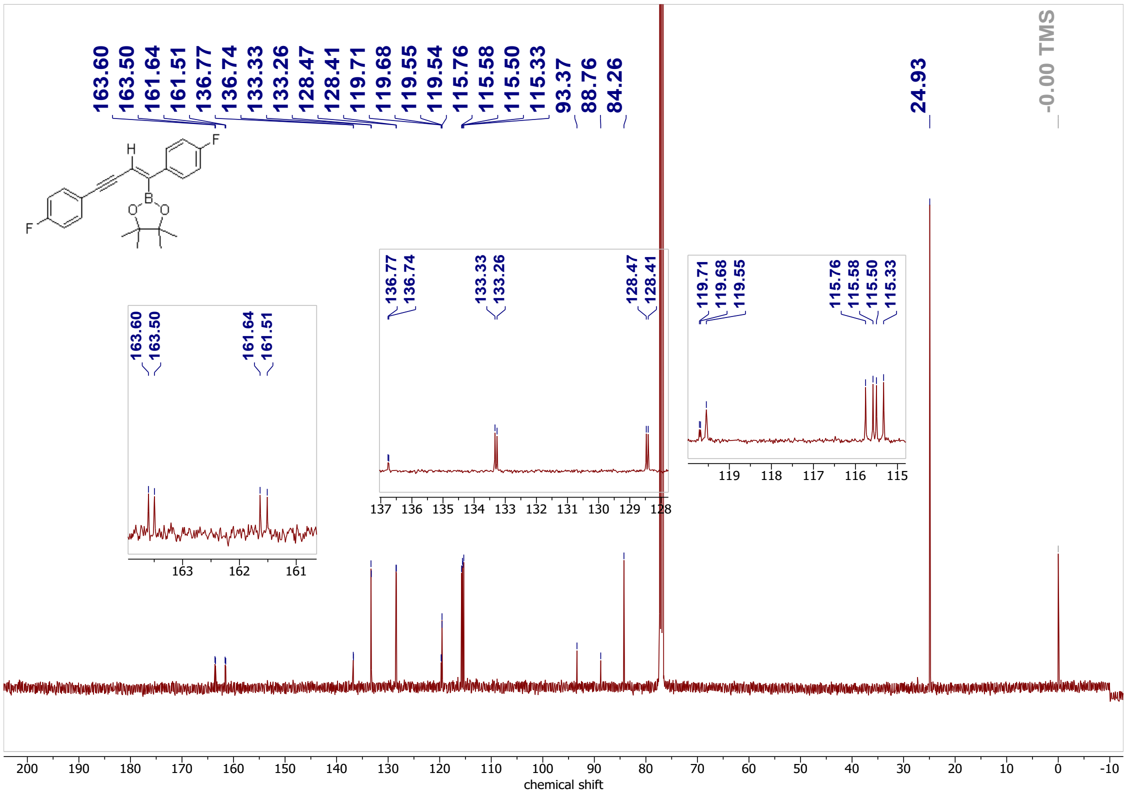

2s-^{11}B

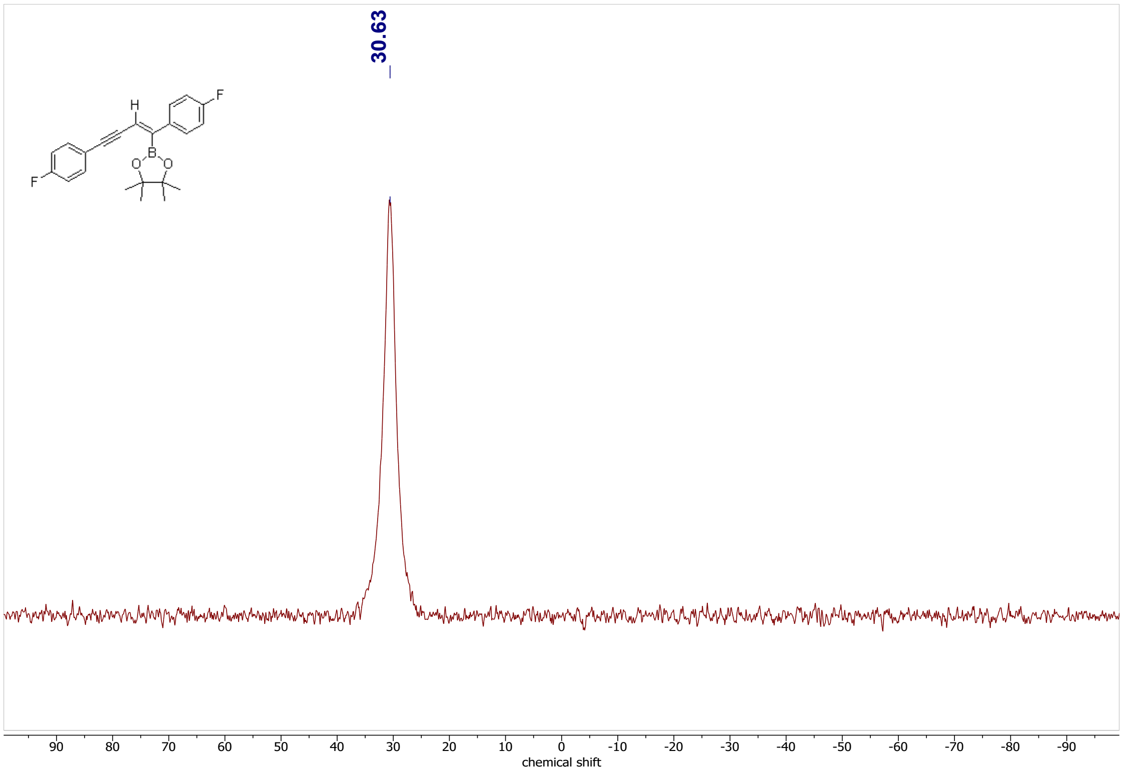

2s-^{19}F

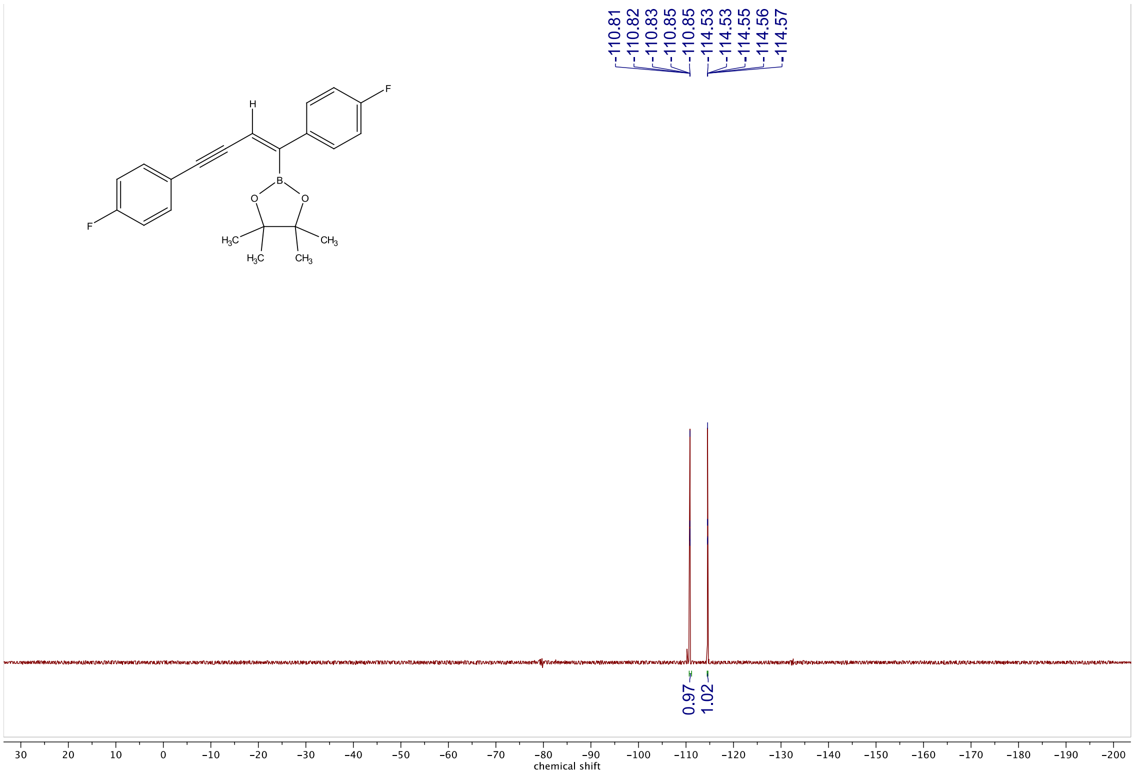

-110.81
-110.82
-110.83
-110.85
-110.85
-114.53
-114.53
-114.55
-114.56
-114.57
F
H
B
O
O
F
H_3C
H_3C
CH_3
CH_3
0.97
1.02
chemical shift

2t-^{1}H

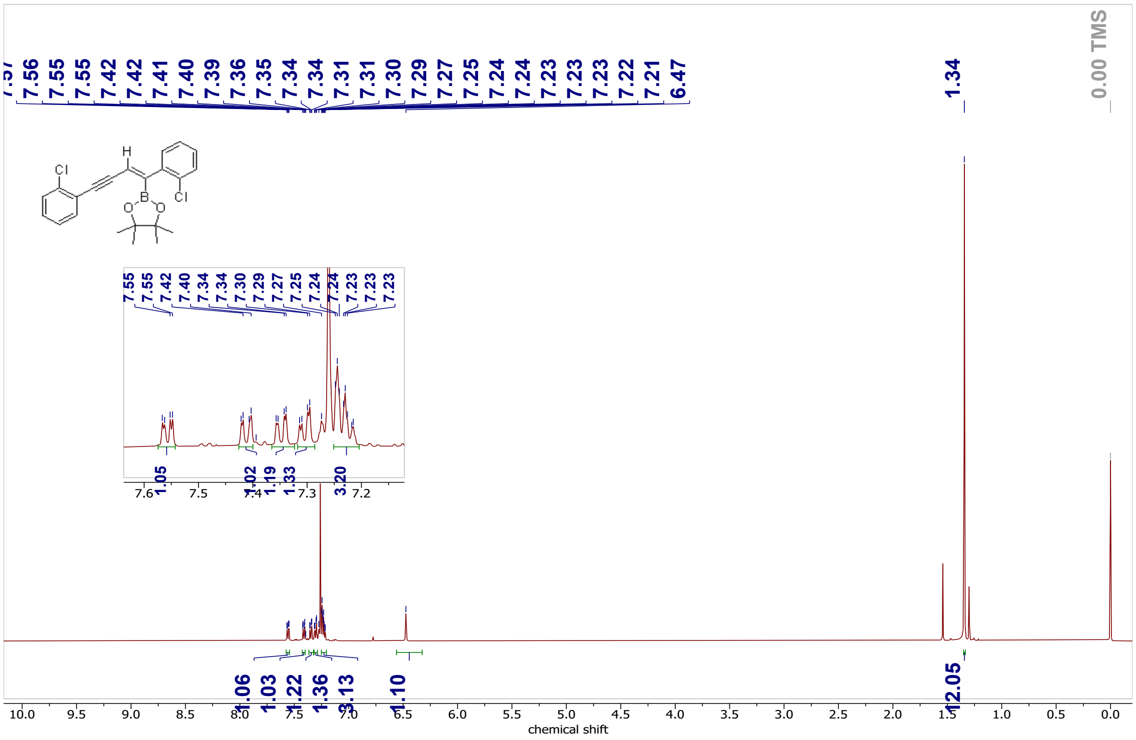

7.56
7.55
7.55
7.42
7.42
7.41
7.40
7.39
7.36
7.35
7.34
7.34
7.31
7.31
7.30
7.29
7.27
7.25
7.24
7.24
7.23
7.23
7.23
7.22
7.21
6.47
1.34
0.00 TMS
Cl
H
Cl
B
O
O
1.05
1.02
1.19
1.33
3.20
1.06
1.03
1.22
1.36
3.13
1.10
12.05
chemical shift

2t-^{13}C

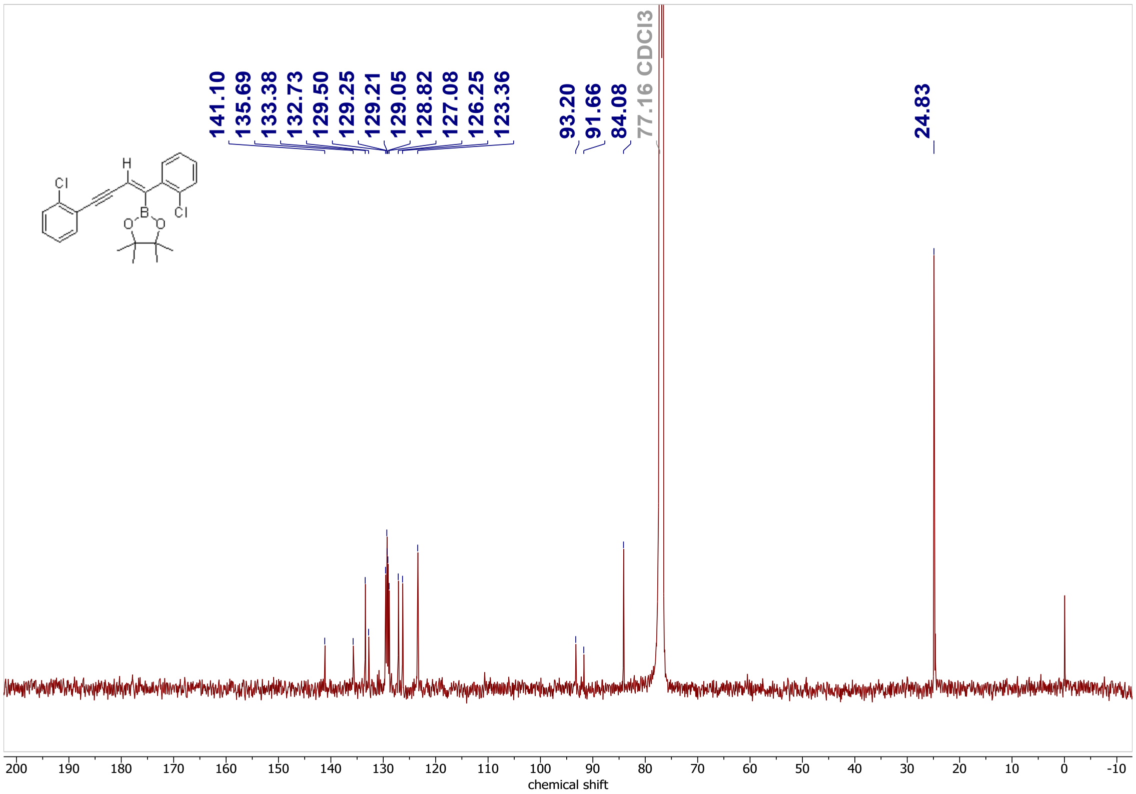
141.10
135.69
133.38
132.73
129.50
129.25
129.21
129.05
128.82
127.08
126.25
123.36
93.20
91.66
84.08
77.16 CDCl3
24.83
Cl
H
Cl
O
B
O
200
190
180
170
160
150
140
130
120
110
100
90
80
70
60
50
40
30
20
10
0
-10
chemical shift

2t-^{11}B

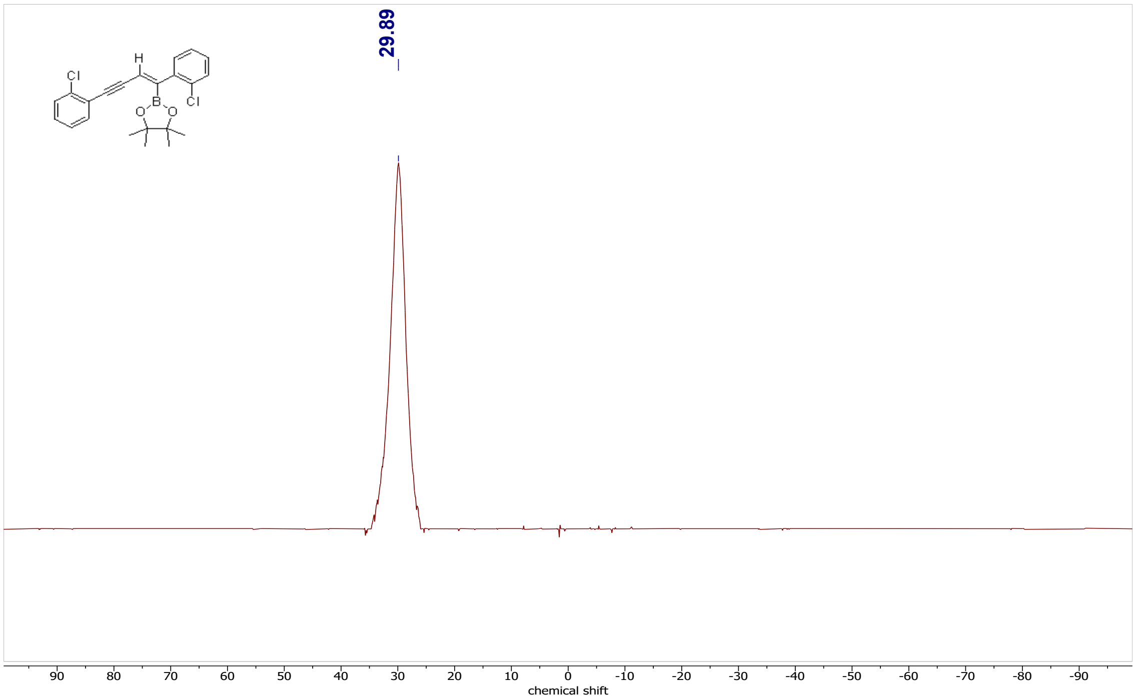
29.89
Cl
H
Cl
O
B
O
90
80
70
60
50
40
30
20
10
0
-10
-20
-30
-40
-50
-60
-70
-80
-90
chemical shift

2u-^{1}H

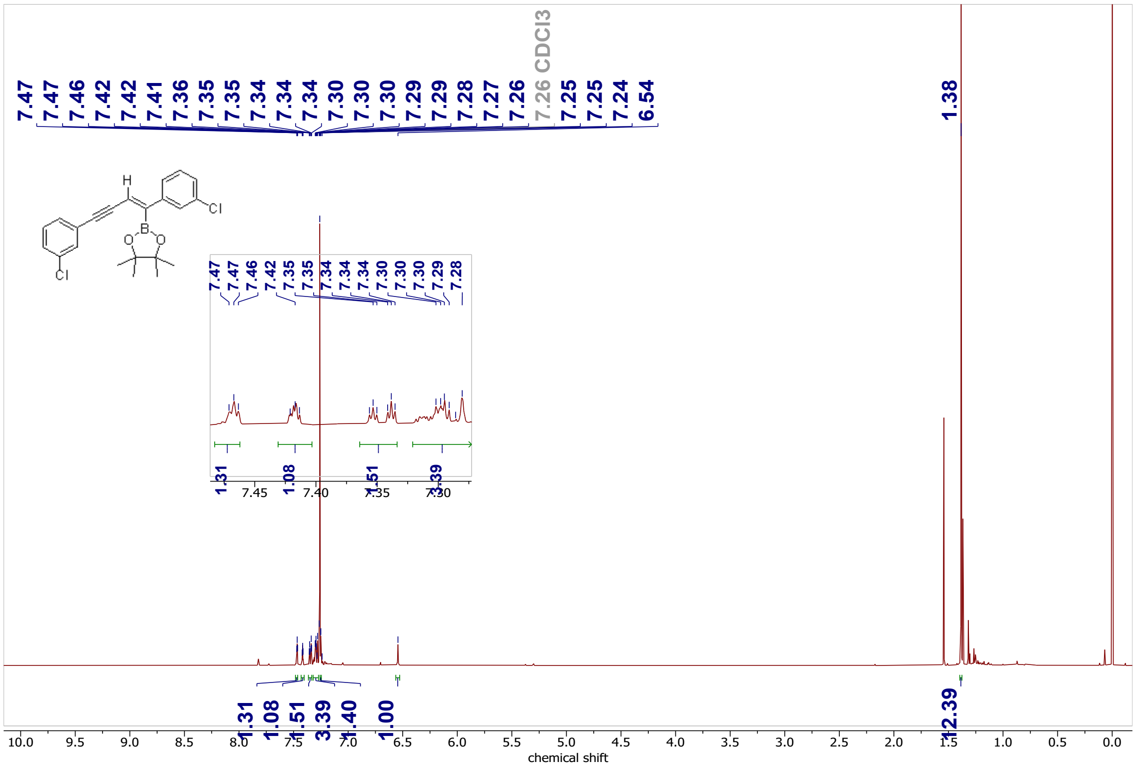
7.47
7.47
7.46
7.42
7.42
7.41
7.36
7.35
7.35
7.34
7.34
7.34
7.30
7.30
7.30
7.29
7.29
7.28
7.27
7.26
7.26 CDCl3
7.25
7.25
7.24
6.54
1.38
H
Cl
B
O
O
Cl
1.31
1.08
1.51
3.39
1.40
1.00
12.39
7.45
7.40
7.35
7.30
chemical shift

2u-^{13}C

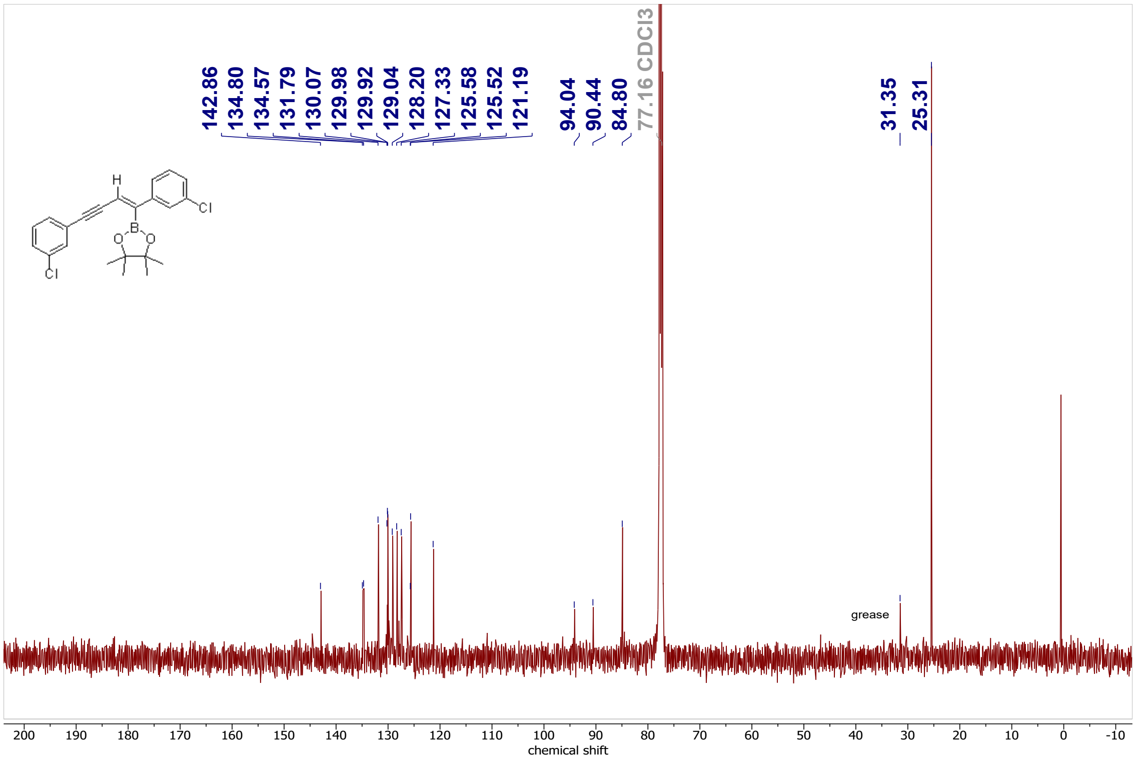
142.86
134.80
134.57
131.79
130.07
129.98
129.92
129.04
128.20
127.33
125.58
125.52
121.19
94.04
90.44
84.80
77.16 CDCl3
31.35
25.31
H
Cl
B
O
O
Cl
grease
chemical shift

2t-^{11}B

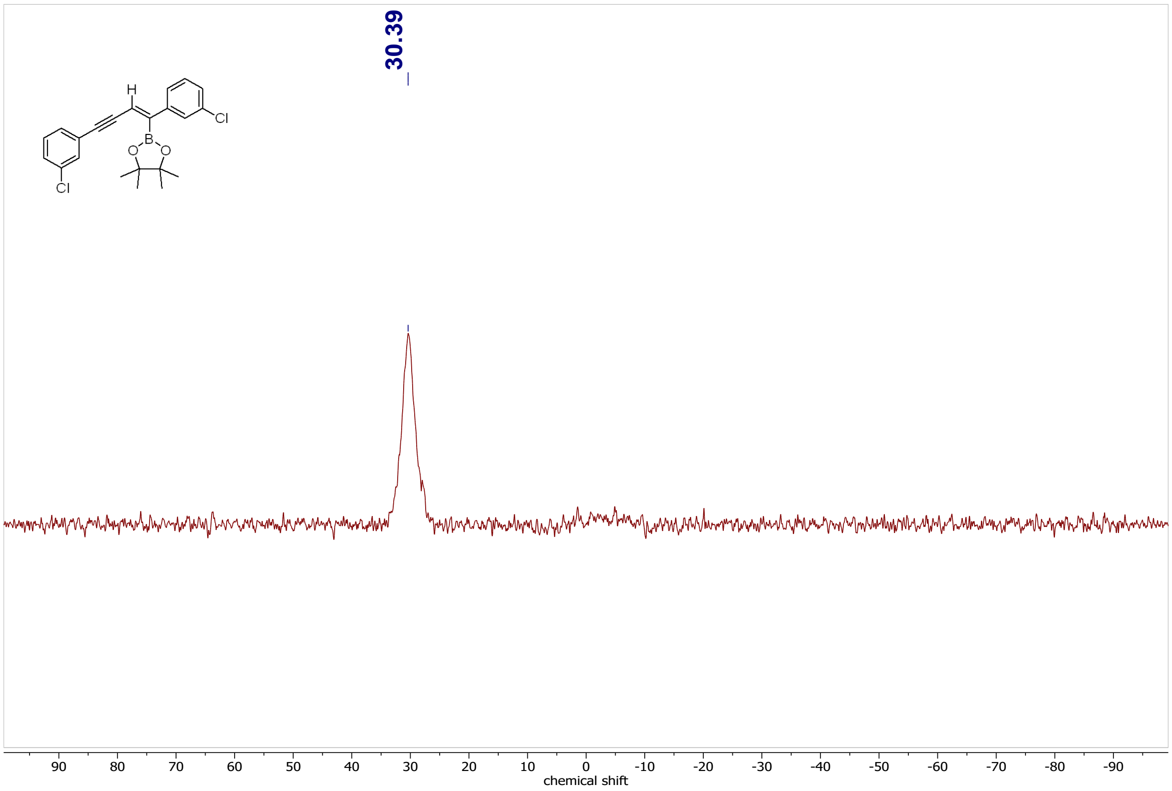
30.39
H
Cl
Cl
B
O
O
90 80 70 60 50 40 30 20 10 0 -10 -20 -30 -40 -50 -60 -70 -80 -90
chemical shift

2v-^{1}H

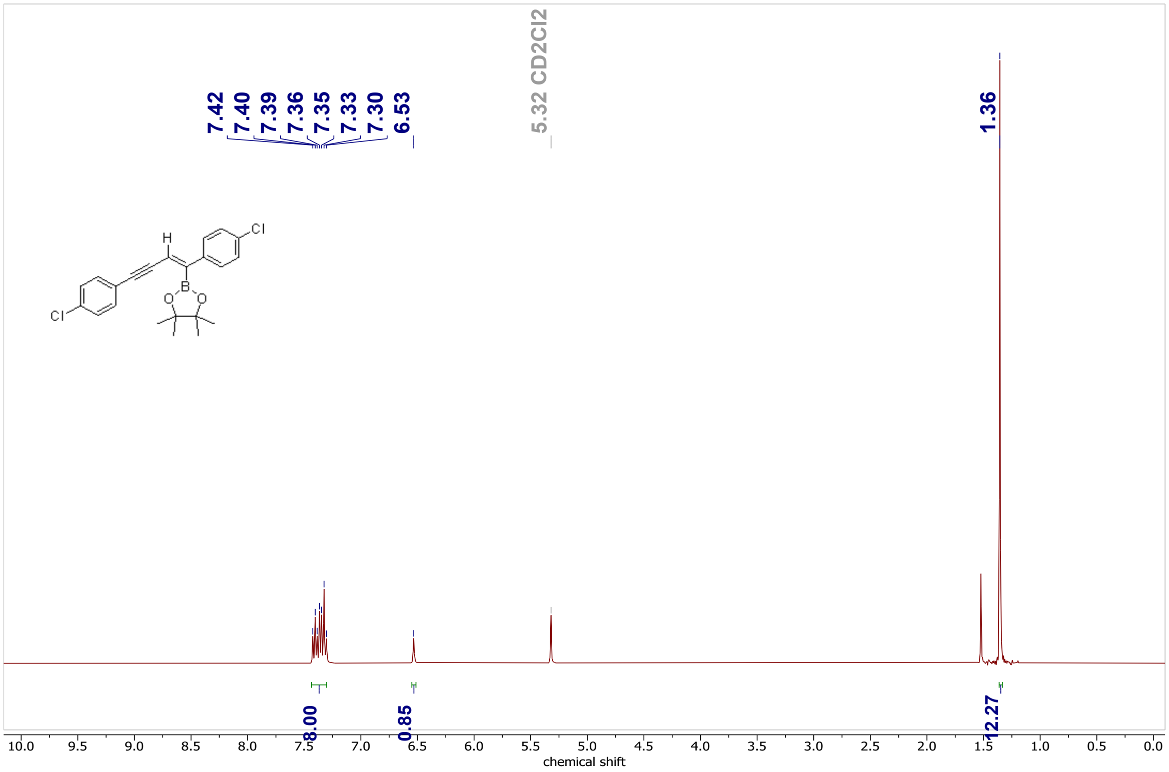
7.42 7.40 7.39 7.36 7.35 7.33 7.30 6.53
5.32 CD2Cl2
1.36
H
Cl
Cl
B
O
O
8.00
0.85
12.27
10.0 9.5 9.0 8.5 8.0 7.5 7.0 6.5 6.0 5.5 5.0 4.5 4.0 3.5 3.0 2.5 2.0 1.5 1.0 0.5 0.0
chemical shift

2v-^{13}C

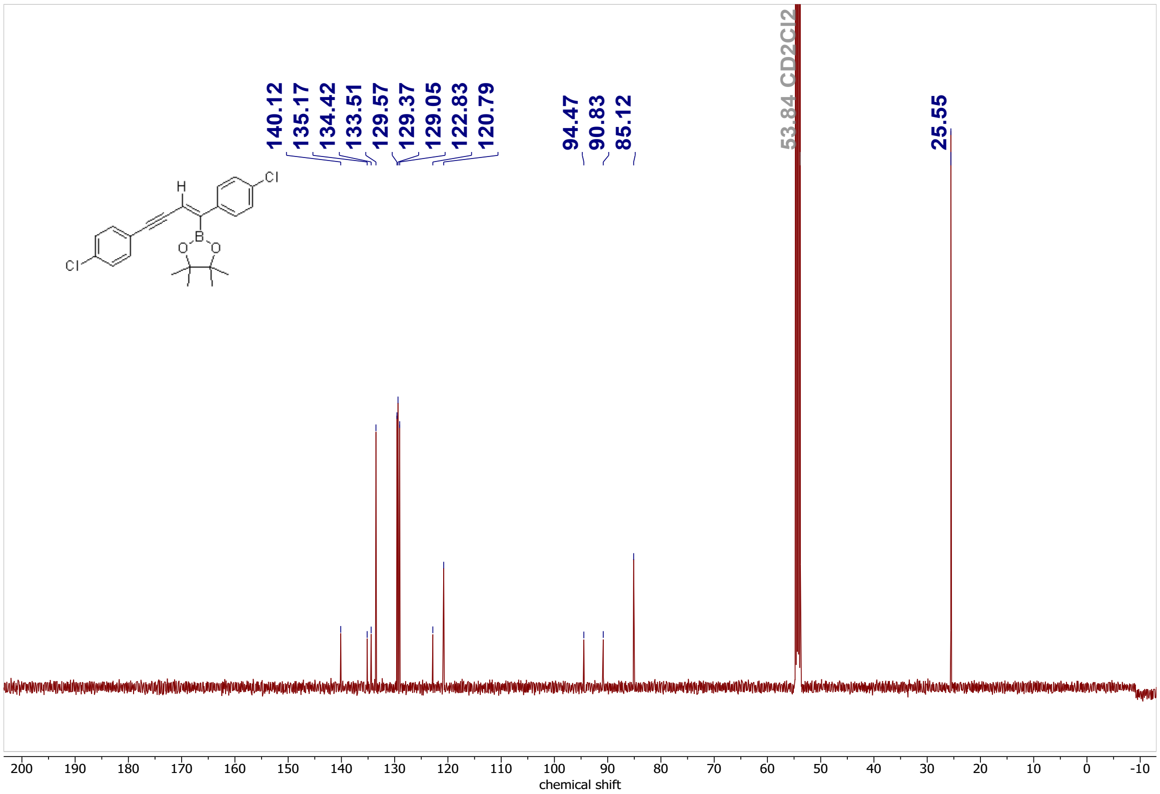

140.12
135.17
134.42
133.51
129.57
129.37
129.05
122.83
120.79
94.47
90.83
85.12
53.84 CD2Cl2
25.55
H
Cl
Cl
B
O
O
200 190 180 170 160 150 140 130 120 110 100 90 80 70 60 50 40 30 20 10 0 -10
chemical shift

2v-^{11}B

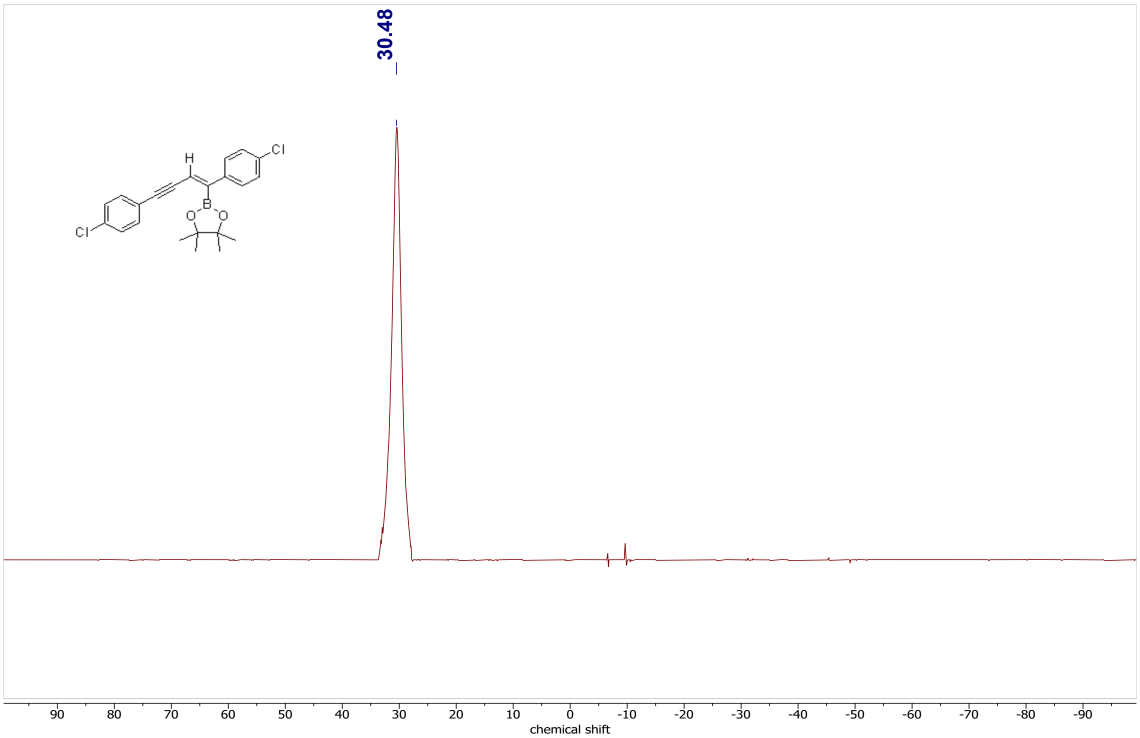

30.48
H
Cl
Cl
B
O
O
90 80 70 60 50 40 30 20 10 0 -10 -20 -30 -40 -50 -60 -70 -80 -90
chemical shift

2w-[1]H (12% *Z* isomer is present)

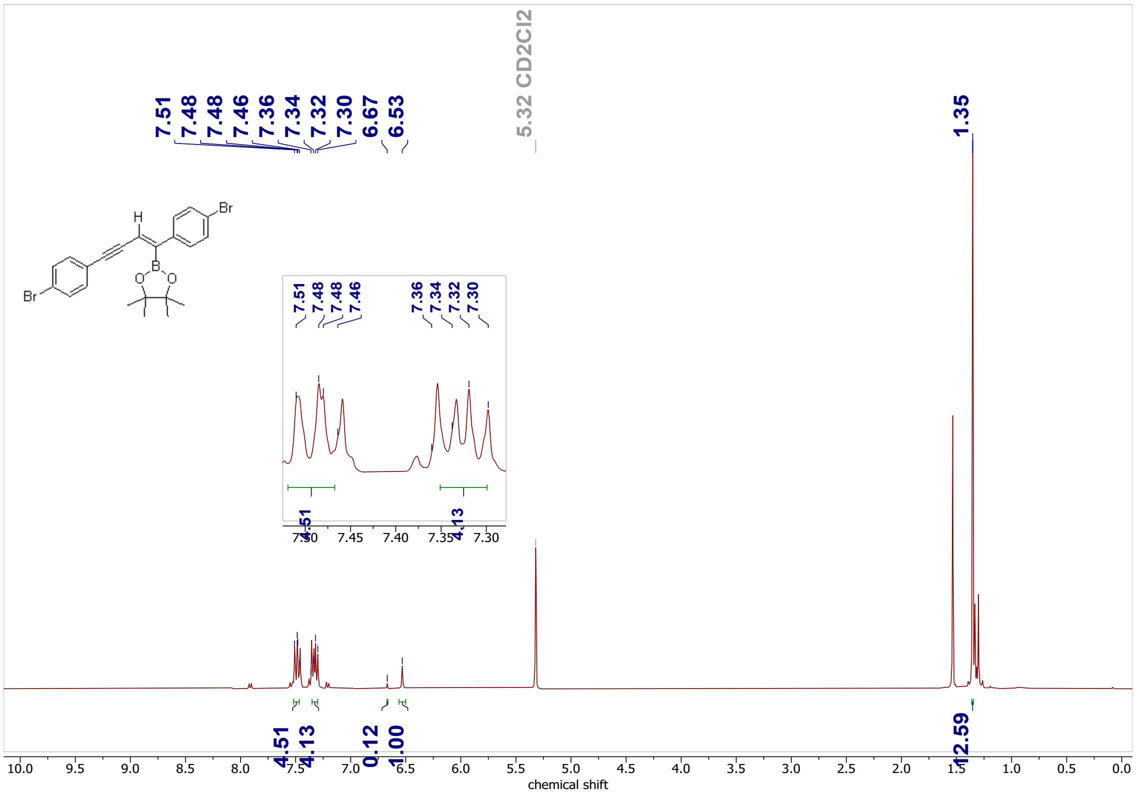

2w-[13]C

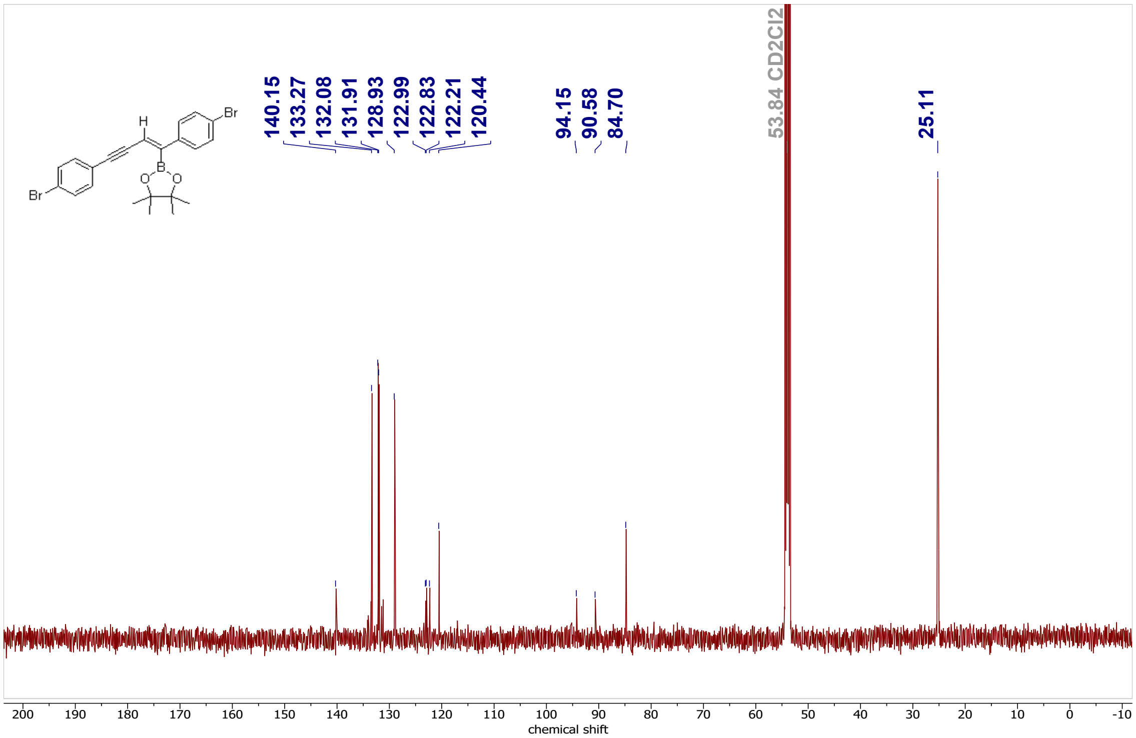

2w-^{11}B

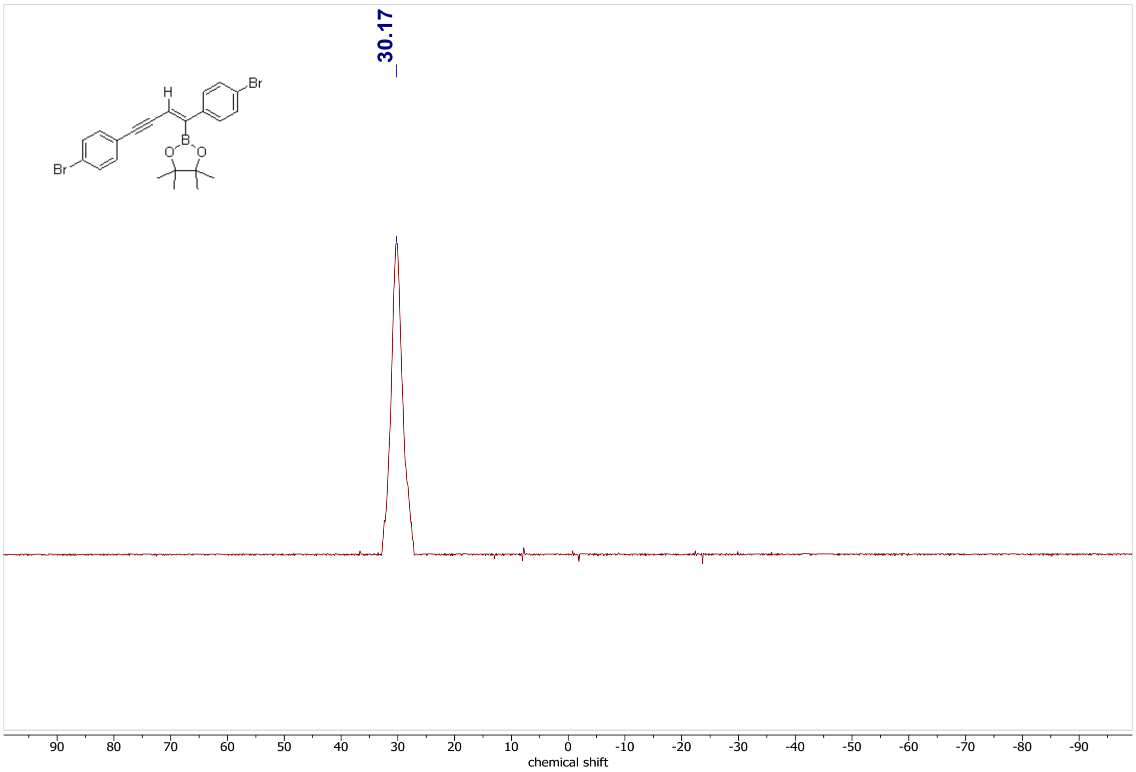
30.17
chemical shift

2x-^{1}H

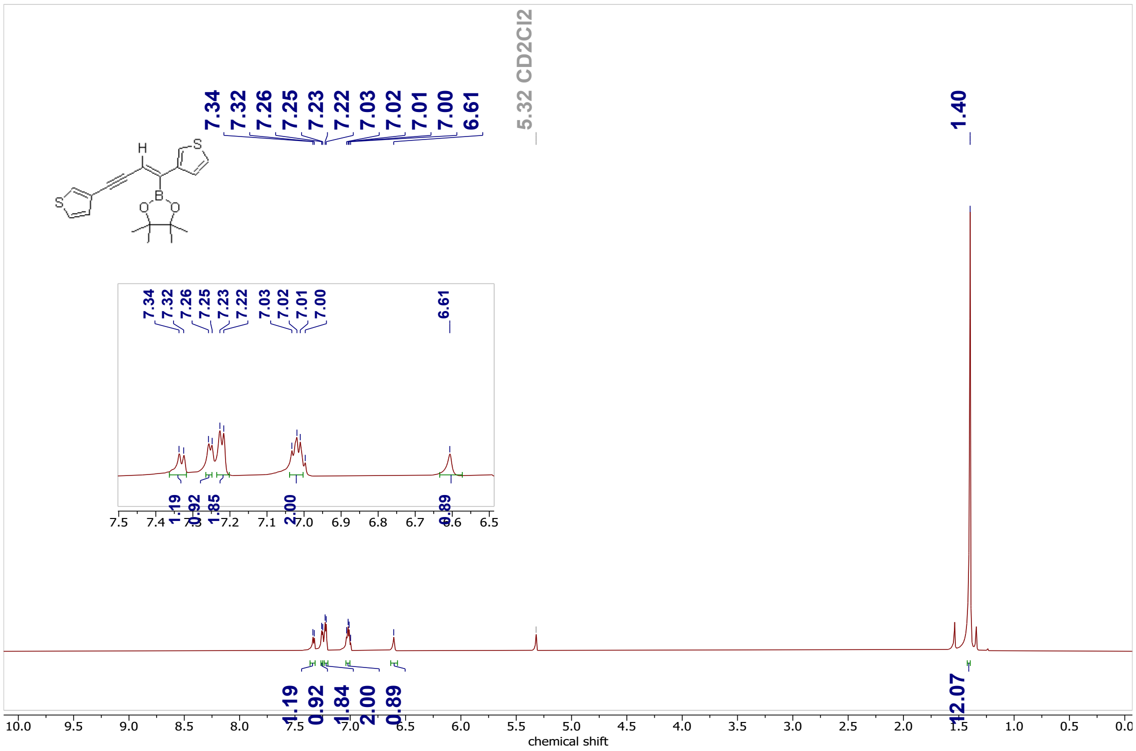
7.34
7.32
7.26
7.25
7.23
7.22
7.03
7.02
7.01
7.00
6.61
5.32 CD2Cl2
1.40
1.19
0.92
1.84
2.00
0.89
12.07
chemical shift

2x-^{13}C

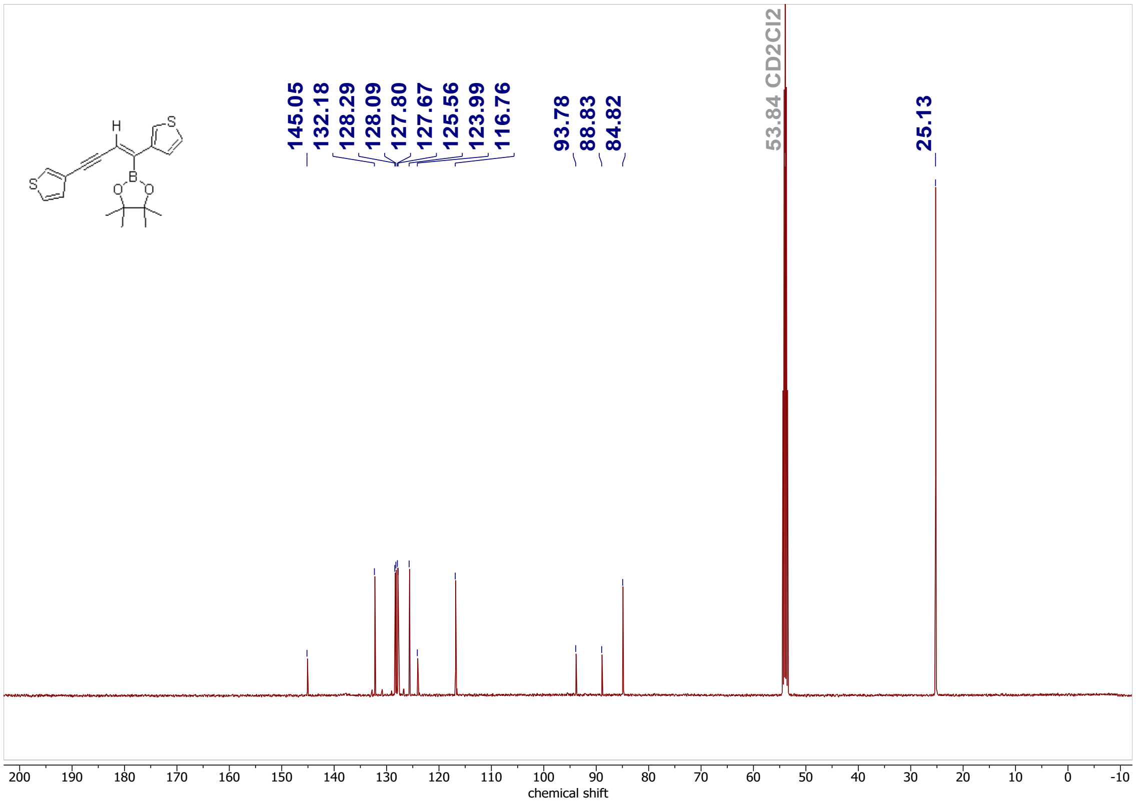

145.05
132.18
128.29
128.09
127.80
127.67
125.56
123.99
116.76
93.78
88.83
84.82
53.84 CD2Cl2
25.13
200 190 180 170 160 150 140 130 120 110 100 90 80 70 60 50 40 30 20 10 0 -10
chemical shift

2x-^{11}B

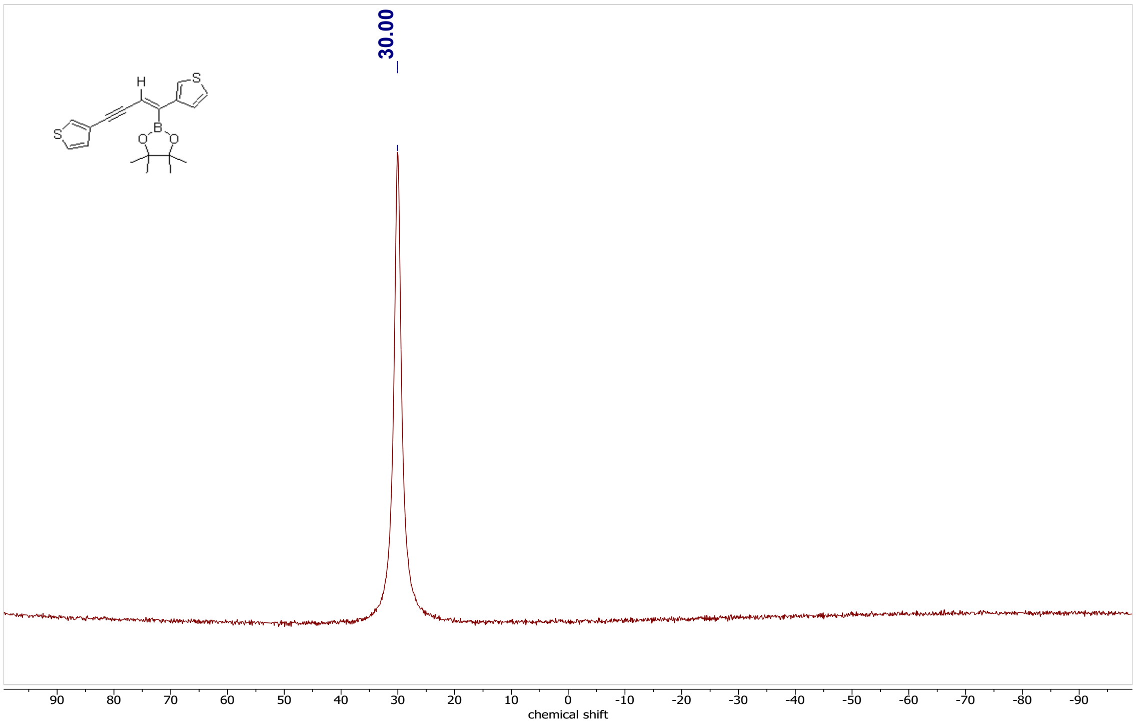

30.00
90 80 70 60 50 40 30 20 10 0 -10 -20 -30 -40 -50 -60 -70 -80 -90
chemical shift

2y-[1]H

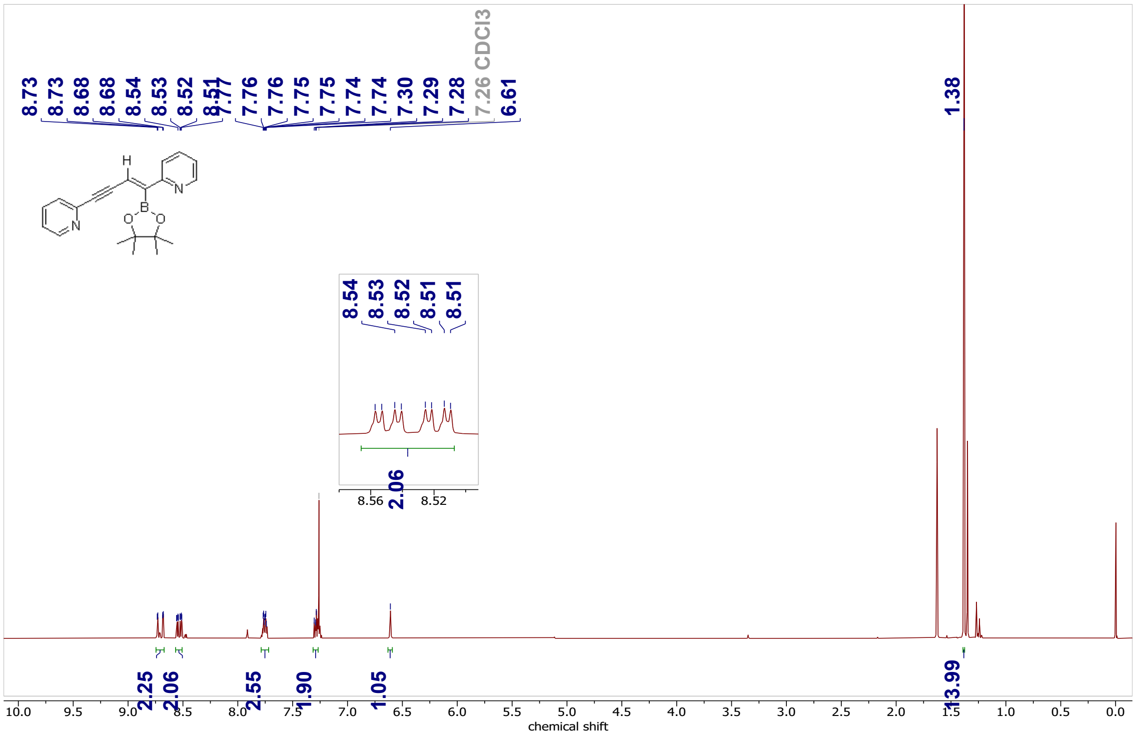
8.73
8.73
8.68
8.68
8.54
8.53
8.52
8.51
7.77
7.76
7.76
7.75
7.75
7.74
7.74
7.30
7.29
7.28
7.26 CDCl3
6.61
1.38
H
N
B
O
O
N
8.54
8.53
8.52
8.51
8.51
2.06
8.56
8.52
2.25
2.06
2.55
1.90
1.05
13.99
10.0
9.5
9.0
8.5
8.0
7.5
7.0
6.5
6.0
5.5
5.0
4.5
4.0
3.5
3.0
2.5
2.0
1.5
1.0
0.5
0.0
chemical shift

2y-[13]C

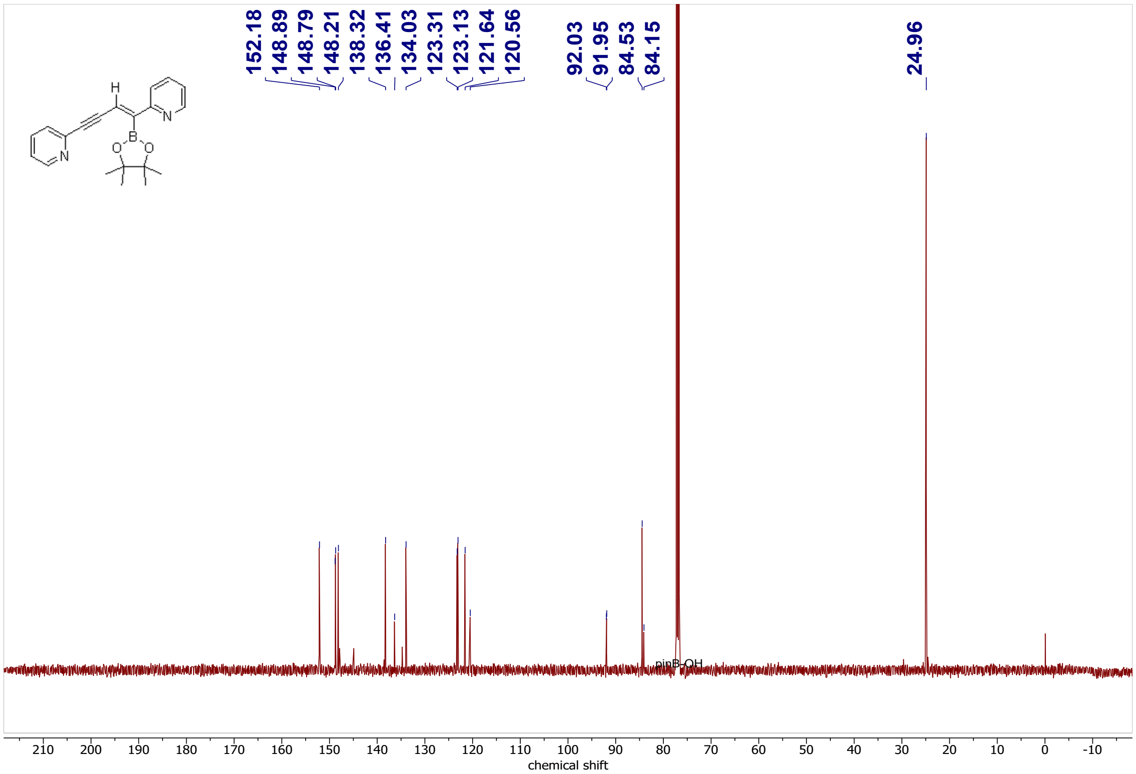
152.18
148.89
148.79
148.21
138.32
136.41
134.03
123.31
123.13
121.64
120.56
92.03
91.95
84.53
84.15
24.96
H
N
B
O
O
N
pinB-OH
210
200
190
180
170
160
150
140
130
120
110
100
90
80
70
60
50
40
30
20
10
0
-10
chemical shift

2y-^{11}B

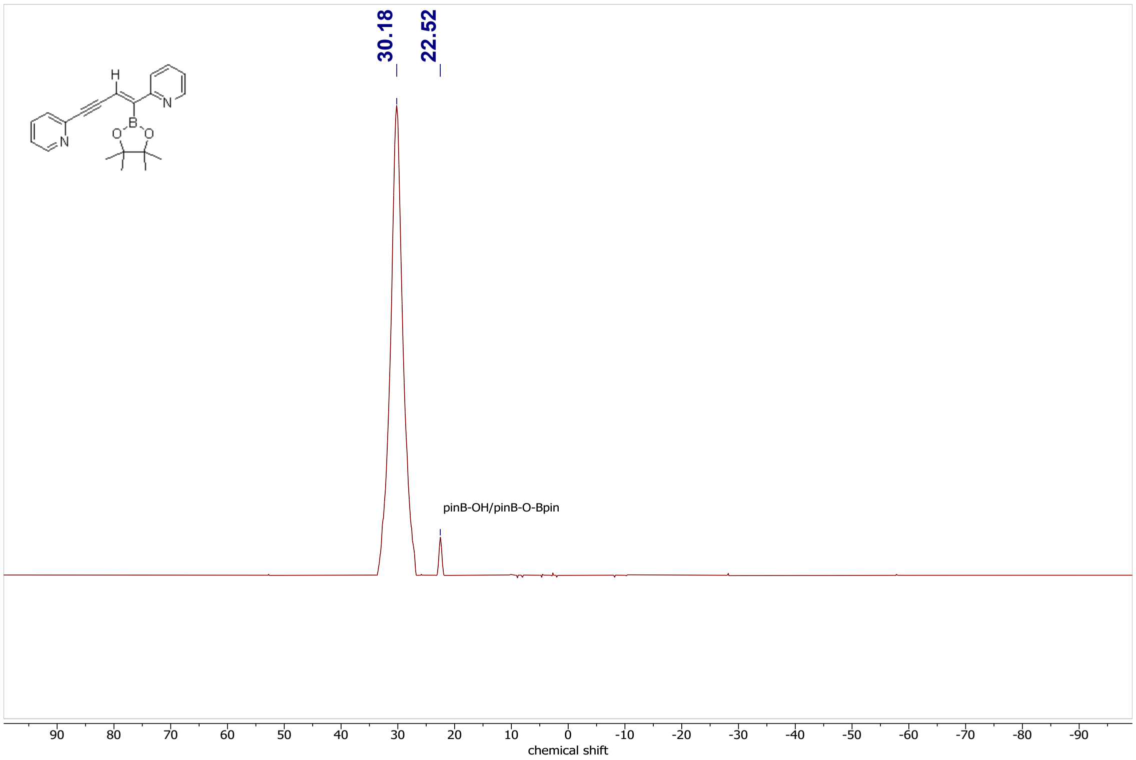

2z-^{1}H (crude NMR)

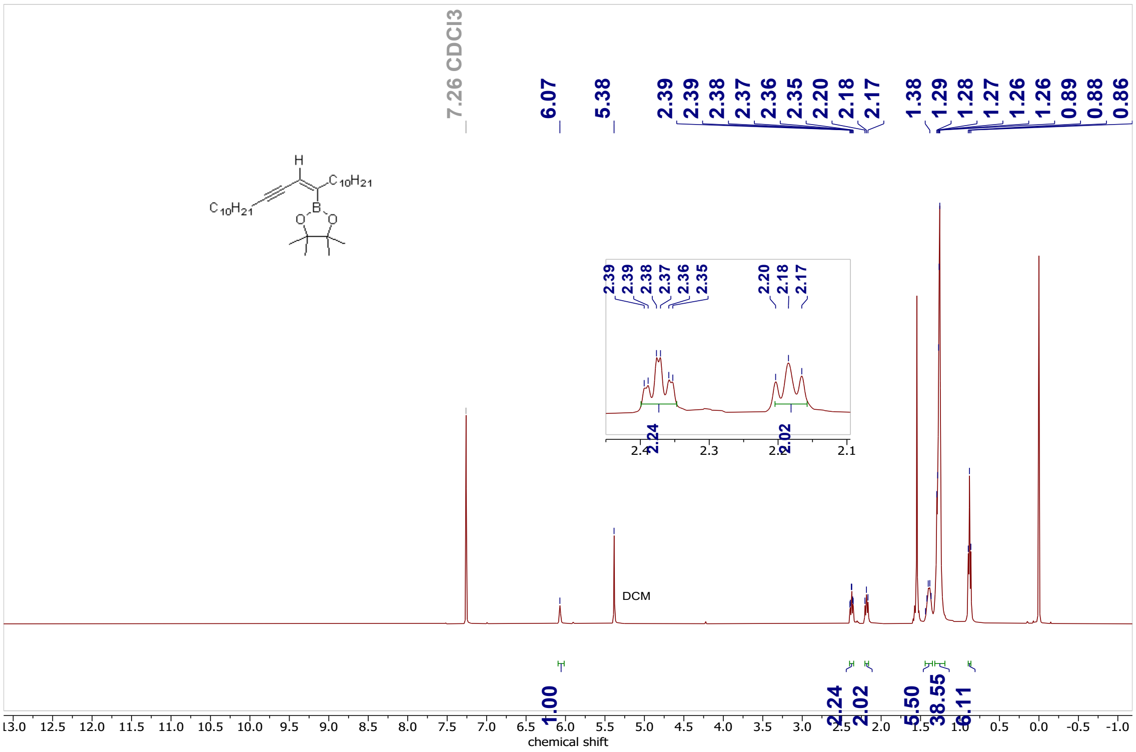

2z-^{13}C (crude NMR)

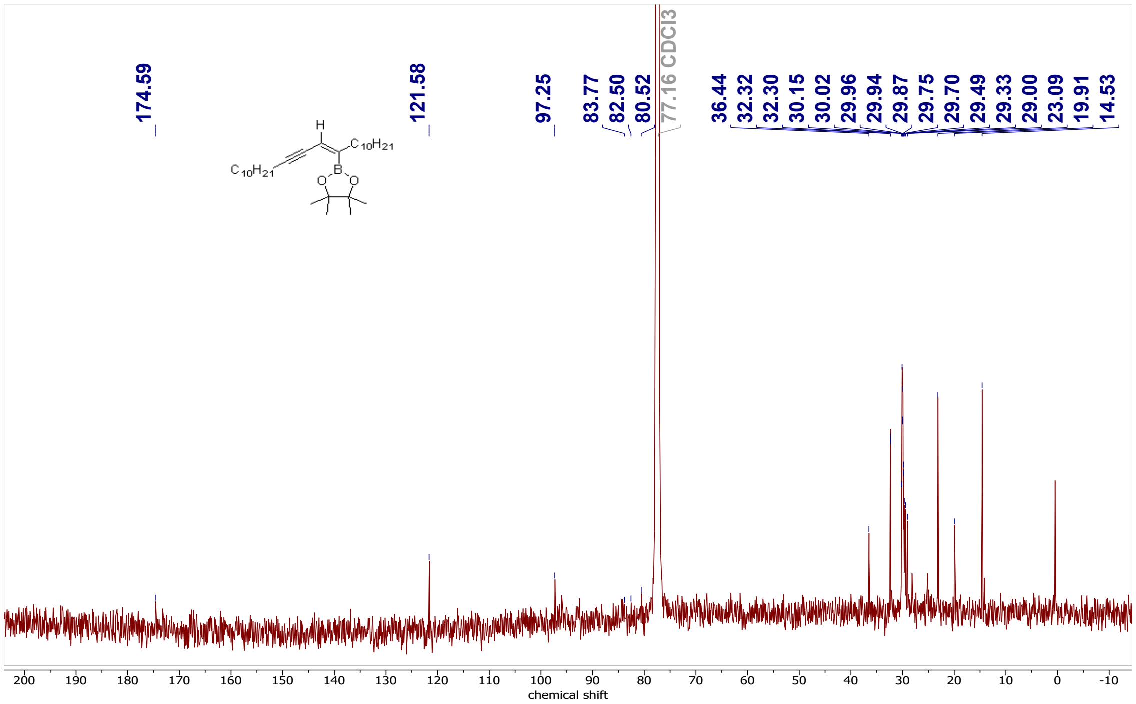

2z-^{11}B

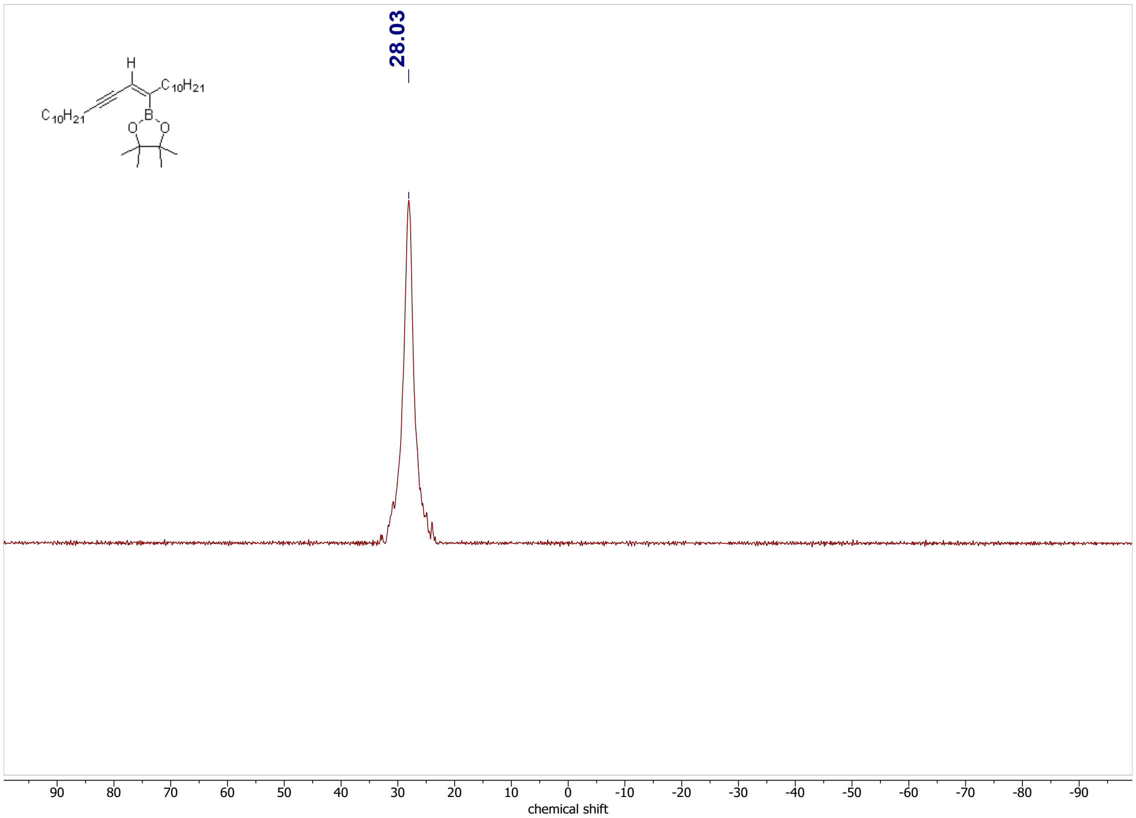

2aa-^{1}H

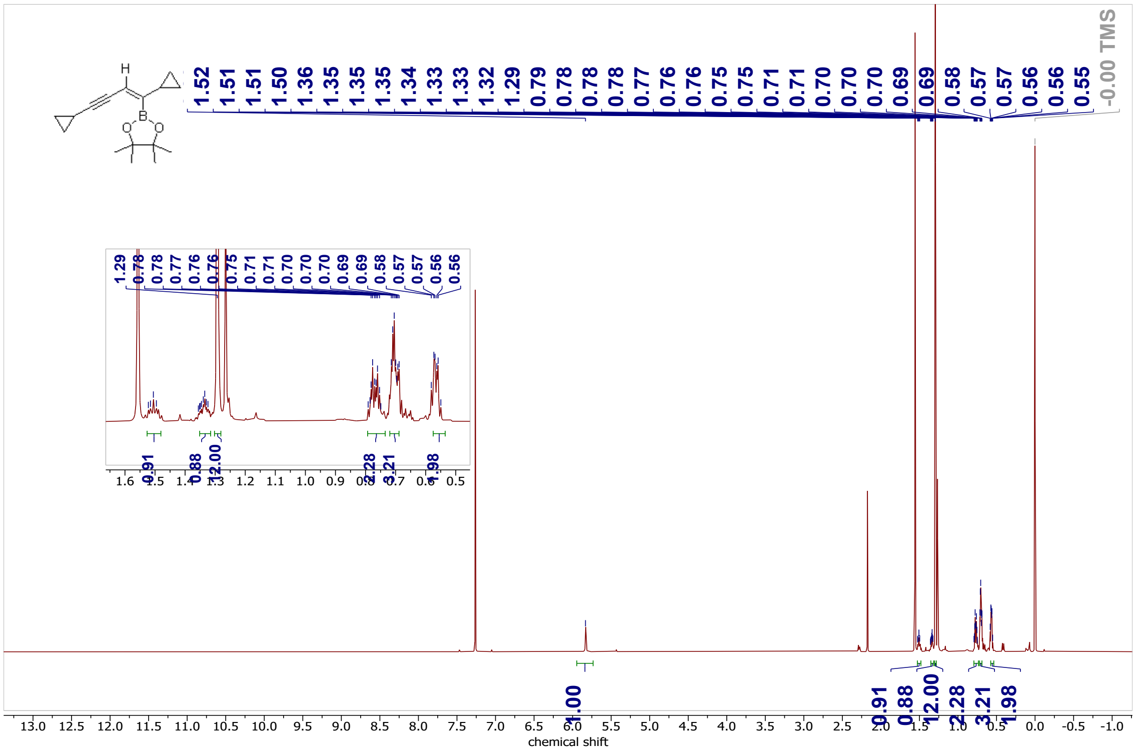

2aa-^{13}C- contains residual starting material and/ *Z* isomer

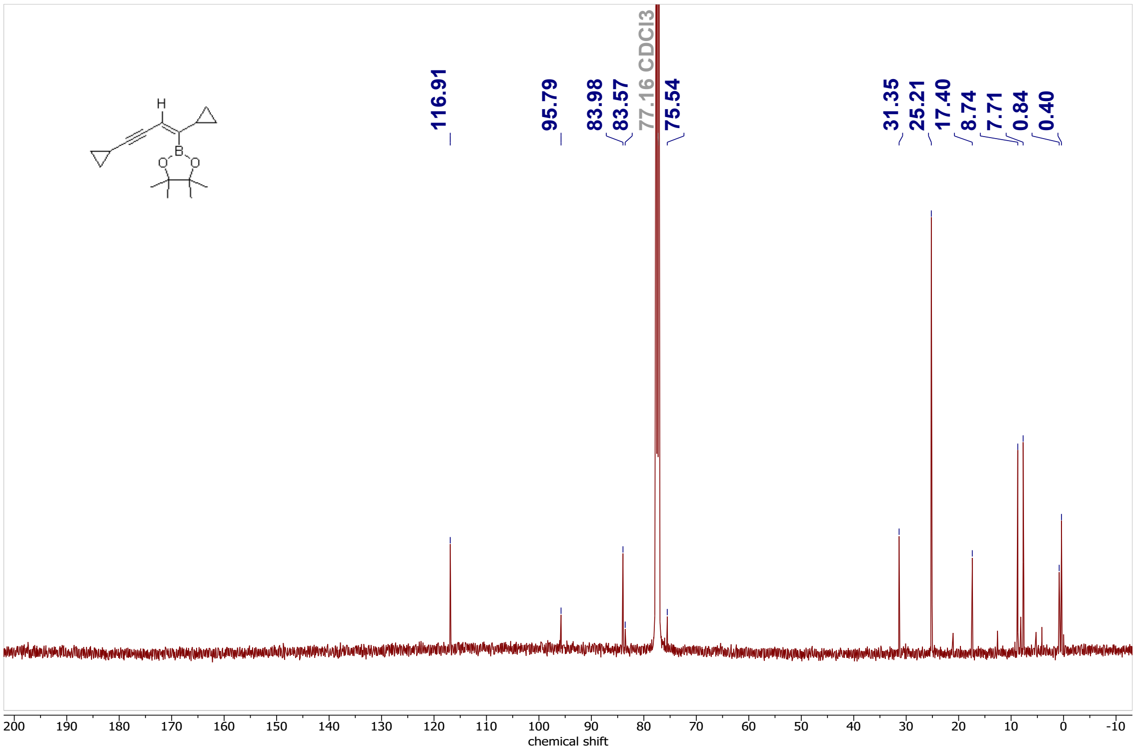

2aa-^{11}B

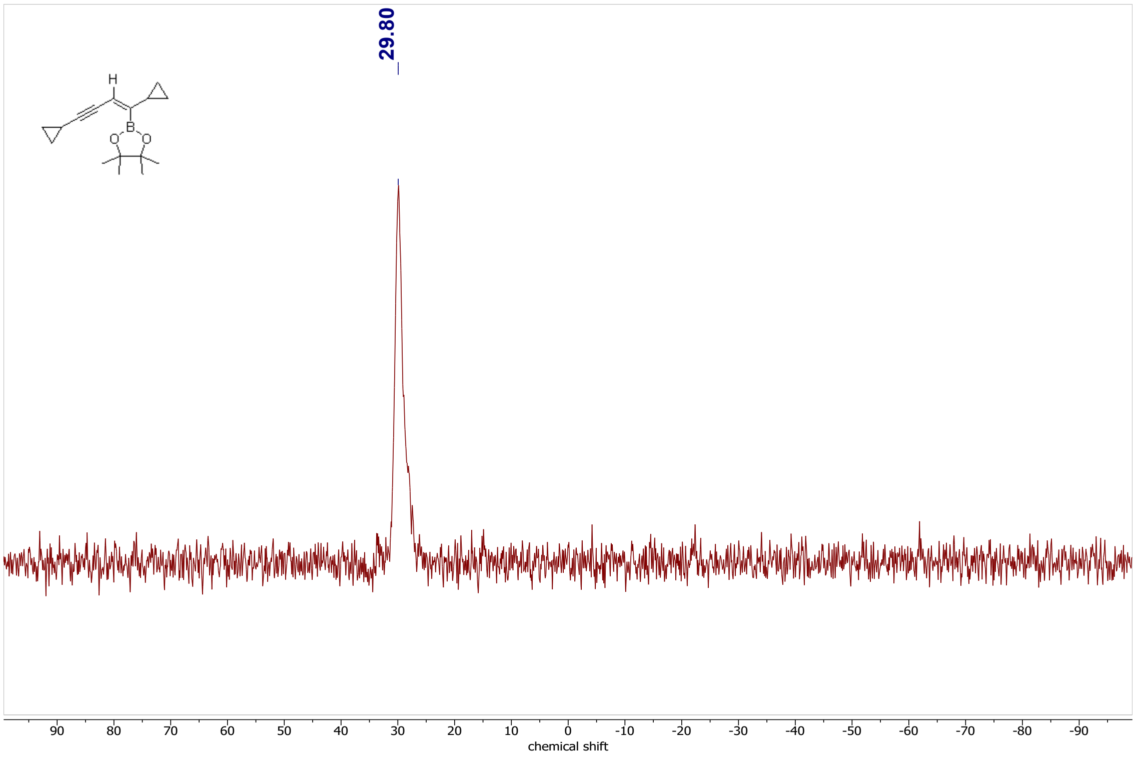

2ab-^{1}H

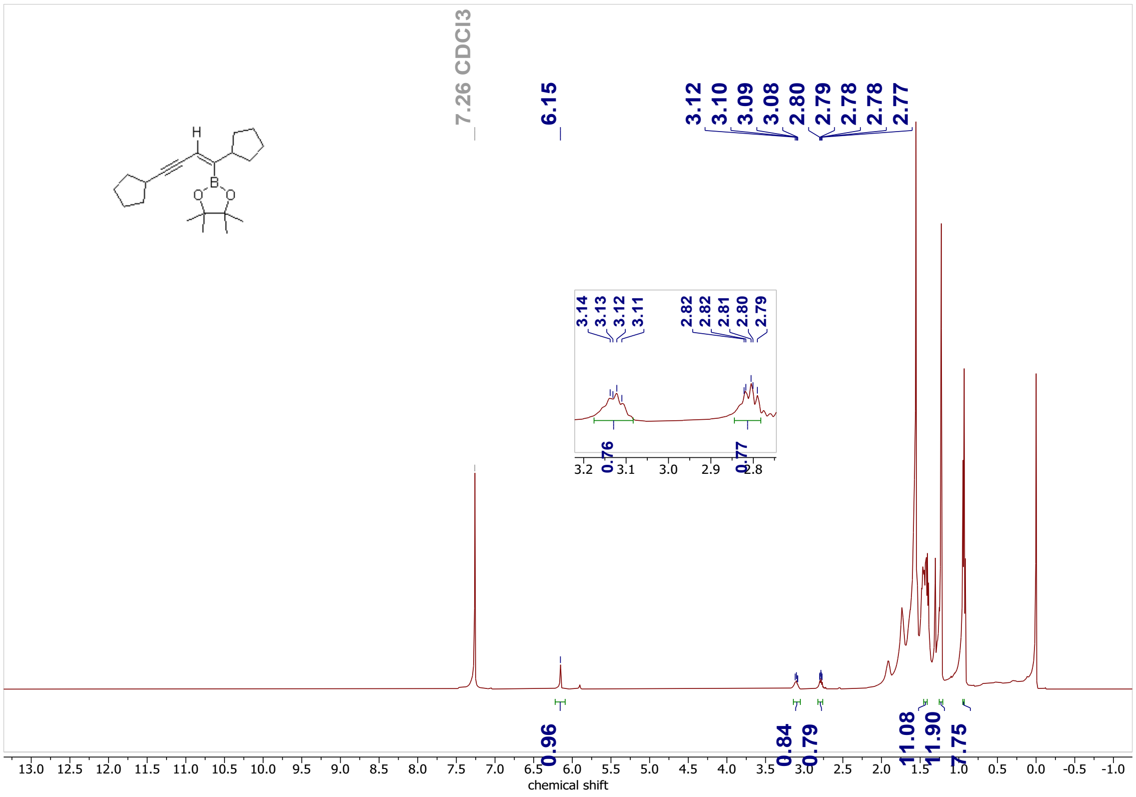

2ab-^{13}C

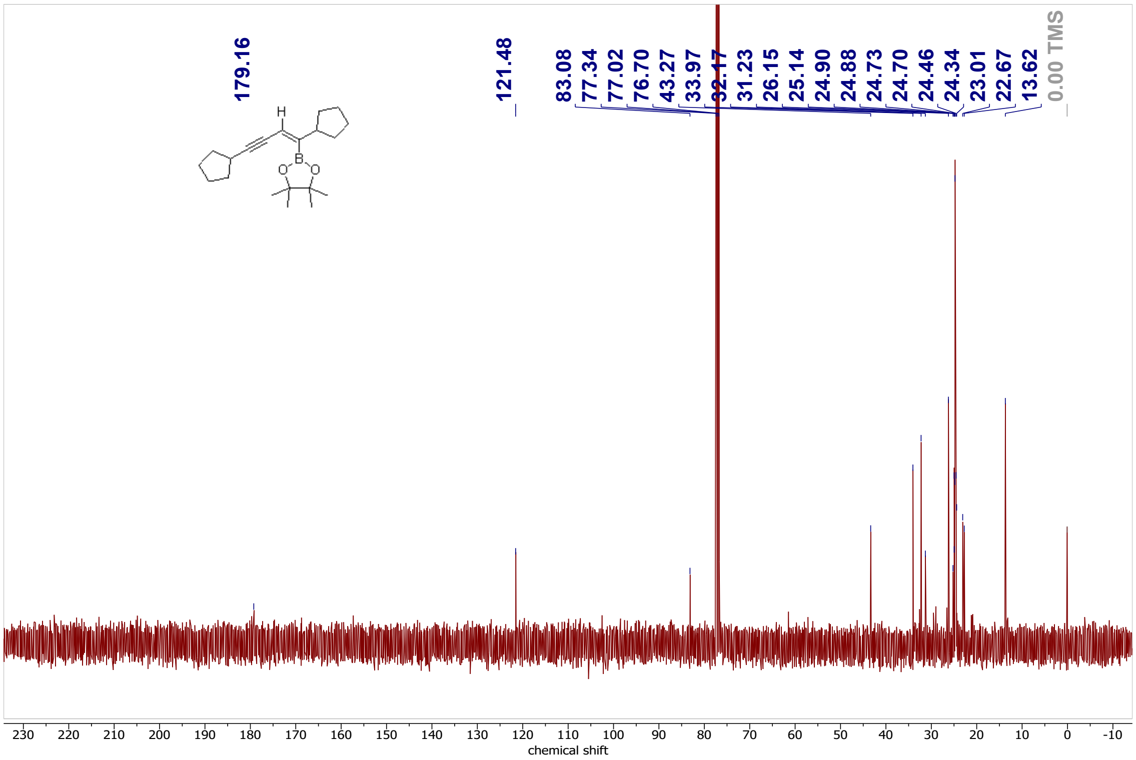

2ab-^{11}B

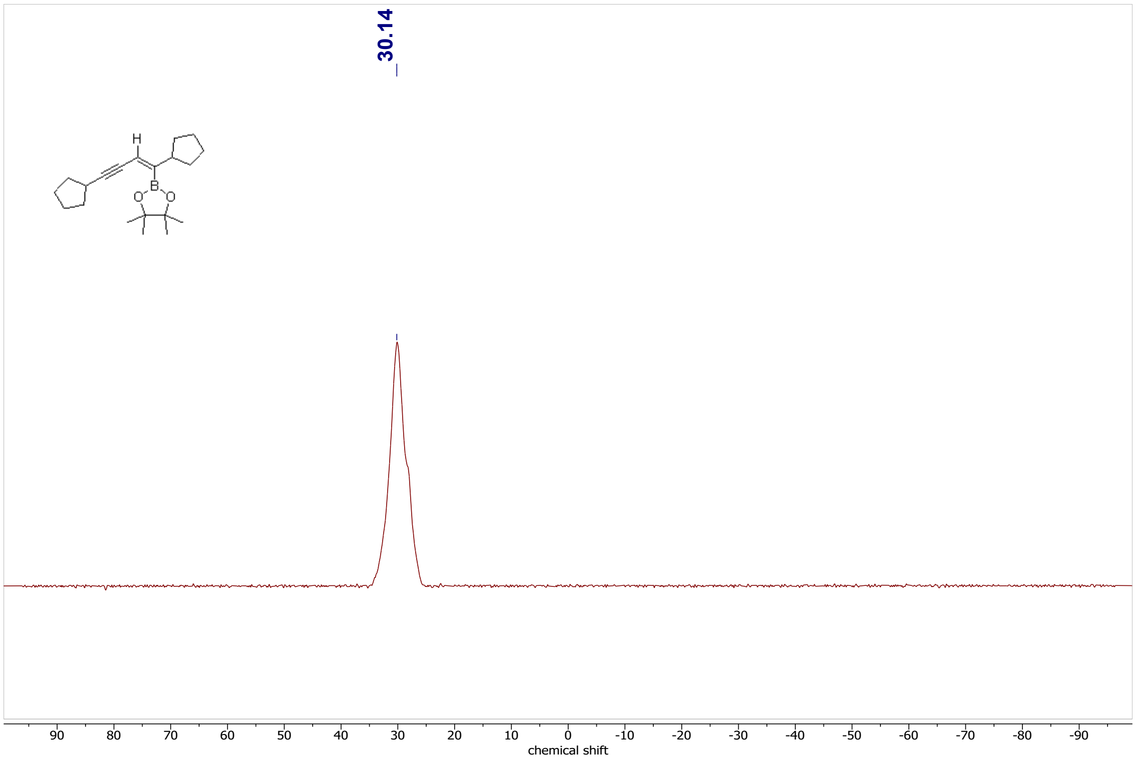

2ac-^{1}H

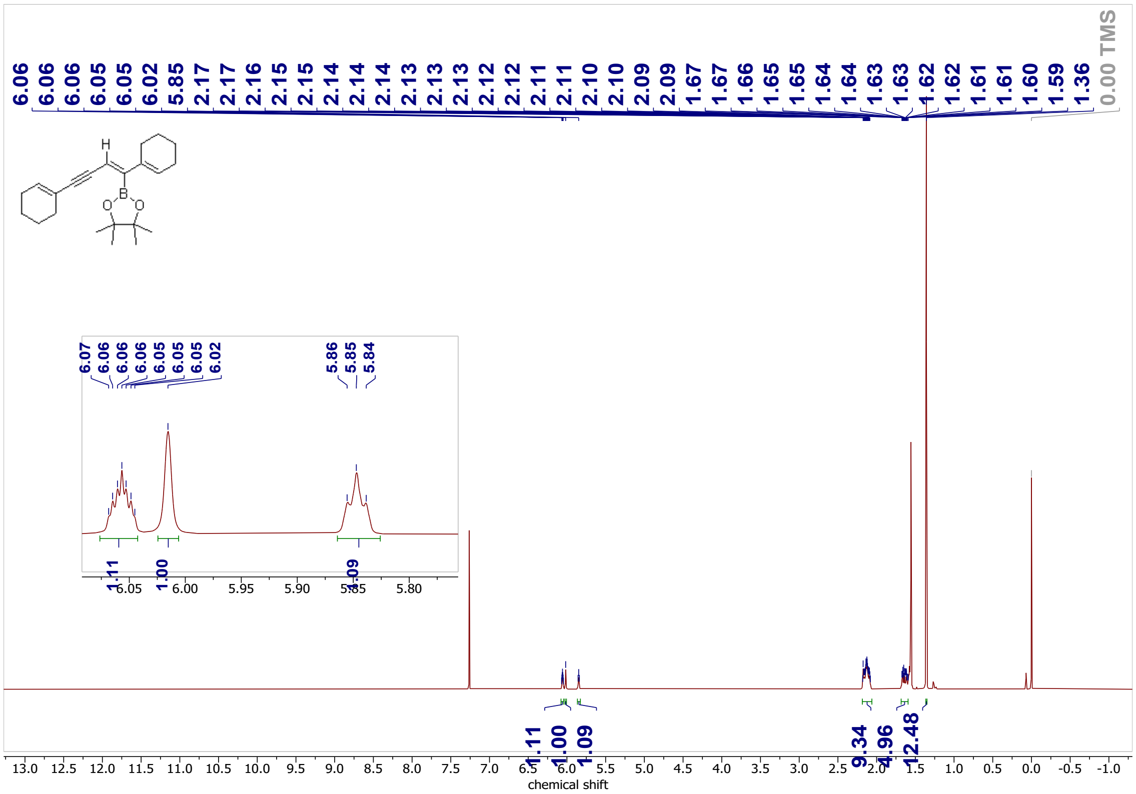

2ac-^{13}C

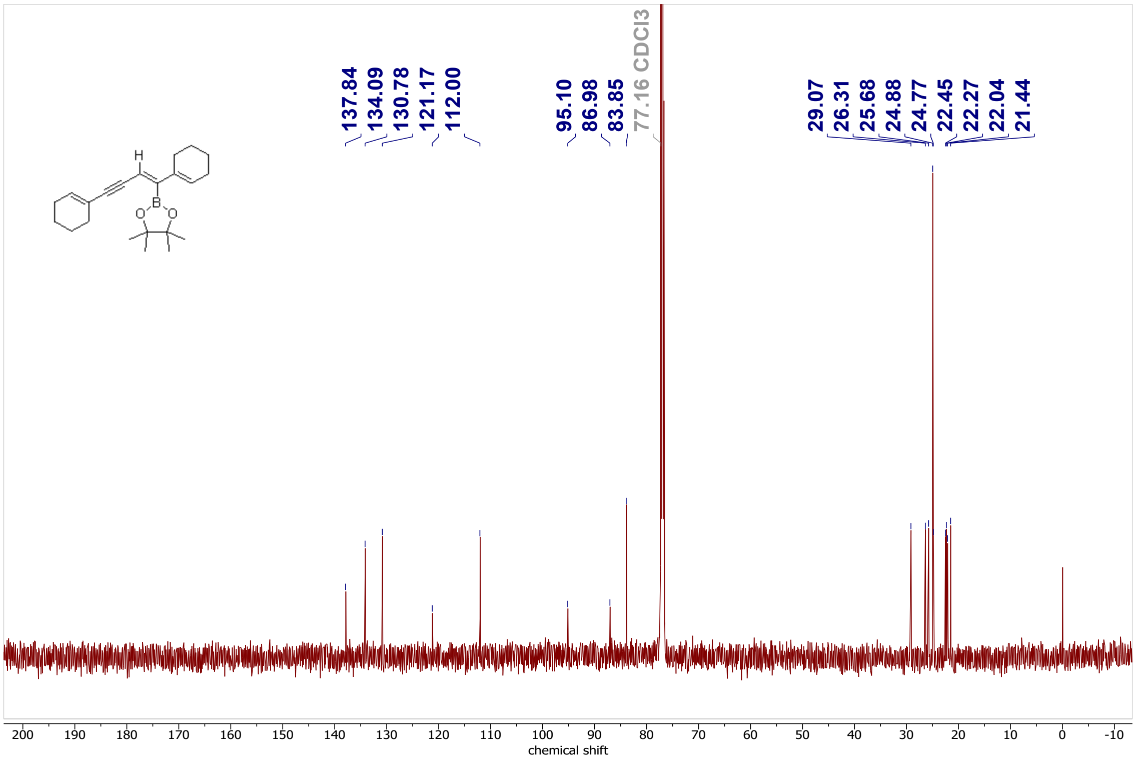

2ac-^{11}B

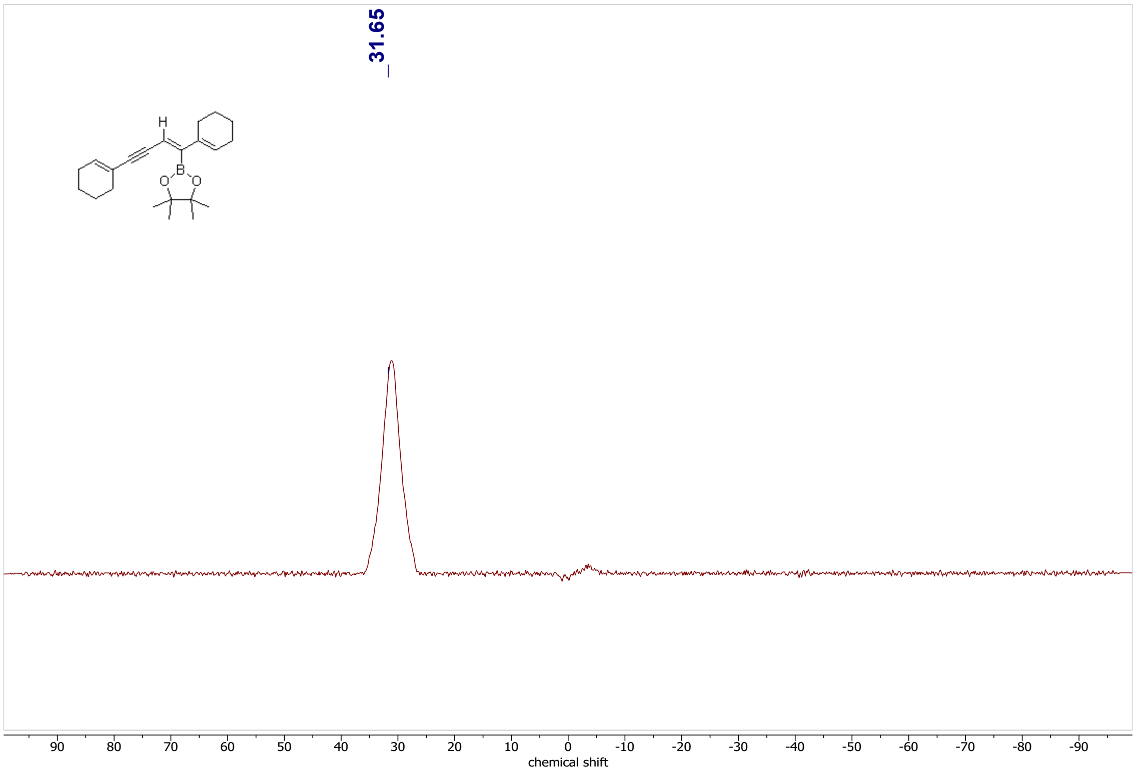

2ad-^{1}H

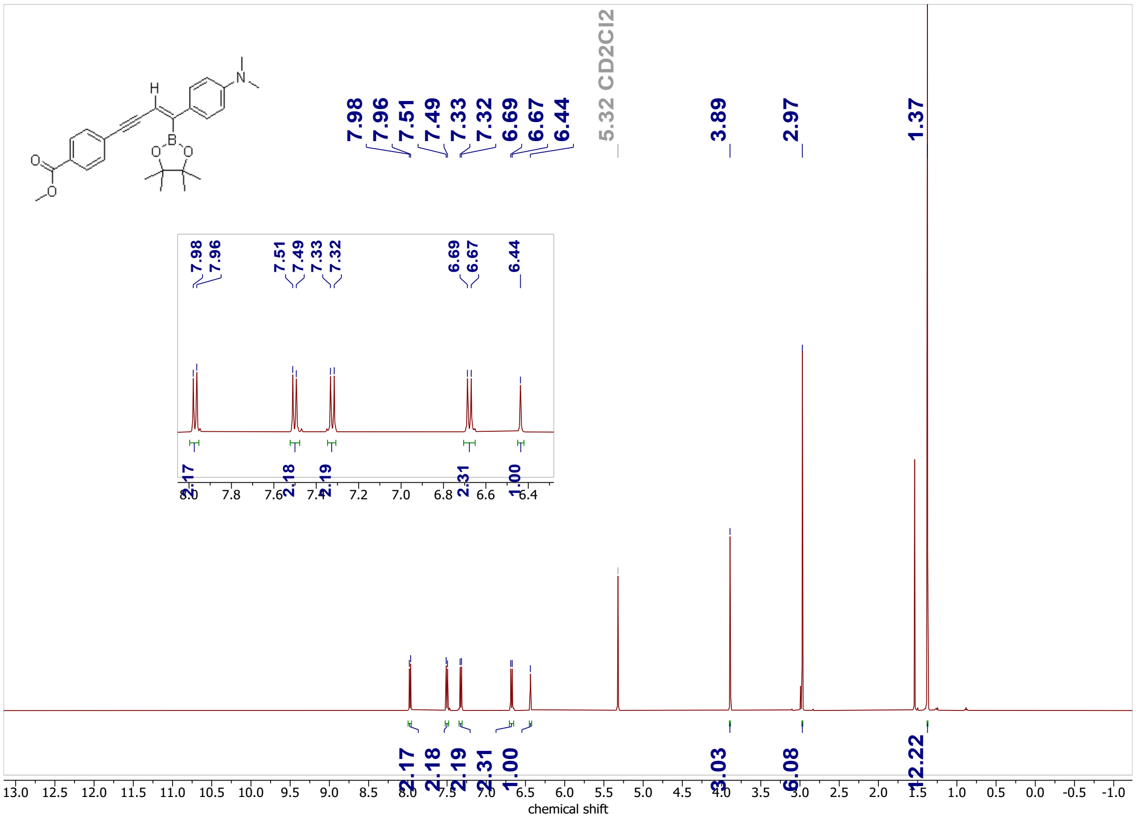

2ad-^{13}C

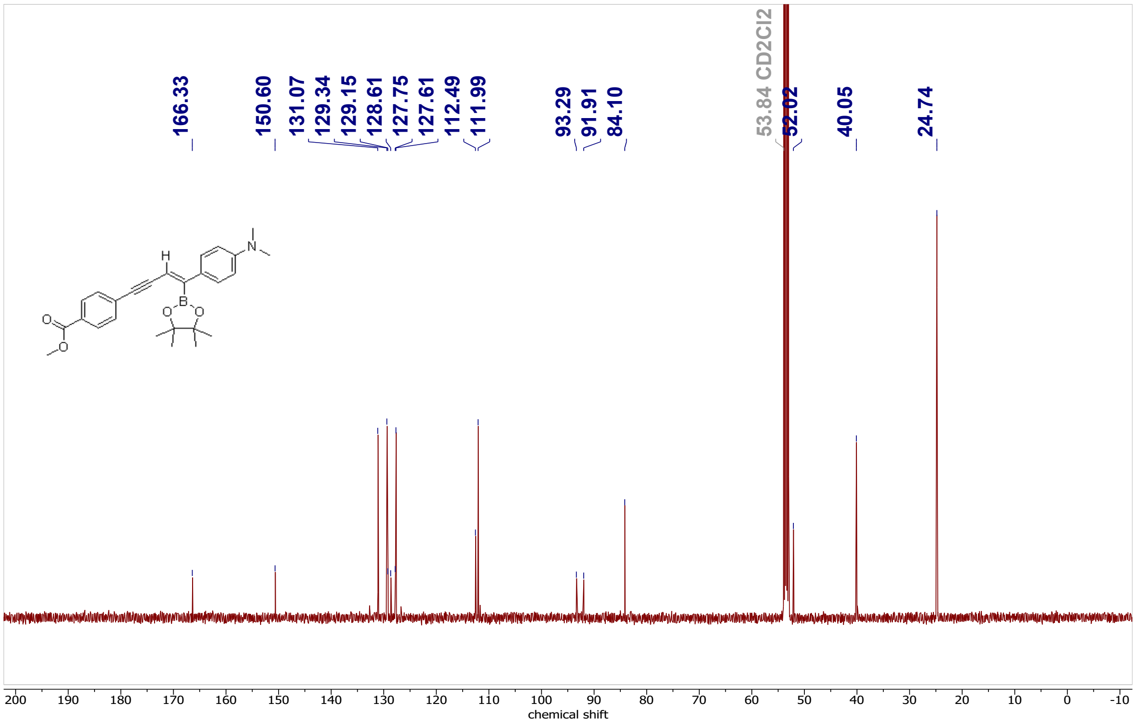

2ad-^{11}B

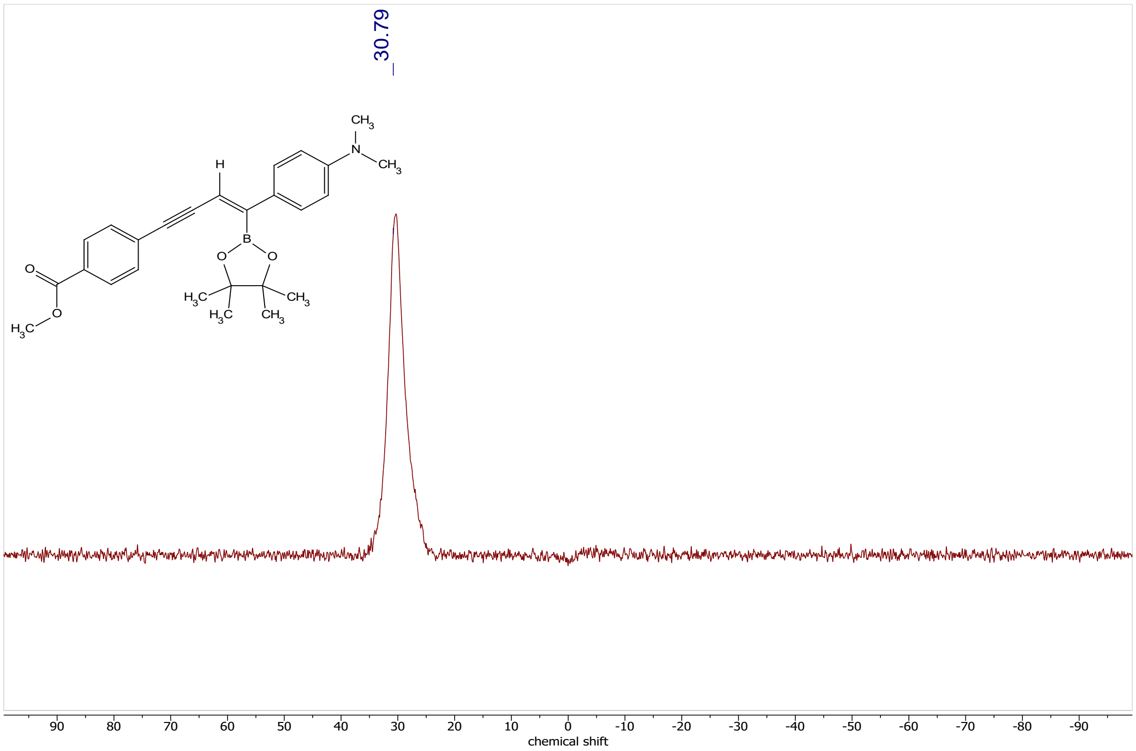

2ad1-1H

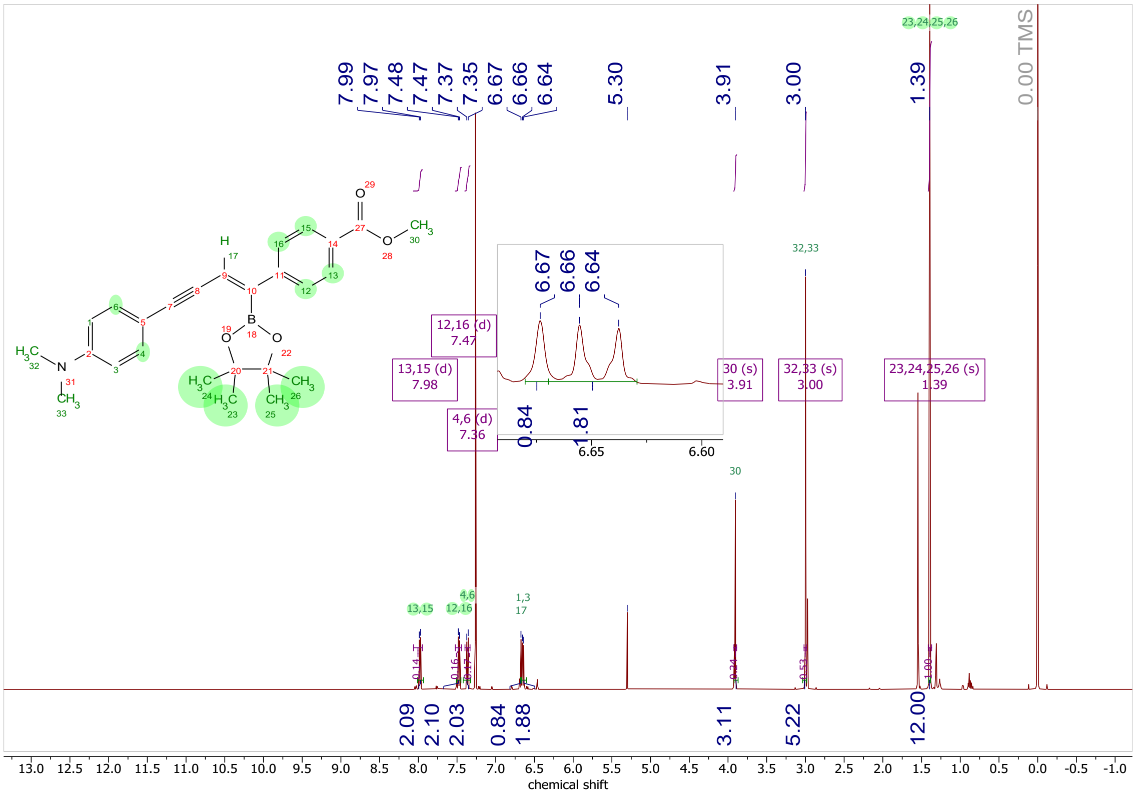

2ad1-13C

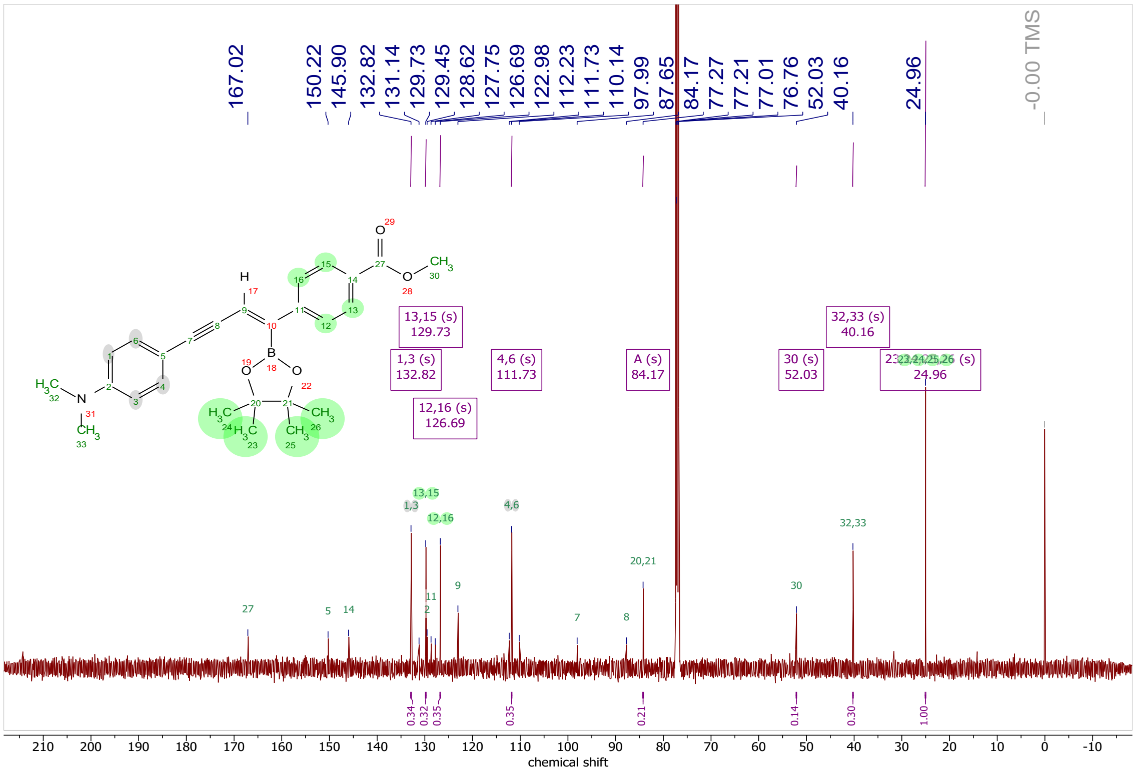

2ad1-11B

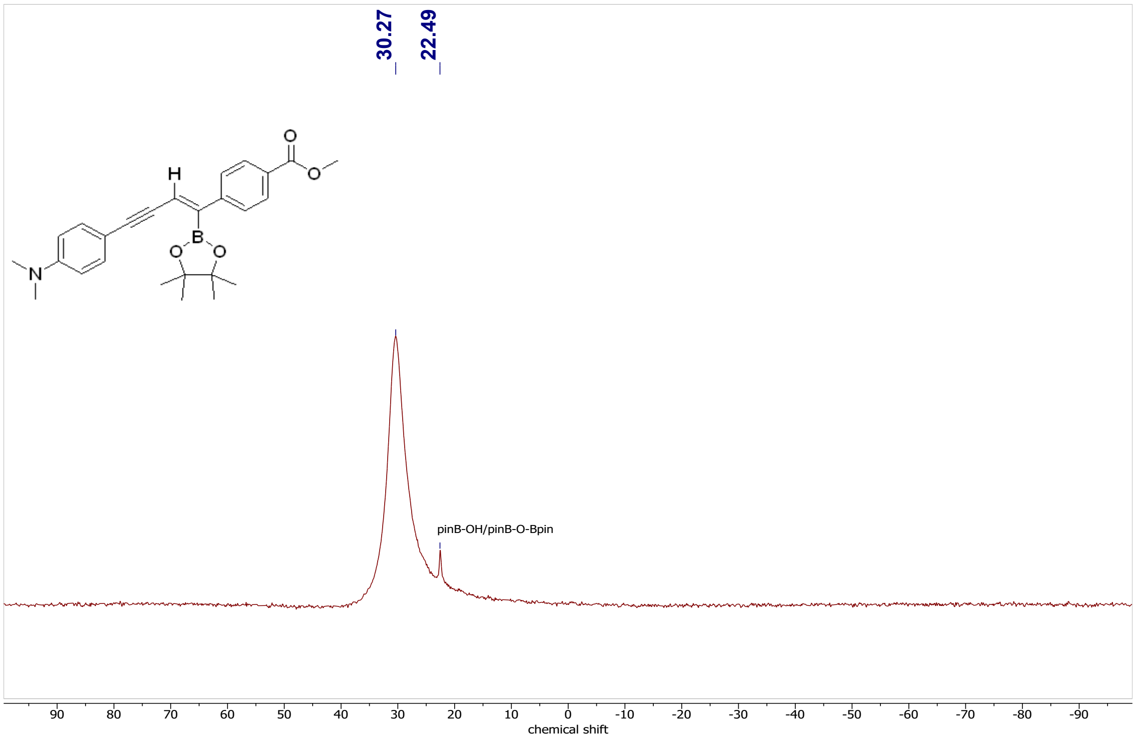

HSQC

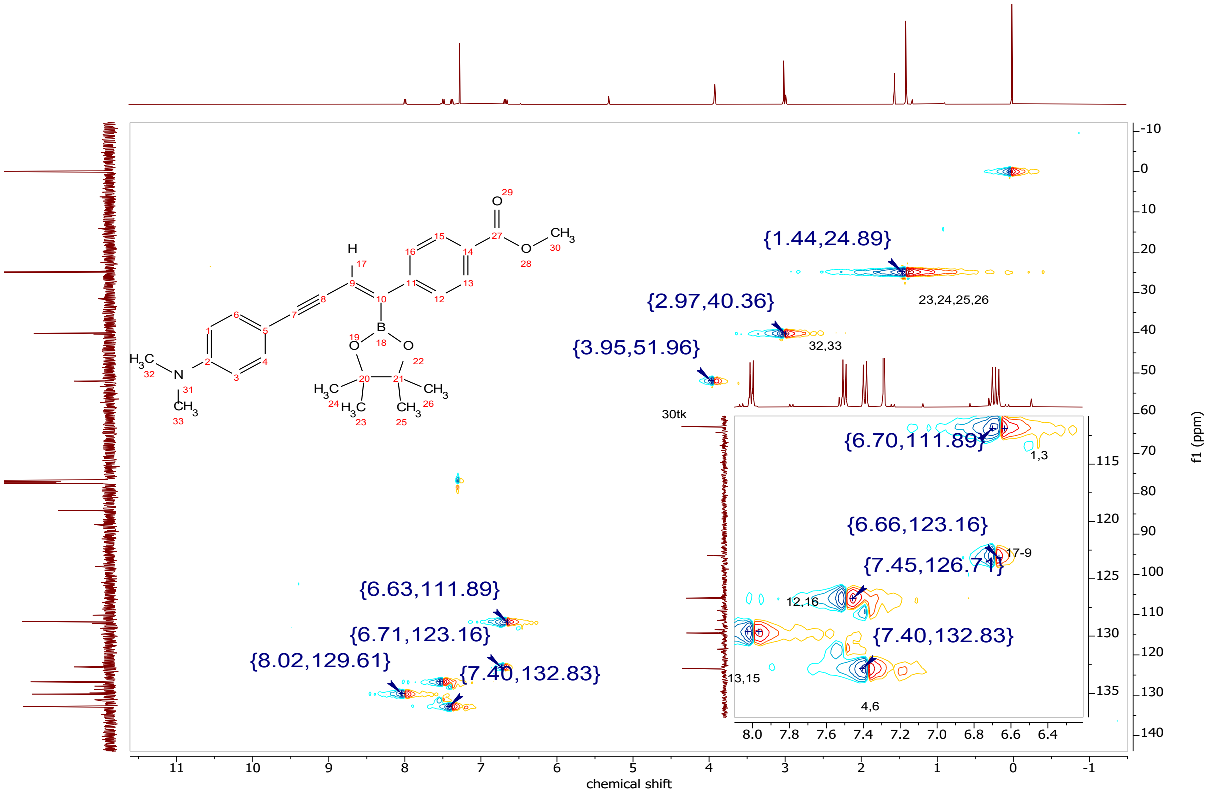

HMBC

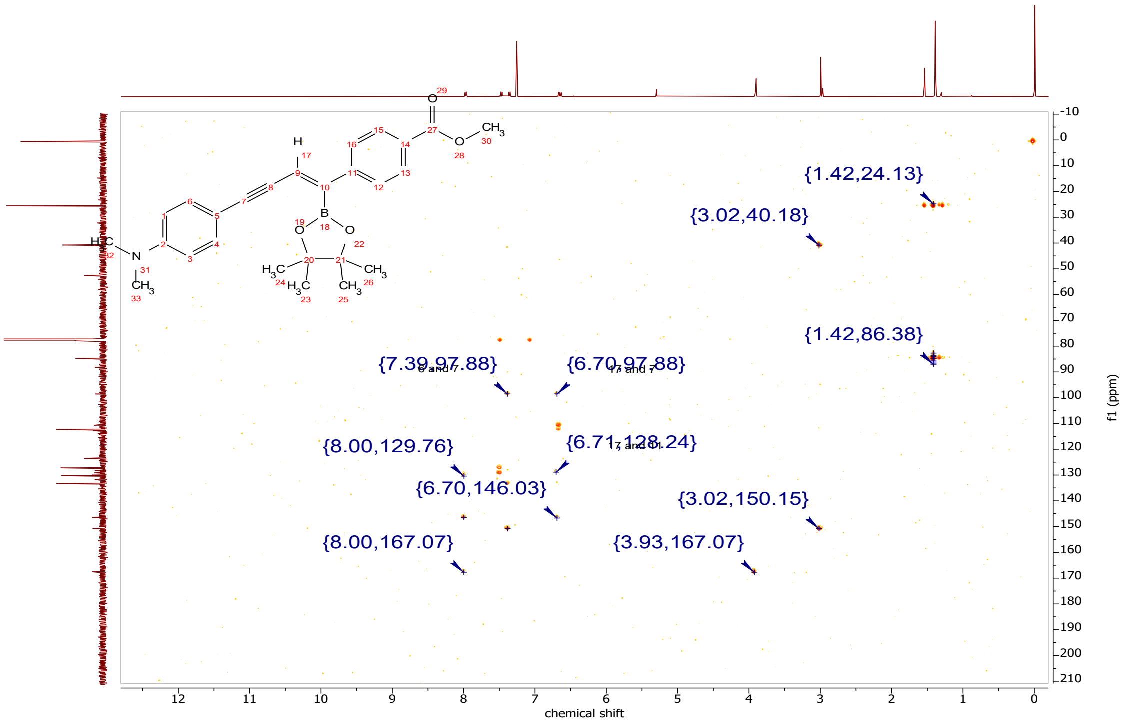
{1.42,24.13}
{3.02,40.18}
{1.42,86.38}
{7.39,97.88}
{6.70,97.88}
{8.00,129.76}
{6.71,128.24}
{6.70,146.03}
{3.02,150.15}
{8.00,167.07}
{3.93,167.07}
chemical shift
f1 (ppm)

2ae-^{1}H

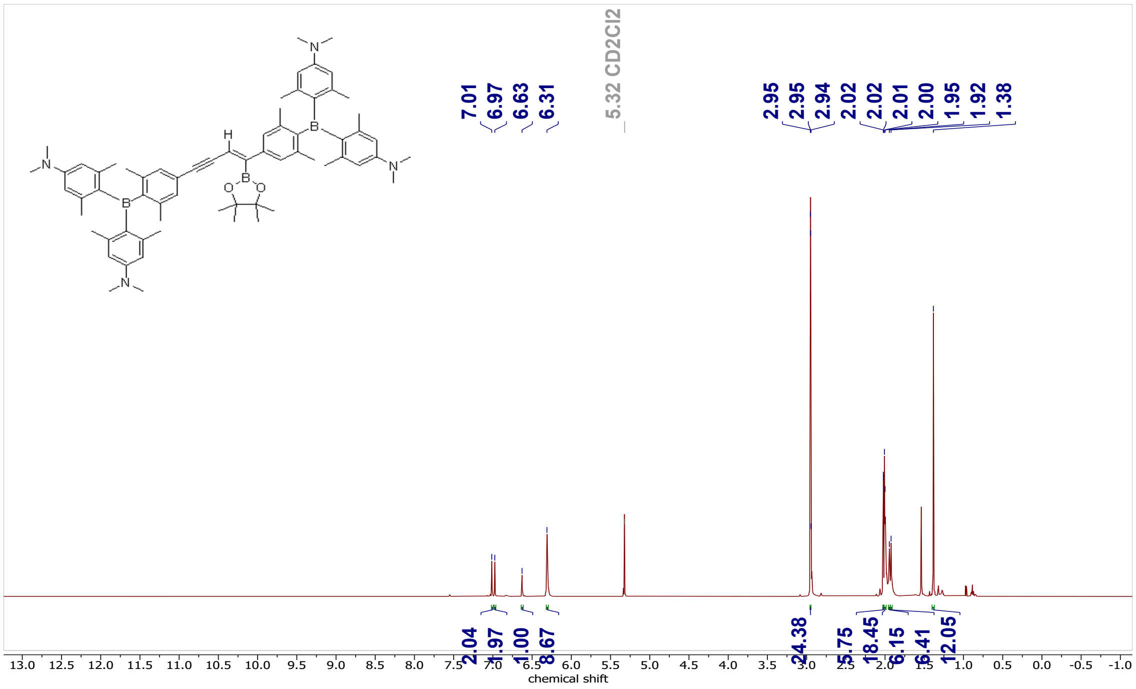
7.01
6.97
6.63
6.31
5.32 CD2Cl2
2.95
2.95
2.94
2.02
2.02
2.01
2.00
1.95
1.92
1.38
2.04
1.97
1.00
8.67
24.38
5.75
18.45
6.15
6.41
12.05
chemical shift

2ae-^{13}C

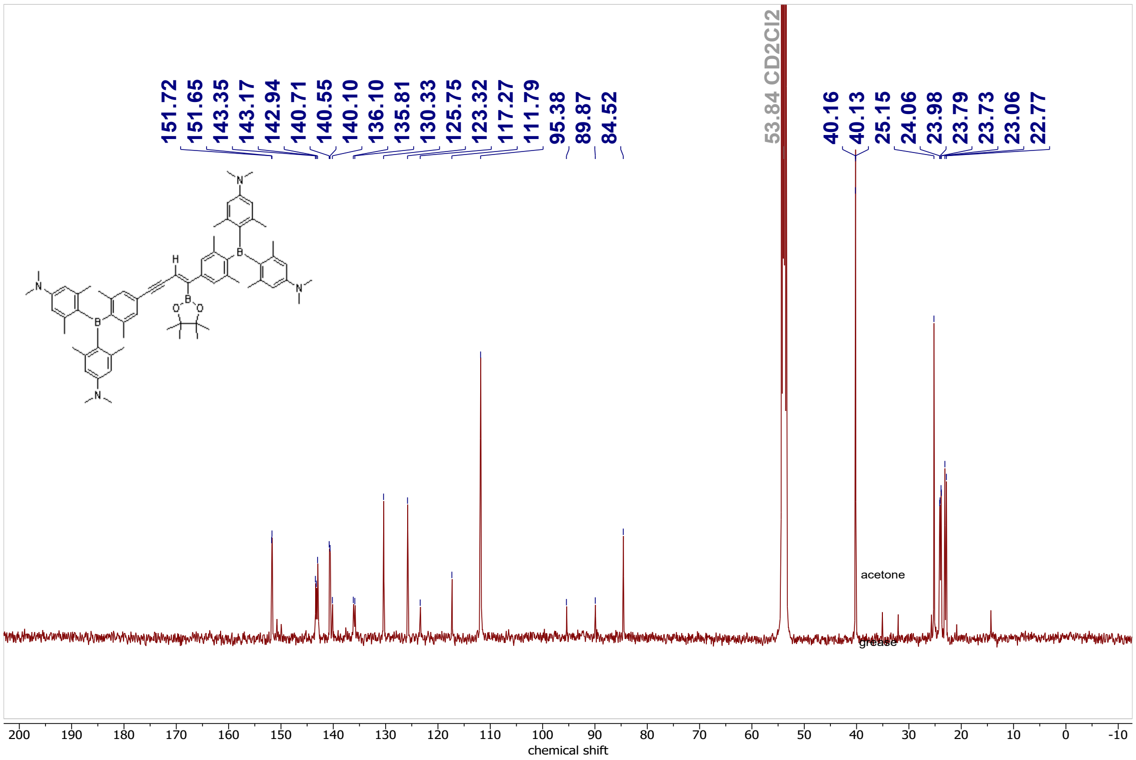

2ae-^{11}B

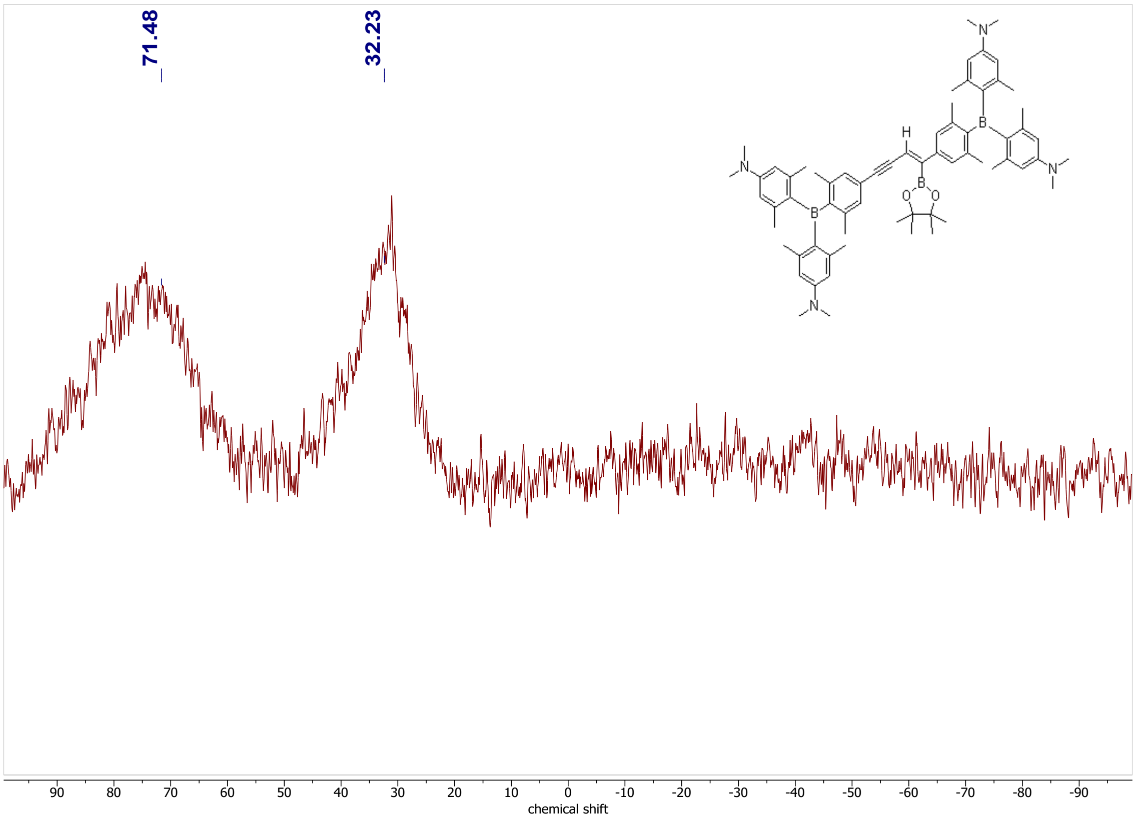

3b-^{1}H -crude

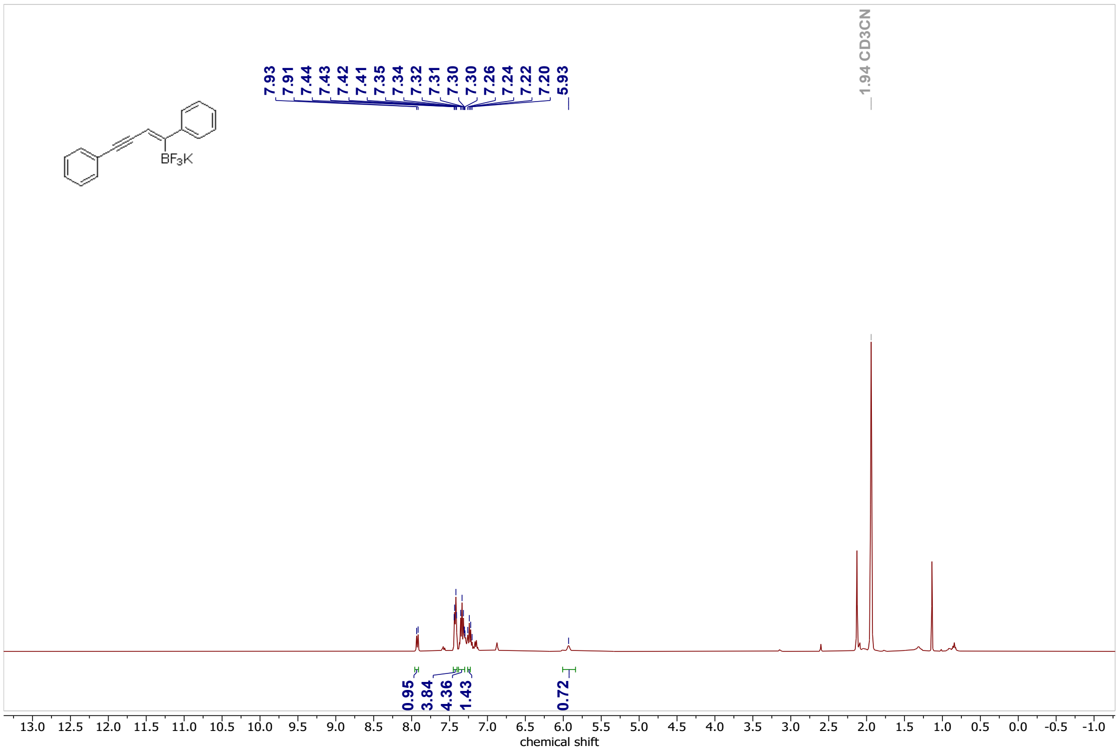

3b-^{13}C-crude

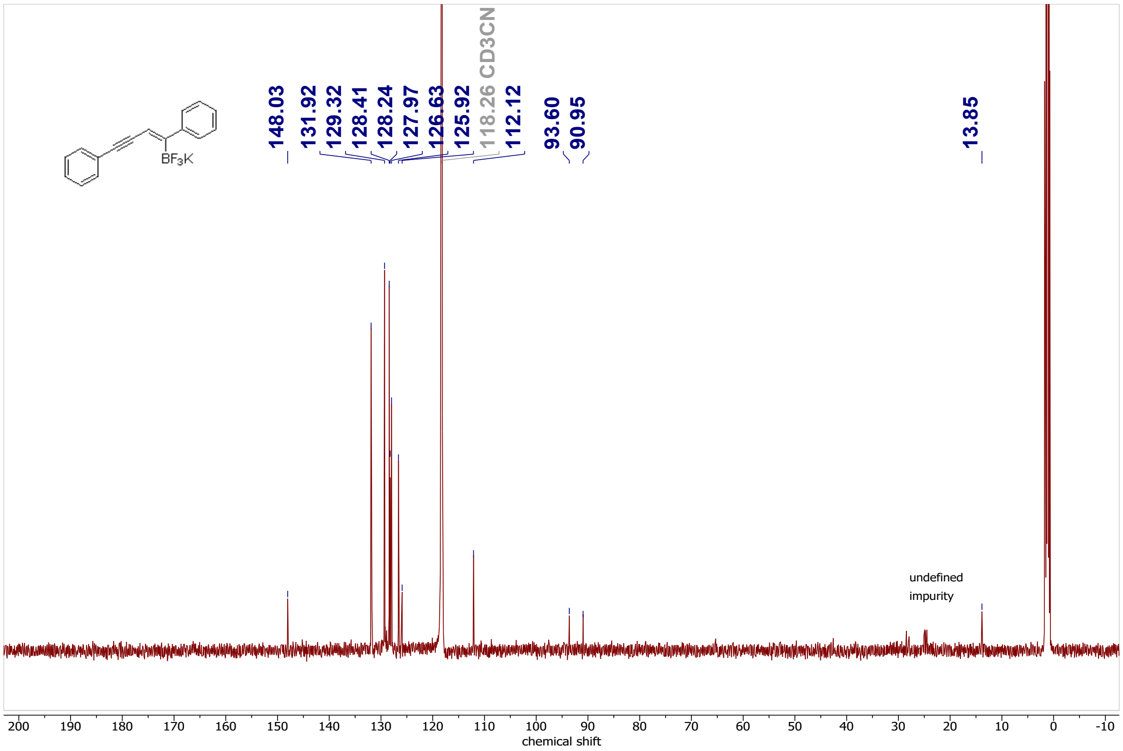

3b-^{11}B

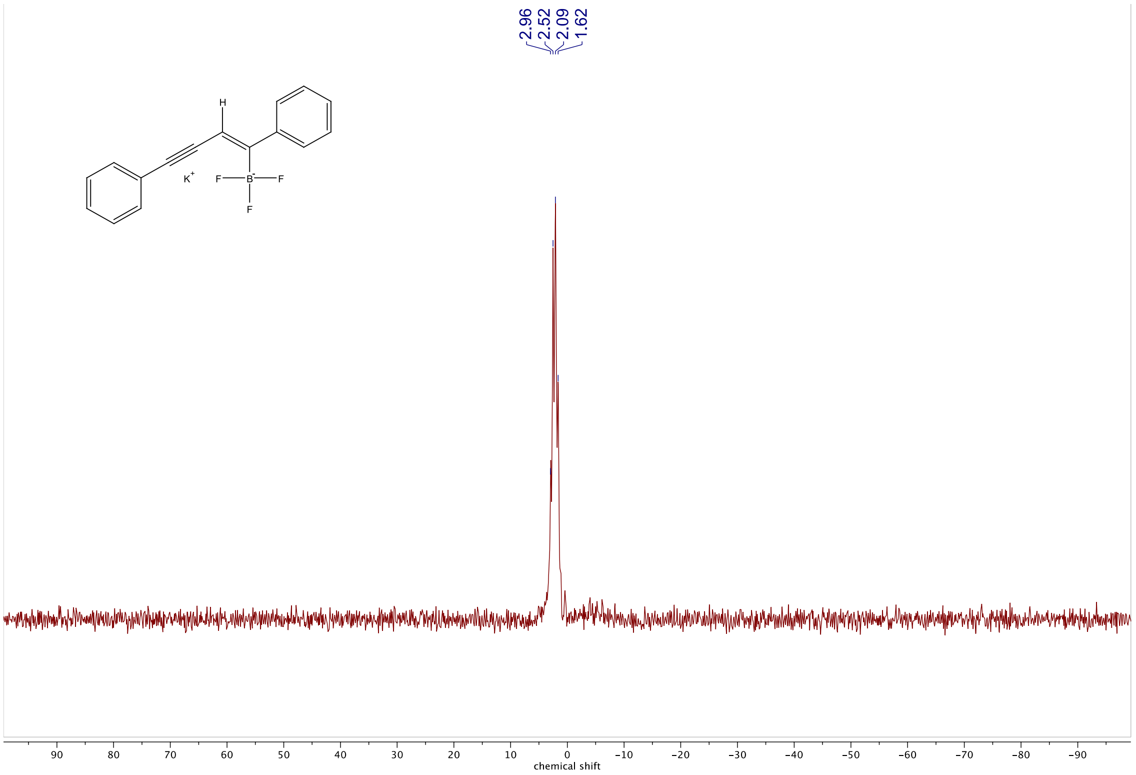

2.96
2.52
2.09
1.62
K+
F
B-
F
F
H
chemical shift

3c-^{1}H

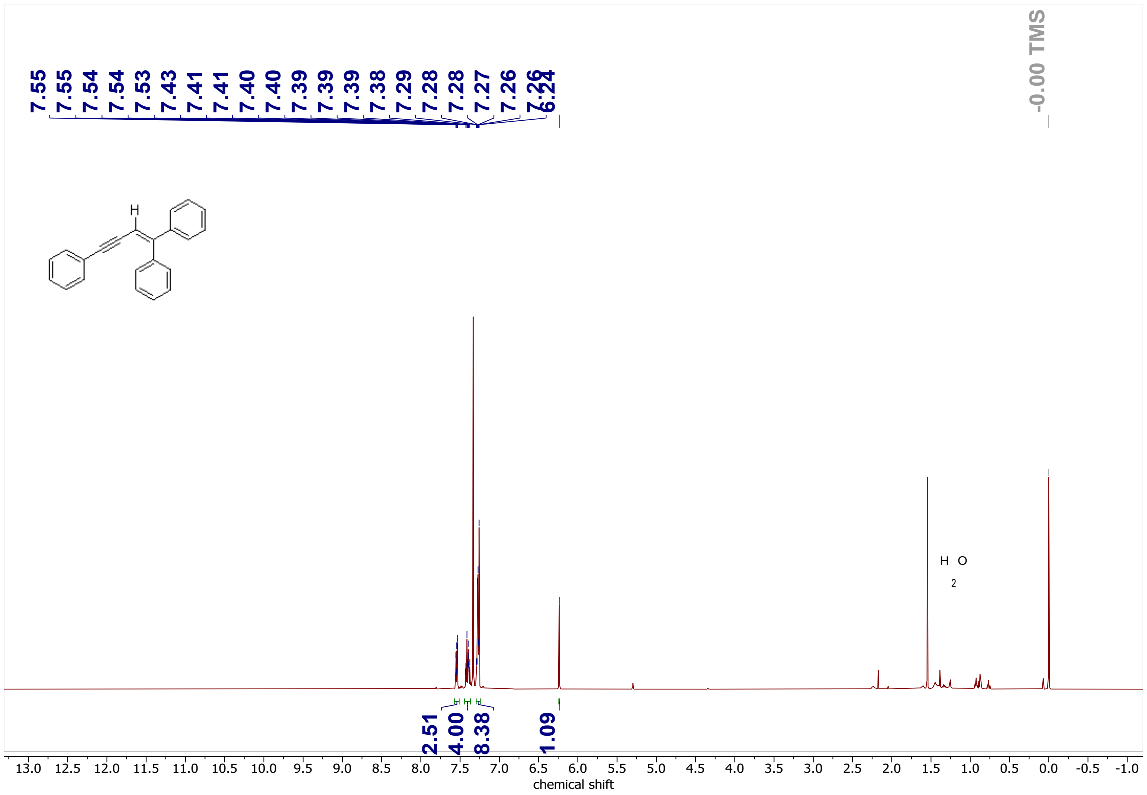

7.55
7.55
7.54
7.54
7.53
7.43
7.41
7.41
7.40
7.40
7.39
7.39
7.39
7.38
7.29
7.28
7.27
7.26
7.26
6.24
-0.00 TMS
H
H O
2
2.51
4.00
8.38
1.09
chemical shift

3c-^{13}C

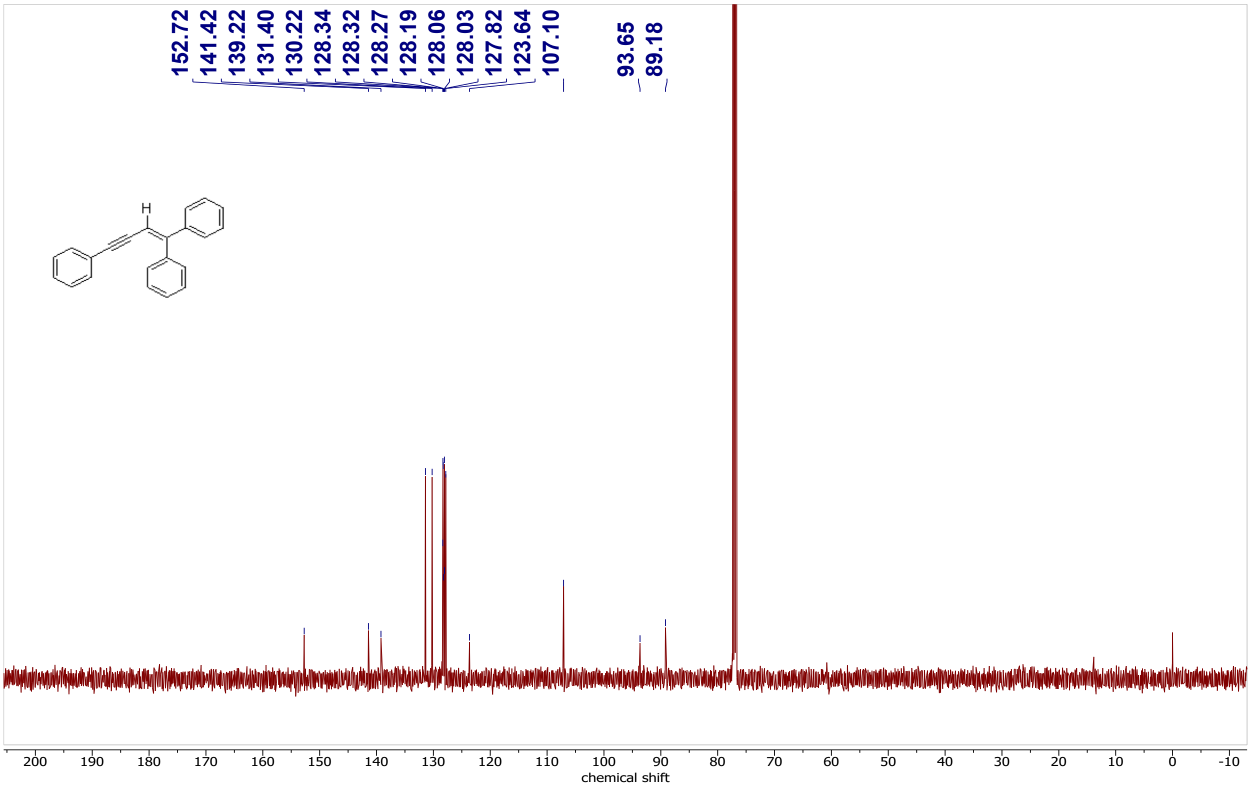
152.72
141.42
139.22
131.40
130.22
128.34
128.32
128.27
128.19
128.06
128.03
127.82
123.64
107.10
93.65
89.18
H
chemical shift
200 190 180 170 160 150 140 130 120 110 100 90 80 70 60 50 40 30 20 10 0 -10

3d-^{1}H

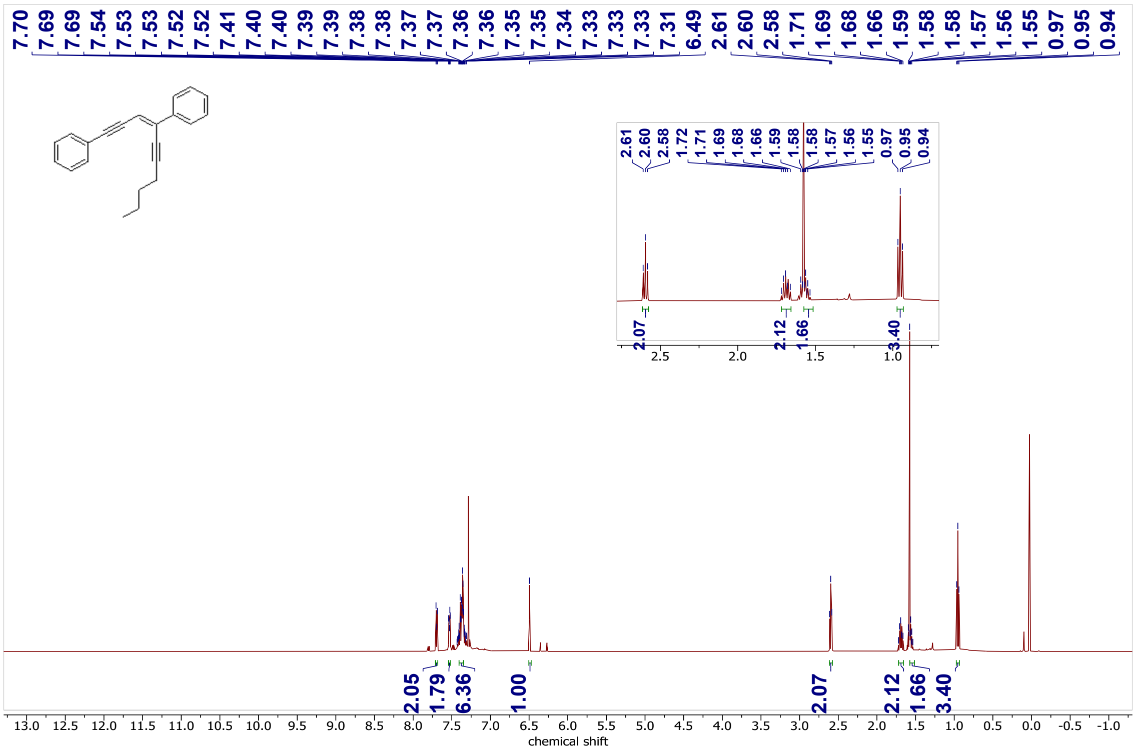
7.70 7.69 7.69 7.54 7.53 7.53 7.52 7.52 7.41 7.40 7.40 7.39 7.39 7.38 7.38 7.37 7.37 7.36 7.36 7.35 7.35 7.34 7.33 7.33 7.33 7.31 6.49 2.61 2.60 2.58 1.71 1.69 1.68 1.66 1.59 1.58 1.58 1.57 1.56 1.55 0.97 0.95 0.94
2.61 2.60 2.58 1.72 1.71 1.69 1.68 1.66 1.59 1.58 1.58 1.57 1.56 1.55 0.97 0.95 0.94
2.07 2.12 1.66 3.40
2.5 2.0 1.5 1.0
2.05 1.79 6.36 1.00 2.07 2.12 1.66 3.40
chemical shift
13.0 12.5 12.0 11.5 11.0 10.5 10.0 9.5 9.0 8.5 8.0 7.5 7.0 6.5 6.0 5.5 5.0 4.5 4.0 3.5 3.0 2.5 2.0 1.5 1.0 0.5 0.0 -0.5 -1.0

3d-[13]C

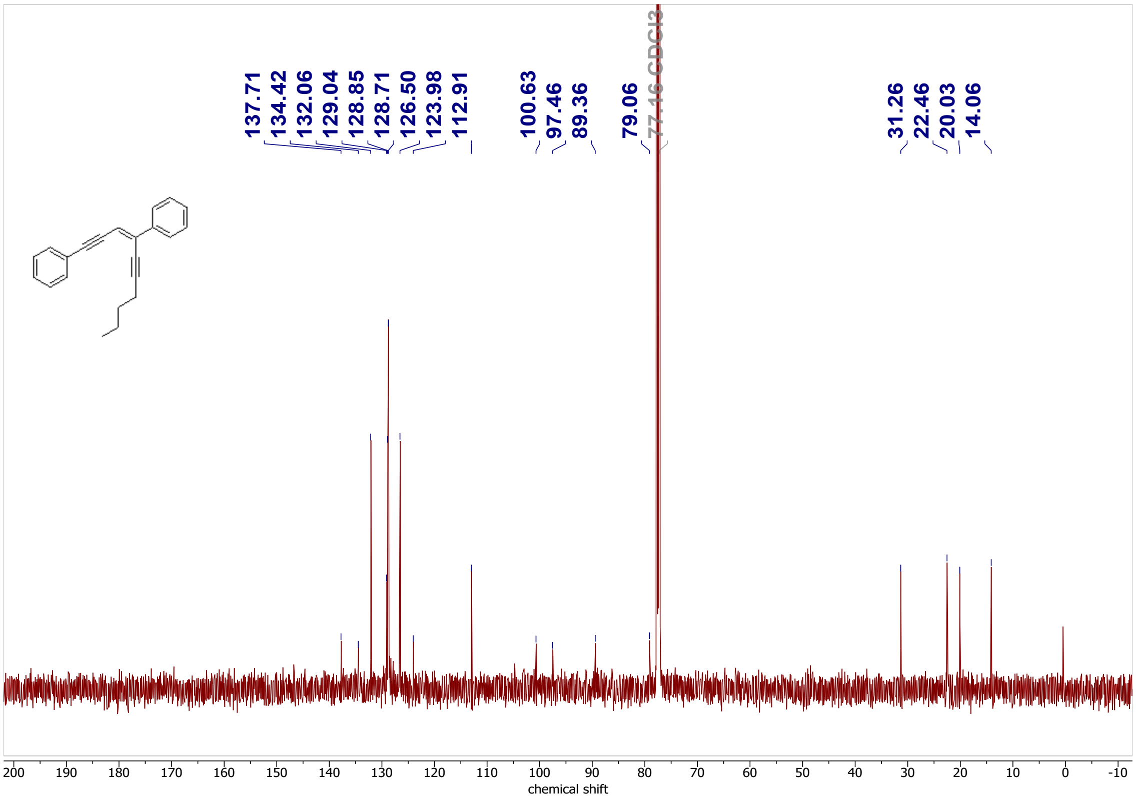
137.71
134.42
132.06
129.04
128.85
128.71
126.50
123.98
112.91
100.63
97.46
89.36
79.06
77.16 CDCl3
31.26
22.46
20.03
14.06
chemical shift

3e-[1]H

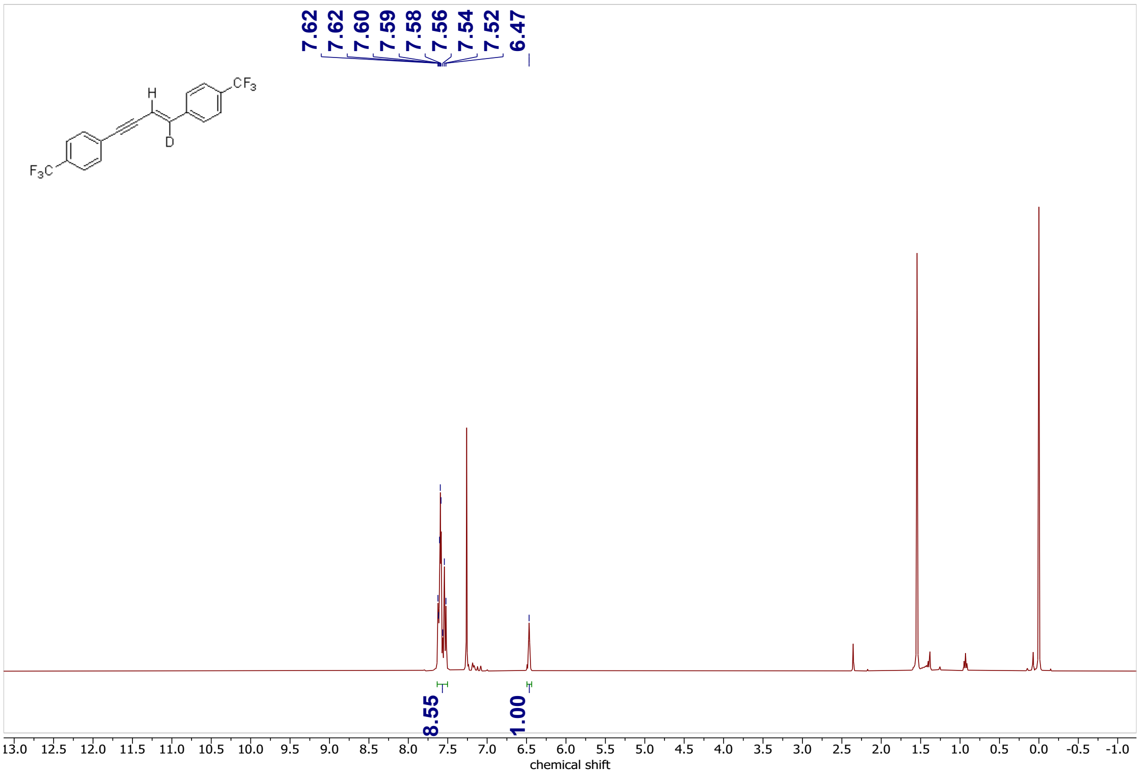
7.62
7.62
7.60
7.59
7.58
7.56
7.54
7.52
6.47
H
CF3
D
F3C
8.55
1.00
chemical shift

3e-^{13}C

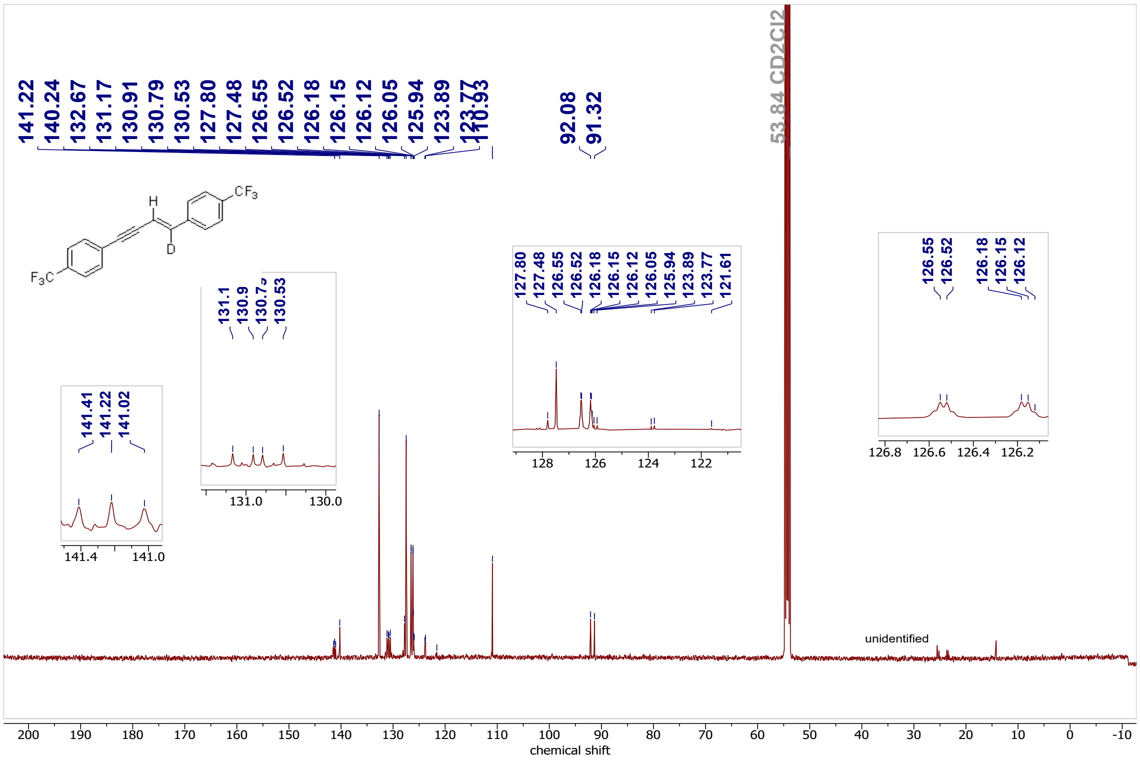

141.22
140.24
132.67
131.17
130.91
130.79
130.53
127.80
127.48
126.55
126.52
126.18
126.15
126.12
126.05
125.94
123.89
123.77
110.93
92.08
91.32
53.84 CD2Cl2
H
CF3
F3C
D
141.41
141.22
141.02
131.1
130.9
130.53
127.80
127.48
126.55
126.52
126.18
126.15
126.12
126.05
125.94
123.89
123.77
121.61
unidentified
chemical shift

3e-^{19}F

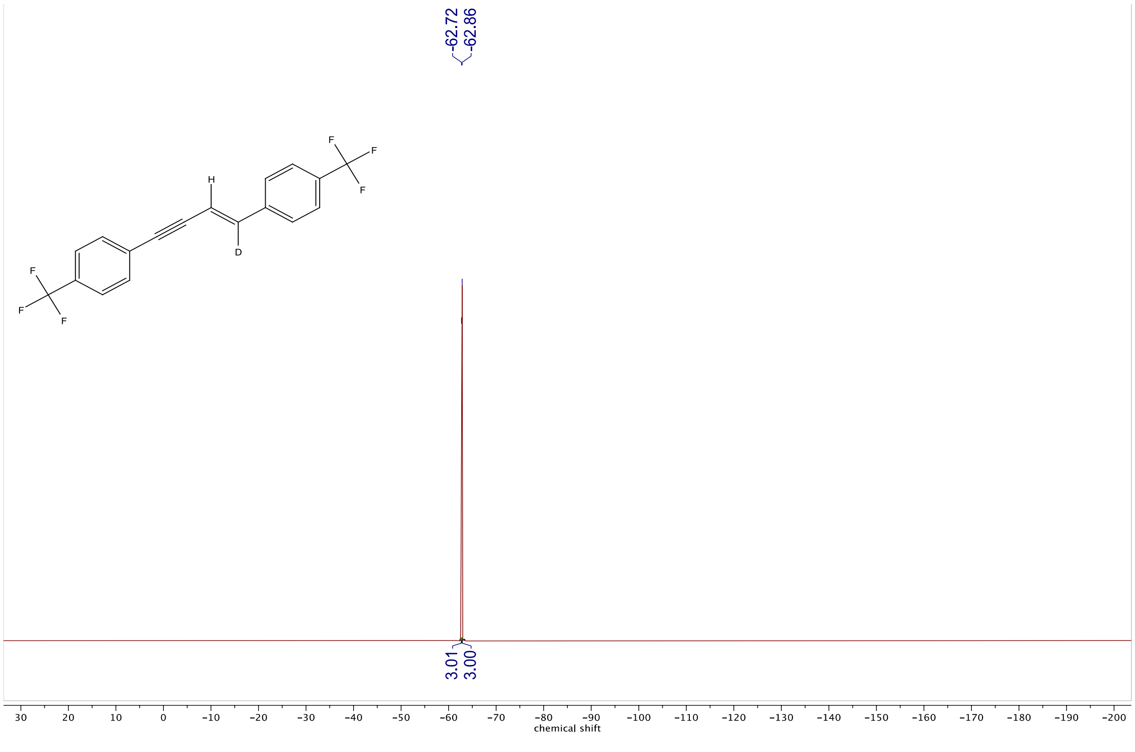

-62.72
-62.86
H
D
F
3.01
3.00
chemical shift
